# Determinants of adherence to recommendations for cancer prevention among Lynch Syndrome mutation carriers: A qualitative exploration

**DOI:** 10.1371/journal.pone.0178205

**Published:** 2017-06-01

**Authors:** Annemiek Visser, Alina Vrieling, Laxsini Murugesu, Nicoline Hoogerbrugge, Ellen Kampman, Meeke Hoedjes

**Affiliations:** 1 Radboud Institute for Health Sciences, Radboud University Medical Center, Nijmegen, the Netherlands; 2 Department of Health Sciences, VU University Amsterdam, Amsterdam, the Netherlands; 3 Department of Human Genetics, Radboud University Medical Center, Nijmegen, the Netherlands; 4 Division of Human Nutrition, Wageningen University, Wageningen, the Netherlands; King Abdulaziz University Hospital, SAUDI ARABIA

## Abstract

**Background:**

Lynch Syndrome (LS) mutation carriers are at high risk for various cancer types, particularly colorectal cancer. Adherence to lifestyle and body weight recommendations for cancer prevention may lower this risk. To promote adherence to these recommendations, knowledge on determinants of adherence in LS mutation carriers is needed. Therefore, this study aimed to identify determinants of adherence to lifestyle recommendations for cancer prevention in LS mutation carriers.

**Methods:**

Five focus groups were conducted with DNA confirmed LS mutation carriers (n = 29). Transcripts were analyzed by thematic analysis, using the Health Belief Model (HBM) as a theoretical framework.

**Results:**

Tolerance of an unhealthy lifestyle because of the desire to enjoy life and avoidance of LS dominating their life were most frequently reported as important barriers of adherence to the recommendations. Most important facilitators of adherence to the recommendations were enhancement of wellbeing and intolerance of unhealthy foods due to colon surgery.

**Conclusions:**

This study provided a comprehensive overview of determinants of adherence to recommendations for cancer prevention. These determinants, of which some are typically and unique for LS mutation carriers, can be used to design a lifestyle intervention that meets the needs of LS mutation carriers.

## Background

Lynch Syndrome (LS), also known as hereditary non-polyposis colorectal cancer (HNPCC), is an inherited cancer syndrome [1, 2]. LS mutation carriers have inactivating mutations of DNA mismatch repair (MMR) genes (MLH1, MSH2, MSH6 and PMS2) or a deletion of the EPCAM gene, which cause this autosomal dominant syndrome [1]. LS mutation carriers are at increased risk of various cancers, especially of the colorectum, endometrium, but also of the, small intestine, ovary, hepatobiliary system, sebaceous glands and urinary tract [3, 4]. E.g., the risk of developing colorectal cancer (CRC) up to age 70 years is 25–70% in LS mutation carriers [2], whereas the lifetime risk in the general population is around 5%. Furthermore, LS is characterized by early onset of cancer development. LS mutation carriers who were affected by CRC often experience long-term consequences of treatment, e.g., a reduced colon function, restrictions caused by having a stoma, or reduced energy levels [5, 6].

In the general population, approximately one third of all cancer incidences can be prevented by adherence to lifestyle recommendations such as those issued by the World Cancer Research Fund (WCRF) and American Institute for Cancer Research (AICR) [7–9]. To prevent cancer, the WCRF/AICR advices: a body weight in the normal range (Body mass index (BMI) between 18.5–24.9 kg/m^2^), physical activity on a daily basis, limited intake of energy dense foods, refined starchy food, alcohol, salt and red meat, avoidance of processed meat and sugary drinks and meeting nutritional needs through diet alone instead of relying on food supplements. In the general population, adherence to these recommendations varies between 15% (red and processed meat) and 66.2% (alcohol) [10]. Little is known about the extent to which LS mutation carriers adhere to these lifestyle recommendations.

Recent findings suggest comparable or even stronger associations between smoking, BMI, and unhealthy dietary patterns and CRC in LS mutation carriers compared with the general population [4, 7].

To promote overall health and to reduce cancer risk it is recommended to inform LS mutation carriers about the potential benefits of adhering to lifestyle recommendations [11]. However, current guidelines for clinicians do not include advice on these potential health benefits for LS mutation carriers [12, 13]. During genetic counseling, LS mutation carriers are primarily advised surveillance, i.e. biannual colonoscopy starting at age 25. Genetic counseling and cancer surveillance appointments could offer windows of opportunity for health promotion in LS mutation carriers, for example by offering lifestyle interventions. Future health promoting interventions for LS mutation carriers can best be tailored to the specific needs of LS mutation carriers, as such a tailored intervention is likely to be most suitable and effective. A needs assessment is an important first step in tailoring future interventions to the specific needs of LS mutation carriers, and includes identification of determinants of adherence to the recommendations for cancer prevention. To date, little is known about the determinants of (non-)adherence to the WCRF/AICR recommendations for cancer prevention in LS mutation carriers [14].

Theories and models of behavior change are often used to explain and predict health behaviors, and to map out determinants of health behaviors. The Health Belief Model (HBM) is a commonly used theoretical framework, which has previously been used to explain screening behaviors in populations at increased risk for hereditary cancer [15]. According to this model, determinants of (non-)adherence to a WCRF/AICR recommendation include the perceived susceptibility (i.e. the chance of getting cancer), the perceived severity (the perceived seriousness of cancer and its consequences), the perceived benefits of adherence to a WCRF/AICR recommendation, the perceived barriers of adherence to a WCRF/AICR recommendation, cues to action (triggers for prompting engagement in the WCRF/AICR recommendation), and perceived self-efficacy (perceived ability to successfully adopt and/ or maintain adherence to a WCRF/AICR recommendation) [16]. The HBM is expected to be a suitable model to explain adherence to the WCRF/AICR recommendations among LS carriers since the concepts of perceived susceptibility and perceived severity are expected to be important determinants of adherence in this particular patient population. Using this model as a theoretical framework, this exploratory study explored determinants of adherence to WCRF/AICR recommendations in LS mutation carriers.

## Methods

### Study design and participants

Focus groups were conducted from September 2014 to January 2015 to identify determinants of (non-)adherence to the WCRF/AICR recommendations for cancer prevention in LS mutation carriers. Eligible patients (n = 103) were DNA confirmed LS mutation carriers, living in the Netherlands (regions of Nijmegen or Amsterdam), who were able to read, write and communicate in Dutch. LS mutation carriers were selected from the GeoLynch study, a prospective cohort study among LS mutation carriers [17]. In response to the invitation letters (n = 103), 40 LS mutation carriers (39%) signed informed consent. Purposive sampling based on sex and age was conducted to obtain a representative sample of LS mutation carriers, resulting in a sample of 29 participants. Written informed consent was obtained from all individual participants included in the study. All procedures performed were in accordance with the ethical standards of the Declaration of Helsinki and the Committee for Human Research (CMO), Arnhem—Nijmegen region. The CMO Arnhem—Nijmegen region declared that this study did not fall under the scope of the Dutch Medical Research Involving Human Subjects Act (WMO).

### Data collection

Prior to the focus group, all participants completed a brief questionnaire to obtain data on demographic factors (education, marital status, age, gender and nationality), anthropometric factors (height and body weight), LS-related factors (year of LS diagnosis, personal and family cancer history) and smoking habits.

The focus groups were held outside the hospital setting at a university. The groups were moderated using a topic guide containing open-ended questions concerning participants’ experiences regarding their LS diagnosis, the amount of received information concerning lifestyle recommendations and their beliefs concerning the relation between lifestyle and cancer risk. Also, participants were asked to describe their perceived barriers and perceived facilitators of adherence to the WCRF/AICR recommendations. Finally, each participant was asked to mention the determinant(s) of (non-)adherence to the recommendations they perceived to be most important, with a maximum of 3 determinants per participant. The HBM was used as theoretical base in developing the questioning route (S1 Table) [16]. The topic guide was pilot tested in a group of researchers prior to the focus groups, and small adaptations to the topic guide were made accordingly.

In each focus group, the moderator (MH) guided the discussion, whereas the facilitator (AV) assisted, observed, took notes, composed a list of all determinants mentioned during the focus group on a white board, and noted the frequency with which a particular determinant was mentioned as most important. MH and AV are both trained female researchers in the fields of medical psychology and health promotion, and both have ample experience in conducting qualitative research, particularly in patient populations. The focus groups had a maximum duration of two hours and were digitally audio-recorded. The audio recordings were transcribed verbatim (S1 File).

The number of focus groups that were held was determined by data saturation, i.e., the point at which no new information or themes were observed in the data. The moderator and the facilitator discussed each focus group directly after the end of each focus group, using the notes and the list of determinants composed by the facilitator. Focus groups were held until no new determinants were observed.

### Data analysis

Thematic analysis was performed using Atlas.Ti. The analysis was conducted in six phases [18]: 1) becoming familiar with the data by reading all transcripts, 2) coding of all transcripts independently by two researchers (AV and LM), using open coding. Coding was performed first in a data-driven way, followed by a theory-driven coding approach by keeping the HBM in mind, 3) categorizing the different codes into potential themes. Two levels of themes were distinguished: main themes and sub-themes. The subthemes were discussed in group meetings between researchers (MH, AV and LM) until consensus was reached, 4) reviewing and refining themes and subthemes. First, all codes for each subtheme were checked for non-coherent patterns, which in some cases led to reconsideration of formulation and categorization of the chosen (sub)themes. This phase was finished as soon as all researchers agreed that the themes reflected the essence of the complete dataset, 5) defining and refining themes by identifying the ‘story’ that each theme told, 6) relating back results to the research questions by identifying determinants which were particularly relevant for LS mutation carriers.

## Results

Five focus groups were conducted, with 5 to 7 participants. Characteristics of the study population (n = 29) are described in Table 1. Participants were aged between 33 and 65 years (mean = 54.6±8.0). The number of years since DNA confirmed LS diagnosis ranged between 7 and 32 years (mean = 15.9±7.3). The fifth focus group yielded no new determinants, which suggested that data saturation was reached.

**Table 1:** Characteristics of Lynch Syndrome mutation carriers who participated in the focus groups (n = 29).

	**Mean (sd)**
**Age**	54.6 (8.0)
**Years since DNA-confirmed LS diagnosis**	15.9 (7.3)
	**N (%)**
**Gender**	
Male	13 (45)
Female	16 (55)
**Education level**	
Low	0 (0)
Moderate	20 (69)
High	9 (31)
**Marital status**	
Married or cohabiting	24 (83)
Unmarried, never been married	4 (14)
Divorced	1 (3)
**BMI (kg/m**^**2**^**)**	
18.5–24.9	16 (55)
≥25	13 (45)
**Smoking habit**	
Current smoker	4 (14)
Never smoker	16 (55)
Former smoker	9 (31)
**Colorectal cancer**	
Yes	8 (28)
No	20 (69)
Missing	1 (3)
**Other cancer type(s)**	
Yes	10 (35)
No	18 (62)
Missing	1 (3)
**Surgery**	
Removal of colon (colectomy)	6 (21)
Partial removal of colon (hemicolectomy)	2 (7)
None	20 (69)
Missing	1 (3)
**Colorectal cancer in 1**^**st**^ **degree relatives**^a^	
Yes	23 (79)
No	4 (14)
Missing	2 (7)
**Colorectal cancer in 2**^**nd**^ **degree relatives**^b^	
Yes	25 (86)
No	1 (3)
Unknown	3 (10)

BMI = Body Mass Index

^a^ Including parents, siblings and children;

^b^ Including grandparents, uncles and aunts

### Determinants from the Health Belief Model (HBM)

Five concepts of the HBM (perceived barriers, perceived benefits, perceived susceptibility, cues to action, and perceived self-efficacy) were reported to be determinants of adherence to WCRF/AIRC lifestyle recommendations. Table 2 provides an overview of the facilitators and barriers of adherence which were perceived as most important by LS mutation carriers.

**Table 2:** Overview of reported facilitators and barriers of adherence to recommendations for cancer prevention that were perceived as most important by Lynch Syndrome mutation carriers and the frequency and the proportion with which they were mentioned.

Facilitators	N (%)	Barriers	N (%)
A healthy lifestyle enhances the feeling of wellbeing	15 (37.5)	Tolerance of an unhealthy lifestyle because of the desire to be able to enjoy life	19 (47.5)
Intolerance of unhealthy foods due to colon surgery or other intestinal problems	9 (22.5)	LS should not dominate life	8 (20)
Living a healthy life is good for general health, it helps to stay fit and it improves physical endurance	4 (10)	Intestinal problems restrict LS mutation carriers to eat healthy or to be physically active.	4 (10)
Living healthy increases the longevity	4 (10)	Social environment stimulates a unhealthy lifestyle	5 (12.5)
Information about lifestyle act as a trigger to change lifestyle	3 (7.5)	Lack of time to live healthy	1 (2.5)
Diagnosis of cancer led to awareness of their own lifestyle	1 (2.5)	Regular colonoscopy caused perceived lower susceptibility	1 (2.5)
Being a role model to children stimulates parents own healthy lifestyle	1 (2.5)	Habit influenced unhealthy lifestyle	1 (2.5)
Influences of a regular daily routine on lifestyle	1 (2.5)	Personal preferences for an unhealthy lifestyle	1 (2.5)
The perceived relation between lifestyle and occurrence of cancer in their own social environment	1 (2.5)		
Habits influenced healthy lifestyle	1 (2.5)		

#### Perceived barriers

Tolerating an unhealthy lifestyle because of the desire to enjoy life was most frequently mentioned as an important barrier towards adherence to the lifestyle recommendations. See Table 2. LS mutation carriers reported that it became more important to enjoy and cherish every moment in life for them after the diagnosis of LS. *“…it changes life for a moment*, *you get a different perspective on life*. *[…] We should enjoy every moment*. *[…] So in our family enjoying life is a central theme*.*”*

Also, LS mutation carriers mentioned that they did not want LS to dominate their life. They perceived that constantly trying to comply with the recommendations would interrupt a carefree life, which they highly valued. Therefore, LS mutation carriers reported to have the same lifestyle as they did before LS was diagnosed.

Another barrier that was mentioned was experiencing consequences or complaints related to colon surgery or intestinal problems, such as a perceived lower energy level, an increased perceived need for salt, and rapid digestion, making it difficult to maintain weight. This led to unhealthy adjustments to their diet. For example, LS mutation carriers said: *“To have sufficient energy*, *I add some extra sugar in my coffee*.*”* LS carriers with a history of cancer mentioned consequences or complaints related to colon surgery or other intestinal problems as one of the most important barriers towards adherence to the recommendations by many LS mutation carriers (see Table 2).

Other perceived barriers included: a perceived limited availability of healthy foods (e.g. experiencing difficulties in finding unprocessed meat or alternatives for processed meat and red meat), lack of time to exercise or to eat regularly, ageing (experienced as a barrier to physical activity and losing weight), personal preferences for an unhealthy lifestyle (e.g. disliking sports, liking unhealthy food), and the social environment stimulating an unhealthy lifestyle. In addition, many LS mutation carriers experienced difficulties to adhere to the recommendations on special occasions. E.g., many LS mutation carriers treated their selves with an unhealthy snack after a regular colonoscopy.

#### Perceived benefits

A highly valued benefit of adherence to the WCRF/AICR recommendations was experiencing feelings of increased wellbeing (Table 2). Besides, LS mutation carriers mentioned the importance of adherence to these recommendations to promote general health and to stay fit or to improve endurance. Engaging in particular health behaviors to decrease their increased cancer risk was not mentioned as an important benefit. Moreover, LS mutation carriers explicitly emphasized that LS was not a motivator to change their lifestyle. LS mutation carriers did mention an increased longevity as a benefit of living a healthy lifestyle. *"Well*, *I have had cancer and I want to live longer*, *that is very important to me*.*"* Maintaining a slim figure was also mentioned as an important benefit of a healthy lifestyle.

#### Perceived susceptibility to the development of cancer

Due to regular colonoscopies, LS mutation carriers reported to perceive a decreased susceptibility to develop cancer. These regular checkups were considered as reassuring. LS mutation carriers even perceived that their susceptibility for cancer was lower compared to non-LS mutation carriers, who do not have these regular checkups. Perceived susceptibility was also influenced by their experiences with lifestyle and the occurrence of cancer in their social environment. For example, perceiving individuals in their social environment with an unhealthy lifestyle who did not develop cancer influenced their thoughts on the relation between lifestyle and cancer and decreased their perceived susceptibility. One LS carrier said: *“My grandmother smoked one package of cigarettes every day and she became 91*.*”* On the other hand, perceived susceptibility increased if individuals in their social environment with an unhealthy lifestyle were affected by cancer, making them more aware about the importance of their own lifestyle. Another LS carrier mentioned: *“When my sister had surgery*, *[…]*, *my first thought was instantly*: *do I live healthy*?*”*

#### Perceived self-efficacy

While some LS mutation carriers reported to be confident that they could adhere to lifestyle recommendations, others reported low levels of self-efficacy. For some participants, meeting the WCRF/AICR recommendations for cancer prevention was considered to be too demanding. Others reported a lack of skills to be able to adhere to the recommendations.

#### Cues to action

Several triggers or cues to action for adherence to the recommendations were mentioned. LS mutation carriers reported that receiving information or gaining knowledge about the relation between lifestyle and cancer acted as a trigger to change their lifestyle. Although it became apparent during the focus groups that most LS mutation carriers did not receive any information about the influence of lifestyle factors on cancer risk, some LS mutation carriers mentioned that they had searched for information themselves, which had led to lifestyle changes. One of the participants said: *“I also read a lot of leaflets about cancer and you receive a lot of information through the media*. *[…] This triggers me to reflect on my lifestyle each time*.*”* At the time of the focus groups, many LS mutation carriers were unaware of the relation between lifestyle and cancer and of the WCRF/AICR recommendations. They learned about this during the focus groups. Some realized during the focus group that they did not comply with some of the recommendations. Some LS mutation carriers said that their awareness of the influence of lifestyle on the occurrence of cancer and awareness of the WCRF/AICR recommendations was perceived to be a cue to action to improve their lifestyle. However, for others it was not perceived as a cue to action. Some of the LS mutation carriers who had been diagnosed with cancer, stated that receiving a cancer diagnosis increased their awareness of their own lifestyle, and had led to an increased motivation to change their health behaviors, and to actual improvements in their lifestyle. Finally, the desire to be a role model for their children stimulated LS carriers’ own healthy lifestyle.

### Other determinants

Besides the five concepts of the HBM, three other determinants of adherence to WCRF/AIRC lifestyle recommendations were reported: perceived facilitators of adherence to the WCRF/AICR recommendations for cancer prevention, habits, and perceived locus of control in relation to cancer risk.

#### Perceived facilitators

Intolerance of certain unhealthy foods due to the consequences of colon surgery or other intestinal problems was reported as a facilitator of adherence to the WCRF/AICR recommendations. Another reported facilitator was a personal preference for a healthy lifestyle, such as liking fruits and vegetables or being a sporty type. Finally, LS carriers reported that a regular daily routine made it easier to adopt and maintain a healthy lifestyle.

#### Habit

Adherence to the WCRF/AICR recommendations was perceived to be easier if a healthy lifestyle was considered to be a habit. *“Every morning I start with eating fruits and hazelnuts*. *I won’t skip a day*.*”* Contrary, unhealthy habits made it more difficult to adhere to the WCRF/AICR recommendations.

#### Perceived locus of control

LS mutation carriers mentioned different perspectives about their perceived locus of control in relation to cancer risk. Some had an external locus of control and accepted having no control at all on their cancer risk. They considered receiving a cancer diagnosis to be an accidental coincidence or bad luck. Moreover, other LS mutation carriers expressed a strong believe that one cannot decrease cancer risk by adhering to the WCRF/AICR recommendations. They felt that lifestyle changes would be useless. One of these LS mutation carriers stated: *“Well*, *I can eat 10 kilos of fruits a day*, *but it will not change anything*, *right*?*”*. Others reported an internal locus of control, and believed that they could decrease cancer risk by living a healthy lifestyle, which motivated them to adhere to lifestyle recommendations. As one participant stated about influencing cancer risk: *“…I will try to do anything I can…”*.

## Discussion and conclusions

This first explorative qualitative study on the determinants of adherence to the WCRF/AICR recommendations for cancer prevention showed that the Health Belief Model (HBM) can partly be used to explain adherence to these recommendations in LS mutation carriers. In addition to determinants from the HBM, habits, perceived facilitators, and perceived locus of control in relation to cancer risk were found to be determinants. These determinants were not captured by the HBM, as this model mainly assumes that health behaviors are conscious and controlled decisions, while often behaviors are determined by combinations of individuals’ habits, emotions, attitudes and unconscious or non-rational reactions [19]. Moreover, not all determinants from the HBM were mentioned as determinants of adherence to WCRF/AICR recommendations during the focus group interviews. LS mutation carriers did not explicitly mention perceived severity of cancer as a determinant, as assumed by the HBM.

As this is the first paper on determinants of adherence to WCRF/AICR recommendations in LS mutation carriers, and the number of papers on lifestyle behaviours in LS mutation carriers is limited, the results of this study are placed into a broader perspective, and compared with findings in other high-risk populations and results from studies performed in populations prior to genetic testing.

Receiving a LS diagnosis was not reported as an important determinant of adherence to lifestyle recommendations. However, it should be noted that at the time these participants received their diagnosis (7 to 32 years ago, with a mean time since diagnosis of 15.9±7.3 years), they did not receive any information on the potential cancer risk-reducing effects of adherence to WCRF/AICR recommendations. Receiving a LS diagnosis made participants realize the importance of enjoying life (as long as possible), which was actually found to be a barrier to adherence to WCRF/AICR recommendations. When participants did receive information about the influence of lifestyle on the occurrence of cancer, and about the WCRF/AICR recommendations, this was perceived as a cue to action for some LS mutation carriers. However, other LS mutation carriers mentioned that LS and adherence to lifestyle recommendations should not dominate their lives. Previous findings on the influence of genetic counseling on lifestyle behaviors are inconclusive. Some studies reported no association between increased familial or hereditary breast cancer risk and improved lifestyle behaviors [20, 21]. Whereas others reported a positive association between perceived cancer risk and likelihood of adopting a healthy lifestyle in families at risk for hereditary colorectal cancer, including patients and family members [14, 22, 23]. In line with a previous study prior to genetic testing in at-risk families, having a personal history of cancer or having family member(s) who suffer(ed) from cancer were determinants of adherence to lifestyle recommendations [22].

Although having LS did not directly lead to adherence to WCRF/AICR recommendations, some LS mutation carriers did (try to) have a healthy lifestyle to improve their general health and wellbeing. In accordance with these findings, previous research in healthy women who received genetic counseling for *BRCA*-mutations also found that improvement of general health and wellbeing was an important determinant of a healthy lifestyle [24]. Having LS seemed to affect several lifestyle behaviors more indirectly, since LS mutation carriers mentioned several determinants which were closely related to or consequences of having LS (e.g. experiencing consequences or complaints related to colon surgery or other intestinal problems).

Perceived locus of control of cancer risk varied widely among participants. This variety of beliefs about the effect of lifestyle changes on cancer risk may be influenced by the limited information provision on this topic by medical professionals. Our findings that an external locus of control of cancer risk was reported to be a barrier to adherence to WCRF/AICR recommendations, and that receiving information on the WCRF/AICR recommendations and their influence on cancer risk was reported to be a cue to action, suggest that creating awareness of the association between lifestyle behaviors and cancer risk could be a first step to promote adherence to WCRF/AICR recommendations in LS mutation carriers [25].

### Strengths and limitations

The qualitative design of the study enabled us to collect a comprehensive overview of relevant determinants, without the risk of missing determinants caused by pre-selection of determinants by researchers, as would be the case in quantitative research. Another strength of our study was that the validity of the results was constantly checked by discussion of the determinants in group meetings until consensus was reached. In addition, coding was performed by two researchers separately, which enabled us to detect and discuss differences in interpretation of quotes. Finally, the number of participants and focus groups in our study is according to guidelines on the size of focus group studies [26]. According to these guidelines, a focus group study typically comprises 3 to 5 focus groups with a range of 4 to 12 participants, with a typical group size of 6 to 10 [26]. Our study consists of 5 focus groups, with 5 to 7 participants per focus group, and a number of 29 participants in total, which is in line with these guidelines.

The following limitations need to be taken into account. Our study sample consisted of participants diagnosed ≥6 years ago. Therefore, the results of this study cannot be generalized to individuals who were more recently diagnosed with LS. Future research should be focused on more recently diagnosed LS mutation carriers as these persons might report different determinants. Moreover, since our study sample consisted of relatively older, moderate or higher educated LS mutation carriers, future research should also explore determinants in lower educated and in younger LS mutation carriers. Another limitation that should be addressed is a possible selection-bias. Although, we provided limited information on the content of the focus groups in the invitation letters for this study, it is possible that selection of LS mutation carriers with an interest in lifestyle occurred. However, given the large variety of reported determinants and the number of LS mutation carriers who reported not to be focused on living a healthy lifestyle in daily life, such selection seems unlikely. Since data was collected anonymously, quotations could not be related to particular participant characteristics (e.g., personal history of cancer).

### Practice implications

Our findings suggest that at least a part of the LS mutation carriers would like to receive information about the WCRF/AICR lifestyle recommendations and their potential influence on cancer prevention. Our findings also suggest that receiving this information could be a cue to action for improving lifestyle. However, in current clinical practice such information is not yet provided to LS mutation carriers. Therefore, an important first step in promoting adherence to WCRF/AICR lifestyle recommendations in LS mutation carriers would be to inform LS mutation carriers during counseling or surveillance appointments.

Our findings also suggest that LS mutation carriers vary in their readiness to make lifestyle changes in response to receiving information on these lifestyle recommendations. Readiness for change should be taken into account in attempts to promote a healthy lifestyle in LS mutation carriers since each stage of change requires different behaviour change techniques (e.g. creating awareness vs. providing practical advice)[27]. Clinicians (e.g. clinical geneticists) are in a favourable position to assess this readiness for change and the potential need for a lifestyle intervention to promote adherence to the WCRF/AICR recommendations.

For LS mutation carriers who are ready to make lifestyle changes, but would like to receive support to be able to do so, a lifestyle intervention could be offered. To achieve an optimal intervention effect, and to promote participation and compliance, it is important that a lifestyle intervention offered to LS mutation carriers meets the needs of this particular patient population. To our knowledge, such a targeted lifestyle intervention for LS mutation carriers does not yet exist. The determinants of adherence to WCRF/AICR lifestyle recommendations that were identified in this study can be used to tailor future interventions to the specific needs of LS mutation carriers. For example, future interventions might focus on learning LS mutation carriers how to deal with the consequences or complaints related to colon surgery or other intestinal problems in a healthy way, e.g. by providing suggestions for healthy alternatives to unhealthy dietary adjustments in case of low energy levels. In addition, since our findings suggest that LS mutation carriers don’t want to be constantly remembered about their increased cancer risk and related recommended lifestyle changes, an intensive lifestyle intervention would be less suitable for this particular patient population. A low-intensity intervention that easily fits into daily routines may be more appropriate. Finally, the variation in determinants between LS carriers observed in our study underline the importance of personalized lifestyle promotion for LS carriers, tailored to the specific needs of each LS carrier.

### Conclusions

This study provides a comprehensive overview of determinants of adherence to lifestyle recommendations, of which some are typically and unique for LS mutation carriers. Tolerance of an unhealthy lifestyle because of the desire to be able to enjoy life was most frequently reported as important barrier of adherence to the WCRF/AICR lifestyle recommendations by LS mutation carriers. Also, LS mutation carriers frequently mentioned that they did not want LS to dominate their life. Most important facilitators of adherence to these recommendations were enhancement of wellbeing and intolerance of unhealthy foods due to colon surgery or other intestinal problems.

The results of this study underline the importance of health education and the potential benefits of health promotion in LS mutation carriers, and can be used to promote adherence to lifestyle recommendations for cancer prevention in this population.

## Supporting information

S1 Table Questioning route. 1 = perceived susceptibility; 2 = perceived severity, 3 = perceived benefits, 4 = perceived barriers, 5 = cues to action, 6 = self-efficacy.
(DOCX)

S1 File Transcripts focus groups. (PDF)
