# Supplementary material for: Determinants of adherence to recommendations for cancer prevention among Lynch Syndrome mutation carriers: A qualitative exploration
Source: PLoS One. 2017 Jun 1;12(6):e0178205. doi: 10.1371/journal.pone.0178205 (PMC5453435; doi:10.1371/journal.pone.0178205)
Supplement: S1 Table — 1 = perceived susceptibility; 2 = perceived severity, 3 = perceived benefits, 4 = perceived barriers, 5 = cues to action, 6 = self-efficacy. (DOCX) [file pone.0178205.s001.docx]

**Supplementary Table I.** Questioning route

| **Question** | **HBM concept(s)** |
| --- | --- |
| 1. **What kind of information or support did you receive at the moment you received the LS diagnosis?** |  |
| - Was the received information comprehensive? Did you miss any information? What kind of information did you want to receive? |  |
| - Have you ever received information about a healthy lifestyle in relation to the risk of cancer or Lynch syndrome? |  |
| - Where did you receive this information? |  |
| - Was the information clear to you? If not, which aspects were unclear and what made them unclear? |  |
| - Did this information lead to lifestyle changes? |  |
| 1. **What is, according to you, the importance of receiving information related to lifestyle and the influence on cancer risk?** | 2, 1, 3 |
| 1. **Did you try to change your lifestyle in your past?** |  |
| **If yes:**   - Why did you want to improve your lifestyle (motivation)? | 3, 1, 2 |
| - Which lifestyle behaviour did you try to change? |  |
| - What made you succeed in changing your lifestyle? | 6, 3, 5 |
| - How long did you maintain these changes? |  |
| - What made you fail to improve your lifestyle? | 6, 5 |
| - How did you maintain improvements in your lifestyle? | 6, 3, 5 |
| - What caused that you were unable to maintain the improvements in your lifestyle? | 6, 5 |
| **If no:**   - What is the reason you never changed your lifestyle? | 6, 4 |
| ***Description of AICR/WCRF recommendations for cancer prevention by moderator*** | |
| 1. **Were you aware of these recommendations?** |  |
| - To which recommendation(s) do you adhere? |  |
| - On which recommendations is room for improvement in your personal lifestyle? |  |
| - At this moment, after seeing these recommendations, how motivated are you to improve your lifestyle? |  |
| - Which lifestyle behaviour(s) would you like to change? |  |
| - At this moment, what causes your motivation to improve your lifestyle? | 3, 4, 5, 1,  6 |
| - What kind of support could help you to improve your lifestyle? What kind of support would help you in maintaining these changes? | 5 |
| - Which determinants have the strongest effect on your lifestyle behaviours? (Give a top 3 of most important factors). |  |

^1= perceived susceptibility; 2= perceived severity, 3= perceived benefits, 4= perceived barriers, 5= cues to action, 6= self-efficacy^
