# Supplementary material for: Determinants of adherence to recommendations for cancer prevention among Lynch Syndrome mutation carriers: A qualitative exploration
Source: PLoS One. 2017 Jun 1;12(6):e0178205. doi: 10.1371/journal.pone.0178205 (PMC5453435; doi:10.1371/journal.pone.0178205)
Supplement: S1 File — (PDF) [file pone.0178205.s002.pdf]

Transcripts focus groups Visser et al PONE-D-16-46517

Transcript focus group 1: page 2 - 36
Transcript focus group 2: page 37 - 64
Transcript focus group 3: page 65 - 99
Transcript focus group 4: page 100 - 127
Transcript focus group 5: page 128 - 154

Correspondence:
Meeke Hoedjes, PhD
VU University Amsterdam
Faculty of Earth and Life Sciences
Department of Health Sciences
Room T-636
de Boelelaan 1085
1081 HV Amsterdam
E-mail: m.hoedjes@vu.nl

Focusgroep 1 Amsterdam

Context/ Wat voor informatie heeft u gehad?:

Gesprekleidster: Hoe lang u al weet dat u Lynch syndroom drager bent? Misschien kunt u heel even kort iets over u zelf vertellen, wat u kwijt wilt. Hè, dat maakt verder niet zo heel veel uit. Zal ik met u beginnen?

M: Ja is prima. um Waar zal ik mee beginnen? Ik um naja mijn opa stierf op zijn vierenveertigste als gymleraar niet rokend niet en sportleraar en um hij had vier dochters. Daarvan zijn er drie overleden aan, na ja, buikpijn zal ik maar zeggen. En dus sindsdien begin jaren 90 omdat ik uit zo'n familie kom ben ik, um dat was hier achter ook nog in *** um huisje daar was de geo en omdat ik uit die familie kwam hebben ze me toen gevraagd om bloed te geven. En gingen ze daarmee uitzoeken waar het uiteindelijk bij *ZKH1* terecht was gekomen. Dus eigenlijk wist ik het al van toen af aan. Je ziet zoveel in je familie hebt dan denk je ja dat is toch wel heel veel. 0.19

Gesprekleidster: Ja en wanneer was dat? Dat u er achter bent gekomen?

M: um naja mijn moeder stierf in ‘80 ofzo ‘79. En ze wisten al vrij snel en mijn zus al vrij snel daarna, dus toen was er al een vermoeden. Toen begin ‘90 was het onderzoek en ik denk dat in ‘94 ofzo ze er achter gekomen zijn. Toen kwamen ze met de computers ofzo. Ze dachten dat het heel lang zou duren, want het was één lettertje in het telefoonboek zeiden ze, dat ze zochten het verschil. Dus naja sindsdien weet ik het wel al ja en sindsdien doe ik ook elk jaar het onderzoek zeg maar. Um alleen kwam er een complicatie in 2000 en um toen had ik opeens erg veel buikpijn en toen was ik hier bij de *ZKH2*. En toen zeiden ze oh nee het is een maagzweer. Dat ging maar niet over, nou nog meer antibiotica. Toen werd ik uiteindelijk geel en toen bleek dus dat het um het HSE, syndroom Lynch, zich ook in het twaalfvingerige darm kon um. Ja, dus toen was het eigenlijk al te laat. Je weet alleen maar natuurlijk, ja. En toen heb ik een whippleoperatie ondergaan. En dat betekent eigenlijk dat ze alles weghalen en weer bij elkaar plakken. Dat is dan je galblaas, een stuk pancreas, onderkant van je maag en dat plakken ze allemaal aan je dunne darm. Um ja dus wat dat betreft mijn um opvatting naar aanleiding van voedsel en leefstijl wel ietsje anders dan misschien bij de anderen. En wat betreft de informatie heb ik eigenlijk nooit informatie gekregen tot nu toe. Er waren op een gegeven moment wel verhalen maar nooit echt via de de instanties zeg maar. Ik bedoel je leest er natuurlijk naar en ja je komt er wel achter dat het rood vlees vinden ze dan slecht voor je, schijnt slecht te zijn. En groente, fruit en dat soort dingen is dan weer goed zeggen ze.

M: Zeggen ze

Gesprekleidster: zeggen ze

M: Nou ja goed, dat geldt voor iedereen.

Gesprekleidster: Maar daar komen we zo meteen ook nog even op terug. Op um hè, de informatie

M: Naja, toen na die operatie ach ja goed die wisten ook niet zo goed aan dat eigenlijk die operatie voor pancreas kanker is ontwikkeld. En die hebben niet zo'n grote kans van overleven, maar omdat ik natuurlijk die andere achtergrond heb. Um is het nu al 14 jaar geleden en terwijl na die operatie is het volgens mij operatie maximaal 5 dus. Ja, en dat verandert toch een beetje je houding van kwaliteit van het leven ten opzichte van wat je laat staan en dat soort dingen.

Gesprekleidster: Ja, zeker

M: Um na ja, qua informatie ook na de operatie met die diëtiste het is um. Ik denk dat ze zelf ook niet zo veel weten nog wat dat betreft. Er is me wel geadviseerd om veel vezels te eten en dat je massa in beweging te houden. Dus ja eigenlijk heel weinig ja.

Gesprekleidster: Oké, En jij, zou jij iets?

V: Ja hoor nou um ik ben *V1* ben 42. En ik weet sinds 2005 dat ik um erfelijk belast ben met het Lynch de

MHL1 en um mijn vader is daaraan overleden in ‘75. Mijn opa, mijn oom en tante leven nog, maar die hebben meerdere keren kanker gehad. Mijn neef is overleden in 2001. En inmiddels ben ik zelf nu ook twee keer getroffen, in 2011 en 2012 in m'n dikke darm. Nou die heb ik zo goed als laten verwijderen. Ik heb vorig jaar preventief mijn baarmoeder, baarmoederhals en eierstokken laten verwijderen. En ja, hier zit ik dan. Ja um. Niet heel veel informatie over voeding mee gekregen. Nee, alleen inderdaad vezels eten. Ik loop wel bij een diëtiste, die begeleidt mij wel heel erg goed moet ik zeggen.

Gesprekleidster: En is dat ook in het ziekenhuis, of is het..?

V: Nee

Gesprekleidster: Gewoon los van het ziekenhuis

V: Ja, los van het ziekenhuis en eigenlijk op eigen initiatief, ja dus dat heb ik ook niet meegekregen. En ja, zij vindt mij sowieso een interessant geval. Ik sta wel in nauw contact met de internist en de diëtiste, want ik ben nu weer met de medicatie bezig. Ook om dat voedsel wat steviger te krijgen, dus we zijn wel daarmee bezig. Maar het is wel heel weinig wat je krijgt, lijkt wel alsof ze het inderdaad niet weten.

Gesprekleidster: Ja ja, oké en hoe um hoe is dat voor jou?

M: Ik ben *M1* ik ben 51. Eigenlijk weet ik pas sinds begin de jaren ‘80 dat in ieder geval in onze familie erfelijk kanker voorkomt. Oma, tantes, ooms, mijn broer is in ‘83 overleden ook aan dikke darm kanker. En eigenlijk vanaf dat moment ben ik regelmatig onder controle geweest. Ik denk dat ik ook aan hetzelfde onderzoek als *?* heb meegewerkt begin jaren ‘90.

M: Hierbeneden ergens

M: Hier bij de *ZKH2* ook en um bloed gegeven en um inderdaad 2, 3 jaar later um kregen we uit *ZKH3* gedaan denk ik, kreeg ik het bericht van nou het is gevonden en we weten dat dit het is. En opnieuw bloed geven om um voor de zekerheid um dat er geen vergissingen werden gemaakt. Maar dat was voor mij heel belangrijk om het te weten, want je kan dan kiezen, wil je het wel weten of wil je het niet weten. Um als je kinderen hebt denk ik dat het handig is om dat te weten van jezelf, zodat zij weten dat ze het risico lopen daarop. Zeker omdat mijn broer ook pas 23 was dat die dood ging. En um de afgelopen paar jaren heb ik zelf inderdaad ook um zeg maar paar keer tijdens het onderzoek zijn er kwaadaardige plekken weggehaald. Nou gelukkig niet zo ver dat er al um andere acties ondernomen moeten worden. Dus ik heb alles nog.

V: Ik was ook zwanger op dat moment.

Gesprekleidster: Oh, nee toch

Meerderen: oh ja ja

V: ".." dus dat gebeurt_ #00:07:30-5#

M: En ja wat ik te horen heb gekregen is eigenlijk van dat ja als je maar voldoende regelmatig controleert. Dan hebben we het idee dat we er op tijd bij zijn, dat het vooral, ja, dan kunnen we de kleine plekken weghalen en we testen dat en ja is het kwaadaardig, dan is het kwaadaardig, maar we hebben we alles weg. Dus ik heb een tijdje elke drie maanden het onderzoek gehad, dat is niet fijn.

Meerderen: Zo ja, pff ja

M: Maar ja aan de andere kant, ik heb alles nog.

V: Ja, ik was wel blij want ik heb nu alleen sigmoïdoscopie #00:07:57-9# dat was nog het enige blijdschap die ik had.

Gelach

M: Ja, soms word je blij met dingen waarvan je zou denken dat je er niet van verwacht dat je er blij mee zou zijn.

V: Ja, ja eerlijk is eerlijk

M: Dus inmiddels ben ik overgestapt na het *ZKH1* voor het onderzoek. En die geven eigenlijk aan dat ze met de techniek die zij nu beschikbaar hebben het wel aan durven om het eens in het jaar te doen in plaats van eens in drie maanden

Gesprekleidster: Oké, dat is mooi

M: Ik ben nu weer terug na een jaar, dat vind ik wel spannend, maar..

Meerderen: ja

M: Maar goed, um, ze hebben daar voldoende ervaring, want zo'n onderzoek is best wel belastend elke keer weer.

Gesprekleidster: oké, en hoe was het bij jou?

V: Ja, um ik kom uit een gezin van 13. Ik ben de een na jongste en van de 13 waren er 8 drager. En ik ben de enige die nog geen kanker ontwikkeld heeft. En er zijn ook al een aantal dus eraan overleden op jonge leeftijd. Ik heb in 2007 preventief mijn baarmoeder en eierstokken laten weghalen. Um ik denk dat wij een beetje in dezelfde categorie zaten, want ik weet ook sinds 1994 dat ik drager ben. En ook al vijf jaar daarvoor de eerste familie gesprekken gehad en um weinig ook over voeding. Inderdaad ook dat over rood vlees, dat is denk ik toentertijd misschien erin gehamerd van denk aan dat rode vlees. Verder eigenlijk ook weinig wel van beweging.

M: Nou ben ik met mijn um zoon, die is nu 19, ben ik hier geweest vorig jaar die is dus ook zeg maar drager van het gen. Toen kreeg ik van degene die het onderzocht had eigenlijk een verhaal van ja weet je wat het is, als je um dat lynch syndroom niet hebt dan heb je tussen de 0 en 4% kans op kanker in je dikke darm te ontwikkelen, he dus um statistisch gezien. Na ja, jullie als drager zitten wel op 4, 75%. Dus ga lekker barbecueën want of je nou 74% of 77% kans hebt, dat verschil zien wij niet zo. Dus dat werd daarmee ".." vorig jaar was dat zo. 10.00

V: Ik ben.. mijn dochter is ook draagster en daar hebben we eigenlijk geen informatie gehad over voeding. En dat is net drie jaar terug denk ik.

M: Nee, de informatie die ik heb was van nou gewoon lekker doen wat je doet want dat veel is toch niet zo..

V: Ja dat heb ik ook gehad.

M: Ja?

V: Je voelt zelf wel wat je wel kan hebben en wat je niet kan hebben. .. dan ga je automatisch minder mee bij wijze van spreken. Dus ja, maar rood vlees is mij eigenlijk nooit gezegd, van ja het is vervelend als je rood vlees gegeten hebt als je daarna onderzoek hebt of ontlastingsonderzoek dan kunnen ze moeilijk kijken van he zit er bloed in of zit er geen bloed in. Maar voor de rest hebben ze mij eigenlijk nooit gezegd van je mag geen rood vlees eten

V: Ik heb wel begrepen van rood vlees, misschien kunnen jullie het me beter uitleggen of dat er een bepaalde stof in zit of een bepaalde verbinding maakt waardoor de kans op bloedvorming sneller is.
#00:10:59-0#

M: het sap wat het afbreekt dat dat juist ".." #f1 00:11:02-8#

V: ja ik weet niet precies hoe dat zit dat um.. #00:11:08-8#

M: maar ik begrijp van jou dat je gewoon doet en laat wat je wilt

Gelach

M: Ja..

Gelach #00:11:14-1#

M: Dat is misschien ook wel het verstandigste, want je kan ook heel ergens anders aan dood gaan natuurlijk.

V: Daarom, geniet van het leven. 00.11

Gesprekleidster: Hoe lang weet jij al *V2* dat je drager bent?

V: Daar heb ik nu al ontzettend over na zitten denken. Ik ben dan um als .. #00:11:36-6#
Ik ben 58, bijna 59. En het begon eigenlijk in 1977 dat mijn nicht overleed van 21 met hersentumor. En hersentumor komt dus heel weinig voor in verband met Lynch syndroom. Um toen kreeg mijn oom het. Um mijn oma werd ziek. Mijn vader werd ziek. Mijn broer werd ziek. Ze zijn inmiddels allemaal overleden. En de één is overleden aan baarmoederkanker, dat is mijn oma dan, waar het dus ook vandaan komt het Lynch syndroom. Mijn vader is overleden aan hersentumor. Mijn broer is overleden aan darmkanker. Um de vader dan van mijn nicht die heeft het alle twee gehad, darmkanker en hersentumor. Dan heb ik nog twee tantes en de één heeft borstkanker gekregen en is daar niet aan overleden, maar aan een hartstilstand. En mijn andere tante is overleden aan hersentumor. Dan heb ik nog een andere oom die is ook overleden aan darmkanker. En dan hebben we dit jaar is mijn neef overleden aan hersentumor. Vorig jaar is mijn neef ook geopereerd aan "galgang" kanker. Dus het is heel divers. 00.12.

Meerderen: Nou, ja inderdaad

V: Dus alles wordt ook bij mij hier in het *ZKH2* eigenlijk onderzocht en bij mij is het begonnen ergens in de begin jaren '90. Dokter *naam*

Meerderen: Ja

V: Dat je je bloed moest laten onderzoeken van de hele familie. Dus alles ging dan inderdaad naar *ZKH3* toe. En toen ben ik een tijdje uit beeld geweest. En iedereen werd onderzocht en toen ben ik gaan opbellen van waarom word ik nou eigenlijk niet onderzocht. Nou ze waren mij eventjes kwijt, is niet erg, maar op dat moment kon ik dus op bloed onderzocht worden of ik het nou wel had of niet had. Mijn twee broers die nog leven, die hadden het gelukkig niet. En toen heb ik me laten prikken en ik heb het wel. En um, nou ja, ik had zoiets van ik wil toch gewoon goed gecontroleerd. En geniet van het leven, we zijn er maar even. En um er is nog steeds geen kanker bij mij geconstateerd. In 2004 is wel mijn baarmoeder en eierstokken preventief weggehaald. En dan heb ik om de drie jaar een hersenscan. En om het anderhalf jaar heb ik een endoscopie. #00:13:51-5# En dan heb ik ook nog even voor de urinewegen geloof ik maar dat is alleen een urineonderzoek. En dat is ongeveer um ja wat ik heb. Net wat ik zeg je eet ja wat je lekker vindt maar ik ben geen zoetekauwer dus zoveel suikers zal er bij mij niet binnen komen. En um voor de rest ben ik gek van groentes. en um vorig jaar heb ik tijdelijk een hormoonbalans dieet genomen, waar me ontzettend goed bij voel. Maar um ik had een hele zware galblaas ontsteking, daar heb ik jarenlang mee gelopen. En um daarna ben ik eigenlijk niet meer mee verder gegaan. #00:14:36-9#

M: Wat is een hormoonbalans dieet? #00:14:40-0#

V: Ja dat is van één of andere dokter hier uit *ZKH1*. En dat um ik ik ik werd steeds dikker dikker dikker

dikker. Ik ben nou inmiddels al 10 kilo afgevallen, ik ben nog steeds aan het afvallen maar dat gaat heel langzaam. Maar door die ontsteking die ik had, die galblaas ontsteking, werkte mijn schildklier niet meer. Maar dat was net 1 punt te hoog ik zat op um 12 en als het 11 is krijg je medicijnen. Dus ja ik groeide maar. Dus dan ga je gewoon dingen zoeken. Het hormoonbalans dieet is eigenlijk een heel lekker dieet want je mag eigenlijk heel veel eten. Behalve het is geen brood. En het brood wordt vervangen door rijstwafels en op die rijstwafels mag je allemaal lekkere dingen doen. Helemaal super!

Gelach

V: heerlijk rauwkost met tonijn enzo, allemaal lekkere dingen. Dus het is niet zo dat je weinig eet. Het heet ook zo, het is een dieet zonder honger, maar je valt er wel van af op één of andere vage manier. En um ik heb het niet extreem gedaan, want dan heb je speciaal brood en speciaal dit en dat is allemaal te veel toestand. Dus ik heb het half half gedaan. En daar voelde ik me eigenlijk wel heel erg goed bij. En het is inderdaad niet veel genoemd.

Gesprekleidster: Oké, nou interessant. Ja, ik had er ook nog nooit van gehoord.

V: even googlen

Gesprekleidster: Ja Oké..Hé en um wat voor informatie heeft u gehad, he, op het moment dat u te horen of je, sorry hoor, dit zal ik wel vaker gaan doen, dat ik “u” erdoor heen gooi. Op het moment dat je te horen kreeg dat je drager was van het Lynch syndroom, wat voor informatie um heb je toen zoal gekregen?

M: Nou, ik helemaal niets eigenlijk. Behalve dat je je af en toe moet laten controleren en dat was toen nog met die ".."onderzoek enzo #00:16:54-4#

M: Dat is van vorig jaar of niet?

V: En jouw zoon ook niet, dus blijkbaar is dat nog steeds niet zo dat um er geen enkele informatie is op um voeding

V: Mijn twee dochters worden wel hierin het *ZKH2* gecontroleerd, maar ze zijn er nog niet op geprikt. Dat hebben we bewust gedaan, omdat dat toen nog een beetje lastig was met hypotheken aanvragen en verzekering.

M: Ik heb daar nooit last van ondervonden hoor

V: Nee maar maar daar werden wij toen voor gewaarschuwd. Ik zei toen van nou jullie staan toch hartstikke goed onder controle laten we het maar doen. Er zijn wel bij alle twee zijn er wel poliepjes bij weggehaald. Bij de jongste is er vorig jaar dan een onrustig poliepje zoals ze het dan noemen weggehaald.

V: Hoe oud zijn ze?

V: *V3* is 32 en *V4* is 30. Dus ze hebben zelf zoiets van met 35 laten we het prikken.

M: Maar iedereen kan natuurlijk kanker krijgen en het voordeel van als je je niet laat prikken is dat je altijd gecontroleerd wordt en geen risico "..". En op het moment dat je je wel laat prikken en je hebt het niet word je ook niet meer gecontroleerd en kun je wel kanker ontwikkelen maar komen ze er niet zo snel achter. #00:17:59-2#

Gelach Meerderen: Ja

V: Ja maar, ik praat er ook goed met mijn dochters erover hoor. We maken er ook helemaal geen probleem van. Het is geen heisa ofzo. Ja, het onderzoek is even een heisa, even verstand op nul en we gaan het gewoon weer door#15029110 .#

M: Ja, dat zijn afwegingen hè? Waarover je eigenlijk niet over geadviseerd wordt hè, van moet je je nou wel laten prikken, moet je je niet laten prikken. Wat zijn de consequenties daarvan?

Wat voor informatie heeft u gemist?

Gesprekleidster: Dat is eigenlijk de volgende vraag inderdaad ook. Is er nou informatie die u gemist heeft? Hè, is er bepaalde informatie die u graag had willen hebben tijdens die..

M: Ja het is makkelijk dat je niet alles zelf hoeft te verzinnen. Als je ook gewoon wordt aangereikt, over dat soort vraagstukken.

V: Nou, met *naam dokter* hebben we daar wel geloof ik een uur of twee uur over zitten praten hoor.

M: Ja, of je het wel of niet wilde weten.

V: nee, nee ook voor mijn dochters

M: met kinderen

V: ook met mijn dochter erbij, van laten we nou wel prikken of laten we nou niet prikken. Wat zijn de voors, wat zijn de tegens?

M: Maar toen was hij er zelf niet

V: Maar *Dr.1* zelf is er niet meer, maar dat was toentertijd zo.

M: Ja

V: Ik weet ook eigenlijk niet meer sinds ik wanneer ik het weet, ik denk zoiets van 2000. Omdat ik natuurlijk eerst tussenuit gevallen was. Anders had ik het veel eerder geweten. #00:19:11-5#

V: Wat ik wel als heel moeilijk ervaren heb, naar aanleiding dan van dat bloedprikken. Bij ons hadden ze dus ook allemaal bloed geprikt. Mijn jongste zus eigenlijk een beetje onder druk van, naja ze had vier kinderen en um het zou toch wel wenselijk zijn dat ik weet dat je wel of niet drager bent. Zij bleek dus ook draagster te zijn, maar is het alternatieve "circuit" opgegaan. En uiteindelijk dus ook overleden op 48 jarige leeftijd. En dat vond ik heel moeilijk om mee om te gaan van um, van wat zij de keuze bewust gemaakt had van geen onderzoek, alternatief. En daar zo bespraakt in was en zo overtuigend kon vertellen dat je het bijna ging geloven. #00:19:57-2#

Meerderen: Ja #00:20:01-4#

V: En pas nadat zij um overleden was dat is dan 7 jaar terug, besefte ik wat voor druk mij dat gegeven heeft in al die jaren dat ik wist dat ik drager was. En iedere keer dat ik zag van ... waarom deed je nou ik geen onderzoek? Dat was lastig. #00:20:21-8#

Gesprekleidster: Dat is toch ook voor iedereen een afweging die je moet maken, hè, zelf? #00:20:24-4#

V: Ja, ja ik heb het ook wel is aangekaart hoor bij de *ZKH2*, en de *ZKH2*. Wist dat natuurlijk ook wel. Maar dan kom je enorm op het gebied van privacy en dat snap ik ook wel. En vanuit de specialisten kant snap ik dat helemaal heel goed. Maar als dat zo dichtbij mij is, dan is dat bijna niet te snappen. Dan dan zie je eigenlijk um iemand um mogelijkerwijs van je wegglijden. Dat is nou eenmaal zo. #00:20:50-5#

Gesprekleidster: Ja, kan ik me voorstellen.

M: Ik geloof wat ik me nog kan herinneren, is dat ik lang geleden volgens mij was het *Dr.1* inderdaad die zij van ja als je kinderen dan 18 zijn, voor 18 gebeurt niets, dus daar hoef je ze niet mee lastig te vallen. Op het moment dat ze 18 zijn kan je het erover hebben. Dan zijn ze in principe volwassen genoeg om daar zelf over

te gaan nadenken hoe ze daar mee om willen gaan. En als iemand beslist om het te negeren, ja wat kan je daar van zeggen. #f0 21:15-7#

M: Ja, of wel het onderzoek laten doen maar niet laten prikken. #00:21:15-3#

V: Je probeert alleen de opvoeding nu mee te nemen, hoe klein ze ook zijn. Gaandeweg nadat ze 18 zijn, dat ze dan zeggen: mam, gaan we? #00:21:29-2#

Gelach #00:21:29-5#

M: Joepie #00:21:29-5#

V: Nou, ik weet wel dat ik dat heel moeilijk vond, dat mijn kinderen.. van.. 25 was voor mij een beetje een magische grens. Dus toen ze dat nog 16, 17 waren heb ik heel veel tantes zeggen, ah dat duurt nog wel even kan het nog wel uitstellen. Ja, totdat de grens van 20, 21 kwam en dat de vraag kwam van, goh, mam ik wil me wel laten onderzoeken. Waar moeten we zijn? Ja, ja, ja komt wel. Dat ga ik nog wel opzoeken, dat was van dat ik er moeilijk mee had. Hun waren wel al zo ver, maar ik niet. Ik wilde hun dat op één of andere manier nog niet mee belasten. Alle zorg daarin mee um. #och8-3#

M: Ja, maar ja aan de andere kant, ik was wel blij dat ze allemaal onderzocht worden. #00:22:13-3#

V: Ja, dat is dus de andere kant. #00:22:15-5#

M: Want mijn oudste zoon is ouder dan mijn broer geworden. #00:22:21-4#

V: Ja, ja dat is ook zeker de andere kant. #00:22:24-0#

V: Maar dat is ook een raar gevoel vind ik. Dat je als mens gaat overleven. Hè, je wordt ouder dan je vader, je wordt ouder dan je broer. En um je wordt ouder dan je ooms en je wordt ouder dan je tante. Dat is wel een raar gevoel vind ik. Terwijl er nog best wel jong allemaal mensen overlijden. Ik vind het wel een raar gevoel. #2:48-8#

M: Ja, en op een gegeven moment denk je dan ben ik "home" free zeg maar. Dat dacht ik namelijk ook tot drie jaar terug en toen kwam op een gegeven moment toch nou juist bij zo'n onderzoek van ja is toch niet goed. En dat was wel een klap, want ik dacht wel van eigenlijk um kijk uiteindelijk kunnen ze met die percentages goochelen wat ze willen, maar je krijgt het wel of je krijgt het niet. Dus dan is het eigenlijk altijd fifty - fifty. #00:23:06-1#

Gesprekleidster: Ja precies, dat is het inderdaad ook hè. Dat is hetzelfde met de invloed van leefstijl. Je krijgt het of wel of je krijgt niet, dus je kunt wel percentages krijgen maar je bent, ja, je krijgt of wel of niet. Dus dat ja blijft heel lastig hè, om die percentages dan te interpreteren ook. Hè, van wat kun je nou daar dan mee? Hè, dat um.. #00:23:25-9#

M: Ja, ik heb zes nichten en een neef van die kant zeg maar, of nee 8 en daarvan hebben, 6 um zijn positief. Maar ja, die ene neef die het niet had die is nu dood want die had multimum ".." of zoiets, die had een soort leukemie. #00:23:40-9#

Gesprekleidster: Tjeetje #00:23:40-9# Ja, ja #00:23:41-9#

M: Het is gewoon allemaal ".." is al wat. #00:23:43-2#

V: Datzelfde verhaal heb ik eigenlijk ook. Wij waren dan met 8 dragers en er was dus al heel veel gebeurd. Ja en dan is er één niet-drager en die is 51 en die krijgt een hersentumor. Zonder enkele kans. #h:56-7#

V: Dan ben je nog steeds overtuigd dat er een link moet zijn, dat ze nu nog niet weten. Dat bestaat gewoon niet, dat geloof ik niet. Dan is er zoveel gebeurd. Hij is niet drager. #00:24:09-6#

M: En ik vraag me dan af op het moment dat hè, zoals in jouw familie het veel voorkomt en iemand is niet drager, maar ook nog niet getest daarop dus die wordt wel elk jaar gecontroleerd, kun je dan nog wat in vroegtijdig stadium doen? "Dus waar doe je wijs aan?" #00:24:21-3#

V: Bij ons waren het wel dragers. #4:22-9#

M: Ja oké, maar stel dat het niet zo is word je ook niet meer gecontroleerd. En dan kun je het toch ook ontwikkelen.   #00:24:30-0#

V: Nou, ik ben de enige die um regelmatig dan een MRI krijgt voor mijn hersenen. Omdat ze ook niet snappen hoe dat gerelateerd wordt aan het Lynch syndroom. Het is wel heel raar dat degene die het Lynch syndroom hebben dan dat wel ontwikkeld hebben, dus ze snappen er nog helemaal niets van. #00:24:48-2#

Meerderen: ja, ja

M: Er zijn ook variaties waarschijnlijk.

Gesprekleidster: Wat dat betreft is er..

M: Zoals bij mij met mijn twaalfvingerige darm, ik krijg dan ook nog een gastro er ook nog bij. Ja ik heb laatst ook begrepen in maag daar zit ook ".." #00:25:00-7#

V: Volgens mij had een oom van mij ook in de twaalfvingerige darm. #00:25:08-4#

M: Ze zeiden tegen, dat is 3%   #00:25:10-2#

V: Mijn broer die dan overleden is, die had het aan de dikke darm en de dunne darm en uitzaaiingen aan de lever op een gegeven moment en dan zit het op een gegeven moment overal natuurlijk. #00:25:21-6#

M: Dan is het gauw klaar. #00:25:24-4#

V: Ja, dat mijn broer blaaskanker kreeg en ook daaraan overleden is. Toen was het gevolg dat wij ook allemaal een blaasonderzoek gingen doen. #00:25:34-5#

M: Die zijn niet fijn, hè? #00:25:34-5#

V: Wat zegt u? #00:25:34-5#

M: Die zijn niet fijn, die blaasonderzoek *lachend* #1 :25:36-5#

V: Um misschien dat voor mannen dat het anders is   #00:25:38-3#

Gelach #00:25:39-9#

Gesprekleidster: Ja natuurlijk, ja #00:25:44-1#

V: Ja ik ook #00:25:44-1#

M: Ik heb ook één keer gedaan en toen dacht ik van nou dat doen ze maar op een andere manier. #00:25:49-1#

V: Ja en dan zeggen ze u doet één keer in het jaar een onderzoek, maar dat vergeet ik ook wel eens, hoor. #00:25:53-2#

Gesprekleidster: Ik wil even naar de volgende vraag gaan. Probeer een beetje op het schema te blijven *lachend* #00:26:01-4# We hebben het daarnet over gehad hè, dat jullie zeiden, vertelden net dat jullie eigenlijk weinig informatie hebben gekregen over leefstijl. Um, in hoeverre zouden jullie het belangrijk vinden om daar wel informatie over te krijgen? #00:26:16-7#

M: Nou, voor me zelf niet meer, omdat ik het eigenlijk zelf heb uitgevonden. Maar ik denk dat het wel handig is om gewoon iets te hebben waarvan je zegt, waarvan je keuzes kan maken # 0:26:43-1#

Gesprekleidster: Ja #00:26:43-1#

M: Dus die keuzes maak je toch wel. Of je bent er wel of niet in geïnteresseerd, of je negeert het. #00:26:47-9#

V: Ik geloof dat jij het net al eerder aangaf, op een gegeven moment kom je ook voor keuzes te staan. Ja jongens we kunnen overal wel aan gaan denken en je leefstijl enorm gaan aanpassen. Wat dan ook weer een hele grote invloed op je leven krijgt, waardoor je er altijd maar mee bezig bent. #00:27:04-2#

M: mee bezig bent ja #00:27:04-2#

V: En *zucht*, ja, het komt zoals het komt. Na ja, precies, weet je wel #00:27:09-4#

M: En één keer per jaar is al meer dan genoeg als je voor zo'n onderzoek enzo moet #00:27:11-5#

V: Precies #00:27:11-5#

Gesprekleidster: Maar zegt u daar mee dan ook dat het voor u niet zo belangrijk is om um om daarover geïnformeerd te worden? Over, over #00:27:21-9#

V: Ja, zeker wel. Tuurlijk, die informatie dat is altijd goed. Kijk, ik bedoel als ik alle dagen dik aan de patat zit en um heel vet ga eten, maar dat is voor iedereen goed. #00:27:34-2#

Gesprekleidster: Niet specifiek voor.. #00:27:35-6#

V: Ik denk wel van dat rood vlees, vlees eten. Dat denk ik wel en ik denk sowieso um dat het hele westerse levensstijl dat die mogelijk misschien wel invloed heeft. Als je nou kijkt vanuit de Japanners, die daar veel meer vegetarisch eten of veel meer vis eten. Waardoor veel minder darmkanker in ieder geval daar voorkomt. Dat daar misschien wel een link zit. #00:28:00-9#

M: Ja maar de Japanners gaan uiteindelijk ook dood. #00:28:04-3#

V: Ja, dat snap ik. #00:28:02-7#

Gelach #00:28:02-8#

Gesprekleidster: uiteindelijk allemaal, hè #h:05-6#

V: De mensen die om de Middellandse zee leven dat daar minder darmkanker is. En ik heb zoiets van die mensen eten wel heel veel tomaten, zou het door die tomaten komen dan? #00:28:21-1#

V: Ik eet ze elke dag. Tomaten zijn heel gezond.

M: Of gaan ze gemiddeld vroegtijdig aan andere dingen dood, waardoor ze niet die kankeronderzoek hebben. #2#

V: Nee, nee ze kunnen behoorlijk oud worden. Dus ik heb er wel aan zitten denken. Ik eet zelf ook veel tomaten, maar dat komt omdat ik het ontzettend lekker vind. Dus dat is um het zijn voor mij net snoepjes.

Dus dat is geen probleem. Maar um, daar heb ik wel is aan zitten denken. Dat er misschien toch een bepaalde stof inzit of weet ik veel allemaal. Dus eet heel veel tomaten. #00:29:00-0#

M: en olijfolie #00:29:00-0#

V: en olijfolie #00:29:03-4#

V: Ja, mijn moeder die is 79 geworden. Ze is dus inmiddels ook al twintig jaar dood. En een echte West-Friese vrouw, gewoon van het platteland. En die aten gewoon alles wat los en vast zat. Precies, gewoon een normale eter. Niet zozeer van alle extraatjes. #00:29:20-7#

Gesprekleidster: Van tegenwoordig eigenlijk, hè? #00:29:21-7#

V: Van tegenwoordig heel erg is. En zij was een enorme kaaseter en dat heb ik dus ook wel gedaan. Ik vind kaas sowieso erg lekker, totdat natuurlijk bleek dat mijn cholesterol heel erg hoog was. #00:29:36-9#

Gelach #00:29:36-9#

V: Ja maar, snap je? haha #00:29:41-2#

V: ja, ja en dan ben je nog zo slank. #00:29:43-7#

V: Ja, maar dat kwam doordat ik de kaas gestopt heb. #00:29:48-2#

M: Ja, van kaas zet je wel aan. #00:29:49-9#

V: Hè? #00:29:49-9#

M: Van kaas zet je wel aan. #00:29:51-2#

V: Ja #00:29:56-3# #00:30:01-8#

Gesprekleidster: En *V*, hoe is dat voor jou bijvoorbeeld, zou jij het belangrijk vinden om informatie over leefstijl te krijgen?

V: Ja natuurlijk, ja natuurlijk. Ja maar dan kijk ik wel hoe ik het in pas in mijn leven. Ik zal niet er niet naar naar gaan leven. Ik zal wel kijken hoe ik het zal inpassen, want anders ben ik er teveel mee bezig. # 0:30:19-7#

Aanpassen leefstijl:

Gesprekleidster: En voor jou, V1? #00:30:22-2#

V: Ja, ik ben net nu paar weken begonnen om echt um goed um te kijken naar wat ik eet. Drie keer op een dag wat eten en proberen daarbij te houden telkens. Ik heb wel problemen van die dikke darm die ik niet meer heb. Nu ben ik er ook pas aan toe om heel goed te kijken. En ik houd het bij. Net zoals mijn plas en urine. Ik maak er een heel dagboek van zeg maar momenteel. Maar dat is puur om.. #00:30:49-3#

V: Maar daar kan ik me wat bij voorstellen. #00:30:51-4#

V: Ja en dat is puur om me even weer te kunnen oppakken. Nou, als ik het had geweten had ik echt wel voor die stoma gegaan, dat zeg ik heel eerlijk. #00:31:06-4#

M: Ja, toch wel? #3#

V: Ja, ja, ja en fatsoenlijk een dag weg kan ik niet. Ik moet weten waar de toiletten zijn en dat soort dingen. Of ik eet niet, zonder eten doe je dan toch niet heel optimaal. Nee, dan ik dat echt gedaan, als ik nog een

keer getroffen word. #00:31:24-6#

V: Je hele dikke darm is verwijderd, hè? #00:31:24-6#

V: Zo goed als. #00:31:29-2#

V: Ja, een groot deel van ons die geopereerd zijn hebben dus ook die operatie gehad. En ik zeg dan ben helemaal verbaasd. Hoe is het mogelijk dat jullie dat allemaal weg krijgen. Dan heb ik een knoflooksaus op tafel staan en de bak knoflooksaus wordt gewoon leeg gegeten. #00:31:44-9#

V: Ja, in principe kun je wel alles eten natuurlijk. Alleen wel met gevolgen. #00:31:55-2#

Gesprekleidster: En hoe..? #00:31:55-2#

M: Het duurt ook wel even denk ik, weet niet maar dat is. #00:31:57-3#

V: Ja, dat gaat snel hoor. #00:31:56-8#

M: Maar die dunne darm neemt vrij veel functies over op een gegeven moment. #00:32:01-5#

V: Ja kijk, je vitamine dat blijft allemaal in de dunne darm, dat behoud je allemaal wel op zich. Um maar bij mij gaat het vrij snel, ga toch wel 12 tot 15 keer, als ik normaal eet. #00:32:20-8#

V: Het heeft grote invloed. #00:32:22-0#

V: Het heeft absoluut invloed. #00:32:25-4#

Veranderingen in leefstijl:

Gesprekleidster: En hoe zit het met um met de leefstijl, hè, in het verleden, hebben jullie in het verleden geprobeerd om jullie leefstijl te verbeteren bijvoorbeeld? #00:32:36-6#

M: Verbeteren? #00:32:39-4#

Gelach #00:32:42-9#

Gesprekleidster: Te veranderen, hè, na ja goed, hè, kan zijn van niet inderdaad. Dat het al of goed of goed genoeg is, voor jullie gevoel. Dat het niet beter kan worden. #00:32:57-2#

V: Ben wel gestopt met roken. #00:32:58-4#

Gesprekleidster: Oké, ja #00:32:58-5#

V: Ik moet nog stoppen. #00:33:01-2#

Gelach #00:33:01-2#

Gesprekleidster 2: Wanneer bent u gestopt?

V: Maar, ik merk wel dat ik um, dat het toch wel een behoorlijke invloed heeft op je ontlasting. #00:33:12-0#

V: ja, ja

Gesprekleidster 2: Wanneer heeft u besloten om te stoppen met roken? #00:33:14-9#

V: Um 2007, nou 7 jaar gestopt. #00:33:22-3#

Gesprekleidster: Ja, goed #00:33:21-3#

V: En daardoor je moeilijker nu naar de WC gaat? #00:33:21-8#

V: Nee hoor, nee nee, helemaal niet. Toen had ik eigenlijk gewoon dagelijks diarree, dat heb ik nu niet meer. Daarom zei ik toch wel een grote invloed op mijn darmen. #00:33:24-6#

Gesprekleidster: Was er nog een bepaalde aanleiding voor dat u op dat moment gestopt bent? #00:33:39-8#

V: Ja, ik werd oma. Mijn dochter werd zwanger, ik denk die mag geen rokende oma komen. #00:33:44-9#

Gesprekleidster: Ah #00:33:44-9#

V: Dus eigenlijk nog niet eens voor het Lynch syndroom? #00:33:48-4#

Gelach #00:33:48-4#

Gesprekleidster: Voor het kleinkind. #00:33:51-0#

V: Voor het kleinkind, dus die ziet geen rokende oma. #00:33:55-0#

Gesprekleidster: Hoe is dat voor jou *V*? Heb je in het verleden veranderingen in je leefstijl aangebracht? #00:34:01-3#

V: Um nee, niet echt. Niet echt aangepast. Wel van vlees eten, ben ik heel beperkt in. En groente en fruit, ben goed in beweging. Ja, ik rook daar moet ik vanaf. Soms denk ik ook wel van ja, daardoor heb ik een goede darmpassage. Ik denk wel eens, oh jee, als ik daarmee stop dan um ja. De rest heeft niet gerookt die hebben allemaal kanker en ik nog niet. #00:34:31-0#

Gelach #00:34:31-0#

V: Dat is natuurlijk jezelf misschien ook wel verschuilen. Nou ik ben daar um geestelijk aan het voorbereiden van ik moet stoppen. #00:34:39-0#

Gesprekleidster: Dat is een begin. #00:34:40-7#

V: Als je geest zegt van het gaat, dan gaat het. #00:34:45-1#

V: ja #00:34:45-1#

V: Daar hoef je helemaal geen vreemde dingen erbij te doen. #00:34:48-9#

V: Ik begrijp natuurlijk van mezelf natuurlijk ook heel goed dat het ook heel dubbel is. Aan de ene kant is er zoveel gebeurd in de familie dat je denkt hoe is het mogelijk dat je dit allemaal doet? En aan de andere kant denk ik dat in die jaren houvast is geweest om alles te overleven. Ik zit niet aan de drank en aan de pillen. En ik voel me voor de rest erg gelukkig. Dus dat is misschien dan weer de andere kant. Je zoekt een weg voor jezelf waarin je het beste bij voelt. Tot nu toe is dit het. #00:35:20-1#

Gesprekleidster: Hoe is dat voor jou M1? Heb jij je leefstijl ooit aangepast? #och3-7#

M: Ja, maar niet vanwege het Lynch syndroom. Ik heb ook vroeger wel gerookt en in '91 gestopt. Ja en ik drink wel eens wat. #h:36-0#

V: Ik denk allemaal wel.

M: En in de ene periode heb je dat je wat meer alcohol gebruikt, dan de andere keer. Soms helemaal niet en de andere keer dan um vind ik een flesje wel.. #h:46-8#

Gelach #00:35:46-8#

Gesprekleidster: Een flesje ook, ja #00:35:48-0#

Gelach #00:35:48-0#

Gesprekleidster: Geen glaasje, maar een flesje #00:35:50-2#

M: Ja, dat gaat bijna vanzelf dan. Maar er zijn ook keren dat ik helemaal niet drink. Niet echt problematisch. Maar ja echt wijzigen, ik ben vitamine tabletten gaan nemen. #00:35:51-1# #00:36:06-1#

Gesprekleidster: En heeft dat nog een bepaald aanleiding gehad? Dat je dat bent gaan doen? #00:36:09-3#

M: Dat is eigenlijk een wetenschappelijk um onderzoek, dat ik um in de Volkskrant stond over darmkanker. Dat bepaalde stoffen die in vitamine pillen dan zitten, dat als je dat voldoende binnen krijgt dat dat een positieve invloed zou hebben op de ontwikkeling van.

M: Je bedoelt multivitaminen # 0:36:27-6#

M: Ja #00:36:27-1#

V: Oké, dat wist ik niet. #00:36:30-5#

M: Die gebruik ik eigenlijk niet. #00:36:33-5#

M: Ja en dat doe ik nou ik denk wel 25 jaar elke dag gewoon. #00:36:36-3#

V: En welke pillen zijn dat? #00:36:36-3#

V: Gewoon die multivitamine #00:36:39-9#

M: Ja van de Kruidvat. #00:36:44-0#

V: Ja dat is prima zegt de diëtiste, je hoeft niet eens voor een duur merk te gaan. #00:36:47-0#

V: Ja, zo neem ik altijd vitamine C. #00:36:53-4#

M: Ik begin de dag met een uitgeperste sinaasappel. #8#

Gelach #00:36:54-8#

M: Dan heb je de C binnen. #00:37:00-7#

V: Ja daar kan ik dus niet tegen, hè. Uitgeperste sinaasappels, dan kan ik gelijk naar het toilet. #00:37:05-1#

Gesprekleidster: Hoe is dat voor jou V1? Heb jij ooit je leefstijl aangepast? Want je zegt dat je op moment bezig bent met een diëtiste, hè? #00:37:15-3#

V: Nu wel. Een vrouw met.. vroeger niet.. Nooit gerookt. En ja, alcohol met mate, met een etentje dat.. En soms een hele tijd niet en soms, ja. #00:37:30-7#

Gesprekleidster: En is er nog een bepaalde reden dat je nu.. Je had gezegd dat je op eigen initiatief na een

diëtist was gegaan. Is daar nog een bepaalde reden voor? #00:37:40-9#

V: Ja, vanwege die darm die ik niet meer heb. Om te kijken of ik daar toch wat beter kan worden. #00:37:45-8#

Gesprekleidster: Wat adviezen qua voeding kan krijgen, om te zorgen dat um ja de ontlasting beter, um ja, begrijpelijk. #00:37:55-8#

V: Questran slik ik er nu bij, dat is een cholesterol middel, maar um dat verdikt je galzuren en daar heb ik heel veel baat bij. En daar ben ik nu mee aan het sturen, begon met één keer op een dag zit nu op twee keer en ik denk dat ik na drie keer op een dag ga. #00:38:09-9#

V: Tegen je cholesterol? #00:38:21-3#

V: Nee, ja het is een middel voor je cholesterol. Maar ik slik het dus omdat ik geen dikke darm meer heb. Er zit een middel in wat je galzuren wat verdikt en in je dunne darm. #00:38:37-0#

V: Oh ja, dat het wat indikt meer. #00:38:37-7#

V: Ja #00:38:40-8#

Gesprekleidster: En hoe is dat voor jou? Heb je ooit iets.. #00:38:43-7#

M: Nee ik rook ook, maar dan zeg maar cannabis. Ik heb wel een tijdje twee jaar geleden een jaar niet gerookt. Verder echt niets meer. #00:38:53-3#

Gesprekleidster: Ja, ja #f1 38:53-3#

M: Nou ja, ik weet niet in hoeverre het ook invloed heeft op die hele operatie. Ik heb krampen vanwege die operatie. Dus toen ben ik toch maar weer um gaan roken, ja. #00:39:15-3#

V: Er is nog wel één ding waar ik nog wel heel erg op let de laatste twee jaar. Dat zijn um de E-nummers in voeding. Daar let ik wel op. En ik merk dus ook, want voorheen had ik dan, um ja, dat is toch wel makkelijk he zo'n pakje mix huppekee, eten is klaar. En dan had ik daar toch wel last van. En nou gebruik je dat eigenlijk niet, want doe je je eigen kruiden erin en dan heb je er toch minder last van. Dus um, ik denk dat het um die E-nummers um zeker die ene specifieke. Welke was dat ook al weer? 621 ofzo, dat dat niet goed is voor je darmen. #00:40:22-4#

Gesprekleidster: Oké, laten we dan.. #00:40:22-4#

V: De vraag zou dan kunnen zijn. Je zegt het is niet goed voor je darmen, maar ik weet niet in hoeverre dat in verbinding is met het Lynch syndroom. Het Lynch syndroom is gewoon een genetische afwijking en die gaat werken en die zet gewoon je genetische afwijkingen in je hele lichaam. En het openbaart zich het meest in de dikke darm of in maag-darm. #00:40:43-0#

V: Ja, via een poliep, omdat je een snellere celdeling hebt. #00:40:52-3#

Gesprekleidster: Nou is er eigenlijk nog helemaal niet zoveel bekend over waar het zit. #00:40:55-5#

V: Ja, maar ik heb wel zoiets van, je darmen zijn wel geïrriteerd. En in hoeverre heeft een geïrriteerde darm dan invloed met het ontwikkelen van een poliep of met een snellere celdeling. Dat weet ik niet, nee. #00:41:09-8#

M: Ja nou, ik heb nu de laatste tijd, ik eet altijd als ik eet dan moet ik ook enzymen erbij eten omdat mijn pancreas niet goed werkt. En ik had er laatst met iemand over, die zei ja, als je elke keer omdat, nou ja, dat is dan bij mij het twaalfvingerige darm, is eigenlijk een opvang bakje, want dan komt de gal erbij en

pancreassappen en dan pas gaat het je dikke darm in. Bij gaan natuurlijk die capsules je dunne darm, um je dikke darm in. En wat het dan voor je darmwand betekent, dat zien we dan op termijn ook wel um. #1#

M: Als je maar oud genoeg wordt, hè. Water drinken, water drinken, water drinken. #00:41:48-7#

V: Ja, dat doe ik ook veel. Heel veel zelfs. Zonder water kan een vis niet zwemmen zeg me moeder altijd. #00:42:00-8#

Gelach # 0:42:00-8#

Gesprekleidster: Nou, laten we, ja laten we, er is wel wat onderzoek gedaan natuurlijk hè? #00:42:09-5#

Meerderen: aha #00:42:09-7#

Gesprekleidster: Kijk, er komt iets aan #00:42:14-7#

Gesprekleidster: Naja ja, hè, er is behoorlijk wat um onderzoek gedaan wel naar de relatie tussen leefstijl en het ontstaan van kanker. Nou is dat, hebben wij het Lynch syndroom wel wat minder, zo wat minder uitgebreid onderzocht, dan dat voor andere typen kankersoorten. #00:42:49-1#

V: Ben nu van mening dat in de praktijk dat het anders is hoor. #00:42:47-7#

Gesprekleidster: Komt dit voor iemand voor trouwens? Of um #och4-4#

M: Nee #00:42:53-0#

Aanbevelingen WCRF:

Gesprekleidster: Daar hadden we dan geen rekening mee gehouden. Dit zijn aanbevelingen ter preventie van kanker. En die zijn um, die zijn gebaseerd op onderzoek wat er is gedaan. Kennen jullie het WCRF, het World Cancer Research Fund? #00:43:11-8#

M: Nee #00:43:11-8#

Gesprekleidster: Dat is een um stichting, een internationale stichting waar je ook in Nederland een afdeling zit. En die concentreren zich, die houden zich dus bezig met de relatie van leefstijl en het ontstaan van kanker, maar ook met de relatie tussen leefstijl als je al die diagnose hebt gehad en het terugkeren van kanker of de gevolgen daarvan die voor kanker en leefstijl. Daar houden zij zich mee bezig in het onderzoek. Wat zij doen is dat zij houden een update bij van de wetenschappelijke literatuur. Dus er zijn mensen die zijn constant bezig, die houden dat in de gaten. Dus die kijken naar inderdaad, wat is de invloed van rood vlees op darmkanker. En er is iemand die ordent die literatuur en die doet daar, hè, die trekt daar conclusies uit op basis van de literatuur. Die kijkt naar de kwaliteit van de studies en wat er onderzocht is en hoe het onderzocht is. En op basis daarvan komen er aanbevelingen. Nou, nou zijn er um een aantal algemene aanbevelingen ter preventie van kanker die het wereld kanker onderzoek fonds um gedaan heeft. En die hebben we hier even op een rijtje gezet. We hebben straks voor jullie um ook foldertjes, die liggen daar. Die “..” jullie, in, en we vinden ze zelf erg mooi. Wij hebben er niets mee te maken met die foldertjes. Maar wij hadden ze ingekocht, hè #00:44:39-4#

Gesprekleidster: Wat er bijvoorbeeld, wat er bekend is bijvoorbeeld uit de wetenschappelijke literatuur, is dat gewicht, dat BMI dat een relatie heeft met het ontstaan van kanker. En nogmaals, we willen benadrukken en dat is ook al gezegd. Het is, je krijgt wel kanker of je krijgt geen kanker. Je hebt de kans op kanker, die kan beïnvloed worden door verschillende factoren. En het ontstaan van kanker dat komt ook door heel veel factoren. En we weten eigenlijk ook niet zo goed precies door welke factoren allemaal. En hoe belangrijk alle factoren dan zijn. Dus nogmaals het is niet zo dat als je aan deze leefstijl aanbevelingen voldoet, dat je dan nooit kanker krijgt. Dat is natuurlijk niet zo. Het is ook niet zo dat als je aan al die leefstijl aanbevelingen niet voldoet dat je dan geen kanker krijgt. Nee, nou zeg ik het fout. #00:45:36-9#

Gelach #00:45:36-9#

Gesprekleidster 2: Ik denk dat het duidelijk is. #00:45:37-4#

Gesprekleidster: In ieder geval jullie begrijpen wat ik bedoel waarschijnlijk. Als je niet aan de aanbevelingen voldoet, dan wil het niet zeggen dat je kanker krijgt. Als je aan alle aanbevelingen voldoet, wil het niet zeggen dat je geen kanker krijgt. Zo simpel is het gewoon niet. Maar op basis op wat we zien in de wetenschappelijke literatuur, dat er toch wel bepaalde verbanden die er gelegd worden. En op basis daarvan zal je wel bepaalde aanbevelingen kunnen doen. Bijvoorbeeld zien we dat voor ontstaan van dikke darm kanker, dat het BMI daarvoor wel een risicofactor is. Dus hoe hoger je BMI is hoe hoger de kans op darmkanker, dat is ook zo voor borstkanker, ook zo ".." #00:46:15-2#

Gesprekleidster: Wat zegt u? #00:46:17-4#

V: Is bij mij te hoog, hoor. #00:46:22-9#

V: Maar ik hoorde wel dat bij mannen die Lynchsyndroom drager zijn, dat dat daadwerkelijk van toepassing is bij vrouwen niet. #00:46:31-7#

Gesprekleidster: Dat is mij niet bekend. Ik weet niet of dat bij jou bekend is? #00:46:34-9#

V: Er schijnt een heel onderzoek te zijn geweest daarover. #00:46:40-2#

Gesprekleidster: Het kan zijn, want dit ook is, hè, een opeenstapeling van het .. #00:46:45-8#

V: totaal #f1 46:45-8#

Gesprekleidster: .. totaal plaatje inderdaad. Nou verschijnen er regelmatig artikelen en die zeggen dan iets, er blijkt een verband uit, maar bijvoorbeeld uit een volgend artikel kan weer iets anders uit blijken. Je wilt eigenlijk een compleet plaatje uit de literatuur op één gebied te krijgen. En dat wil nog niet zeggen dat je dan al alles weet. Maar hè, dat geeft meer informatie dan dat je na één onderzoek hebt. En het kan zijn dat één zo'n onderzoek in het nieuws komt hè en dat krijgt dan bekendheid. Maar dat wil niet zeggen dat het dan vast staat dat het zo is. Hè natuurlijk, dit staat ook niet 100% vast. Maar op basis van wetenschappelijke literatuur die er is en daar is dan wel hè behoorlijk veel over kunnen we daar wel uitspraak over doen. #00:47:32-2#

Gesprekleidster 2: En dit is op algemene alle kankersoorten en dat soort dingen. #00:47:33-9#

Gesprekleidster: Ja, dit is alles bij elkaar. #00:47:33-9#

Gesprekleidster 2: En er is ook wel gekeken specifiek naar Lynch, mensen met Lynch syndroom in relatie met leefstijl, maar daar komt toch wel grotendeels overeen, soms zijn er verbanden zelfs sterker. Maar het meeste onderzoek is wat algemener. #00:47:49-2#

V: Nou ja, ik vind punt vijf vind ik nogal wat. Minder rood vlees maximaal een pond per dag. #00:47:52-9#

Gesprekleidster 2: Per week, daar staat een fout. #00:48:03-6#

Gelach

V: Ik denk, zo hé #00:48:15-6#

V: Nou, dat kan ik in een week ook niet op, hoor #00:48:19-7#

Gesprekleidster: Nou wat er aanbevolen wordt, is natuurlijk om een gezond gewicht.. Omdat een verhoogd BMI, dat verhoogd het risico op kanker. Dus vandaar dat er aanbevolen wordt om een gezond gewicht te

hebben. Er wordt aanbevolen om voldoende te bewegen. Dat komt ook overeen met de normale leefstijl aanbevelingen. En die 30 minuten per dag komen overeen met de aanbeveling, ook voor hart- en vaatziekten. Dus wat dat betreft is het eigenlijk allemaal hetzelfde en voor diabetes natuurlijk ook. Minder calorierijk voedsel en um suikervrije dranken. # 0:48:51-3#

Meerderen: suikerrijk #00:48:51-3#

Gesprekleidster: Of suikerrijk, sorry ja. Dat komt omdat dat ook het gewicht weer verhoogd, dat heeft daarmee mee te maken. De aanbeveling is om veel groente en fruit te eten. Ze willen een soort van alles door elkaar, omdat we eigenlijk niet precies weten welk stofje in welke groente en fruit precies verantwoordelijk is voor een positief effect. Wordt er gezegd, eet maar van alles door elkaar want dan krijg je zeker weten, dat je de stofjes binnen die goed zijn. Volkoren producten, peulvruchten um en dan vijf porties per dag en dat komt overeen met twee ons groente en fruit die wordt aanbevolen. In het buitenland is dat vaak vijf porties in plaats van twee ons. Um en dan dus minder rood vlees, dat was ook al ter sprake gekomen. De inname van rood vlees associeert dus met verhoogd risico op darmkanker onder andere. Um en dan dus inderdaad maximaal 500 gram per week en niet per dag. En geen bewerkt vlees, bewerkt vlees dat is vlees dat gezout is of gerookt is. #h:09-4#

Gesprekleidster 2: Dat geldt voor alle vleeswaren. #00:50:09-4#

Gesprekleidster: Ja, geen alcohol of in ieder geval zo min mogelijk. Daar wordt eigenlijk een beetje de leefstijl aanbeveling aangehouden ook die voor hart- en vaatziekte geldt. Als je naar de literatuur kijkt wat de invloed van alcohol inname is op het ontstaan van kanker. Dan is het wel heel duidelijk dat alcohol inname een duidelijk verband heeft. Dus op basis van die literatuur zou je eigenlijk zeggen, helemaal geen alcohol. Maar één glas bijvoorbeeld rode wijn per dag werkt juist weer gunstig op het risico op hart- en vaatziekten. Dus je kunt dus niet voor de ene ziekte zeggen van hè. De algemene aanbeveling is maximaal, met mate. Maximaal 1 glas voor vrouwen, maximaal 2 glazen voor mannen per dag. Minder zout eten of gezout voedsel eten. Dat is ook algemeen, hè. #00:51:07-4#

V: Dat is voor mij juist heel goed, meer zout. #00:51:07-4#

Gesprekleidster: En waar heeft dat mee te maken? #00:51:10-3#

V: Met het vocht gehalte, omdat ik dat vanuit de dikke darm niet meer. #00:51:19-7#

Gesprekleidster: Ja precies en zo zie je maar dat het individueel verschilt. Het zijn algemene resultaten. #00:51:24-2#

V: Dat is iets wat ik wel geprobeerd heb, maar het vervelende is, ik heb een normale bloeddruk hoor 60 en 110. En af en toe schiet het naar beneden toe en als ik dan te weinig zout neem dan gaat het naar de 50 en naar de 40 toe. En dan lig ik op de grond en dat is niet fijn. Dus ik neem eigenlijk iets meer zout dan een normaal mens juist. #00:51:50-8#

Gesprekleidster: Ja, dat klinkt wel herkenbaar. En natuurlijk niet roken. Het is ook wat tussen roken en kanker is natuurlijk veelvuldig onderzocht. #00:52:03-2#

V: Weet je wat ik mis in dit lijstje? #00:52:04-1#

Gesprekleidster: Nou? #00:52:04-1#

V: Zuivel, yoghurt #00:52:05-8#

Gesprekleidster: Ja, ja #00:52:05-8#

V: En omdat je eerder die vraag stelde, ik neem wel eigenlijk altijd yoghurt. #00:52:13-5#

Gesprekleidster: Nou, dat komt eigenlijk omdat er onvoldoende is aangetoond dat dat een effect heeft. Dus daarom komt dat niet hier in het algemene lijstje terug.   #00:52:23-9#

M: Maar je hebt natuurlijk ook van Boerma en dat soort dingen ".." geen links draaiend #00:52:29-1#

Gesprekleidster: Geen rechts.. #00:52:29-1#

V: Of de mensen die yacult nemen en dat is natuurlijk allemaal flauwe kul, maar geen yoghurt. Gewoon de normale yoghurt.   #4#

M: Maar neem je dan magere yoghurt of volle yoghurt? #00:52:45-6#   #6#

Gesprekleidster: Als u naar de aanbevelingen kijkt, of überhaupt over die aanbevelingen. Waren jullie daarmee bekend met deze aanbevelingen? #00:53:12-3#

Gesprekleidster: Ik zie jullie "ja" knikken #00:53:15-0#

M: Ja, maar dat heeft eigenlijk niet zoveel met kanker te maken, dat zijn gewoon de normale. #00:53:15-0#

Gesprekleidster: Behalve het rood vlees, hè? #00:53:21-0#

V: Ja, maar dat is toch nog heel veel hoor. Toch nog heel veel wat ze van je vragen. Ja dat zijn speciale mensen die daar gaan um kunnen komen hoor, voor een pond #00:53:32-8#

V: Ja als je nu naar je lijstje kijkt, dan komt het eigenlijk op neer waar we het eerder over hebben gehad van um alle toevoegingen die je nu in onze Westerse wereld allemaal eten, hè. Heerlijk aan de koek en gebak en al het extra wat we doen dat mis je hier allemaal in. Dus het komt er gewoon op neer dat we terug moeten naar de voeding die we in de jaren '50 hadden. #00:53:57-0#

Gesprekleidster: Maar u heeft het eigenlijk over puntje 3 volgens mij hè, minder calorierijk voedsel en suikerrijke dranken? #00:54:02-6#

V: Maar ook minder vlees, ze aten vroeger wel vlees, maar dat was één stuk in de week, want er was geen geld voor. #00:54:05-7#

Gesprekleidster: Ja, precies #00:54:07-2#

M: En vis was goedkoper. #00:54:08-3#

V: En vis was goedkoper #00:54:08-3#

M: Ja, je weet niet of er toen minder veel darmkanker was. Mijn opa was 44, en die um #00:54:16-6#

V: Tuurlijk vanzelf, maar overwegend is het wel zo dat de voedingsstijl van de Westerse wereld enorm toegenomen is in alle toegevoegde dingen die we eten. Ik was eens een week gaan kijken, van eens kijken wat ik eet. Maandag gebak, dinsdag gebak, woensdag een etentje ga maar door. Normaliter sta je er niet bij stil, maar als je er echt bij nadenkt wat je allemaal extra eet, is het gewoon ongelofelijk. #00:54:18-4#

M: Ja, je weet niet of dat minder is dat het dan beter zou zijn.   #00:54:59-7#

V: Ja maar daar hebben we hun voor, die gaan dat allemaal uitzoeken. #00:55:05-3#

Leefstijl verbeteren

Gesprekleidster: Als je zo naar de aanbevelingen kijkt, zijn er dan punten die je zelf zou kunnen verbeteren? Zijn er punten waar je niet aan..? #00:55:13-9#

M: Ik begrijp de 8 niet helemaal die daar staat. #00:55:13-9#

Gesprekleidster: de 8, die heb ik ook helemaal niet genoemd hè? #00:55:24-6#

Gesprekleidster: Nou, het is niet aangetoond, misschien staat het er net even verkeerd geformuleerd. Maar het is, laat ik het zo zeggen, het is niet wetenschappelijk aangetoond dat het een effect heeft op het voorkomen van kanker. #00:55:35-3#

M: Maar waarom staat het erbij? Dat begrijp ik niet. #00:55:38-8#

Gesprekleidster: ja, #00:55:45-4#

M: Die andere dingen zijn, dat moet je minder doen, dat moet je bewust doen en die andere is niet nodig. #00:55:46-6#

Gesprekleidster: Ja, het is niet nodig. Het staat erbij. Het staat erbij. Het staat in het rijtje erbij. Dat is iets wat onderzocht is hè, dat voedingssupplementen houden verband met... Dus dat is ook onderzocht. Of het nou daadwerkelijk wat doet ter preventie van kanker. En het antwoord is nee. #00:56:08-6#

V: Daarom was ik er ook mee gestopt. #00:56:10-7#

Gesprekleidster: Dus, en daarom staat het in het lijstje. #00:56:16-9#

V: Maar, individueel is het natuurlijk weer anders. #00:56:16-9#

M: Dus hij gaat er dus vanuit dat als je gevarieerd eet, dan is het niet nodig. #00:56:19-7#

Gesprekleidster: Precies, dat is het inderdaad. #00:56:24-9#

M: Dus de algemene aanbeveling ter preventie is gevarieerd eten. #00:56:27-4#

Gesprekleidster: Dat klopt. Daar heeft u helemaal gelijk in. De precieze aanbeveling is eigenlijk haal uw voedingsstoffen uit je dieet, uit je voeding en niet uit voedingssupplementen. Dat is de daadwerkelijke, ja. Dat is beter omdat, daar zijn verschillende redenen voor. #00:56:49-6#

M: Dus eigenlijk zou die bovenaan moeten staan wat mij betreft, gevarieerd eten. #00:56:38-4#

M: Ja, want dan volgt de rest eigenlijk ook gewicht enzo en meer bewegen #00:57:03-3#

Gesprekleidster 2: Volgens mij zijn er ook aanwijzingen dat voedingssupplementen bijvoorbeeld na het hebben gehad van kanker vrij is van de behandeling dat juist interactie kan hebben met de behandeling dat het dan eigenlijk wordt afgeraden. #00:57:13-8#

Gesprekleidster: Bepaalde voedingssupplementen kunnen met de chemotherapie, kunnen die een wisselwerking en dat werkt het de chemotherapie tegen. Wordt expliciet gezegd #00:57:29-6#

Gesprekleidster 2: Dus dan wordt het afgeraden zelfs, dus ik denk dat ook één van de redenen is dat die überhaupt op deze manier er tussen staat. #00:57:35-0#

Gesprekleidster: Maar u heeft helemaal gelijk dat het op die manier geformuleerd moet worden. Daar heeft u helemaal gelijk in. Als u deze punten ziet zijn er dan punten die, um, die u zelf zou willen veranderen op dit moment. Zijn er punten die u op dit moment, op bepaald gebied u leefstijl zou willen veranderen of niet. #00:58:11-0#

V: Die BMI mag bij mij wel lager. Ja, nou, ja daar ben ik al een tijdje mee bezig maar dat gaat erg langzaam. #00:58:14-0#

M: Ja, maar ik denk zo werkt, als je de horizon ver genoeg legt. Dat je in 6 weken wilt afvallen. #00:58:28-2#

V: Ja, dat is nu in een jaar gebeurd. Daarvoor was ik er dus ook altijd mee bezig. #00:58:38-0#

M: Maar nu heb je waarschijnlijk je eetpatroon aangepast waardoor je uiteindelijk wat afvalt. #00:58:40-7#

V: Ik heb eigenlijk hetzelfde eetpatroon als daarvoor, behalve dat ik dan geopereerd ben aan mijn schildklier. En dat die ontsteking weg is. Waarschijnlijk is mijn schildklier beter gaan werken. #00:58:59-9#

Gesprekleidster: En u vertelde ook over hormoon um #00:59:06-1#

V: Hormoonbalansdieet, maar daar was ik vorig jaar mee gestopt, omdat ik um, er wordt ook minder galvloeistof aangemaakt door dat dieet. Daardoor voelde ik me eigenlijk wel beter vanwege die ontsteking, die verzakt. Ja, dat zit ik me nu een beetje hardop te bedenken.".." maar, ik zou bijna het punt stress erbij willen zetten.#00:59:37-1# Als je een heel stressvol leven hebt, dat je fysiek al veel moet gaan doen. #00:59:50-0#

Gesprekleidster: Maar bedoelt u dan dat minder stress invloed heeft op het ontstaan van kanker of bedoelt u dat het verband houdt met uw leefstijl? #00:59:57-0#

V: Um, geniet van het leven zeg maar. #01:00:03-6#

Gesprekleidster: Ja, precies. Met andere woorden, zoals het er nou zo staat. We hebben een overzichtje inderdaad. Gesprekleidster 2 is bezig met al wat factoren. Die zijn eigenlijk een beetje ter loops aan bod gekomen al in het gesprek. Wij zouden graag een overzichtje willen hebben van factoren die nou de leefstijl beïnvloeden. Er zijn factoren die de leefstijl bevorderen, die er voor zorgen dat je gezonder gaat leven en er zijn factoren die um die je leefstijl niet.. #01:00:49-7#

V: En als je geniet van het leven kun je ook nog wel gezond zijn. #01:00:51-2#

Gesprekleidster: Nee, dat is ook zo. Factoren die genoemd zijn, inderdaad oma worden, hè, dat was het stoppen met roken. Leefstijl verbeteren hangt samen met oma worden #01:01:06-4#

V: Met leuke dingen. #01:01:10-5#

Gesprekleidster: Vanwege een darmoperatie, dat daarom het voedingspatroon aangepast is. Zijn er nog meer factoren die in rijtje thuis horen? Zijn er nog meer factoren die bijdragen aan dat je je leefstijl zou willen of kunnen verbeteren? #01:01:31-4#

Gesprekleidster 2: Ik hoorde net een hoog cholesterol ook nog. Dat was voor u een reden om uw leefstijl te veranderen. #01:01:40-9#

V: Ja, maar dat staat helemaal los van dit. #01:01:50-1#

Gesprekleidster 2: Dat maakt niet uit. #01:01:50-1#

Gesprekleidster: Zijn er nog meer dingen die in dit rijtje thuis horen? Redenen waarom je een bepaald aspect uit je leefstijl zou willen verbeteren?#01:01:56-2#

M: Of juist niet. #01:02:11-2#

Gesprekleidster: inderdaad #01:02:18-4#

V: Ik heb wel het idee dat ik het wel goed doe. #01:02:20-3#

Gesprekleidster: ja, dat is ook inderdaad. Dat is wat voor het rijtje van de minkant. Ik hoorde het eigenlijk al een beetje aan het begin. Als ik het goed samenvat willen jullie ook eigenlijk niet veel veranderen aan uw leefstijl op dit moment. #01:02:42-0#

V: Dat geeft ook heel veel stress. #01:02:42-2#

Gesprekleidster: De reden daarvoor is dat het eigenlijk al goed is. Dan hoef je niets aan te passen. #01:02:49-4#

V: Anders ben je constant met een dieet bezig, dus je bent constant eigenlijk ook met het Lynch syndroom bezig. Nu doe je het om een gezonde manier, geeft minder stress. #01:03:01-7#

Gesprekleidster: Dat heb ik ook al terug gehoord, de associatie met veel bezig zijn met het dieet of veranderen van je leefstijl en dat je dan ook automatisch met het Lynch syndroom bewuster bezig bent. Dat is ook een reden om het niet te doen.

V: Iedereen weet wel een beetje hoe je gezond kan leven. En het dieet van het Lynch syndroom wijkt niet zo veel af van een normale gezonde eetpatroon. #01:03:28-8#

Gesprekleidster: Is dat herkenbaar voor jullie? Dat dat een reden is, of is het meer gewoon: het is wel goed, ik wil niks aanpassen? #01:03:40-1#

M: Ik heb altijd het idee: wat is het nut van dingen aanpassen? Want wat je de één doet, moet je de ander laten en andersom. Als je het één laat, moet je ander misschien weer doen. Als je jezelf maar fit voelt in je lichaam en de manier waarop je leeft. Kun je wel zeggen ik wil mijn BMI lager hebben, maar op het moment dat je zegt van, ik vind mezelf prima, nou dan zou ik geen reden zien. #01:04:06-5#

V: Ja, maar dat vind ik niet, maar er zijn mensen die dat wel hebben. #01:04:11-1#

V: Ja, tuurlijk #01:04:14-5#

M: Ja, ik denk dat dat belangrijker is dan het getal. #01:04:18-2#

V: Komt dat misschien ook misschien van dat omdat we allemaal in hetzelfde levensjaar jaar groep zaten met de tijd met *Dr 1. *. En dat het algehele een beetje was van, genoeg, we weten nu dat we lynch hebben en dat het meer is van, goh plukte de dag en we leven nu en we zien wel hoe het verder gaat. Ik denk dat dat op het moment dat ik het zo opnoemde toentertijd met de gesprekken met mevrouw **. Nou weten niet meer, we weten wat voor risico we lopen, dus bewuster leven, daar samenhangend dit voor een deel in meenemen. Maar voor de rest genieten, hè? Ik sta nu voor mijn onderzoeken en dat vind ik vanaf nu weet dat ik de 14e onderzoeken heb. Jongens, mooi dat het de 15e is en dan gaat het knopje om en is het weer heel ver weg. Maar, dat wel in dat is dan ver weg. Maar dàt geldt denk ik voor iedereen, ook voor hart-en vaatziekten, voor alles is dit goed #01:05:25-4#

m: Maar ik maak me ook niet veel zorgen over mijn pensioen. #01:05:29-9#

v: bijvoorbeeld #01:05:28-5#

M: om maar is wat te noemen #01:05:32-1#

M: dat nabestaande pensioen belangrijker is dan mijn eigen pensioen #01:05:35-7#

Gesprekleidster: Maar ik hoor ook een beetje dat een reden is dat om het niet aan te passen is dat het geen nut heeft. Klopt dat? #01:05:50-7#

Gesprekleidster: Dat is een hele goede, dat hoort in de minderheid thuis #01:05:58-2#

v: toch denk ik wel dat je toch op het stuk van rood vlees eten wat bewezen is dat dat wel van invloed kan zijn op onze leefstijl. Je mag alles eten en dan leef je lekker en overal waar te voor staat.. #01:06:21-1#

M: wel mooi dat je iets gemeenschappelijks eruit haalt, hè? #01:06:24-4#

V: ik denk dat het toch minder voorkomt omdat je veel langer weet dat je Lynch hebt. Misschien is het interessant om te kijken bij mensen die het net te horen krijgen. #01:06:41-5#

v: Dat is wel interessant daar zou best een verschil kunnen zijn. #01:06:41-5#

v: alhoewel mijn dochter staat daar nog helemaal niet bij stil, dat is nog heel ver weg. #01:07:01-1#

v: ja, mijn dochters zijn wel met gezonde voeding bezig. #01:07:11-4#

m: ik denk dat het ook van invloed kan zijn dat je je gewoon beroert voelt. Ik kan nooit afbak croissants eten of vette dingen. #01:07:29-5#

v: het is ontzettend lekker, maar ik kan het niet eten. #01:07:33-6#

Gesprekleidster: in het rijtje van plus. #01:07:37-4#

v: ongezonde dingen niet kunnen verdragen, je darmen kunnen het niet aan #01:07:48-6#

v: bastogne koekjes kan ik niet eten. #01:07:48-6#

Gesprekleidster: Dat is nou een hele goede tip voor de volgende keer! Gesprekleidster 2, andere koeken moeten we neerzetten. #01:08:00-2#

Gesprekleidster 2: Daar hadden we niet over nagedacht #01:08:01-3#

Gesprekleidster: Dat is zeker net als jij, V, je operatie hebt gehad aan je darmen dan moet je toch letten op wat je eet, dan ben je daar wel bewust mee bezig, wat eet je wel en wat eet je niet #01:08:09-8# #01:08:22-0#

V1: ja, op een gegeven moment moet je wel. #01:08:22-8#

V: maar het grappige vind ik wel, het schiet me net te binnen, dat ik heb nog niets, zij ook nog niet, anderen wel. We hebben wel allemaal gevoelige darmen wat ik hieruit oppak. Dat wist ik eigenlijk niet, dat dat bij iedereen.. #01:08:57-9#

M: dat had ik voor mijn operatie ook al

V: als ik slagroom eet krijg ik pijn in mijn buik #01:09:00-8#

Als ik te veel gekruid eet dan kan ik er op wachten, dat is niet fijn #01:09:16-0#

Dat bedoel ik dus met gevoelige darmen #01:09:17-1#

Gesprekleidster: Herken je dat niet, V? #01:09:17-1#

V: Nee, ik kan niet zeggen dat ik gevoelige darmen heb #01:09:26-0#

V: Oh, toch niet #01:09:30-4#

V: Dan zou ik het anders zijn voor de maag, speculaas tijd #01:09:36-8#

V: Was ik laatst op een verjaardag, hij werd verbolgen voor het feit dat aardappels niet meer smaakten zoals vroeger. Alles werd bewerkt, maar dat is toch wel voor heel veel voeding zo. #01:09:48-8#

Gesprekleidster: bedoel je bestrijdingsmiddelen? #01:09:57-4#

V: maar ook genees... wat doet dat met de voeding? #01:10:02-8#

Gesprekleidster: Daar is niet veel over bekend, in ieder geval dat is niet wetenschappelijk bewezen dat het invloed heeft, zo zou je kunnen formuleren. #01:10:06-7#

Gesprekleidster 2: Hè, maar hebben het nu heel veel over voeding, maar er zijn ook andere leefstijlen zoals bewegen, alcohol gebruik.. #01:10:21-8#

V:Ik beweeg iedere dag #01:10:25-0#

M: Ik heb net een hondje genomen. #01:10:25-3#

Gesprekleidster 2: Dus het hebben van een hond is eigenlijk een positieve factor? #01:10:34-9#

M: Als reden om weer te lopen. #01:10:41-2#

Gesprekleidster: Was dat ook een reden? #01:10:46-2#

M: Ja #01:10:49-4#

Gesprekleidster: om meer te bewegen? #01:10:49-9#

M: Ja, je merkt dat je steeds minder gaat doen.   #01:10:54-5#

V: stok achter de deur #01:10:54-8#

M: mijn oude hond ging dood twee jaar geleden. Dan beweeg je toch iets minder, dan ga je wel fietsen, sporten en spinnen. Maar goed, dat is ook een soort therapie, hè? #01:11:13-3#

V: ik moet bewegen, ik heb net vier rugoperaties achter de rug. en dat zal ongeveer 60% blijven. dus ik moet blijven bewegen. spieren korset noemen ze dat, dus ik train wel #01:11:37-2#

Gesprekleidster: Dus dat is de reden dan om te bewegen? #01:11:42-5#

V: Naja, daarna natuurlijk dan. Je lichaam heeft zo’n klap gehad #01:11:51-7#

Gesprekleidster 2: Was dat voorheen anders? #01:11:53-0#

V: Nee hoor, ik heb voorheen ook veel sport gedaan. en bewegen. Ja, ik ben broodmager, haha, nee hoor #01:12:04-0#

Gesprekleidster: zijn er voor bewegen.. #01:12:14-4#

V: Daarvoor sportte ik ook nog wel, maar het was makkelijker om het op te pakken, om daar mee door te gaan. En nog steeds te door te gaan. Mijn laatste operatie was 2002 geweest, om gewoon om iedere week gestructureerd door te gaan. Want de meeste denken na een half jaartje ben ik klaar. Maar je ben niet na een half jaartje klaar. Het is een levenswijze en dat moet je altijd volhouden. #01:12:42-1#

Gesprekleidster: Wat is het dan precies dat het u lukt om vol te houden? #01:12:41-5#

v: Nou, waarschijnlijk omdat ik altijd gesport heb #01:12:45-7#

Gesprekleidster: gewoonte #01:12:45-7#

V: gewoonte ja, levensstijl is.. #ch52-6#

Gesprekleidster 2: omdat u zich er goed bij voelt #01:12:50-8#

V: Natuurlijk voel je je er goed bij, want als je niet doet voel ik me niet goed. Maar dat komt omdat ik het altijd gedaan.. #01:13:05-6#

Gesprekleidster 2: Dan is volhouden een gewoonte #01:13:07-9#

Gesprekleidster: precies #01:13:07-9#

M: Volhouden op karakter, hè? haha #01:13:13-8#

M: Ik loop drie keer in de week hard en ik vind er geen klap aan, saai. #01:13:17-6#

V: Dat vind ik dus saai #01:13:20-1#

M: Maar ik voel me wel fijn. Ik heb een uithoudingsvermogen en ik haal er zeker heel veel. En als ik dat niet doe, als je op vakantie bent geweest twee maanden, gewoon 6, 7 kilo zwaarder en dan voel je minder fit. #01:13:38-4#

Gesprekleidster 2: Dus uithoudingsvermogen en gewicht. #01:13:45-6#

M: beheersing #01:13:44-2#

Gesprekleidster 2: ja #01:13:44-2#

M: Ik denk ook dat je, nou ja, we zijn allemaal op die leeftijd, 50, rond de 50 zal ik maar zeggen, dat je wel merkt dat je lichaam veel sneller achter uit gaat dan vroeger. #01:13:55-5#

M: Dat je er wat aan moet doen. #01:13:59-3#

M: “spiereel” veel sneller slap wordt #01:13:59-3#

V: Mijn gezin heeft dan de luxe dat we een woning hebben in Spanje. Nu zit er een zwembad bij die vrij groot is, dus ‘s morgen gaan we baantjes zwemmen, dus ik kom in de vakantie nooit aan. Daar begin ik mee, dat is zo super decadent vind ik dat. #01:14:16-0#

Gelach #01:14:16-0#

V: he, je loopt je bed uit en doet je badpak en je gaat een stukje zwemmen en dan lekker ontbijten. Goed ontbijten, nou heerlijk. #01:14:33-5#

V: En de tomaten smaken lekkerder daar #01:14:35-4#

V: En de tomaten smaken lekkerder #01:14:43-2#

Gesprekleidster: En voor wat betreft roken, zijn daar um. We hebben het over bewegen gehad en voeding. En dan hebben we nog roken. 1 ding nog eigenlijk over.. #01:14:56-1#

V: alcohol dus ook #01:14:56-1#

Gesprekleidster: oh ja, sorry haha #01:15:02-4#

Wat um.. #01:15:06-4#

Gesprekleidster 2: Ik hoorde dat er een paar mensen gestopt zijn. #01:15:08-6#

v: Wat, met alcohol? #01:15:08-6#

Gesprekleidster 2: met roken #01:15:15-3#

Gesprekleidster: Ja, maar dat hebben al, dat weten, hè? #01:15:17-6#

Maar jij rookte nog voorheen, jij rookt ook en jullie roken allebei niet hè, of jij? #01:15:23-9#

V: Ik heb nog nooit gerookt #01:15:23-9#

M: Ik ben gestopt #01:15:26-3#

Gesprekleidster: Jij bent gestopt, precies. #01:15:26-1#

M: Al heel lang. #01:15:26-1#

Gesprekleidster: Heel lang, oké. En jullie hebben geen intentie om te.. #01:15:40-6#

M: Naja, twee jaar geleden heb ik een jaar niet gewerkt, ik vond het zo saai en ja ik miste het blij gevoel #01:15:53-2#

Gesprekleidster: Reden om niet te stoppen is dat je niet prettig voelt? #5:58-2#

M: ja #01:16:00-9#

Gesprekleidster: En is er voor jou een.. #01:16:09-4#

V: Ik ga stoppen met roken, daar ben ik wel mee bezig. #01:16:17-6#

Gesprekleidster : Met de mentale voorbereiding? #01:16:17-6#

V: precies #01:16:17-6#

M: maar wat zijn nou, wat zorgt er op dit dan voor dat je er niet mee bent gestopt? #01:16:27-3#

V: gewoonte #01:16:27-3#

Gesprekleidster 2: ja #01:16:27-3#

M: ja, nicotine is ook gemeen, hè? #01:16:30-5#

Gesprekleidster: Ja, het is een verslaving, hè? Laten we wel.. #01:16:45-1#

Gesprekleidster: Ja, maar het is een fysieke verslaving. #01:16:51-3#

V: Heb ook veel in mijn omgeving gezien, want alle niet rokers zijn allemaal dood. #01:16:56-8#

Gesprekleidster: Ik begrijp dan van, het maakt niet uit, of ik stop of niet. #01:17:08-1#

V: Ja dat is natuurlijk je kop in het zand steken. Het is natuurlijk algemeen bekend dat roken niet goed is. Misschien is het dan makkelijker voor mezelf. #01:17:27-9#

V: Nou, ik moet zeggen, ik betaal er niet meer aan, helemaal niet meer. #01:17:30-4#

V: En ik voel me prima, op beweging en ik fiets, ik wandel, ik loop en mijn gewicht is goed. Eigenlijk voldoe ik aan het rijtje alleen die onderste zin. hahaha #01:17:42-8#

Gesprekleidster: Ja, inderdaad. #01:17:46-3#

Gesprekleidster 2: Oh, dat is het alarm. #01:17:47-0#

Drie belangrijkste factoren:

Gesprekleidster: Oh ja, ik wou net zeggen, wat gaat er nou af? We hebben nou een mooi rijtje van factoren die de leefstijl beïnvloeden, hè, factoren die er voor zorgen dat je gezond blijft leven dat je meer gaat bewegen, minder gaat roken en factoren dat je juist niet gaat gezonder leven en ongezonde gedragingen blijft houden. En ook wat puntjes die met het volhouden van te maken hebben. Als jullie naar dit rijtje kijken, welke drie factoren zijn nou de belangrijkste voor jullie? #01:18:30-9#

V: Geniet van het leven. #01:18:35-7#

M: En goed bij voelen. #01:18:34-6#   #01:18:34-6#

V; Ja natuurlijk #01:18:35-9#

Gesprekleidster: Laten we even per persoon gaan kijken welke factoren zijn voor jou nou, als je drie factoren zou moeten noemen. Welke factoren zijn dan voor jou het belangrijkste? #01:18:51-1#

V: Nou ja, ouder worden, natuurlijk. #01:18:49-7#

Gelach #01:18:56-1#

Gesprekleidster : ja ja precies ja #01:18:55-7#

V: Geniet van het leven. Niet constant mee bezig willen zijn, dat geeft te veel stress. #01:19:01-5#

Goed bij voelen #01:19:06-8#

Gesprekleidster 2: Je mocht er drie noemen #01:19:06-1#

Gelach #01:19:08-5#

Gesprekleidster 2: Dus is die belangrijker dan '1 van de andere factoren? #01:19:14-6#

V: Even kijken, oh nou er staan er drie, klaar: oma worden, genieten van het leven en niet constant mee bezig zijn, want dat geeft te veel stress. #01:19:28-6#

V: Ja, bij mij staat duidelijk ook voorop genieten van het leven, en ik denk als ik dat doe, dan gaat het goed, ja. En niet constant mee bezig bent, is voor mij een beetje dubbel want er zijn ook periodes dat je er wel mee bezig bent. #01:19:57-8#

V: bij onderzoek #01:19:59-2#

V: Ja, maar ook als er weer wat gebeurde in de familie. Ik heb een hele grote familie, dan is het weer spannend, want de volgende generatie komt eraan en je schrikt er van hoeveel dragers erbij zitten. Mijn broer heeft vier kinderen en die zijn allemaal dragers. Dat is hartstikke veel, zijn allemaal jonge mensen en allemaal voor onderzoek. Ik bedoel en als dat rustig is dan zakt dat weg.   #01:20:32-4#

Gesprekleidster 2: Op het moment dat je er wel veel mee bezig bent, relateer je dat dan ook aan.. #01:20:37-1#

V: dat Lynch toch wel heel wat doet #01:20:44-6#

Gesprekleidster: ..stress? #01:20:48-6#

V: De confrontaties, dat mensen ook goed voor hun onderzoeken moeten gaan en niet laten zakken. En dat soort dingen gebeuren, gewoon. #01:21:04-6#

Gesprekleidster: Welke zijn dingen voor jou het belangrijkste? #01:21:03-8#

M: Niet constant er mee bezig willen zijn, voor mij belangrijk. En de afweging kosten en baten en je goed voelen. #01:21:19-6#

V: Hoe bedoel je, kosten en baten? #01:21:21-1#

M; Nou, zou je het een niet doen, wat levert het je op? Als je nou heel veel van biefstuk houdt en dan ga ik geen biefstuk eten, want dat staat in het rijtje. #01:21:31-2#

V: ja ja #01:21:33-5#

M: wat levert het je dan op? #01:21:41-0#

Gesprekleidster: je mag er nog een? #01:21:50-9#

M: ik heb er al drie #01:21:50-9#

Gesprekleidster: Ja, nou het gaat niet specifiek, maar drie mogen zijn, maar het is meer: wat is voor jullie het belangrijkste? Dat willen we naar boven halen. V, welke dingen zijn voor jou het belangrijkste? #01:22:03-3#

V1: Ja, door de darmoperatie genieten van het leven. Ik wil er ook niet constant mee bezig zijn, maar ik ben nu noodgedwongen. Ik ben hoop op langere termijn, dat ik het dan een beetje goed krijg. #01:22:22-0#

M: Bij mij is de ook de operatie van invloed. Maar, ik heb bij niet constant mee bezig zijn, alleen is het bij mij met stress gerelateerd, maar dat je niet wil dat je leven de boel bestijgt. #01:22:34-6#

M: Maar, dan bedoelen we allemaal hetzelfde #01:22:37-4#

Gesprekleidster 2: beetje hetzelfde #01:22:42-2#

Meerderen: Maar, dat bedoelen we allemaal denk ik. #01:22:42-1#

V: Het is ongeveer hetzelfde. #01:22:45-8#

M: Ja, die stress is niet zo direct voor mijn gevoel. #01:22:52-5#

Gesprekleidster 2: ik zet hem toch even apart #loch-5#

Gesprekleidster: Ik denk dat stress ook los is, het niet constant er mee bezig willen zijn. Is ook weer, staat ook weer apart van stress. #01:23:14-6#

V: Als je er steeds mee bezig bent, geeft het stress, dan wen je dus niet. #01:23:20-7#

Gesprekleidster 2: Is het dan bezig zijn met Lynch syndroom of met leefstijl, of is daar een link? #01:23:31-6#

Gesprekleidster: Ik hoorde net wel inderdaad, dat je levensstijl verbeteren gelinkt is met Lynch syndroom. #01:23:46-6# ? #01:23:46-6#

V: In mijn geval niet. #01:23:52-4#

M: Nee, is je algemene gezondheid. Je gaat niet kijken, heb ik nou 50 gram groenten. Dat je zo super bewust er mee bezig bent. Je eet gewoon waar je trek in hebt en dan of over het algemeen zo gevarieerd mogelijk. #01:24:18-2#

V: En dat staat dan in het plaatje van gezond leven en niet per se door het Lynch syndroom. #01:24:22-5#

Gesprekleidster: Oké, hoe ver.. Had je al drie dingen genoemd? #01:24:33-7#

Gesprekleidster: Dat je leven er niet door wilt laten bepalen, daar waren we een beetje gestrand volgens mij. Is er nog een ander iets? #01:24:41-2#

M: Ja, je goed voelen. Vind ik belangrijk. #01:24:50-2#

Gesprekleidster: Ja, je goed voelen inderdaad. #01:24:49-0# #01:24:57-3#

Gesprekleidster 2: Ik denk dat wel zo wel goed dingen genoemd.. #01:24:57-3#

Gesprekleidster: Ja, precies.

Samenvatting

Gesprekleidster: Ik denk, we hebben ook zeker gezien het alarm.. we gaan het afronden. Ik zal proberen het samen te vatten aan de hand van het lijstje. Onderbreek mij gerust als het niet overeen komt. Dan probeer ik een beetje samen te vatten wat er gezegd is. Ik hoor jullie zeggen dat jullie niet echt veel willen veranderen op dit moment en factoren dat je je leefstijl verbeterd zijn onder andere: oma worden, hè, dat je stopt met roken; de darmoperatie heeft effect op het voedingspatroon met name; een hoog cholesterol zorgt ervoor dat je je dieet gaat aan passen; slecht verdragen van ongezonde voeding, heeft ook weer te maken met de darmen, hè, dat je daardoor de ongezonde.. minder goed verdraagt en dat je daardoor ook minder.. dus dat is positief. Meer willen gaan bewegen en daarom een hond aangeschaft en daardoor ga je structureel meer bewegen. De rug operatie, die heeft, voor het herstel na de rugoperatie, spieren beter ontwikkeld zijn, reden om meer te bewegen en blijven bewegen zodat spieren goed bewegen ter preventie van rugklachten weer gaan beginnen. Omdat je je gewoon beter voelt als je meer beweegt, lekkerder in je vel zit, dat daardoor prettig is om meer te bewegen. Om je uithoudingsvermogen te vergroten, als je beweegt meer uithoudingsvermogen, dat is een reden om te blijven te bewegen. Maar, ook op gewicht te blijven, dat was, met name bewegen, hè, wordt er genoemd als reden. En de voornaamste reden die genoemd werd is gewoonte om het vol te houden. En de factoren die op een negatieve manier invloed hebben, die ervoor zorgen dat je niet gezonder nog, in hoeverre dat nodig is, hè, om nóg gezonder te gaan leven. Met name inderdaad omdat willen genieten van het leven en daarbij hoort dan ook dat daar misschien een keer ongezonde producten bijzitten, maar daar willen jullie eigenlijk niet constant mee bezig zijn. Met of iets gezond of ongezond is.. En stress is genoemd als een factor die bijdraagt aan ongezond leven. Zouden jullie daarop wat meer in kunnen gaan, hoe stress samenhangt met ongezonde leefstijl? Dingen of.. niet verbeteren in ieder geval.. #01:28:34-3#

V: Stress kan er voor zorgen dat je gewoon minder honger hebt en door stress ben je kan je dan foute voeding tot je nemen. Ik heb dan toch van, laat ik dat dan maar nemen. #01:28:46-7#

Gesprekleidster: Is het dan ook net voor onderzoek dat je meer geneigd bent om weer, of is dat.. #01:28:57-9#

V: daarna #01:28:59-0#

V: ik heb wel eens dropjes gezogen terwijl ik aan het keuren was #01:29:04-3#

V: nee hoor, na het onderzoek broodje kroket of #01:29:08-6#

Gelach #01:29:08-6#

V: In het ziekenhuis? #01:29:08-6#

Bij de koffie corner #01:29:17-7#

Gelach #01:29:21-1#

v: Je hebt zo'n honger en je bent er natuurlijk ook twee dagen mee bezig. #01:29:25-4#

M: En het vervelende is dat je trek hebt in die dingen die je normaal ook niet eet. Maar, omdat je niks mag, heb je er trek in. #01:29:31-6#

V: Nee, hierzo, bij de *ZKH2*, lekker gezond, haha. Maar, of dat dan afreageren is, van maar dat smaakt hartstikke lekker, hoor. Dan ben je weer voor een jaar klaar mee. haha #01:29:46-8#

V:Wat grappig

Gesprekleidster: Ja, wat ik voornamelijk al heb gehoord: een reden om je leefstijl niet te veranderen is omdat jullie al een gezonde leefstijl hebben. Dan hoef je je gezondheid niet te veranderen, de kosten zijn hoger dan de baten. Dus je moet eigenlijk meer inleveren om een gezonde leefstijl te aan te nemen, dan dat je er voor terug krijgt. Dus waarom zou je het aanpassen. Het is saaier, om een gezonde leefstijl te hebben, dat heeft met name met de cannabis te maken, hè? Minder prettig om een gezonde leefstijl te hebben, ook met name met cannabis te maken. En um, ook je leven er niet door te laten bepalen, dat is ook wat ik meer terug heb gehoord, hè. #01:30:35-6#

V: Ik denk dat het ook hetzelfde is als niet constant mee bezig zijn. #01:30:38-7#

Gesprekleidster: Ja, dat is een beetje hetzelfde, hè? #01:30:40-4#

V: Niet door laten bepalen is voor mij hetzelfde. #h:45-2#

Gesprekleidster: Precies, dat is eigenlijk wel hetgeen wat het sterkste uitkomt, hè? Als je zo naar het overzichtje kijkt, is dat wel de belangrijkste reden om het niet te willen veranderen. En om het juist wel te willen veranderen, los van oma worden, de darmoperatie en je er goed bij voelen. Dat zijn de 2 dingen die het meeste uitkomen.. En bij het blijven, um, blijven hebben van ongezonde gedragingen, hangt dan mee samen dat het.. #01:31:27-8#

M: dat het anders saai is #01:31:27-8#

Discussie

Gesprekleidster: Ja haha, Dat het een gewoonte is, het roken met name. Missen jullie dingen in deze samenvatting. Zijn er dingen die ik niet noem die wel van belang zijn om het te noemen. Zijn er überhaupt nog dingen die we niet genoemd hebben? #01:31:52-2#

M; Nou, ik vind wat wel genoemd hebben wat redelijk gezamenlijk was dat het idee van pluk de dag. #01:31:58-6#

Gesprekleidster: Ja, dat is een hele goede #01:32:01-8#

Gesprekleidster 2: Beetje van, geniet van het leven, of toch nog iets anders? #2:01-8#

M: Dat vind ik toch iets anders dan pluk de dag, doe het vandaag of #01:32:09-8#

V: het besef #01:32:12-3#

V: we gaan het niet doen als we met pensioen.. #01:32:16-3#

Gesprekleidster: Dat gaat een beetje samen met ik ben niet met mijn pensioen bezig. Meer in het nu leven.. #01:32:24-9#

V: Dat is bij ons ook de reden geweest dat we toentertijd, al een huis in Spanje gekocht hebben. #01:32:28-8#

Gesprekleidster: Ja, stel niet uit. #01:32:29-0#

V: Stel niets uit, als je het kan doen, dan kan je het doen en dan moet je het doen #01:32:34-5#

Gesprekleidster 2: Ja, precies #01:32:39-0#

Gesprekleidster: Dat is een hele goede aanvulling, heel goed. We gaan nu naar het einde toe. zijn er nog dingen die jullie hebben gemist in de discussie, zijn er nog bepaalde dingen die we niet besproken hebben die jullie zouden willen bespreken? En laten we dan meteen het rondje even maken. Dat iedereen individueel nog dingen kan zeggen of vragen. En dan betrek ik ons er eventueel bij, dat wij nog een vraag kunnen stellen. V, zijn er wat jou betreft nog dingen die niet besproken zijn, dat je nog vragen hebt? #01:33:09-9#

V: Kan er zo niet opkomen. Misschien thuis. #01:33:14-3#

Gesprekleidster: Dat is meestal, hè? #01:33:14-3# *V* zijn er wat jou betreft nog dingen die ik nog niet besproken heb? #01:33:18-2#

V: Ja, ik zou toch nog wel willen benadrukken, want het valt me op dat het toch een overwegend gezonde leefstijl is hier bij ons. Ik vraag me dus af, jij zei we zitten in dezelfde leeftijdscategorie. Hoe hebben de lynch mensen die net horen.. #01:33:40-5#

Gesprekleidster: Dat is wel interessant inderdaad. Ik hoorde het eerder ook al. #01:33:42-2#

V: Is dat heel anders? Of is dat ook zo? Of hebben die.. #01:33:51-9#

Gesprekleidster 2: Over het algemeen kunnen we stellen dat de populatie met Lynch syndroom dragers heel vergelijkbaar is met algemeen. Voldoen aan de leefstijl.. #01:34:07-0#

Gesprekleidster: Maar dat is inderdaad wel interessant om te vergelijken, hè? Willen mensen die net te horen krijgen dat ze het Lynch syndroom hebben, zijn die geneigd om te veranderen, ja of nee? Dat is iets wat we kunnen registreren in het onderzoek wat we gaan doen. Wat dat soort dingen gaan we neerzetten. #01:34:20-1#

V: en of die überhaupt nu een gezonde leefstijl hebben, ik denk bijna interessanter is op het Lynch syndroom dan van ons hier. Ik denk dat wij toch allemaal.. #01:34:36-8#

Gesprekleidster: Dat gaan we meten.

V: generatie bepalend dan, bepaalde leeftijdsgroepen #01:34:41-7#

Gesprekleidster: Ja, dat is een heel interessant punt. #01:34:42-0#

V: en misschien ook omdat wij al veel langer weten dat wij Lynch syndroom dragers zijn. #01:34:51-7#

V: Dat is het punt dat wil ik nog wel benadrukken. En ik mis de yoghurt   #loch-7#

Gelach #01:34:56-7#

M: op tafel of op het bord? #01:35:00-3#

V: op het bord #01:35:03-4#

Gesprekleidster: Ja inderdaad, M1 zijn er wat jou betreft nog dingen, die..? #01:35:07-5#

M1: Ik heb geen dingen. #01:35:06-1#

V: nee, ook niet #01:35:10-0#

M: nee #01:35:12-9#

Gesprekleidster: Ik heb eigenlijk ook geen aanvullende vragen meer, ik denk dat we de hoofdvragen hadden staan. En ik denk eigenlijk dat we gewoon de belangrijkste dingen benoemd hebben. #01:35:28-0#

V: Het enige wat ik wel wil weten, zijn er ziekenhuizen die wel informatie meegeven? #01:35:33-3#

Gesprekleidster: Dat weten we niet #01:35:33-3#

Gesprekleidster 2: In de richtlijn is het niet opgenomen, dus in principe gaan we ervan uit dat het niet gedaan wordt. #01:35:44-3#

V: Had ik nog wel een andere vraag, het gaat er eigenlijk om dat omdat vanuit het * ZKH5* in het voorjaar een announcement is gemaakt over het medicijn dat eventueel een vaccinatie. Daar ze zijn ze daar mee bezig. Wordt dat in het onderzoek ook meegenomen? Of is dat staat het helemaal los van elkaar? #01:36:08-5#

Gesprekleidster: Dat staat los van elkaar. #01:36:18-9#

V: Ja, omdat de afdeling genetica, zijn natuurlijk ook weer bij ons terecht gekomen.   #loch-4#

Gesprekleidster: Ja precies, er staat wel meer informatie over op de site van het * ZKH5* #01:36:28-7#

V: Ja, eigen patiënten haha, eerst eigen patiënten ze zijn eerst met hun eigen patiënten bezig #01:36:40-7#

Gesprekleidster 2: Ja, het is nog een heel vroeg stadium dat onderzoek, dus dat wordt nog niet heel grootschalig ingezet. #01:36:39-7#

V:"..” had ik begrepen #01:36:50-8#

Gesprekleidster 2: Ja, zo precies weet ik dat eigenlijk niet.   #01:36:46-0#

V: Wel interessant #01:36:51-4#

V: super interessant #01:36:57-3#

V: ja echt #01:36:58-9#

V: ook voor jou #01:36:58-9#

V: Ja, mijn vader is overleden, ben ik er nog en misschien straks voor mijn kinderen "..", dus ik vind echt.. ben heel benieuwd. #01:37:16-3#

V: Ja, maar toch ga ik misschien wel heel ver dan krijgen ze geen coloscopieën. Je hebt gewoon een genetische afwijking.   #01:37:19-5#

V: Het is dan een tegenmiddel, dat betekent dat het dan opgelost is hoop ik. #01:37:22-4#

V: Nou, ik weet ook dat ze ook vanuit de *ZKH2* bezig zijn met een pil. Zijn ze al vrij ver mee met die pil, en dan krijg je die pil en dat die registreert dan alles en die mag je uitpoepen en dat schijnt ergens dan vanuit een computer opgepakt te worden. Met et of wat dan ook en dan krijg je geen coloscopie meer. #01:37:46-0#

M: dat is een flitskeur #01:37:45-8#

V: weet ik veel, nee, daar zijn ze mee bezig #loch-5#

V: voor de dunne darm met name #01:37:50-7#

M: omdat je dunne darm zolang is kunnen ze dat niet met een scopie en dan krijg je zo'n dat flits de hele tijd. Maakt elke keer een foto.. #01:38:00-1#

V: dus um, hij is nog niet helemaal uitontwikkeld, had ik begrepen #01:38:05-6#

M: ik heb 'm al gebruikt, je moet het opvangen en dan weer terugbrengen en dat lezen ze wel #01:38:08-8#

Gesprekleidster: Toch bijzonder, he, dat het..   #01:38:12-1#

Gesprekleidster 2: Ja, de ontwikkelingen gaan ontzettend snel op dat gebied.   #01:38:15-6#

V: Ook een internist, nou ben ik weer zijn naam kwijt ** die zij tegen mij: we zijn nog niet helemaal ver genoeg mee. En straks krijgen al mijn patiënten die een coloscopie hebben krijgen straks die pil. Als je het aanvraagt, dan heb je ‘m gelijk. #01:38:33-9#

M: het was wel heel vervelend hoor, het duurt langer dan die scopie. #01:38:37-7#

V: oh, meen je dat? #01:38:39-7#

M: ja, want het duurt veel langer dat je nuchter moet zijn. Je moet ‘s ochtends beginnen met.. hoeft het daarna niet te spoelen maar je moet uiteindelijk een etmaal. Terwijl bij die scopien begin je ’s avonds, ten minste dat heb ik altijd. ‘s ochtends het onderzoek dan ben je om 11 uur klaar. #01:38:58-4#

M: ja, dan kun je weer aan de kroket. #01:38:58-4#

Gelach #01:39:01-6#

M: ja, je moet wachten totdat die eruit is, weet je wel. Bij mij was het er heel snel eruit, omdat de helft weg is. Maar daarna mag je ook niet eten, hij moet eerst eruit zijn, dus het duurt veel langer. #01:39:12-0#

V: Oh, dat is, ik niet, dat had je er niet bij hoeven te zeggen hoor. #01:39:15-2#

Gesprekleidster: Nee, dat is een klein detail, hè? #01:39:26-7#

V: Ja, dat heb ik al die jaren gehad.   #01:39:26-7#

V: En gaat dat goed? #01:39:28-1#

V: Ja, ik was daar zeer steeds te spreken over. Dat ".."krijg ik niet weg want ik heb wel van m’n 18.. #01:39:38-2#

V: Nee, ik bedoel het onderzoek.   #01:39:34-0#

V: propofol dat is een dieperre ".." dan darmkanker #01:39:44-6#

V: Nee, ik heb nog altijd ".." #01:39:43-5#

M: Ik kan juist niet tegen het nieuwe spul #01:39:48-4#

V: ja, ik had dat hoe het ook alweer propofol ook   #01:39:52-8#

V:".movieprep."   #01:39:52-8#

V: nee, nee, twee keer 200 mL en voor de rest hoefde ik alleen maar water te drinken. En dat heb ik vanaf mijn 18 gebruikt. Ik heb wel cleanprep, vreselijk. #01:40:04-4#

V: movieprep is beter #01:40:08-2#

M: die andere is veel scherper   #loch-4#

V: oh, fosforal had ik #01:40:10-4#

V: oh, fosforal   #01:40:15-4#

V: dat ken ik niet #01:40:18-7#

V: dat onderzoek onder propofol #01:40:19-4#

M: ja, in het *ZKH1* mag ik dat kiezen. #01:40:19-4#

V: jij mag dat, heb jij het al wel eens gedaan? Is dat goed gegaan?#01:40:26-3#

M: ja, heel erg pijnlijk, iedere keer wakker bij het onderzoek, dat is niet fijn. #0:30-9#

V: en nu ben je niet "wakker"? #01:40:30-9#

M: Nee #01:40:30-9#

V: ah, helemaal geweldig #01:40:30-9#

M: Na ja, je bent er wel een beetje bij, maar veel vager #01:40:39-4#

V: Je moet wel meewerken #01:41:28-4#

V: Dat ziet er allemaal lekker uit. #01:41:28-4#

Gesprekleidster: Dat ziet er kleurrijk uit. Ik vond het er mooi uit zien # 1:41:32-7#

V: Ja, ik zou er zo mee beginnen. #01:41:36-6#

V: Ik ben altijd verbaasd wat jullie allemaal uit een onderzoek weten te halen. Ik heb ooit een keer een vragenlijst ingevuld en toen was ik er ook een vraag over kaas. Een x aantal weken later werd er gevraagd

wat voor kaas dat was. Hoe is dat toch mogelijk? #01:41:56-8#

V: van Wageningen, want die had mij ook gebeld, rijstcrackers eet je helemaal geen brood dan #01:42:02-7#

Gesprekleidster: Echt waar? #01:42:02-6#

Gelach #01:42:02-6#

V: Dat was vorig jaar #01:42:11-2#

V: dat geeft aan dat er gekeken wordt #01:42:13-8#

M: hebben jullie ook bloed gegeven? #01:42:13-8#

Gesprekleidster: Ja, want het maakt wel veel uit of je 48+ of 40+ of 30+ of 20+ of geitenkaas dat maakt veel uit. #01:42:25-7#

Daar wordt onderzoek naar gedaan inderdaad #01:42:32-1#

Ja, als je zo'n vragenlijst krijgt dan denk je: wat moeten ze hiermee? #01:42:32-6#

Gesprekleidster: ja, ze zijn heel groot heel lang #01:42:36-2#

V: ja, daar moet echt voor gaan zitten #01:42:43-4#

V: nou, dat invullen dat gaat wel, maar om dat uit te zoeken. Ik dacht, ja, we vullen het wel in, maar.. #01:42:50-9#

Gesprekleidster 2: Ja, er wordt echt wat mee gedaan. #01:42:56-5#

Gesprekleidster: We hebben nog formulieren voor de reiskostenvergoeding die we jullie mee willen geven. Dus die kunnen jullie invullen met retour enveloppen erbij . Daar kunnen jullie gebruik van maken. #01:43:12-9#

En verder willen wij jullie heel erg bedanken voor jullie deelname. Heel erg fijn dat jullie wilden komen en we hebben heel veel informatie denk ik uit dit gesprek gehaald. Nogmaals, jullie worden op de hoogte gesteld van het onderzoek. #01:43:33-5#

Gesprekleidster 2: Als u reiskosten vergoeding wilt, wilt u mij het formulier terug geven als u het al had ingevuld? En daarbij vermelden of u met openbaar vervoer of eigen vervoer bent.

Focusgroep 2 Nijmegen 140930 00:00:00-0

Context/Persoonlijke informatie

Gesprekleidster 1: ..wie je bent en hoe lang je al weet dat je Lynchsyndroom hebt? En eventueel wat u hierover kwijt wilt. Of u geopereerd bent, of u kanker heeft gehad, hè.. Is er iemand die wil beginnen met voorstellen? # 0:00:22-3#

M: Oh, dat wil ik wel. Ik ben M1. Ik ben ongehuwd en ik ben 63 jaar kom uit **, **. Dat is een klein plaatsje midden in de **. En ik weet sinds 1982 dat ik het Lynchsyndroom heb. Dit is aan het licht gekomen door een familie onderzoek. Mijn vader is daaraan overleden dus, en naderhand is een oom die ook een x aantal keer geholpen is aan het Lynch syndroom. Dat rechtvaardigde toen een familie onderzoek, opgezet door het ** en die hebben het hele familie onderzoek van vaderskant helemaal doorgelicht. En daar ben ik dus 1982 uitgerold. Om 9.00 kwam ik hier binnen en in het *ZKH5* en nog minder gezond te zijn. Ik stapte om 14.00 naar buiten en toen wist ik dus dat ik een grote poliep zo een beetje op de scheiding tussen de dikke en de dunne darm. En eentje 30 cm achter ongeveer, en ik lag met een maand ongeveer hier in het ziekenhuis, werd geholpen. En hebben ze dus het hele dikke darm verwijderd. Ik heb geen stoma, achter is een stukje endeldarm "..". En sindsdien ben ik hier nog steeds onder controle bij Dokter "**" jaarlijks. En ja nou voorlopig is dat het wel zo een beetje dacht ik.

Gesprekleidster 1: Zou jij iets over jezelf willen vertellen? 00:02:22-8

V: Ja, ik ben V1 ... ben 49. Moeder van twee kinderen, 14 en ik kreeg aan de kant van vader, zijn, zijn vader, drie zussen, vier broers, allemaal overleden aan.. de dames aan de eierstok en de mannen aan de darm, mijn vader ook toen hij veertig was. Ik was drie, mijn vader 42 en ja, wij wisten niet beter als dat darmkanker niet erfelijk was. Dat was toentertijd, dat klinkt al heel ernstig, wisten niet.. In 1997, de rest mijn vaders hele gezin, 1 tante in leven, 83 of 86 is ze. Als jullie iets willen daarmee dan moeten jullie het nu doen, want ik leef nog en nou ja, de huisarts had altijd gezegd van, nou ja, het is niet erfelijk. Dus we hadden zoiets van, nou ja, we doen wel mee, heel, ja, nonchalant. Daarvoor zijn wij naar *ZKH3* geweest, kan dat? Of *ZKH4*, *ZKH3*, weet ik veel. Nou, daar werd mij bloed afgenomen en dan kon je na twee weken of een week later kwam de huisarts op visite. Dat, um, welke nichten en neven en mijn oudste zussen en ik gendrager waren van het HNPCC, want het Lynch dat weet ik pas sinds twee weken. Daar had ik echt nog nooit van gehoord. 00:04:11-0

V: Ja, dat begrijp ik intussen. Nou ja, dat was redelijk paniekerig, want ik had nog geen kinderen en ik wist ook niet of ik dat wel of niet wilde. Toentertijd was het, eierstokken moeten er preventief uit. Ik ben naar het *ZKH4* ziekenhuis in ** gerend en die hadden zoiets van je moet zo snel mogelijk de boel eruit. En mijn oudste zus had dat ook gedaan, maar die wilde ook geen kinderen. Die was ook al wat ouder, die had zoiets van, eruit, eruit. En hoe dat daartussen is gegaan dat weet ik allemaal niet precies. Ik ben, uiteindelijk heb ik er voor gekozen om naar het *ZKH5* te gaan en ben ik ook bij dokter ** gekomen meer voor de darmen. Die heb ik 1 keer gezien sinds 1999 heb ik het gehoord, ken hem niet. Die heeft het een beetje uitgelegd. En gezegd waar ik naartoe moet en sindsdien heb om de twee jaar een onderzoek naar darmcontrole. En bij dokter ** voor mijn eierstokken en baarmoeder die er nog steeds in zitten. Ik ben niet aan mijn darm geopereerd, vorig jaar de diagnose beginstadium borstkanker gehad. Moet wel zeggen dat dat het laatste was wat ik verwacht had. Want dat hoorde er helemaal niet bij. En ik had al iets je bent eigenlijk altijd bezig met dìt stuk. En ja, de rest, ja, naïef natuurlijk, maar ja, dat heeft het wel dichtbij gebracht. Dus ik ben recentelijk weer op controle geweest voor mijn eierstokken en mijn baarmoeder dan. Ik maak me eigenlijk meer zorgen over mijn darm, dat is veel makkelijker te vinden. En als je veel onder controle bent, maak ik me daar minder zorgen over. Tenminste zo is het eruit gehaald.

Gesprekleidster 1: Ja, 00:06:18-2

V: Ja en er zag er goed uit. En verder hebben wij informatie. Ik weet wat ik weet, maar er komt niets, ik hoor bijvoorbeeld ook op het schoolplein dat er een moeder op die hier werkt een vaccin heeft uitgevonden. En die een groep heeft en ik zei: oh, maar ik heb dat ook en dat ze dan zegt: oh, wat erg, dan had je bij mij in die

groep gekund. We wonen praktisch bij elkaar om de hoek. Dus ja, dat heeft me heel erg gerust, maar ook teleurgesteld, want we kennen elkaar we horen gewoon bij haar in de groep. Dat is onzin, maar snap je? 00:07:02-2

Gesprekleidster 1: U had verwacht dat u daar eerder over geïnformeerd zou worden? 00:07:02-4

V: Dat is onzin. Maar zij werkt daaraan, ik hoor via mensen op het schoolplein dat er iets op is gevonden. Zo van, jij had toch iets aan je darmen, dan moet je met haar praten. Nou ik bel haar op en zij vertelt: ja, ik heb dat en dat uitgevonden. En ik zeg: oh, maar dat heb ik. Ze zegt heb jij dat? Sowieso zo'n vaccin vind ik wel heel revolutionair. Weet ook niet de ins en outs daarvan dus in die zin zou ik meer verwachten. 00:07:24-7 00:07:36-2

Gesprekleidster 1: Ja precies, zou jij? 00:07:39-0

V: Ja hoor. Ik kom uit ".." ben getrouwd, ik 62. Ik heb sinds tien jaar geleden te horen gekregen, maar we weten wel dat ".." erin zit. We hebben in '82 met de familie aan mijn moeders kant laten onderzoeken, omdat iedereen aan kanker overleed. En een zus daarvan waren al wat jongere vrouwen, dus de dochters alweer. En toen zijn wij dus ook hier geweest, de informatie gehad dat dus iedereen eigenlijk onderzoek kon doen, wat ze heel graag wilde, maar in die tijd, jonge mensen en daar waar veel maar'tjes aan. Als we bijvoorbeeld in een huis wilde en het zou bekend zijn, dan was het "..". Dus op zich hadden we gezegd, hallo, laten we het even. Dat was in mijn familie helemaal zuust, hè, er werd veel over gesproken. We deden heel trouw allemaal “postonderzoek”. Moet ik wel zeggen, omdat veel heftiger aanwezig was en uit onderzoek kwam er uit dat dus en borstkankergen en de darmgen aanwezig was. En 1 oom had maar het darm gen. Dus we maakten ons daar niet heel druk over. Dus wij hebben gezegd, als we ouder worden zitten we allemaal op onze plek en de kinderen zijn er, dan kunnen we nog eens kijken van, hoe zit dat nou precies. Dus, dat is eigenlijk een stukje wat toen heel duidelijk naar voren kwam "..." En ik maakte een overgang, ben aan het vloeien gegaan en toen werd, baarmoederkanker gehad, en toen kwam ik in *** terecht, dat moet ik erbij zeggen. Toen ben ik één jaar onder controle geweest, maar op een bepaald moment krijg je veel meer informatie. Ik had als, is aangekaart, is het gen van de baarmoeder eigenlijk niet hetzelfde als de darmen? Nou, ik kon met die man eigenlijk niet goed door 1 deur, want hij zei als je je laat onderzoeken dan vind je altijd iets. Ik denk met mijn ".." geschiedenis en de geschiedenis van de familie dat het een beetje.. Maar goed, dat ging niet helemaal, ben ik weer terug naar de huisarts gegaan. Ik zeg, goh, ik wil me laten onderzoeken ik heb nou de leeftijd, mijn leven zit weer op de rit en ik wil gewoon weten of ik wel of geen draagster ben. Nou, dat was natuurlijk meteen bingo en ik kwam daarna ook pas geopereerd, mijn dikke darm is eruit gehaald. 00:10:06-6

V: Dus toen had je al kanker, maar je wist niet dat je het gen had?

V: Nee, dat was toen in het gesprek, ben eerst bij de huisarts geweest, toen zei ik van: ik wil dat gen onderzocht hebben. ".." Voor mij was het eigenlijk al klaar. Ik was ook niet van, oh, ik schrik, want ik heb dat gen. Ik schrok niet, want ik dacht 1 en 1 is 2. Dus toen zei ik van nou ik kom nu in het vervolg terecht. Dus, ben bij die mevrouw geweest, die had uitgelegd dat ik drager was. Toen ben ik bij ".." terecht komen. 00:10:35-8

V: Dat is hier onderzocht, ja. Ik heb dan nog 2 broers met hetzelfde gen. En ik ben een gezin van 8, twee hebben het laten onderzoeken en die jongeren die nog niet ".." 00:10:55-4

M: Zijn wel onder controle?

V: Het zijn dames, ze zijn wel voor borstkanker controle maar niet voor de darmen. 00:11:05-0 Ja, vertel mij wat..

V: maar borst is een apart gen, dat hoort er toch niet bij, bij Lynch?

V: Nee, nee, nee, borst is een ander gen

V:Ons moeder is inderdaad overleden, dus heeft dat veel meer indruk gemaakt als dat we nu meemaken. Dus dat is wel een stukje en bij de jongste blijf ik aan het zeuren, maar nee, dat.. 00:11:32-9

V: Nee.. een aantal van mijn vaders kant die willen het ook niet weten..

M: Nee, je hoeft het niet te weten, maar daarmee kun je wel in het onderzoek zitten, toch? 00:11:32-9

V: Ja 00:11:39-1

V: Nee, ook niet

M: ik vind het wel stressvol

M: Het is nog altijd de keus, de keus die ze maken.

V: Ja, dat vind ik ook en ik zeg ook altijd. Eentje heeft geen kinderen, de ander wel. Dan doe je het maar niet voor jezelf, maar voor je kinderen. Ik heb een andere zus die heeft 3 kinderen, 3 meiden. Hè, dat houdt wat in. Als die draagster zouden zijn van een of ander gen. Hè, dat is natuurlijk wel een stukje. Een van hen kan het nu afsluiten want zij is geen draagster en kan het dus niet doorgeven. Dus dat is het stukje.. maar goed.. komt nog wel..

Gesprekleidster 1: Maar, je zei, je darmen zijn verwijderd. En uw baarmoeder en eierstokken?

V: die zijn ook allemaal weg

Gesprekleidster 1: ook allemaal weg. <u>00:12:21-3</u>

V: nee, dat is als eerst gedaan, wij hebben de kinderwens gehad dus daarvoor is het nodige al gebeurd dus.

Gesprekleidster 1: oké

M: Heeft u dan ook een stoma? <u>00:12:34-1</u>

V: Nee, ik heb geen stoma.. nee, dat stukje van, nee

Gesprekleidster 1: V2?

V: Ja ik ben V2 kom uit **. Ik ben 64 jaar en in 2001 kreeg mijn zus darmkanker en gynaecologisch kanker. Die is toen met spoed geopereerd, ver weg op vakantie, acuut terug gekomen. Hadden ze trouwens daar al gezien. Toen is in 2005 haar gevraagd of ze familiair wilde doorgeven als broers en zussen, we zijn met vijven, dus daar aan mee wilde werken. Dat hebben gedaan samen en sinds 2007 kregen wij de uitslag. Dat 1 broer en ik dus drager zijn. En de andere twee niet, toen hebben dus jaarlijks onderzoek gekregen meestal bij dokter **. Eigenlijk hebben we ieder jaar wel poliepen dus dan um ".." . Dat is een beetje een geruststelling want dan beetje steeds op tijd, dan weet je van die voorzorgsmaatregelen zijn getroffen. Laatste keer dat ik nog op controle was zeiden, goh wanneer heb gynaecologische controle gehad? Dat is in al geen jaren gebeurd, dat heb ik nou voor een paar maanden terug gehad en dan meten ze de grootte van de baarmoeder op en dat bleek prima te zijn. Dus gaan we nou weer om de anderhalf jaar ongeveer om de controle te doen. Bleek dus uit mijn moeders kant in hun gezin, zeg maar, waren die problemen darmen en ook een neef heeft ook darmkanker.   # 0:14:26-4#

Gesprekleidster 1: Oké en M2? <u>00:14:26-3</u>

M: ja, M2, 62 jaar. Ik ben toen ik 42 was, kreeg ik darmkanker, toen was niet bekend of er wel of niet wat was. Mijn moeder is ook ooit geopereerd aan darmkanker. Toen ben ik in daar tussen in ergens 3 of '94 ben ik via de achterdeur bij Professor 2 terecht gekomen. Want ik voldeed niet aan de ** criteria voor DNA onderzoek. Maar via de achterdeur ben ik bij hem terecht gekomen en heb ik DNA onderzoek gedaan. Dat

was nog in het stadium dat je negen maanden moest wachten totdat je de uitslag kreeg. Dus, ik ben drager, daarna ben ik in '99 voor de tweede keer geopereerd, toen is mijn hele dikke darm verwijderd. "Een ..*Pouch*" gekregen dus het laatste stukje dunne darm is omhoog gezet. Um, mijn moeder is voor de tweede keer geopereerd, maar die heeft verder nooit geen DNA onderzoek laten doen. Mijn broer en zus wel, broer is geen drager, mijn zus wel. Is ook geopereerd aan darmkanker, ondertussen ook geopereerd aan longkanker, maar dat heeft niets met elkaar te maken. Ik ben zelf nu ook bij de huidarts want ik had op een gegeven moment een zo'n keratoacanthoom. Zo'n bult, daarvan kunnen ze niet zeggen of het wel of niet kwaadaardig is. 00:15:50-1

Gesprekleidster 1: Wat voor een bult?

M: Keratoacanthoom. Dat lijkt een soort dikke wrat maar de je hebt het syndroom van Muir-Torre, dat is het zelfde, hangt een beetje vanaf hoe je bij Lynch komt. Je kunt huidkanker hebben, die heet ".." via die huidkanker blijkt dus ook dat mensen darmkanker hebben. En dan kom je dus ook bij Lynch terecht. Dus, je kunt als Lynch drager ook last krijgen van huidkanker. Dus je moet daar ook heel alert op zijn. Alleen die ".." is bijna niet bekend als mankement. Ja, als je er op gaat zoeken kom je het wel tegen, hoor, maar ze waarschuwen er heel nadrukkelijk, omdat de Lynch is in wezen het reparatiegen niet goed. En daar krijg het meest last in je darm of andere plekken. Maar, onder andere in je huid kan het dus ook. 00:16:48-0

V: Nee, ik vraag het me gewoon af.

M: Nee, maar goed, daar doen ze verder geen onderzoek naar, want je ben toch al drager. Maar er zijn dus mensen die komen bij de huidarts met klachten en dan blijkt dus dat ze ook darmklachten te hebben. En zo komen ze dus eigenlijk ook bij Lynch, maar omgekeerd als je Lynch hebt wordt er niet gekeken of je ook huidkanker hebt.

Ik heb drie kinderen, drie dochters. Die 3 dochters hebben geen DNA onderzoek laten doen. Maar die zitten wel in het screeningcircuit. En die zitten ook in het gynaecologisch screeningcircuit. En ze zijn ook alle drie aangemeld bij de *spoed*, dus op zich als ze zelf niets doen, dan krijgen ze via het *spoed* een signaal of ze wel of niet voor controle gaan. De kinderen van mijn broer, die mijn broer, hebben ook geen DNA onderzoek laten doen. Die zitten wel in het controle circuit. En mijn neven en nichten ook, die heb ik allemaal gewaarschuwd, maar die kijken allemaal een beetje om zich heen en ik heb mijn best gedaan en ze zoeken het maar uit.

V: Ja maar, ook mijn zus had ook helemaal aan mijn moederskant, zeg maar, dit aangegeven en hebben 9 van de 10 zich laten onderzoeken. Ook kinderen van mij, broers en zus hebben allemaal match gemaakt, maar niemand is drager 00:18:14-6

V: Nee, van de kinderen niet. Ze hebben echt zoveel geluk dat alle goede match hebben gemaakt. Dat is wel heel frappant. 00:18:25-0

M: Ik ben bezig met een stamboom onderzoek en kun je toch vanuit mijn moederskant, want het komt waarschijnlijk van moeder als je dan de lijn volgt, dan zie je toch dat relatief jonge mensen overlijden. Alleen heb je daar geen bewijsstukken van, maar je kunt wel een lijn volgen van het moet uit die richting komen, want daar zijn alle mensen jong. Ja, in 1800 ging er wel meer mensen jong dood.

Ontvangen informatie

Gesprekleidster 1: Ja, dat klopt. Daar was de oorzaak gewoon niet bekend. Oké, en wat voor informatie heeft u ontvangen nadat u te horen had gekregen dat u het Lynch syndroom had? 00:19:06-3

M: In de tijd bij Professor 2, was er maatschappelijk werk bij. Daar heb ik contact mee gehad en eigenlijk verder niet zo veel. In wezen waarschuw je familie en informatie over of het MLA1 of MSA2 of wat dan ook was dat is dan wel genoemd, maar wat dat inhield, dat weet je dan eigenlijk niet. En de meeste informatie krijg je uiteindelijk toch via jezelf, via internet. En op een gegeven moment tussen vereniging HNPCC die is nu samen met Polyposis. En als je dat op een gegeven moment volgt dan krijg je toch op een gegeven moment wat meer informatie in der loop der tijd.

V: Ik meende al toen mijn zus geopereerd werd, daarna toen we te horen kregen dat mijn broer en ik drager waren, had ik in eerste instantie direct in mijn hoofd, van doe ik wel gezond genoeg eten en leven? Want mijn zus deed toen ze kanker kreeg het Moermandieet en die heeft een patroon van eten, die raakte heel erg aangepast. Dat had ik ook toen met mij, waar ik zo van opkeek, is dat je daar niet zo heel veel kan beïnvloeden. Natuurlijk is het altijd om een gezonde leefstijl te hebben door voeding. Ik dacht daar kan ik meer mee doen. Maar ja, als je drager bent, dan ben je drager en kun je het toch evengoed krijgen. Alhoewel ze wel zeggen eet geen rood vlees, vind ik niet lekker, dus daar heb ik geluk bij. Die soort dingen, mate van alcohol.

Gesprekleidster 1: Daar komen we zo meteen nog op terug.

V: Bij mij nu pas met dat GeoLynch gebeuren, ik heb meteen aan de arts gevraagd, moet ik anders gaan eten. Het had geen enkel nut, dus dat had ik ook niet gedaan. En dan fiets ik hier naartoe en dan voel ik me daar toch schuldig over, dan denk ik, oh god, ik moet veel meer groente eten en veel meer fruit. En dan denk ik, ach ".." 00:21:12-3

V: Dat verschilt ook een beetje per twee jaar lijkt het wel. Dat je de ene keer te horen krijgt daar heb jij geen invloed op.

Gesprekleidster 1: Maar die inzichten die veranderen natuurlijk ook. Het is ook niet.. 20 jaar geleden werden er weer hele andere dingen gezegd, toen was er veel minder bekend dan nu. Er komt steeds meer informatie beschikbaar en het laatste wat wij willen, wat iedereen wil, is dat je daar schuldig over gaat voelen. Dat is helemaal niet nodig. Nou ja, we komen zo meteen ook nog op leefstijladviezen terug. Voel je vrij.. we snappen het.

M: veel onbekendheid, want je kunt wel vragen van: kan ik naar diëtetiek? En kan die mij wat vertellen, dan kom je ook niet veel verder, want die weten ook niet. #h:06-3#

Gesprekleidster 2: Dadelijk komen we terug op wat er wel bekend is. Misschien is het goed daar even verderop in te gaan #2:10-4#

Gesprekleidster 1: En hè, wat voor informatie heb je toen ontvangen? Toen jullie het te horen kregen?

V: Nou, toen heb ik wel te horen gekregen. Probeer een gezonde leefstijl aan te houden.

M: ik heb niks te horen gekregen

Gesprekleidster 1: Dat was ook recenter, hè? 00:22:29-2

V: Ja, het was recenter als de anderen, ja. Ze zeiden van, je hebt het niet helemaal in de hand, want je kan het krijgen omdat je dus drager bent. Nou, dat heb ik dus een plekje moeten geven en dat lukt ook best wel. Maar probeer gewoon een gezonde levensstijl aan te houden. Doe niet roken, doe ik toch al niet. Alleen ja, ik heb wel enkele malen een sigaretje gerookt voor de gezelligheid toen ik in de tienertijd was, dus het was goed dat ik niet rookte. En beweeg voldoende en let op je voeding. Nou dat wist ik al, want mijn zus had zo resoluut haar eetpatroon gewijzigd.

Gesprekleidster 1: Nadat ze te horen had gekregen dat..

V: Na de operatie, hè 00:03:07-4

Gesprekleidster 1: Ja, precies 00:03:06-7

V: En omdat ze dus ook Moerman dieet natuurlijk ging doen, daar had ik ook al wat van meegekregen. Dat was alleen dat moet je wel doorvoeren "..", want ik was nooit zo zwaar geweest. Dus moet je ook opletten dat te licht in gewicht wordt, want als je ooit ziek zou worden. Maar ja, vond wel dat ik daar wel ook van ooit

de kant van het ziekhuis. Ik heb in eerste instantie nooit de behoefte gehad om steeds, zeg maar, via maatschappelijk werk of via psycholoog, want je moet toch uiteindelijk zelf verwerken en in mijn eigen kring van mijn familie en eigen gezin kon ik toch voldoende over praten naar mijn gevoel, dus daar had ik geen behoefte aan. Ja, het enige wat dan.. ze hebben mij later ook nog eens gebeld, als ik had gewild had ik toch nog gesprekken kunnen krijgen. En dat daar weer uitgekomen wat is dan belangrijk, maar die behoefte was er op dat moment niet zo. In wat ik straks aankaartte was eigenlijk, viel me echt tegen, ik dacht nou als ik nou veel groente veel fruit noem maar op de gezonde voeding matig alcohol echt goed zorgen dat je voldoende beweegt en op gewicht blijft, nou dan heb ik in ieder geval minder kans, maar ja, zo werkt het dan niet. 00:04:23-5

Gelach 00:04:23-5

V: Weet u het wel? *lachend*

V: Ja, ik weet het wel, ja, ik ben 26 kilo afgevallen de afgelopen de afgelopen twee jaar en toen heb ik borstkanker..

V: Ja, knap! 00:04:31-3

V: Ja, naja, knap? Dat schuld gevoel, hè? 00:04:38-4

V: Nee, naja

V: niet specifiek, maar het speelde mee? 00:04:37-6

V: ik heb een gehandicapte zoon, dus ik moet wel erg in leven blijven. Die nood is, die verantwoording is heel erg aanwezig, dus ik had wel zoiets van nou moet ik iets aan doen. Maar ja, toen werd ik toch ziek, dat ondermijnt dan zo alles waar ik in geloof. Dat ik denk, nou ik ga maar heel gewoon doen want mijn oma is 93 geworden op aardappels, groente, vlees. En af en toe een appeltje en een uitgeperste sinaasappel. Nou, dan zal ik toch ook wel op kunnen.

V: Dat doe ik ook, want ik heb een gehandicapt kind dus het is altijd hetzelfde.

M: Die gingen op tijd naar bed en altijd hetzelfde patroon. 00:05:11-8

V: ja, dat heb ik ook de hele dag hetzelfde ja, dus in die zin zit ik goed

Gelach

Gesprekleidster 1: Hoe was dat voor jou V1, wat voor informatie heb jij gekregen toen je te horen kreeg dat je kanker had? 00:05:29-0

V1: Ja, ik heb het, het enige wat ik weet, dat voor mij echt heel erg speelde van, jeetje, wil ik kinderen, wil ik geen kinderen, ik had net een nieuwe vriend, um. Moeten we er even spurt erachter zetten, wat we uiteindelijk gedaan hebben en verder ja, ik hoor de haar maatschappelijk werk ik ben een jaar erg depressief geweest omdat ik al het nodig achtige heb gehad in het leven. En ik had zoiets van nee, dit kan gewoon niet waar zijn. Niks. Ik kan me niets herinneren.

V: maar, was je dan ook in het *ZKH5* hier? 00:06:08-6

V: Nee, ik was nergens. Het stoet kwam me bekend voor en ik ben in *ZKH3* geweest en daar werd het je gewoon medegedeeld min of meer. En um, daarna ben ik naar huis gegaan ja en ben ik heb een paar maanden achter het raam gezeten dat ik dacht ik ga dood. Ook heel hard kwam het binnen en heel groot, ook omdat natuurlijk de hele familie van mijn vader. Ik had net een nichtje, die was net te laat in die screening gekomen, die had al eierstokkanker dus die was al ja aan het dood gaan en misschien een beetje niet diplomatiek, maar dat ik dacht ik heb jarenlang bij de huisarts gevraagd is het erfelijk? Is het erfelijk? En

dat was het niet en nu heb ik iets ja en ja, naja, schijnbaar ben je heel flexibel. Ik had zoiets van jeetje, ik ben uitverkoren want ik sta onder controle en ja, iedereen houdt mij heel goed in de gaten. En dat is eigenlijk ook wel, ja 00:07:10-2

Gesprekleidster 1: een voordeel? 00:07:10-2

V: Mijn vriendinnen zeg altijd wij zullen het wel eerder krijgen dan jij, want jij staat overal.. dus, zo ervaar ik het nu. Maar informatie over eten of dat ik met iemand zou praten, of diëtiek of.. niets. Er wordt nooit over gepraat. 00:07:29-8

M1: Nou, bij mij ook ongeveer precies hetzelfde, het was bij mij, was het dus mijn operatie stamt dus uit 1982. Dus het is al een heel eind op weg en ja dat is ik heb net nog gezegd. Dan stap je ‘s morgens binnen, 9.00 binnen, denkende dat je gezond bent en ‘s middags om 14.00 sta je buiten en dan weet je dus dat je, hè. En het werd eigenlijk meteen meegedeeld, dat je toch de hele dikke darm eruit gehaald werd. En ja dan ga je naar huis toe en dan denk je dat je je rest van je leven als een halve invalide zult moeten slijten. Als je het haalt zou ik nog zeggen en um, ja, dus ja, dan blijk dat achter af wel allemaal mee te vallen. Maar ik denk toch dat in die tijd de informatieverstrekking heel beperkt was, denk ik. Tenminste, ik kan me hier niet van herinneren dat we zeg maar, dat je dus, dat ik erop gewezen ben dat ik geen, ik zal maar zeggen geen, nou ja, dat ik natuurlijk gezonde leefstijl, na ja, dat kom ik mezelf niet tegen. Het zal niet zijn dat ik super gezond leef, maar dat wel, ja in mijn ogen redelijk normaal bestaan *ZKH3* dat wat mij betref niet zo van, zou kunnen komen.. Maar, ik zal heel ongezond geleefd hebben want ondertussen ben ik in 2005 ben ik geholpen aan een slokdarmtumor. En voor ik daar, ben ik toevallig, ik hoorde toevallig over huidkanker dat het er eventueel bij in zou zitten vond ik wel, want tussenhaakjes, leuk om te horen. 00:09:21-3

M: *lacht* want, dit was ja, ik ging met een vlekje naar de huisarts toe, die eerst zei dat het een schraal plekje was.

M: ja 00:09:34-6

M: En na een half jaar zalfjes smeren bleek het toch geen schraal plekje te zijn, maar bleek het de ziekte van Bowen te zijn. 00:29:39-3 En wat het dan ook, de ziekte van Bowen schijnt een voorstadium te zijn van huidkanker dus het is geen huidkanker maar het schijnt wel een voorstadium te zijn. Nou daar ben ik voor behandeld en het is weer, ja nou, het lijkt redelijk rustig. Dus ook daar neem ik aan dat dat voorlopig weer onder controle is. En ik zou ook zeggen, je denkt dat wel eens, dat heb ik heel erg gedacht bij mijn slokdarm tumor, wat per ongeluk goed gegaan is, zal ik maar zeggen. En ik heb heel erg gedacht, nou ben ik, zeg maar, 23 jaar onder controle geweest voor mijn dikke darm kanker. Je zou eigenlijk toch tussen haakjes en heel hard gezegd de pijp uit gaan door slokdarmkanker. Dan denk ik van: wat is het dan, waar zijn we dan mee bezig? Je wordt van alles onderzocht, je wordt jaarlijks onderzocht. Maar dan neem ik dan maar aan, puur op die dikke darm hè en terwijl dat je dus te horen krijgt in één keer dat je een slokdarmtumor hebt. En wat veel erger, veel heftiger was als wat ten minste met de dikke darm kanker dus meemaakte. 00:31:19-4

Gesprekleidster 1: V4, wat voor informatie..? 00:31:17-7 00:31:20-8

V4: Ja, bij mij was het, ik kwam bij die dame van het onderzoek die zeiden je bent drager doorsturen, en verder eigenlijk niets. Nee, nadat ik ze had gesproken, je wordt ingepland om te opereren en dat was het. Dus ik kon niet echt zeggen, ik heb zitten denken, ik heb niemand gesproken en er is eigenlijk ook aangeboden. 00:31:41-8

Gemiste informatie

Gesprekleidster 1: En wat voor informatie heeft u gemist?

V4: Ja nee, eigenlijk ik vind dat ikzelf niet zo, want ik denk van als het nodig is doe je dat zelf wel. Denk ik hè? ".." 00:31:51-7

V: Nou, ik moet ook eerlijk zeggen dat ik dat toen niet gemist heb, maar nu de vragen zo op tafel ligt, denk ik wat raar eigenlijk dat je zoiets te horen krijgt en dat je dan maar moet uitzoeken.

V4: Maar ik heb het ook niet ervaren van ik moet zo nodig naar iemand toe of ze hebben het mij niet verteld. Dus ja, nee, eigenlijk niet, het enige wat ik weet kwam op controle. Ik ben bij mijn diëtiste geweest en verder.

V: Wel naar een diëtiste geweest? 00:32:17-7

V4: Ja het ging allemaal niet zo ".." eventjes een steuntje nodig maar verder, maar verders, ja, nee, ik moet zeggen dat als ik terug denk ik dat ik, nee, we hebben daar gezeten, heb naar haar geluisterd. Nou ja, goed, dan maak je een afspraak en vervolg dat komt en we zien wel. 00:32:34-9

V: Maar, het lag ook heel erg in de week denk ik zoals jij dat vertelt. Het lag heel erg in de lijn even wachten.

V4: Ik was iedere keer versteld van het één en het andere kwam er weer aan. Je bent er ook zo niet bezig geweest. Denk van ziek is ziek dat moet eruit, want ik wil nog wel even. Dat is dan.. #2#

Gesprekleidster 1: Hoe is dat voor de anderen? Hebben jullie informatie gemist op dat moment is er specifieke informatie die u graag had willen hebben en die u niet heeft gehad? Het is een beetje raar, het is al een beetje aan bod gekomen hoor. 00:33:03-6

V: Ik vind dat wel van die huid, wat ik nu hoor. Ja, dan moet ik ook zeggen, ik ben al een paar keer aangestipt.

V: ja, want dat heb ik al jaren, oh ja, ik moet weer, want ik zie dat het er zit.

V: mag ik er dan vanuit gaan dat je met het Lynch syndroom toch eigenlijk verhoogd kanker hebt op alle soorten kanker. Want dat gevoel heb ik al heel lang namelijk. 00:34:07-7

V: Dat is toch niet waar. 00:34:07-7

V: Want dat hoor ik in huid, slokdarm in de borst, Je hebt de twee genen.

M: Baarmoeder 00:34:17-8

V: Ja, maar dat hoort bij het Lynch.

M: Ja, maar dat zit allemaal in dezelfde 00:34:25-2

V: Ja, maar borst zit niet bij Lynch, slokdarm zit niet bij Lynch

V: Ik heb zoiets van, ik heb hier dan ook een vlekje op mijn gezicht, dan vertrouw ik erop dat hij toch kan zien of ik dan daarmee naar de huidarts moet. En anders denk ik van, ja, laatst was er ook iets in mijn hart onderzocht, dan heb ik nou heb ik dus eigenlijk genoeg 00:34:45-7

M: oh nee, maar dat is ook prima. Maar, het is, je moet je er bewust van zijn dan er meer kan zijn dan alleen maar een plekje.

V: maar het is toch van de zotten, dat ik dat van jou moet horen en niet van

M: ik kom bij de huidarts of bij de huisarts omdat ik een bult heb, dikke bult heb aan mij wenkbrauw. Dan zegt de huisarts, oh, die kan ik wel wegsnijden. Ondertussen lees ik een artikel over muir torre, had ik ook nog nooit van gehoord, dus ik zeg tegen de huisarts: ik wil liever naar de dermatoloog, maar de dermatoloog zegt, dat snijd ik zelf niet aan, dat doet de plastische chirurg. Ik bedoel, de eerste stap is de huisarts. 00:35:33-4

V: Mijn huisarts wist niet eens wat HNPCC was. 00:35:51-7

V: Ja, maar daar kom je niet mee weg. Daar ben ik het niet mee eens. 00:35:54-1

M: Nee, nee, dat is wel waar. Die huisarts had het moeten weten, of die had er voor open moeten staan. Maar de kans dat ze iemand tegenkomen per jaar is gewoon heel klein. Ze krijgen heel veel mensen met "oude mensen kanker", zeg ik altijd. Gewoon endeldarm kanker, omdat je oud bent. Maar niet de jonge mensen, alle mensen met Lynch, die hebben een opstijgend deel en een dwarse deel. Daar zit bijna altijd de kanker. En de meeste mensen krijgen het in hun neergaande deel. Ja, het is ook, als je een darmonderzoek doen bij ouderen, beginnen ze altijd sigmoïdoscopie, alleen het laatste stukje. Terwijl je voor normaal, de Lynch, de hele darm moet zien tot aan de dunne darm. Maar, je wordt heel vaak voor gek versleten, ik kwam dus ook niet in aanmerking voor het DNA onderzoek, maar je voldoet niet aan de criteria. Want mijn grootmoeder was overleden, alleen dat was in de oorlog, daar was verder niets over bekend. Ja, en mijn moeder had darmkanker gehad, maar ik bedoel, zo kan ik.. 00:37:06-1

Meerderen: ja 00:37:06-1

M: Dan word je dus door je internist weggestuurd van ja, nee, het voldoet niet.

Gesprekleidster 2: Wat misschien wel belangrijk is om te noemen, is dat er wel individuele verschillen zijn. Wie er een verhoogd risico heeft, waarop, er wordt met familie geschiedenis ook bekeken. Mogelijk dat sommige vormen van kanker niet genoemd zijn, maar dat er een reden voor is om dat niet te noemen.

V: Ik begrijp niet wat je zegt. 00:37:31-3

Gesprekleidster 2: Nou, dat er verschillende genmutaties en die hangen samen met verschillende vormen van kanker die verhoogd zijn, dus mocht u iets anders horen van iemand anders, betekent dat niet dat in uw situatie precies hetzelfde is.

V: Oké 00:37:51-7

M: Welke mutatie heeft u?

V: Nou, daar zou ik mijn boek op na moeten slaan, daar heb ik ooit dat moet ik heel eerlijk zeggen. Ik vind het heel interessant om met gelijkgestemden hier te zitten, maar het is me niet meer iets wat me dagelijks bezig houdt. 00:01:06-0

M: Nee, maar wat zij bedoelt is of je MSA2 of MSA1 hebt? 00:01:14-8

V: Volgens mij 2 00:01:15-2

M: Want dan kan het zijn dat je wel meer kans op, er zijn ook sommige die last hebben van hun dunne darm, daar wordt niet op onderzocht. Alleen op basis van familie, maar dat speelt bij de één meer als bij de ander. 00:01:25-7

V: Ja, huid heb ik nooit gehoord, nierbekken heb ik ooit iets van gehoord, ben ik ook nooit op onderzocht. 00:01:32-6

V: Hebben ze ook tegen mij.. heb ook de nierfunctie laten prikken. 00:01:34-2

V: Nog nooit gedaan 00:01:36-3

V: Heb ik drie keer gedaan, de huisarts zei: ga je daar dan niet te ver in? Kies daar zelf in. Wil je dat nog steeds ja of nee? Toen heb ik ook gezegd dat wil ik niet meer, want het kan ook zijn je kan ook iets in de nieren krijgen terwijl dat helemaal los staat van. 00:01:51-1

V: Maar, in het begin van mijn onderzoeken werd wel mijn urine onderzocht, maar dat, waar dat is gebleven? 00:01:55-6

V: Nee, ik vind het ook heel mooi, mijn broer zit in het *ZKH6* en daar doen ze altijd heel uitgebreid bloedonderzoek. 00:02:02-1

V: Dat klopt ,mijn zus zit ook in het *ZKH6*. 00:02:02-1

V: Ik word nooit meer geprikt. Vind ik ook zo apart, terwijl daar die informatie is en hier is die informatie, geef ik elke keer door. Ik krijg alleen een jaarlijks onderzoek, ik vind het ook allemaal prima. Maar dan denk ik van, als je hetzelfde hebt kan er zo'n andere benadering zijn. 00:02:16-3

M: Omdat die tumormarker van de darmkanker niet zo veel oplevert, zeggen ze dan. 00:02:22-7

V: Zegt u nog eens een keer? 00:02:22-7

V: Je hebt een tumor marker CEA en die daar komt niet zo vaak, wat uit, ze hebben meer aan de scopie, levert meer op dan het bloedprikken. Voor de prostaatkanker, die reageert veel sterker als tumormarker. En dat is bij de darmkanker minder. 00:02:46-8

V: Maar, ik word geprikt voor de marker van baarmoeder en eierstokken maar nog zegt hij tegen mij vorige week van, gezien het feit dat je afgelopen jaar met je borst geklooi hebt gehad, het ziet er allemaal prachtig uit daar van binnen. Heeft hij allemaal besnuffeld en bekeken. Ziet er allemaal keurig uit, normaal werd ik dan naar huis gestuurd. Nu niet, hij wil op celniveau gaan keuriteren, heb ik nog geen twee jaar geleden.. Maar, hij wil het extra in de gaten houden. Dan ga ik naar huis, waarom wil hij het extra in de gaten houden? Heeft dat dan met die borst te maken? Weet je wel, dat zijn van die korte opmerkinkjes dan zit je op de fiets en denk ik: wat maakt nou eigenlijk dat hij het extra in de gaten wil houden? 00:03:24-9

V:Soms denk ik, is dat dan dat de wetenschap daar meer aan heeft? Als jij, hoe ver ga je daar in, hè? Van willen de artsen.. 00:03:36-1

V: Dat soort vraagjes ik zou dan meteen willen bellen met iemand en kunnen vragen van goh ik kom net thuis hij heeft dat en dat gezegd maar waarom nu extra?00:03:40-8

Gesprekleidster 1: Daar zou u meer informatie over willen uiteindelijk? 00:03:46-5

Gesprekleidster 1: Of u vraagt het hemzelf he, wat hij daar meer informatie over wil geven, waarom zegt hij nou eigenlijk zoiets? 00:03:51-6

V: Maar op dat moment 00:03:51-6

Gesprekleidster 2: Denk je daar niet aan. 00:03:51-6

V: Hoor ik dat niet, dat hoor ik pas als ik naar huis ga. Goh vorige keer zei hij het is goed toen ging ik gewoon naar huis ik zie je over een jaar. En nu is het 00:04:00-9

Gesprekleidster 1: Misschien is het een idee om de volgende keer als u terug gaat die vraag toch nog is te stellen. Van waarom is dat eigenlijk dan? 00:04:11-0

M: Schrijf je vragen vooraf op een papiertje 00:04:15-3

V: Ja, maar ik zie hem pas over een jaar. 00:04:16-4

M: Maakt niet uit, als je er slecht van slaapt moet je.. 00:04:20-4

V: Nee, ik slaap er niet slecht van. 00:04:20-4

M: Nee, maar als je gaat, schrijf al je vragen vooraf op, want een specialist vraagt maar 1 ding: Hoe gaat

het? Jij zegt goed en dan gaat hij naar de volgende bladzijde, dan is hij alweer klaar. Op het moment dat jij je briefje hebt zegt hij geef dat briefje maar hier, al heb je 10 vragen loopt hij ze allemaal af. Onthoud 1 ding: de mondige patiënt leeft langer. 00:04:43-8

V: Ja, dat wordt vaak gezegd. 00:04:44-6

Belang van informatie

Gesprekleidster 1: Ik ga weer heel even terug naar de vragen, want we gaan een beetje afwijken van het onderwerp. In hoeverre vindt u het belangrijk om informatie te ontvangen met betrekking tot leefstijl? 00:05:00-9

M: Ik vind het fijn. 00:05:03-6

Gesprekleidster 1: Oké, u vindt het belangrijk om daar informatie over te krijgen. 00:05:06-7

M: Ja, of je dan, um, als je er niets mee doet is dat je eigen keus. 00:05:13-9

V: Ja, ik vind het ook heel belangrijk. 00:05:15-5

Meerderen: ik ook 00:05:15-8

Gesprekleidster 1: Vindt u het ook belangrijk om informatie daarover te krijgen? 00:05:16-1

M: Ja, ik weet niet of ik het, um, ik denk dat ik het wel doorlees, maar ik weet niet of ik meteen mijn hele, net werd er al gezegd, hè, dat als je er wat mee wilt doen, dan kun je er wat mee doen en anders doe je er niet wat mee natuurlijk. Maar, ik heb zoiets van, ik heb het nou toch, ik kan nou nog tien kilo fruit per dag eten, maar daar wordt het niet anders meer van hè? 00:05:51-3

V: Je krijgt er diabetes van, dat heb ik gehad. 00:05:52-7

Gelach 00:05:54-4

Gesprekleidster 1: U heeft ook diabetes nu dan? 00:05:59-0

V: Door te veel fruit voor de kanker 00:06:02-9

V: Maar, als er ooit een gesprek komt over darmkanker of wat voor vorm van kanker ook. En dan zegt men van, ja, maar mijn moeder is 90 geworden en die rookte en toen dronk ze ook een borrel en dan ben ik altijd heel heftig. In de zin van, ja, maar we leven in een andere tijd. De meeste mensen bewegen minder, eten ongezonder en noem maar op. Dan ben ik daar altijd een beetje heftig naar toe. En stress. 00:06:26-4

Verbeteren leefstijl

Gesprekleidster 1: En heeft u in het verleden geprobeerd om uw leefstijl te verbeteren? 00:06:33-0

V: Als je denkt dat je het goed doet, dan is het goed hè? 00:06:36-8

Gesprekleidster 1: Dus u heeft geen reden gezien om uw leefstijl te verbeteren hè, dat is wat u zegt? 00:06:42-5

V: Ja 00:06:44-1

Gesprekleidster 1: Hoe is dat voor de anderen? Hebben jullie een.. 00:06:48-2

M: Matig leven, ja, toch in die zin, dat je in wezen, ik rook niet, relatief weinig alcohol en toch proberen om qua gezonde voeding, ik eet wel rood vlees, maar ik ben van varkensvlees afgestapt. 00:07:04-8

Gelach 00:07:06-5

V: compromis 00:07:06-5

M: Nee, dat is dan weer 00:07:10-3

V: In overleg met mijn gen 00:07:10-3

Gelach 00:07:12-4

M: Maar, mijn zus is geen vegetariër, maar die eet dus eigenlijk geen vlees meer, dat is dan weer keuzes die je maakt, maar je probeert toch wel wat rekening te houden met je leefwijze. 00:07:30-3

V: Nou, dus, als je de informatie krijgt denk je er ook beter over na. Dan denk je van, ach, dat kan ik eigenlijk best laten. 00:07:38-6

V: Je moet ook eigenlijk weer die prikkel krijgen, want je komt in een bepaalde manier van leven en daar krijg je niets van mee terug. Het is een keer gezegd van, oh ja, eten van niet, vind ik altijd zo leuk want je hebt het over roken en drinken maar als je het nooit gedaan hebt, dan denk ik wat moet je dan laten? 00:07:55-4

Gesprekleidster 1: Daar hoeft dan niets verbeterd te worden. 00:08:02-7

V: Dat is dan ook dat stukje van wat is dan voor jou van toepassing? 00:08:06-8

Gesprekleidster 1: En V2, ik hoorde je net al zeggen dat je onder invloed van je zus naar je voedingspatroon hebt gekeken en daar ook aanpassingen in gemaakt had. 00:08:18-1

V2: Ja, die is nog steeds lid van de Moerman vereniging, dus dan lees ik het tijdschrift. En dat is natuurlijk voor alle vormen van kanker van toepassing, wat daar voor artikelen in staan. Nou ben ik pas met vakantie geweest. En denk, nou, laat maar even, nou ben ik in een land geweest waar ze veel vlees eten. Maar dan zet ik toch nog de rem er op met name met vlees hè. 00:08:41-4

Gesprekleidster 1: En V, hoe is dat voor jou? Ben jij? 00:08:44-5

V: Ik ben gestopt met roken. En met drinken 00:08:48-4

Gelach 00:08:51-6

V: Dat klinkt alsof ik 00:08:53-9

V: opstond met een borrel. 00:08:55-0

V: Dat was niet zo. Maar ik, nou ja, ik vond dat ik teveel dronk. Nou ben ik recentelijk wel weer begonnen 1 glaasje witte wijn per dag. En dat bevalt me prima, tijdens het koken dan ben ik zo, pff, aangezien altijd in hetzelfde ritme past helemaal niet bij mij, want ik ben een hartstikke wolle waai, dan heb ik even, nou, ik denk niet dat ik daar dood aan ga. Ik heb meer zorgen over mijn zoon. Die heeft een lastige motoriek, dus die beweegt weinig. Um, ik ben 25 keer bestraald en ik ben nog steeds heel erg moe, dus ik heb weinig energie om hem op mijn schouder, met hem te gaan fietsen en god mag weten wat. Dus dat ligt op zijn gat een beetje, hij eet slecht groente, te weinig denk ik. Ja, daar gaat dus een wortel in en een uitgeperste sinaasappels in en een appeltje en ja ,dat vind ik wel lastig, daar zit wel een stuk zorg, meer om hem. Ik ben ook geen groente geen fruit eter, ik heb überhaupt niet zoveel met eten. Ik bedoel, ik moet op de been blijven, maar of dat nou met een salade is of een droge boterham met pindakaas. Dat maakt mij niet zoveel uit, maar ik, ja, voor de kinderen, daar heb ik zorg over met name met eten. Ik zou bijvoorbeeld willen dat ik wist, hij heeft ook last van de ontlasting omdat hij te weinig beweegt en hij is onrustig dus hij gaat niet lang genoeg zitten blablabla. Dus dan denk ik, dan wil je voorkomen, dat ik zeker weet dat hij binnen heeft wat hij

nodig heeft. 00:10:00-0   00:47:30-2

V: Dat zou dan eventueel bij een homeopathische arts kunnen, want die mijn zus vanaf die tijd dat zij Selium en Magnesium nog iets, ben even de naam kwijt, blijven slikken. Vanaf 2001 is zij en dat zou natuurlijk kunnen helpen. Ik dacht nog, goh, ik heb iets aan mijn darm, bleken het aambeien te zijn. Kreeg ik achter elkaar een drankje mee, krijg je zo een opgeblazen gevoel van. Dat was, ik weet niet meer precies wanneer dat was. Ik dacht, oh jee, nou is het helemaal foute boel, hè, Want het was wel heftig met bloedverlies. En toen, ja, dat drankje, pfoe, mensen dat, ik denk dat doe ik niet meer. Ja, toen toch nog in de beginnende tijd van computer enzo kwamen, ja, dan maar meer groente en fruit. Want bewegen deed ik al voldoende, want dan krijg je natuurlijk betere.. 00:48:24-5

Gesprekleidster 1: V, wat was hè, je zegt ik ben gestopt met drinken en uiteindelijk gestopt met roken, hè? Wat was de motivatie daarvoor toen? 00:48:33-5

V: Nou, sowieso voor de kinderen, want ik stond in de tuin een peukje te roken en dan kwam mijn dochter naast mij staan mij staan met een soepstengel. Toen dacht ik, ja, dit kan eigenlijk niet. Ja, en voor de gezondheid, ik bedoel de gezondheid “in general”.   00:48:56-0

Gesprekleidster 1: de algemene gezondheid. 00:48:56-0

V: Ja, gewoon van de 1 op de andere dag, klaar mee was.   00:48:58-5

Gesprekleidster 1: En M1, heb jij iets aangepast in je leefstijl? 00:49:04-9

M1: Ik kan me niet, ja, ik heb vroeger gerookt en ik nu rook nu niet meer. Maar ik kan me niet voorstellen dat ik dat gedaan heb, want ik ben al voor die tijd, allang voor die tijd, gestopt voordat ik überhaupt wist dat ik dat gen had, laat ik het maar zo zeggen. Dus toen ben ik al gestopt met roken, nou, ik kan me niet voorstellen dat ik verder echt heel veel aan mijn levensstijl aangepast hebt. Bewegen deed ik volgens mij meer dan genoeg, dus daar ligt het niet aan. En ja, de hele onbenullige leefstijl heb ik nooit gehad. Dus nee, ik kan niet zeggen dat ik nou heel veel aan mijn levensstijl aangepast heb. 00:49:47-1

Gesprekleidster 1: Oké 00:49:47-1

V: Dat wil niet zeggen, het is niet zo dat je bewust aan je levensstijl gedaan hebt, maar je kunt gewoon niet meer alles eten. Ik zit eigenlijk te denken van bewust maar ik kan niet alles eten. 00:49:58-1

V: Ja dat zal inderdaad anders zijn voor mensen die.. 00:50:00-9

Gesprekleidster 1: Maar waar heeft u dan wat kan u bijvoorbeeld niet eten nu? 00:50:02-8

V: Ik kan bijvoorbeeld geen kerrie, geen paprika, ik kan kwijt, nee, dat kan ik niet meer. Dus op zich heb ik niet bewust wat gedaan. 00:50:16-3

Gesprekleidster 1: Maar toch het voedingspatroon is aangepast vanwege.. 00:50:18-4

V: Ja, omdat ik het gewoon niet kan verdragen. Ik heb het niet bewust gedaan. 00:50:18-7

Gesprekleidster 1: Is dat dan ook vanwege de operatie?   00:50:24-6

V: Ja, ik um. Nee, het zijn echt dingen wat ik gewoon um bijvoorbeeld vlees "..". Ja, ik denk gewoon van, oké, dat kan niet meer. Dus dan zal ik ook echt niet.. 00:50:39-9

V: En ze hebben bij u de hele dikke darm? 00:50:44-4

V: Klein stukje. 00:50:44-4

V: Klein stukje eruit? 00:50:44-4

V: nee, klein stukje zit er nog. 00:50:47-6

V: En wat heeft dat dan voor invloed? 00:50:49-7

V: Nou goed, ik ben altijd aan de diarree, dus drinken is heel belangrijk 00:50:57-8

V: uitdroging? 00:50:57-9

V: Ja, daar heb ik dan ook last van. Dus op zich is dat, het lijf geeft dat wel aan. Ik eet liever, zal ik maar zeggen, kleine hoeveelheden, dat werkt nog altijd beter als dat ik dus in één keer heel veel eet. 00:51:13-9

V: Maar, belemmert het je dat ook om een dagje uit te gaan? 00:51:16-3

V: nee hoor, we zijn afgelopen week op vakantie geweest. En ik weet precies wat ik eet, kijk ik eet niet meer voor, midden en na. Ik eet het tussenstuk of ik eet twee kleine voorstukjes. Nou prima, dat is eigenlijk eigen geworden. Dat is wel veranderd natuurlijk, maar ja, dan houdt het gewoon op. En als ik denk, oh, ik baal dat ik dingen niet kan proeven, nou dan laat ik mijn man proeven. En dan zegt hij: ik wil het scherper. Dat risico loop ik niet. 00:51:44-7

v: Dan word je ook, um. 00:51:43-1

v: En anders dan stimuleert je darm natuurlijk. Daar heb ik veel last van en anders gaat het stuk. 00:51:53-8

M: Maar, ik heb dus ook geen dikke darm meer. Ik kan in principe alles eten, ik moet alleen wel op tijd eten. Anders dan heb je te weinig energie. Dus je moet echt je drie.., maar ik weet of je, ik gebruik elke ochtend een zakje psyllium vezels. 00:52:10-1

V: Oh god, nee. 00:52:10-1

V: Nee, nee, maar ik zal zeggen waarom, die psyllium vezels gebruiken normale mensen die last hebben van verstopping. Want psyllium vezels houdt vocht vast in je darm, waardoor dat je nu vocht vast houdt in je darm, is je ontlasting ook iets dikker. Dus je hebt, zeg maar, in de loop van de dag, heb je een veel rustiger patroon. 00:52:38-6

V: Is dat eigen initiatief, of ben je bij een homeopathische arts geweest? 00:52:41-1

M: Nee, op een gegeven moment gewoon via de internist. En die heeft toen aangegeven in wezen met dat verhaal. Um, ik kan in principe alles eten, ook in het buitenland. Alleen in Nederland gebruiken ze veel toevoegingen die uit potjes komen en kan je gelijk van aan de race. 00:53:04-5

Gelach 00:53:05-7

M: Nee, je kunt beter in Indonesië of Turkije, of waar dan ook Tunesië. 00:53:13-6

V: Ja, maar daar komt ook veel minder kanker voor. 00:53:15-0

V: Ja, maar dan ben je ook veel voorzichtiger. 00:53:18-3

M: Nee, maar je kunt daar beter eten als hier. Als je hier buiten de deur eet, dan kom je eigenlijk altijd uit op koffie. 00:53:27-1

V: Maar ook als je niet geopereerd bent, misschien is dat het gen. Maar, ik kan ook slecht tegen buiten de deur eten. “Darmtechnisch” dat doet iets. 00:53:33-3

M: Dat weet ik niet of dat um.. 00:53:37-0

V: Als ik met vakantie ga, is dat leefstijl natuurlijk heel vervelend, maar dat heet nu anders: Emoli, maar dan krijg ik zo een buikpijn, dat laat ik dan ook wel. Dan denk ik van, maar zakjes is natuurlijk dan toch net iets anders. 00:53:56-3

M: Het is het proberen waard. Emolium is ook vloeibaar te krijgen en dan kun je het in kleinere doseringen. 00:54:05-0

V: Ik heb nou gewoon tabletten genomen. 00:54:07-6

V: Ik zou voorlichtingsbijeenkomsten gaan houden haha, nee, echt super. 00:54:11-5

M: Nee, maar emoilium dus eigenlijk, heb je, dan krijg je eigenlijk als je normaal aan de diarree, dat legt je darm stil. Daarom krijg je vaak ontstoppingen, wordt het probleem alleen maar erger. 00:54:21-4

Gesprekleidster 1: M1, hoe is dat voor jou? Je mist ook een dikke darm. Zijn er aanpassingen in.. zijn er dingen die jij niet kan eten daardoor? 00:54:36-4

M: Ik kan eigenlijk alles eten. Maar sinds mijn slokdarmoperatie natuurlijk alles in hele kleine hoeveelheden. Een slokdarm-maag-buis operatie gedaan, dan word je maag dus omgebouwd naar een slokdarm. En dan dus, je hebt dan ook geen maag meer. Dus ik heb nu mijn voedselpassage gaat nu super snel hè. Dus wat ik vanmiddag om 12 uur eet, dan is het, kan heel goed dat dat om 's avonds om 8 uur weer in de wc pot ligt. 00:55:17-1

V: Maar, dan heb je toch een ontzettend energie probleem? 00:55:22-4

M: Ja nou, ja, ik zeg altijd, ik moet net zoveel moeite doen om op gewicht te blijven als menigeen moet doen om niet aan te komen. Eigenlijk kortweg gezegd eigenlijk moet er een handel zijn in kilo's. Ik kan amper op gewicht blijven ondanks dat ik 6 tot 7 keer per dag wat eet. Dat is heel moeilijk, mijn gewicht op peil houden. Terwijl de menigeen zeggen niet te veel, niet te veel. 00:55:52-1

V: Maar op zich kan je wel wat advies geven, hoor. 00:55:54-1

Gelach 00:55:54-1

V: Hé, maar ben je dan ook niet moe? Ik kan me voorstellen dat alles.. Het heeft tijd nodig om in je cellen te komen, maar dat gaat dus te snel. 00:56:05-6

M: Sinds mijn operatie, mijn operatie ging in eerste instantie niet goed en toen ben ik twee keer geopereerd. Ja, ik heb 45 dagen geslapen op het intensive care, Dus met, nou ja, kantje boord dat ze familie en vrienden lieten komen om te zeggen als je hem nog zien wil dan moet dat nu, maar want anders hoeft het niet meer. En als je dan dus weer wakker wordt, dan kun je dus helemaal niet meer. Je kunt nog geen arm meer van je bed tillen. Je moet weer leren lopen, als je een rondje om je bed kan lopen heb je wereldprestatie geleverd, zal ik maar zeggen. En um ja, dan kun je dus zeggen dus inderdaad, ik heb veel minder power als voor de operatie. Maar komt dat dus, want ja, je wordt ook ouder. Je bent ook al 60 geweest, maar als ik dus een halve dag lichamelijk inspannend werk doe, dan kan je aan het einde van de dag wel weg leggen. Maar ja, goed, ook dat regelt het lichaam zelf en als je niet meer wilt, dan houdt dat vanzelf op. Dus het is heel moeilijk om voldoende energie in te krijgen. 00:57:23-2

Gesprekleidster 1: Laten we kijken naar het bord, Gesprekleidster 2 is van alles aan het opschrijven. Dat zijn factoren die u leefstijl beïnvloeden. Er zijn natuurlijk factoren die uw leefstijl bevorderen of die een gezonde leefstijl beïnvloeden. Redenen waarom u bijvoorbeeld uw leefstijl zou willen verbeteren en er zijn factoren die ervoor zorgen dat u uw leefstijl niet verbetert. Die ervoor zorgen dat een gezonde leefstijl in de weg staan. 00:57:59-3

M: mag ik misschien 1 ding erbij vermelden, dat is die energie als min..

Gesprekleidster 1: Gesprekleidster 2, misschien kan jij door het lijstje heen gaan, want jij hebt het opgeschreven. Jij hebt beter in je hoofd hoe of wat. 00:58:19-5 Vul aan als u iets mist. Maar, wat ik op heb, redenen om uw leefstijl te willen aanpassen zijn om gezond te willen zijn. ".." U voelt zich verantwoordelijk voor de kinderen. U wilt daar ook voor zorgen. Ik heb schuld gevoel gehoord, als reden om leefstijl te willen veranderen. Wat verschillende mensen hadden genoemd is gevoelige darmen, waardoor je vanzelf wel oplet wat je eet. Het hebben van een ritme, op constante momenten iets eten of iets doen. Darmoperatie ligt een beetje samen met een reden daarvoor. Bijvoorbeeld uit de omgeving. Volgens mij heb ik gehoord, V2 had gezegd, dat over haar zus die aan een dieet zat wat u ook gemotiveerd heeft ".." een gewoonte dus eigenlijk. Constant gematigd leven. Het is gewoon een onderdeel van het dagelijkse leven. Omdat u al een gezonde leefstijl heeft. En ik heb gehoord dat iemand zei: informatie, als je informatie ontvangt, ook over gezonde leefstijl in relatie met kanker. Kan het net die prikkel zijn, die u er even op wijst. En die u er over na laat denken. Factoren die een leefstijl misschien op een negatieve manier beïnvloeden, is dat er andere zaken zijn waar meer aandacht naar toe gaat, zoals bijvoorbeeld zorg om een zoon. Nou, ongezonder eten smaakt volgens sommigen lekkerder een stukje vlees, of wat alcohol. 01:00:17-6

V: gemak

Gesprekleidster 2: gemak hoort daar nog bij.

V: patatje eten is ook lekker makkelijk, toch? 01:00:20-7

Gesprekleidster 2: Dat is een hele goede. 01:00:21-7

M: En het veelvuldig toiletbezoek, dat is iets wat toch je leven sterker isoleert.

Gesprekleidster 2: en aan welke kant zou u het dan willen zetten?

M: Nee, dat is negatief ook, omdat je dus..

Gesprekleidster 2: want welke aanpassingen doet u? 01:00:48-7

M: nee, ik moet 6 tot 8 keer naar het toilet per dag. En als het slecht is moet ik misschien wel 10 keer of 12 keer.

Gesprekleidster 1: Maar daardoor zorgt u dat u aanpassingen maakt in uw voedingspatroon. 01:01:00-7

M: Maar ook in je leefpatroon, want je moet op gegeven moment.. Je moet er.. ik ga niet bij een bushokje staan, ik ga altijd met de auto op vakantie, want dan kan ik zelf, al moet ik achter een bosje zitten, maar dan kan ik zelf naar het toilet wanneer ik wil. Ik ga soms naar het toilet, maar dan ben je bij de deur en dan moet je weer terug. Dan ben je klaar en dan moet je weer terug. Dan zit je een kwartier ongeveer onderweg, in het vliegtuig ook en dan kom je buiten en staat er een hele rij. 01:01:32-8

V: En om dan weer terug te gaan, hè? Dat doe je niet.

V: Dan krijg ik het gewoon benauwd. 01:01:39-8

M: Ik wil altijd in het gangpad zitten, is altijd een conflict met mijn vrouw. Ik ben voor 50% afgekeurd. Daar ben ik blij om. Dat is heel positief, ik heb geluk gehad dat ik voor 50% ben afgekeurd. Dat mag je misschien als negatief ervaren, maar voor mij is dat positief geweest, omdat je daardoor tijd krijgt om je weer even op te leven en tijd krijgt om de beperkingen die je in energie hebt weer even te compenseren. Wat M1 aangaf, je kunt een halve dag wat doen, een hele dag is er niet. Ten minste bij mij is, ik weet niet hoe het bij jou is. 01:02:23-4

V: Ik werk ook een hele week en ".." #loch-1#

V: Je komt wel heel energiek over

".."

Gesprekleidster 1: Is dat iets wat na de operatie is gekomen? Dat stuk minder energie.

M: Nadat ik voor de tweede keer geopereerd ben, toen die dikke darm is verwijderd. Dat levert zoveel beperkingen op. Toch wel, je kunt er goed mee leven, je kunt er oud mee worden, maar geeft een heleboel beperkingen. En als je op dat moment.. voor mijn gevoel ik ben gered door de arts van het UWV, die zegt u moet anders gaan leven. En dan.. 01:03:03-9

V: Voor uzelf hè? Want je heb het ter harte genomen.

M: Ik ben ook een jaar bij de RIAGG geweest omdat je dat een plek moet kunnen geven dat je dus niet meer kunt werken zoals je werkte. #3#

V: Heleboel artsen zeggen je moet anders gaan leven, ten minste ik heb ook jaren te horen gekregen je moet meer voor jezelf. Dat heb ik in de wind geslagen, totdat ik ziek werd en dacht dit is een signaal dat ik.. Ik heb aan mezelf te danken, want ik heb het eindelijk ter harte genomen en heb je ook duidelijk gezegd. Want je heb het serieus genomen.

Gesprekleidster 1: En met ziek geworden bedoel je ? 01:03:44-2

V: voorstadium van borstkanker. Dat is een signaal geweest van hier en niet verder.

V: Je lichaam zei, ho even..

Gesprekleidster 1: En welke aanpassingen heb je gemaakt. Hoe heb je je leefstijl veranderd?

V: Nou ja, ik ben al zes jaar op zoek naar werk. En dat ik werk 1 dag in de week bij een slager, tussen het rode vlees..

Gelach

V: ik haast minder, ik kwam dus ook te laat, ik kan niet meer. Vroeger ik heb ook ADHD. Kon ik om 1 minuut voor tien weggaan, jaste ik de fiets en racete ik hier naar toe en was zo, ik ben op tijd. Dat lukt niet meer. En daar doe ik ook geen moeite, ik ben lief voor mezelf geworden. En aanpassingen: tussen 1 en half drie slaap ik en dan doe ik geen huishouden komt er niemand op visite. Ik ga, zo meteen heb ik een vrijwilligersbaantje op school, overblijven met kleuters en ik heb de hele ochtend hier gezeten en daarna ben ik kapot, ben ik misselijk, duizelig, dan is het klaar. En ja, ik zeg vaker nee, en mijn huis is minder netjes. En loslaten, en dat bevalt me heel erg goed. Ik ben echt een ander mens geworden, creativiteit, het heeft me zoveel gebracht. Ik ben blij dat ik dat..

Gesprekleidster 1: Goed dat u dat gedaan heeft.

V: Ja, ik kan niet anders, het was point of no return.

Gesprekleidster 1: En welke aanpassingen? Hoe heb je je leven anders ingericht na..?

M: Op een gegeven moment ben ik, in eerste instantie ben ik, ging ik van vijf dagen werken naar maandag, dinsdag werken, woensdag vrij, donderdag weer werken en vrijdag vrij. En dan begint het weekend, ja, dan is het weekend, heb je nog weekend. En anders is het: je werkt vijf dagen en dan begint het weekend en dan moet je rustig tot maandag en kun je weer beginnen aan de week en heb je geen weekend gehad. En dat zijn dingen, ik zit op groot verlof dus ik ben verlof aan het opmaken en dan stop ik met werken. Ik ben eigenlijk al gestopt. Of het nu geld kost of niet, dat zal me op een gegeven moment een zorg zijn, want dan je kunt wel door werken tot je 66 of 67ste en daarna houd het leven op. Omdat je dan opgebrand bent en

dan kun je beter eerder stoppen. Heb je misschien wat minder geld, maar dan kun je in ieder geval verder leven. En dat zijn de afwegingen. Datzelfde is als je vaak naar het toilet moet dan is het op een gegeven moment, moet je dat zien in te passen in je hele leefpatroon en in je vakantie patroon. Wij kampeerden vroeger altijd, dat doe ik niet meer, ik wil rustig op het wc kunnen zitten en niet op een wc met boven een stuk vrij en onder een stuk vrij.

V: voor die tijd wel dus? 01:07:08-3

V: het gaat dan ook nog eens vreselijk hard waaien. Het is heel gênant. Je moet altijd toilet nakijken voordat ".." onvoorstelbaar hoe vaak dat eruit komt. En in het vliegtuig dan staat er een rij, maar je moet eerst allemaal schoonmaken en je hebt al heel lang zitten bewaren. Dus je moet eerst bekomen wat ben ik toch stom dat ik niet eerder ben gegaan. Dus elke keer is dat die wc's, het hoort bij je, maar het is niet prettig. Dat vind ik, ja, vervelend, maar ik blijf dan in de auto stoppen.   01:07:44-9

M: Ja, in de start weet je overal waar je terecht kunt.

V: Is waar, ik weet ze overal. Maakt mij ook helemaal niet uit. En benauwd is benauwd. 01:07:53-2

V: Ja, ik zit alweer te denken, maar misschien is dat juist helemaal verkeerd. Dat is dan toch ook een tekentje of iets. Ik kan me ook voorstellen, stel nou dat je in zo'n bijeenkomst zit zoals we nu zitten, wat hier niet over gaat, maar over brildragende vrouwen. Ik noem maar wat. 01:08:06-5

V: Maakt mij niets uit.

V: En je moet 7 keer naar het toilet.

V: Dan sta ik gewoon, ik moet nu eigenlijk naar het toilet doe ik het wel zo lang mogelijk uitstellen. Maar dat maakt mij helemaal niets meer uit. 01:08:17-7

M: Maar, als je naar een begrafenis moet of crematie dan ga je in de aula aan de buitenkant zitten. Of je gaat een beetje achteraan zitten, als je weg moet, kan je gaan. 01:08:27-7

V: Altijd van te voren gaan.

M: Ga je naar een vergadering? Zorg je ervoor dat je aan het gangpad zit, of aan de buitenkant. Ik bedoel, ik wil nergens midden in zitten, want dan moet door de hele rij heen. En dat soort dingen, dat is dan misschien langzaam erin gegroeid dat je daar al langzaam op let.

Gesprekleidster 2: Je bent er altijd mee bezig eigenlijk. 01:08:41-7

Gesprekleidster 1: Ja

M: Je bent er altijd mee bezig.

Ontbrekende factoren
Gesprekleidster 1: Laten we heel even gaan afronden, het is half 12. Jij moet zo meteen weg. Ik wil even nog naar de leefstijlaanbevelingen toe, die ik zo meteen ga noemen. En ik zou heel even snel nog willen weten of hier nog iets aan ontbreekt op dit bord wat we er nog bij kunnen zetten. Dat zijn 2 dingen die ik nog even wil doen voordat je..

Gesprekleidster 2: En dan richten we ons op, misschien wel belangrijk om te noemen, leefstijl, daarmee bedoelen we in dit geval eten, voeding, beweging, alcohol en roken. Leefstijl omvat natuurlijk veel..

V: Ja het zijn allemaal factoren die..

M: En   01:09:24-4

Gesprekleidster 1: Ontbreken hier factoren? Zijn er factoren die wij niet hebben genoemd nu, die er eigenlijk wel bij zouden moeten? En dan vraag ik dat in eerste instantie aan jou, V? Of denk je dat de belangrijkste dingen wel staan? 01:09:43-9

Gesprekleidster 2: En het op langere termijn volhouden, zijn er nog andere factoren misschien van belang dan het..?

M: Probeer onbezorgd te leven. Je hebt een jaarlijkse controle. Als je auto hebt, ga je 1 keer per jaar voor de APK. Nou, daarna denk je, dan is die goed, tot over een jaar. Als je hetzelfde kunt doen met je onderzoek, je moet wel alert blijven. Maar, je moet eigenlijk denken: ik heb vandaag controle gehad, dan ben ik goedgekeurd. Ik kan er weer een jaar tegen. 01:10:14-4

M: Ik zeg altijd, ik heb er weer een jaar bij gekregen.

M: Nee maar, precies, dat gevoel dus.

V: Ja, maar daar ben ik ook helemaal niet mee bezig. Ik moet gewoon mijn controle doen. Ik had dus gevraagd of het zou kunnen dat we 1 keer in de 2 jaar. En toen vonden ze dat dat niet van toepassing was, maar ik ben er ook niet mee bezig van ik krijg maandag jaarlijks onderzoek en ik sta op en ".." de volgende dag heb ik het gewoon gehad. Laat ik echt niet toe, dat dat mijn leven beheerst. 01:10:47-9

Uitleg Aanbevelingen

Gesprekleidster 1: Laten we even doorgaan naar de aanbevelingen, gezien de tijd. Op basis de inzichten die we uit onderzoeken hebben verkregen. Dat is een fonds, of het wereld kanker onderzoeksfonds, dat doet onderzoek naar de invloed van leefstijl op het ontstaan van kanker. En er zijn op basis van het onderzoek.. Zij houden constant het onderzoek in de gaten, wat daar over verschijnt en dat vatten ze samen. En op basis van die samenvattingen zijn er aanbevelingen voor kanker geformuleerd. Ze staan daar op het bord, ik zal ze even langs gaan.

Gesprekleidster 2: Je krijgt ze zo dadelijk ook in een boekje mee.

Gesprekleidster 1: U krijgt zo meteen een folder mee. Daar staan ze allemaal in. Wat hierbij ook belangrijk is om te melden.. Nou hadden we het daar net ook al over. Het is even goed om erbij te vermelden, van deze factoren is bewezen uit onderzoek dat ze invloed hebben. Dus deze factoren verlagen de kans op kanker. 01:12:03-0

V: En dan mag je je niet schuldig voelen. Als je geen taille hebt en het toch krijgt.

Gesprekleidster 1: En dat is mijn volgende punt inderdaad. Ja precies, dat is precies het volgende wat ik wilde zeggen daarnet. Dat is dus niet de bedoeling. Het is dus niet zo.. U krijgt kanker of u krijgt het niet. De kans die wordt beïnvloed door veel factoren, genetische aanleg, hè, wat u allemaal heeft. Maar er zijn dus ook andere factoren, die leefstijlfactoren zijn niet de enige factoren die daaraan... Wat we weten is dat dat het inderdaad wel beïnvloed. U kunt het een beetje vergelijken met dat u met alcohol op in de auto gaat zitten en een eindje gaat rijden. Hè, als u in de auto gaat zitten en u neemt alcohol, dan is de kans groter dat u een botsing krijgt.

V: maar, dat is toch hartstikke onverantwoordelijk! Dus als ik een patatje eet is dat toch vragen om kanker? 01:13:03-3

Gesprekleidster 2: Misschien is het in het donker rijden een beter voorbeeld. Dat is iets minder heftig.

Gesprekleidster 1: Na ja, de kans is in ieder geval groter dat er iets gebeurd, maar het wil niet zeggen, dat het ook fout gaat. Als u zich aan al deze leefstijlaanbevelingen houdt, wilt dat niet zeggen dat u geen kanker krijgt. Het is ook niet zo als u helemaal niet aan deze aanbevelingen voldoet dat u zeker weten kanker krijgt. Het is geen zwart-wit verhaal. Er zijn heel veel factoren die invloed hebben. En het is individueel

verschillend, er zijn heel veel factoren die er aan bijdragen. Maar goed, ik hoop dat het op deze manier een genuanceerd beeld oplevert hoe dat in de werkelijkheid zit. Hè, het is niet niet zo simpel. En het is zeker ook niet de bedoeling dat u een schuld gevoel krijgt. Op basis van wat u hier ziet. Van nou ik voldoe niet aan die en die aanbeveling.. 01:14:09-7

Gesprekleidster 2: Er zijn maar weinig mensen die er aan die aanbevelingen voldoen.

Gesprekleidster 1: Dat ook, want de factoren die hier staan komen grotendeels, eigenlijk komen die overeen met de algemene leefstijl aanbevelingen, hè? Dus ook ter preventie van hart- en vaatziekten, ter preventie van diabetes. Die komen er allemaal mee overeen, behalve minder rood vlees, geen bewerkt vlees, dat is typisch ter preventie van kanker. Ik loop ze even langs. En overgewicht is geassocieerd met een verhoogd risico. Dus een aanbeveling is: zorg voor een zo laag mogelijk gewicht. Vermijd ondergewicht, natuurlijk dat is ook niet de bedoeling. En een slanke taille. Beweeg iedere dag minimaal 30 minuten, dat is de beweegnorm. Dat geldt ook ter preventie van andere aandoeningen. Dus beweeg genoeg per dag. Eet minder calorierijk voedsel en weinig suikerrijke dranken. Dat heeft ook natuurlijk indirect temaken met overgewicht, verhoogd BMI. Eet voldoende groente, fruit, volkoren producten, peulvruchten en varieer zo veel mogelijk daarbij. De reden dat dat erbij wordt gezet dat u zo veel mogelijk moet variëren, is omdat we eigenlijk niet precies weten aan welk stofje in groente en fruit en peulvruchten het nou precies ligt. Dus als je zoveel mogelijk van verschillende dingen pakt, dan krijg je het stofje in voldoende mate binnen. Dat is de reden dat je moet variëren. Eet minder rood vlees en geen bewerkt vlees. Rood vlees is vlees wat er rood uitziet als het rauw, dus niet vlees wat je maar half doorbakt zeg maar. En bewerkt vlees is waaraan toevoegingen zijn toegevoegd, bijvoorbeeld gerookt vlees. 01:16:02-3

Gesprekleidster 2: Bijna alle vleeswaren..

Gesprekleidster 1: Ja, waar allemaal stoffen aan toe zijn gevoegd om ze langer goed te houden bijvoorbeeld. Drink liever geen alcohol, en als je alcohol drinkt, voor mannen geldt dan twee glazen, maximaal twee glazen per dag en vrouwen geldt maximaal 1 glas per dag.

V: wat een feest. 01:16:22-8

Gelach

Gesprekleidster 1: minder zout eten, wat ook blijkt uit wetenschappelijk onderzoek is dat voedingssupplement niet bijdragen aan het verlagen van het risico. Wat er wordt gezegd: maak of probeer je voedingsstoffen te halen uit het voedsel wat je tot je neemt en niet uit voedingssupplementen en de reden daarvoor is dat niet is aangetoond dat het risico op kanker verlaagd. Dat is niet aangetoond.

V: En wij krijgen nog informatie, staat daar ook iets in over het gebruik van aspirine, daar lees ik heel veel over. En wil ik nou maar weer beginnen maar dat vergeet ik ook weer iedere dag. 01:17:08-9

Gesprekleidster 1: Daar staat volgens mij niks over in.

V: Want dat schijnt iedere dag een aspirine schijnt de vorm of..

Gesprekleidster 2: Daar wordt wel onderzoek naar gedaan, weet ik.

V: Dat weet ik dan toevallig dat dat al heel lang gaande. Dat een lage dosis, ik weet niet wat dat is, 's avonds voor het slapen gaan de kans bij erfelijke darmkanker verlaagd. Ja, dat kan ik wel uit mezelf gaan doen, maar dan denk ik straks krijg ik daar weer oorkanker van. 01:17:43-0

Gesprekleidster 2: Dat is iets wat u met uw arts zou moeten bespreken.

V: Ik heb geen arts.

Gesprekleidster 2: Er komt ergens een controle neem ik aan. Wel toch?

V: Nou, niet voor mijn darm. Ja, dan krijg ik van die zusters en die gaan met de slang in en dan ga ik weer naar huis. Ik zie nooit iets van rang of stand. 01:17:57-3

M: Maar daar zit een arts bij. Met een darmonderzoek.

V: Ja, maar ik weet niet wie dat is. En ik heb ook niet het gevoel dat daar tijd voor is om het te vragen. 01:18:08-2

V: Dan zou je kunnen vragen om een afspraak te maken.

Gesprekleidster 1: Dat wilde ik net gaan zeggen, misschien is dat wel een goed idee. Om op zoek te gaan naar degene, want u zat toch ook bij dokter **. 01:18:24-6

V: Dan moet je het nu nog doen want Dr. **gaat met pensioen, die wordt 65.

V: Iedereen die ik krijg gaat op vakantie, of met pensioen of die gaat dood. 01:18:34-9

V: Ik moet echt weg.

rumoerig

Gesprekleidster 1: Mochten er nog vragen komen laat het dan weten.

V: Voor een aspirine ga ik dokter ** niet lastig vallen. Nu leeft het heel erg, maar als ik thuis kom dan, um, ik heb een normale leefstijl en ja, soms twee wijntjes per dag toch. 01:19:38-2

V: gewoon doen hoor 01:19:37-9

Gesprekleidster 1: Nou, heel erg bedankt, voor het meedoen. 01:19:50-6

Mevrouw verlaat ruimte

M: Die voedingssupplementen dat, die noemen ze dus in wezen vooraf. Ik heb nu het idee omdat ik geen dikke darm heb dat je opname slechter is. Ik gebruik elke dag die multivitamine en ik ben nooit ziek. En in mijn omgeving zijn heleboel mensen die nemen geen multivitamine en die zijn altijd ziek. Dus of ik nou..

Gesprekleidster 1: Nou, het wil ook niet zeggen dat het helemaal niets doet hoor. Daar staat dat voedingssupplementen niet bijdragen aan het verlagen van het risico van kanker, maar het wil niet zeggen dat het geen andere effecten kan hebben. 01:20:28-1

Gesprekleidster 2: En er zijn uitzonderingen, ook dat als u, binnen een bepaalde situatie heeft het wel toegevoegde waarde. 01:20:38-1

V: Maar als je een kortere dikke darm heb, zo zit dat in mijn hoofd dan, dat de opname van die voedingsstoffen uit je lichaam minder goed is. 01:20:51-9

V: Ze verbazen zich ook dat ik zo groei. Dat is echt waar want ik ben zo geweest, en door de jaren heen word ik steeds zwaarder. En het gaat binnen korte tijd uit, hoe ik dat doe dat weet ik echt niet. Maar ja, ik ben aan het groeien.

V: Maar ik moet echt lijnen ".."

V: Ja, maar ik denk ook dat dat te maken heeft met verkeerde genen. De ene kant van de familie is behoorlijk zwaar. Dus ik denk dat dat ook meespeelt. 01:21:23-1

V: Maar ik begreep dat dat ook een reden kon zijn, om bijvoorbeeld Magnesium of Selium te nemen. 01:21:29-8

V: Maar ik ben nooit ziek, ik eet naar mijn idee gewoon goed. Dan denk je, ja goed, ik heb een lijf waar iets mis mee is. Maar ik heb gewoon een normaal leefpatroon. En dan denk ik, waarom heb ik al die spullen nodig? Die zitten toch allemaal in mijn eten. 01:21:43-9

Veranderen leefstijl

Gesprekleidster 1: Als je nou zo naar die aanbevelingen kijkt, hè, zijn er dan aspecten, daar voldoe ik niet aan? En daar zou ik iets aan willen veranderen? 01:21:53-5

M: Nou, ik zie er wel eentje en daar ben ik wel heel benieuwd naar wat u daarvan vindt. Ik heb, zoals ik al verteld heb, ik heb veel moeilijkheden kan wel zeggen, problemen, maar laat ik het maar moeilijkheden noemen. Om dus voldoende energie binnen te krijgen hè, dan denk, ik doe nog een beetje suiker in de koffie. Ik lust eigenlijk geen koffie zonder suiker, thee zonder suiker, ik denk maar ja, ach, het is ook goed voor mijn energievoorziening. Want dan suiker zit toch energie in, dus denk van dat komt helemaal goed. Dat moeten ik laten prevaleren. 01:22:34-0

Gesprekleidster 1: Dat is een heel goed punt inderdaad, maar suiker is niet het enige waar je energie uit kunt halen. Je kunt ook energie uit fruit halen bijvoorbeeld. 01:22:42-1

Dat is gezonder om daar je suikers uit te halen dan uit gewoon suiker. Zo zijn er, hè, voor iedereen is het natuurlijk individueel verschillend, hè. U zegt inderdaad, dit zijn algemene aanbevelingen en die komen niet altijd 1 op 1 overeen met de individuele situatie. Want er zijn misschien aanbevelingen die daar staan, die er staan dat u denkt nou dat werkt voor mij helemaal niet zo. Ik heb juist te kort aan energie en ik moet extra energie hebben en ik mag geen energierijke of suikerrijke dranken. Maar ja, dat doet me nou net zo goed. Dat kan ik me prima voorstellen, want het is ook, daarom zijn het ook algemene leefstijlaanbevelingen. Maar er zijn, als je er over nadenkt, zijn er ook wel alternatieven. Er valt ook wel een middenweg te vinden daarin. Net als wat ik zei hè, er kan in plaats van suiker in de koffie, suiker, voor energie uit andere voedingsmiddelen halen. 01:23:45-5

M: Maar volgens mij zou je alle deuren openzetten om energie binnen te krijgen. Als dat lukt. Dus ik denk van, er zal door wat dan ook, moet ik energie.. Ah, ten minste, beetje de levensstijl wil handhaven die voor de operatie had.

Gesprekleidster 1: Dat zou nou typisch een goede vraag zijn voor een diëtist. Hoe kan ik welke producten innemen waarvan ik snel energie van op kan nemen. En om te zorgen, geen dik makende producten, maar wel producten die mij de dag door laten komen. Dat zijn nou typische vragen die daarbij terecht.., die zou u echt advies kunnen geven.

M: En toch heb ik daar geen antwoord op gekregen toen ik mijn dikke darm kwijtraakte, want daar zat ik dus ook mee. Dan ben je heel veel afgevallen op dat moment en je hebt een slechte conditie. En je wilt eigenlijk advies hebben van de diëtiste van wat moet ik nu doen om dat daar goed mee te kunnen leven. En daar zitten ze dan toch eigenlijk niet in, ze zijn heel erg, um 01:25:04-2

M: over lijnen kun je 1001 advies krijgen, maar om kilo's erbij te krijgen zeggen ze van, uh ja, um 01:25:14-3

M: Dan ga je zoeken en kijken bij dingen, adviezen voor stoma patiënten, dan kom je dingen tegen waar je meer aan hebt dan, um ja, misschien, ik wil een diëtist dan ook niets verwijten hoor. Maar je hebt een, ze zijn gefocust op diabetes patiënten. 01:25:41-8

Gesprekleidster 1: Geen specifieke oncologie diëtisten. Het was een algemene diëtist..

M: Ja, een algemene diëtist, die zit in streekziekenhuis en al die ontwikkelingen gaan steeds verder, dat is hetzelfde als informatievoorzieningen die gaan in de tijd, vroeger was hier geen klinisch genetisch centrum. Nu is dat er wel, dus nu zijn er een heleboel dingen omheen gebouwd, hetzelfde als je in het ziekenhuis in

komt met borstkanker, dan heb je tegenwoordig vaak zo'n mammapoli, waar dus alles omheen is gebouwd. Voor dit soort dingen kom je overal, incidenteel.. Er zijn maar een paar mensen die met dit soort dingen in een ziekenhuis komen per jaar. Dus er zijn er niet veel, zeker in een streekziekenhuis. Dus ik wil die mensen niets verwijten en gaande weg vind je je weg wel.

Gesprekleidster 1: Even gezien de tijd, het is tien voor twaalf, dus laten even naar de afronding toegaan. Ik zou nog graag willen weten als u naar die aanbeveling kijkt is er nog iets wat u zou willen veranderen. En waarom zou u dat willen veranderen. Zou u daarop kort proberen antwoord op te geven? 01:27:00-8

V: Het gewicht naar beneden, dan heb je gewoon, um, dan beweeg je heel slordig, denk ik altijd. Dus het is, je bent toch iets fitter, ik ben wel afgevallen, dan ben je toch gewoon iets fitter. En verder, ik beweeg niet elke dag, maar beweeg voldoende. En eten dat heb ik al gezegd. 01:27:22-3

V: En als je erg beroerd bent dat is niet altijd te verklaren. Weet ik veel, 's ochtends heb ik dat eens, voor de kinderen jus d'orange. Dan wil ik daar, dat drink ik dan in korte tijd op, dan ".." 01:27:34-7 Dat is zo raar. En dan denk ik, dan willen ze graag een koekje, dan hoef ik niet zo nodig suiker spullen, ik drink bijvoorbeeld nooit fris ik denk of water. Dat vind ik gewoon niet lekker, dan kom je dus, daar verbaas ik me, waarom ben ik dan over de grens gegaan? En dat eventjes net andersom doen, suiker eventjes gebruiken.

M: Moet je eens een Magnum eten 01:28:02-4

V: en dextro

V: Diabetici doen het met dextro, hè?

Gesprekleidster 1: En V2, hoe is dat voor jou? Zou jij iets willen veranderen wat daar staat nu of zou je niets willen veranderen? 01:28:19-5

V2: Nou, misschien de laatste 14 dagen heb ik wel wat wijntjes op. Want we waren in een streek waar mijn man een huisje heeft, waar we druiven geplukt hebben met wijn. Ben ik maar beetje ".." gegaan, maar nu gaat dat gewoon weer terug. 01:28:30-1

Gesprekleidster 1: Dat is meer ook een moment, zeg maar, waardoor het omhoog gaat.

V2: En we zijn ook niet op een leeftijd, dat je ieder moment feestjes heb waar je helemaal op achteruit gaat. Dan zou ik dat ook niet meer doen. 01:28:46-6

Gesprekleidster 1: En M2?

M2: Ja, ik moet punt 1, want ik heb hetzelfde probleem. Ik krijg het bijna niet af, ondanks dat je dus sneller stofwisseling hebt. En als je afgevallen bent dan hoef je ook maar even te bezondigen en het is er weer bij. En ik moet punt 5. 01:29:04-2

Gesprekleidster 1: minder rood vlees

M2: Ja, minder rood vlees, dat is allemaal wel terug naar 100 gram. Daar houd ik die 100 gram wel echt wel aan. En bewerkt vlees, dat heb ik me niet gerealiseerd. 01:29:15-1

V: Dat zeiden jullie straks ook hè, met name ook dan vleeswaren. Want dat wordt allemaal bewerkt natuurlijk. 01:29:23-3

V: Maar bijvoorbeeld gerookt vlees, ik gebruik paarden rookvlees. Dat valt er ook onder, maar ja, kaas is ook zo'n verhaal. 01:29:30-6

Gesprekleidster 2: Alleen die staan niet in het rijtje, omdat daar dan niet voldoende bewijs is dat dat zo is.

V: Maar ik eet dan boterham met zoet, maar dat mag ook niet hè. Dus ik denk ik ga over op paarden rookvlees dat is dat heeft smaak, dan is dat goed om te eten. 01:29:41-3

Gesprekleidster 1: En wat is de reden dat u dat voor u, hè, wat is uw motivatie om dat te willen veranderen? 01:30:01-6

M: Omdat je bewust er van bent, ik heb vleeswaren dus niet in de categorie gezet van dat het en ja kijk, dat afvallen en slankheid dat weet ik wel. En dat is ook wel, dat moet ook wel, maar goed, dat is een probleem op zich.

Gesprekleidster 1: Maar dat is meer voor u algehele gezondheid wilt u zeggen?

M: Nee, maar op een gegeven moment als je niet oppast, kom je elk jaar een kilo of twee kilo bij en als je dan 20 jaar verder gaat. Dan krijg je heleboel andere problemen. Dus dat is gewoo,n voor de rest gaat wel. En dat calorierijk dat is toch een probleem, omdat je kunt overvallen worden door energiegebrek. Je kunt als een deken, dan is het echt op. Voor het eten op, dan heb ik echt warm eten op, dan heb ik bij gegeten, en dan ben ik weer vol. Dan moet je misschien vaker eten, of je moet zorgen dat je dingen bij je hebt, maar dan is het dus wel calorierijk of suikerrijk, want suikerklontjes die werken dan ook. 01:31:26-8

V: Ik heb wel koekjes bij, crackertje bij. En dat voel ik dan, denk ik, oh jee. Nou, en dan drink ik weer en ik drink niet zo vaak. Drinken, drinken, drinken.. 01:31:35-7

Gesprekleidster 1: En M1, wat, zijn er voor jou dingen die er bij staan of denk je van nou ik wil verder geen aanpassingen maken? 01:31:46-5

M1: Nou, als ik ooit nog iets aan zal passen, ik denk niet dat het gebeurd, maar dan eerste wat in het rijtje opvalt, is dat ik minder rood vlees zou eten. Maar dat is al een stuk minder, want sinds mijn operatie is vlees eten eigenlijk wel een ramp, maar ja, ik.. Vroeger kon, ik, men mij eigenlijk geen vlees genoeg geven. Moest ik eerst al het vlees op eten en dan moet ik nog een aardappel zal ik maar zeggen. Maar vroeger was dat toch wel, maar dat is allang niet meer zo, want tegenwoordig met die operatie, wat ik in 2005 gehad, is het vlees eten wel een beetje een ramp. Het wil gewoon niet meer weg en dat is maar, ja bewerk vlees wel, dat doe ik, dus dat zou ik misschien er aan kunnen passen. Maar ik weet alleen nog niet, ik heb altijd zoiets zo van mensen die het gen hebben en die de dikke darm hebben die nog nooit iets of wat, dan denk ik van, ja, dan kan ik me iets bij voorstellen dat bij dat soort mensen van belang is. Maar ik ben mijn dikke darm kwijt en daar hoef ik het in ieder geval niet meer voor te doen.

V: Dat kun je ook niet meer terug krijgen. 01:33:14-9

M: Nee, mijn dikke darm kan ik ook niet meer krijgen

V: in welke vorm dan ook

V: Het is natuurlijk wel zo, het is geen garantie. Dikke darm weg, dat er niet meer ergens iets zweeft, hè? 01:33:30-1

V: Ik kijk jou aan. Als dat een garantie is, dan vind ik, dan hoef ik me niet elk jaar te controleren. Dan heeft dat niet zo heel veel functie natuurlijk. Zo werkt het. Ik heb ook nog een lever en daar zitten vlekjes op, dan zijn dat toch dingen die zij heel graag terug willen zien. Dat is dus niet de garantie.

M: Ik wil me ook niet aan controles of dergelijke ontrekken, dus dat doe ik keurig elk jaar, maar ik heb dus, maar goed, je had het erover om iets te kunnen veranderen. Dan denk ik van, dan kan dat wel iets minder kunnen, maar ja, dat suiker dat zit niet heel, op 1 of andere manier zou ik toch mijn energie moeten komen. En dat geldt voor mij heel erg, anders houd ik het niet bij met mijn energievoorziening zal ik maar zeggen. 01:34:22-8

Gesprekleidster 1: Ja precies, en als u nou bedenkt wat u zou willen veranderen, wat zou nou helpen voor u,

om uw leefstijl aan te passen? Zijn er dingen die bij zou kunne.. *alarm gaat* Het is bijna tijd. 01:34:44-3

V: Ik zou het nu niet meer zo goed weten. De meeste dingen daar vind ik mij wel in daar en probeer ik mij aan te houden, met uitschieters met vakantie of iets aparts. Maar niet iets bij wat ik nu zeg, maar dat komt natuurlijk ook door het familiaire en

M: En toch ik heb al aangegeven dat onbezorgd leven, dat is ook in dit soort dingen. Je kunt wel heel gestrest met je voeding bezig zijn. Ze zeggen wel eens, het Moorman dieet, wil je zus niet aanvallen hoor. Je leeft niet langer, het lijkt langer. 01:35:41-0

Gelach

M: Nee, omdat je er bewust mee bezig bent. En dat is het, maar het moet ook niet omslaan naar de andere kant, van ik mag niets meer. Ik mag niet drinken, mag geen vlees, ik mag dit niet, ik mag dat niet. Dan leef je misschien wel gezonder, maar dan krijg je ook zoveel stress dat dat het probleem wordt.

Gesprekleidster 2: Ik schrijf hem er even bij. 01:36:11-7

V: Laat ik nou even zeggen van zout, want ik was die zoutpot die vliegt bij ons over het eten, die komt niet over het eten. Maar mij werd geadviseerd om meer zout te eten, omdat ik echt vocht vast houdt, vocht zou vast houden. En dat merk ik dus ook, dat je denkt van dat doe je als dat goed voor je is. Ik ben het in de loop van der jaren ook weer gaan minderen, want ik merk dat ik en dikke enkels krijgt omdat je ouder wordt krijg je weer andere kwaaltjes ook gewoon. Dus zoutpot komt bij ons weer aan het *zwiegen*. 01:36:42-1

Maar hier staat dan weer minder zout, maar daar werd gezegd: doe maar wel wat zout in je eten want je verliest heel veel vocht. 01:36:52-9

Drie belangrijkste factoren

Gesprekleidster 1: We gaan even ter afsluiting terug naar het lijstje met factoren, zouden we misschien kunnen kijken welke factoren nou voor u zouden bijdragen een verbetering van uw leefstijl, volhoudt. Er zijn ook wel wat factoren genoemd en ik ben benieuwd wat nou voor u de drie belangrijkste factoren zijn die u leefstijl beïnvloeden. Misschien dat we daar per persoon antwoord op zou kunnen geven ter afsluiting en dan maken we ter afsluiting een rondje dat iedereen even wat kan zeggen en dan ronden wij het af wat mij betreft. Dus vraag 1, zijn er specifieke factoren die voor u beïnvloeden die u dat u een verbetering in leefstijl kunt volhouden en 2 is: wat zijn de drie belangrijkste factoren die u leefstijl beïnvloeden? Zou je misschien willen beginnen.

M: De belangrijkste is toch het rust nemen en het ja, de gewoonte zeg maar, ja dat versta ik onder regelmatig leven, zeg maar. En.. 01:38:16-0

Gesprekleidster 1: Is dat ook wat volhouden beïnvloed, regelmatig leven?

M:Ja, maar het hangt met elkaar samen en de gezondheid is eigenlijk, maar die drie dingen staan. En de 1 staat bovenaan ook 01:38:29-0

Gesprekleidster 2: Ja maar, ik schrijf hem ook even bij volhouden. Dus eigenlijk mag ik gezondheid ook.. 01:38:44-5

Gesprekleidster 1: V2, wat? 01:38:43-7

V2: Nou, daar sluit ik me bij aan. Bij gezondheid en um, even kijken, voorbeeld uit de omgeving is voor heel sterk en de informatie als prikkel. Want ik lees ook heel vaak foldertjes van kanker en dan krijg je ook wat je allemaal via de media tot je krijgt. Voor mij is ook de gezondheid en goed, het ritme van leven goed zicht in krijgen. Merk ik als je andere dingen doet. En informatieprikkel dat dat toch inderdaad, dat je iedere keer weer naar jezelf kijkt.

Gesprekleidster 1: En zijn er nog specifieke factoren die we nog niet hebben genoemd bij het volhouden van de leefstijl die? 01:39:46-0

V: Niet echt 01:39:46-0

Gesprekleidster 2: of die het juist lastig maken

V: Voor mij, ik weet dat ik het heb, maar het moet niet mijn leven beheersen. Als iemand iets aan de hart heeft aan de longen heeft, moet er ook gewoon mee leven. Dat vind ik van dit ook. 01:40:06-9

M: Maar die informatie als prikkel, dat zit ook bij het volhouden, maar ik wil ook weer niet alles meer weten, op een gegeven moment is dat ook.. 01:40:16-5

V: Daar zit een grens aan ja 01:40:16-5

M: Ja, daar zit een grens aan

V: Ja, ja, maar ik ben daar niet zo mee bezig, dus ik vind die prikkel wel lekker. Eens even goed lezen, prima, en je doet er iets mee, of je slaat het op en je kan nog een keer terug kijken. Dus dat is het eigenlijk. 01:40:29-5

Gesprekleidster 1: M1, wat zijn voor jou 3 belangrijke factoren?

M1; Nou, de energie natuurlijk hè, ik heb mijn dagelijks met energie te maken, om genoeg energie binnen te krijgen. Dat is mijn dagelijkse zorg tussen haakjes. Maar ik moet gewoon geregeld eten, dus geregeld zorgen dat ik daar aandacht aan besteed.

Gesprekleidster 1: Regelmatig leven 01:41:03-3

M1: Ja, en dan toch ook de stoelgang beïnvloed toch mijn gaan en staan als ik gewoon goed gegeten heb kan ik niet zeggen dan ga ik een mooi wandelingetje maken ofzo. Dan moet ik eerst wel zorgen dat ik eerst naar het toilet geweest ben. En dan kan ik wel weer, dat bepaald toch, je houdt er wel rekening mee. 01:41:32-7

V: Het is wel mee om te gaan.

M: Ja, je kunt er wel mee omgaan, maar je moet er wel..

V: Je denkt er wel altijd aan. Ik ervaar het niet meer bewust als belastend. Ik ga ook naar de bossen en daar kan ik overal zitten. snap je, dat is gewoon een stukje, maar ik loop ook niet door het dorp. Dan wordt het een beetje moeilijk. 01:41:43-5 01:41:56-4

Afronding

Gesprekleidster 1: Oke, laten we gaan afsluiten met het laatste rondje en zou u daarbij ook willen noemen, u mag vragen stellen, opmerkingen maken, vertellen of iets melden, of er iets nog niet aan bod is gekomen wat u nog belangrijk vindt. Afsluitende opmerkingen of vragen.. 01:42:18-2

M: Ik heb geen vragen meer. 01:42:21-9

V: Nou, had ik daar straks over broodbeleg. Dan kom ik weer een beetje op de voeding weer terug. Het was ik heb ook wel eens een periode banaan of kiwi op brood. En dat smaakt best, banaan beetje combineren met een beetje honing of rozijntjes.

V: nee, nee, dat smaakt, maar goed, dan zit je in een bepaald patroon.

Gesprekleidster 2: even wat creatiever. 01:42:48-6

V: ja, dat is, vaak is dat wel zo, dat je binnen een bepaald tijdschrift van Moerman daar geven ze vaak recepten en tips. Wat kan je dan variëren? 01:43:02-5

Gesprekleidster 1: Krijgt u zo meteen ook mee. Een receptenboekje zit ook daarbij.

Gelach

V: Ik heb verder geen..

Gesprekleidster 1: M1? 01:43:11-0

M1: Nou, ik had eigenlijk over dit niet zoveel meer over te zeggen. Ik had dadelijk ook gehoopt hoe het dan zeg maar is met een onderzoek ofzo of dat soort dingen. Dan had ik een opmerking van, laat mensen zoals ik als je 60 km moet rijden voordat je hier bent. En je krijgt dat onderzoek en als je dan de voorschriften leest ook 's morgens nog voordat je naar het ziekenhuis moet een liter moet drinken. Dan zal ik niet weten als ik zeg maar de laatste keer een opmerking had gemaakt. Ik zeg jongens, als jullie me 's morgens nog en ik moet om 9 uur hier zijn en moet ik ook 's morgens voor die tijd een liter drinken en ik moet dan ook nog anderhalf uur zoals vanmorgen in de auto moet zitten. Dan weet ik niet wanneer ik die liter moet drinken. Rooster die mensen in ieder geval na de middag in.   01:44:17-6

M: Maar het probleem van dan de middag inroosteren, was weer dat je dan, die, zo lang aan het laxeren bent en dat je weer dan zit je dan weer met energie tekort. 01:44:28-7

V: Het kost je, ik denk dan maandag, het kost je dan toch meteen je dag. Zondag houd ik al rekening met mijn eten. Maandag ga ik spoelen. Dan probeer ik nog wel een beetje te werken voor afleiding. En ik heb het al die jaren in de middag gehad. Ik kom thuis ik eet wat en ik ga naar bed, morgen ben ik er weer. Je bent toch niet lekker. Want wat u doet, u moet heel vroeg beginnen, je bent gewoon niet lekker, hè? Want al je vocht gaat eruit. En dan voor de rest van de dag, ik moet zeggen vind het wel heel prettig en ik merk dat ik niet schoon ben in mijn auto want je hebt gewoon die wc elke keer nodig. En ik ga daar goed aan het drinken ik heb het toilet bij en dan is het weer voldoende. 01:45:19-7

M: Maar u laxeert met clean prep, maar begint u ook de avond van te voren?

V: Nee dat gebruik ik allemaal niet.

M: Nee, want ik doe 1 liter ‘s avonds en 1 liter ‘s ochtends.

V: Ik doe met eten rekening houden, dus ik eet al niet meer warm en ik begin ‘s morgens. Ik kan gewoon heel goed drinken. 01:45:48-3

M: Als ik na de middag kan, dan begin ik ‘s morgen pas om 6 uur, ik drink een halve liter tot drie kwart liter en ik ben hartstikke schoon. Met mij gaat het natuurlijk als een speer door natuurlijk. 01:45:58-5

Je krijgt een, zeg maar, de voorschriften mee wat ieder normaal mens heeft. Maar, als je dan in de lengte doorloopt, tussen haakjes, zo'n beetje. Dan denk ik van, twee liter drinken, ik zou niet weten hoe ik dat op moet krijgen.

Gesprekleidster 1: Precies, en zo zie je maar weer 01:46:25-3

M: Eigenlijk, dat soort voorschriften vind ik dan

Gesprekleidster 2: Individueel

V: Het wordt ook altijd gevraagd, ik vind dat zo mooi, van ze zijn er helemaal niet mee bezig. Ze schrijven gewoon een recept voor.   01:46:40-0

Ik denk dat mensen die geen dikke darm hebben niet dat flesje nodig hebben. Als je goed je klep gebruikt en je doet goed het water dan ben je zo doorgespoeld. Ze hoeven ook maar een klein stukje na te kijken. Wordt nooit over gesproken. Dan denk ik van al die medicijnen ik zeg bij de apotheek die hoef ik niet, die hoef ik niet.

Gesprekleidster 1: Dan zou je toch denken dat het beter afgestemd zou zijn. 01:47:05-8

V: Dat is toch raar.

M: Nee, maar we zijn nu met z'n drieën en we doen het alle drie op een andere manier.

V: Dat ligt ook aan de tijd.

M: Doe er dan gewoon een briefje bij, zorg dat u schoon bent en ik zal zeggen en vul zelf in hoe je dat wilt doen. Maar het wordt bijna dwingend als je het zo leest. 01:47:21-7

M: Maar ze doen eigenlijk voor zo’ n signoide scopie alleen voor het laatste stukje, is standaard een klysma. maar als je een klysma krijgt, ben je wel heel laat klaar. Want je hebt geen klep, vroeger zat er een klep tussen de dunne darm en de dikke darm, maar dat is dus weg. Als je nu een klysma krijgt blijft die dunne darm maar doorlopen, dat is dus veel erger. Iedereen vult zijn eigen manier in, ook door de tijd, maar eigenlijk wordt dat niet opgeslagen in een systeem. 01:48:10-6

M: Ik heb nu dus aangegeven, ik wil in het vervolg na de middag een onderzoek.

V: Ja, het is natuurlijk standaard de informatie die je krijgt, sprak gister iemand die maandag dit voor de eerste keer ging doen, die heeft wel alle informatie als je dat de eerste keer of 1 van de eerste keren gaat doen.

Gesprekleidster 1: Nou laten we, we hebben wat foldertjes voor u.

Focusgroep 3 Nijmegen 141008

Context

Gesprekleidster 1: ..u zelf even wilt vertellen en hoe lang u al weet dat u drager bent van het Lynch syndroom en relevante dingen die daarmee te maken hebben. Bent u geopereerd? Wat voor u van belang is. Zou ik aan jou mogen vragen? Daar ging ik al bijna, haha. <u>00:00:33-1</u>

V: Zou ik aan u mogen vragen? haha

Gesprekleidster 1: Ja

V: Um, ik vermoed dat ik, het Lynch syndroom weet ik sinds begin jaren '90 en de bevestiging is gekomen in '99. Omdat ik toen ziek was één van de weinige die een andere vorm kreeg van kanker. Heb inmiddels viermaal kanker gehad. En um, *stilte*, wat had u verder nog gevraagd?

Gesprekleidster 1: Wat voor type kanker heeft u gehad? <u>00:01:02-2</u>

V: Eierstokkanker en um, dunne darm hoger in de buik en toen in de dikke darm, maar de eerste kanker, de tweede en de derde tumoren waren gerelateerd aan de eerste tumor. En de eerste tumor bij het wegsnijden van de tumor is gaan lekken, heb daar ".." spoeling enzo voor gehad. Maar de volgende twee tumoren ".." eerst waren er uitzaaiingen van.. <u>00:01:42-7</u>

V: En hoe lang is dat geleden dat u voor de laatste keer ".."?

V: vijf jaar geleden.

Gesprekleidster 1: En hoe gaat het nu met u? <u>00:01:55-4</u>

V: Um, het is te doen maar ".." of wel voeding of wel vocht. Dat ".." vasthouden ".." En verder door de intensieve behandeling die ik in ‘98 heb gehad. Mijn hele buik is dus bestraald. Dat geeft problemen.

Gesprekleidster 1: En wat voor problemen geeft dat dan? Weet u dat nog? <u>00:02:24-1</u>

V: Onder andere bij de laatste operatie. Heel ".." niet meer.

Gesprekleidster 1: V1?

V1: Ik weet sinds 2006 dat ik het Lynch syndroom heb. Ik heb in 2005 geopereerd aan baarmoeder kanker toen was ook binnen onze familie. Ik was de eerste. En toen kwam heel snel daarna het bericht dat een tante van mij ook baarmoederhals kanker had. En nu is eigenlijk alles in een stroomversnelling gekomen. Ja, voor mij was het eigenlijk 1 en 1 is 2. Dus dat ik uiteindelijk dat syndroom had nou dat kwam voor mij op dat moment niet binnen als verassing. Ja, daar is het bij mij gelukkig bij gebleven.

Gesprekleidster 1: En V2?

V2: Ik weet 25 jaar zeg maar DNA onderzoek dus familiair. Mijn moederskant zijn heel veel allemaal overleden. Met 30, 35 en een neef van mij daar begint het bij mij ook al. Ze zijn ook heel vroeg er mee bekend. We hebben ook een jaarlijks onderzoek in het begin was het 1 keer in de vijf jaar. En nu is dat gewoon jaarlijks. Er wordt elk jaar wel wat gevonden hoor. En dan vooral in de darmen en hele ritueel hebben we dan eierstokken, baarmoeder, de nier, de blaas, de dunne darm weet ik het allemaal. <u>00:04:00-4</u>

Gesprekleidster 1: En heeft u kanker gehad ook zelf? Of niet?

V: Ik heb wel poliepen gehad maar verder niet.

Gesprekleidster 1: Nee, mooi. En M1? 00:04:13-8

M1: Ja, ik weet het sinds 2002. Mijn nichtje was, maandelijks menstruatie was heel heftig. Dus dat vonden ze allemaal, hadden ze onderzoek gedaan. En bleek dat dat een afwijking was. En vandaar uit is het balletje gaan rollen. Ja, vorig jaar zie je gewoon de familie 1 voor 1 wegvallen. En dan ga je zelf over leeftijd dat je dan ook, zeker verzekeringstechnisch, hypotheek etc. eens wat gaat doen, je kunt je kop in het zand steken, maar je weet gewoon het is gevaarlijk. Dus dat heb ik dan ook maar wel gedaan. En um bij mijn zus ook, ik heb vier zussen nog. Twee andere zussen hebben het ook. Die hebben inmiddels ook kanker gehad. En bij mij ik dacht nou als ik jaarlijks onderzoek doe, is er uitgekomen dat ik ook wel positief was en ja, als ik jaarlijks onderzoek doe, ben ik er op tijd bij. En maar, ik wist ook helemaal niet dat een gespecialiseerd ziekenhuis was et cetera. En naar Maastricht is voor mij een beetje ver weg. Ik denk, ik zoek het in de buurt in de buurt. Daar is waarschijnlijk niet ver genoeg gekeken in de dunne darm. En de keer daarop hebben ze wel iets gezien. En toen was het van kom maar snel terug. Toen zeiden we van ja, het is wazig we moeten niet wachten. En in die tijd waren mijn vader en moeder al overleden, mijn tante, mijn oom et cetera. Dus dan ben je zelf aan de beurt, denk je, wil ik dat wel straks wachten. Toen zei mijn zus, doe toch maar niet. Dus ik heb dat um zitten zoeken zeg maar, waar kan ik het beste behandeld worden. Via de vereniging HNPCC, ja, ik moet hier in het *ZKH5* zijn. En vervolgens een brief opgesteld en kom met urgentie hiernaar toe. Gewoon gelijk door elkaar van ja, platliggen en we gaan kijken of we het kunnen weg halen. Want de gegevens zoals in *** was het te doen. Kwamen een keer bij ons thuis, denk wel zeker 5 centimeter zijn op de plek van mijn dunne darm. En dat betekent als je jong bent zoals.. van maak gewoon dat het subtotaal colectomie 00:06:33-4 en dat is schrikken. Wat is het kwaliteit van leven, kom ik eruit, wil ik dat wel? Zoveel vragen en zo weinig tijd. En dan ligt het eraan, zo erin gegaan ben en je bent je hele dikke darm kwijt. En dan is het opkrabben hoe het werkt met alles erom heen. En dan kwam je eigenlijk van kwaliteit van leven en hoe ga je er mee om, wat kun je verdragen en wat kun je niet verdragen? Dat is een hele lange weg en tot nu toe zelfs nog. En ja, ik merk gewoon je hebt gewoon energie ingeleverd ook ander werk zoeken, ga ik dat wel of niet vertellen. Dat is hard. En dat maakt het niet makkelijker op. En ik heb iets van je moet toch verder, want de zon schijnt hè, het regent, het gaat gewoon verder, je moet zelf ook verder voor je gezin, maar je zit er zelf wel mee. Het is wel een stukje wat je mee draagt Dat kun je niet wegstoppen. En elke keer dat onderzoek wat we jaarlijks hebben dat is een hele belasting natuurlijk voor het herstellen. En toch dat ene stukje toch, dat er weer een poliep zit die niet goed is. En dan, ja, je moet het toch blijven doen, ondanks dat stukje. Je ziet, je bent niet de enigste, we hebben allemaal ons eigen verhaal daarin. Het is wel belangrijk wat je zelf mee leert omgaan en waar je het gaat plaatsen. Je kunt het aan heel veel mensen vertellen maar het zit wel in je. En je voelt je nooit een dag hetzelfde ".." Er kan altijd een goede dag zijn en wat minder. Ik werk niet meer volledig, ik heb een parttime baan. Zodat ik die rust krijg, want anders ik merk gewoon dat ik energie ingeleverd heb. Ja, je mist een orgaan dus dat zal iets doen 00:08:23-8 wat ik voor kon eerder dat kan ik nou allemaal niet. 00:08:32-4

Gesprekleidster 1: En wat is er bijvoorbeeld wat u nu niet goed kan verdragen wat u eerst wel kon eten?

M: Ja, zoals kerriesoep, daar moet ik niet aan beginnen. En specerijen..

Gesprekleidster 1: Gekruid eten

M: Terwijl ik het wel lekker vind, maar dat moet ik niet doen. En je merkt gewoon van nou, um, dat gaat gewoon verkeerd. Dan speel je gewoon op safe. Ja, ik begin te barbecueën, maar dat zien ze niet aan de buitenkant. Je weet pas dat een impact heeft als ik meer op de wc zit, dan, ja, dat is niet prettig

Gesprekleidster 1: Ja 00:09:12-1

M: dus ja dat is mijn verhaal, haha

Gesprekleidster 1: Nou, wil jij iets vertellen?

V: Ik weet niet precies hoe lang ik het weet moet ik heel eerlijk zeggen. Ik doe al jaren aan onderzoek mee, ik schat een jaar of tien denk ik. Het komt van mijn vaderskant, is daar in '81 aan overleden. En via nichten,

ooms is dat onderzoek gestart. Ik heb vier zussen en die hebben het allemaal. Dus het is 100% ‘…’. Ik heb preventief mijn eierstokken en baarmoeder weg laten halen. Verder doe ik iedere twee jaar een onderzoek, daar is nooit iets gevonden, dus daar ben ik heel blij mee. En ik heb mijn zoon ondanks getest en die heeft het ook niet. 00:09:55-7

Gesprekleidster 1: Oh dat is fijn. Geruststelling.

V: Ja, dat is wel heel lekker. Maar ik heb dus, ja, nog niets mee gemaakt in dat opzicht.

Gesprekleidster 1: En jij?

M: Ik heb van mijn vader gekregen. Het is aan het licht gekomen dat hij darmkanker had in combinatie met de nier en hij is in ** geopereerd, daar is de link niet gelegd. En twee jaar later is mijn moeder hier in het ziekenhuis gekomen voor borstkanker en nou ja, goed, die oude mensen vertellen gewoon van alles. En toen kwam aan het licht van een combinatie, het kan erfelijk zijn mogen we dat onderzoeken. Ja, dat is onderzocht en inderdaad erfelijk gen. Mijn zus en ik zijn ook allebei drager. Ik weet het nou een jaar of tien um ik hoor wel eens "poliepen gehad..". Um en verder heb ik daar niets mee en nieren. Ik heb 1 heel erg "woekerige" nier, die houd ik wel extra in de gaten. Omdat mijn vader ook nierkanker gehad heeft. Hij verandert niet veel, maar dat die niet helemaal goed is zijn ze ook niet zeker. Maar goed, ze houden plaatje tegen elkaar.

Gesprekleidster 1: Zal het wel goed zijn, ja.

M: Mijn vader is uiteindelijk overleden vijf jaar later na zijn operatie aan *een uitzaaiing van zijn nier*. Toen toch nog, wat in z’n … terecht gekomen is. 00:11:20-9 En ja, goed, mijn moeder staat hierbuiten maar is inmiddels ook overleden aan kanker. 00:11:28-3 Ja, verder heb ik daarna ook wel huidkanker gekregen en in mijn schildklieren een tumor, maar ja dat houdt geen gevolgen met, tenminste.. 00:11:38-4

Gesprekleidster 1: Ja, dat kan hè, daar voor het gesprek inderdaad even over gedaan, dat is misschien iets om op het eind nog even op terug te komen. 00:11:45-7

M: Ik krijg elke twee jaar onderzoek voor mijn darmen elk jaar mijn nieren en blaas en dergelijke, ja, ik ben springlevend. 00:12:00-0 Ja, uiteindelijk de knop om, ik ga, elke maandag ga ik naar de dermatoloog daar hoor ik meer daarover. ".." 00:12:06-3 Die heeft de melanomen weggesneden. Ik heb wel vaak net gezegd van snij een stukje verder, en dan kom je 10-12 dagen later terug en dan is het van ja, we moeten toch een stukje verder.

Gelach

M: Dan moet je twee keer terug, ".." 00:12:29-9 Ja, schildklier, daar loop ik al heel lang mee. Ook al het postuur gezien. Ik moet echt blijven eten om op gewicht te blijven. Ik had gehoopt met, nou ja, nu de helft weg was 00:12:39-8 de tumor had die weg was. Dat het beter zou zijn. Niet dus. Toch nog echt goed blijven eten. Om op gewicht te blijven. Ja en verder ben ik springlevend, ja. 00:12:55-1

Gelach

Gesprekleidster 1: En bij u? 00:12:57-2

M: Ja, ik weet het sinds 2005 en mijn vader is 40 jaar overleden aan darmkanker. En um, mijn broer, twee na oudste broer had ook darmkanker. En toen is het gaan rollen eigenlijk. Of dat we mee wilden doen aan onderzoek. En dat hebben we toen ook gedaan en in 2005 hebben we allemaal DNA onderzoek gehad en van de 12 thuis, zijn er twee niet. Dus die horen niet bij de familie zeggen we dan. En ik kom hier elk, bijna elk jaar, voor mijn onderzoek. En verders niet. Ze hebben wel twee keer poliepen weggehaald had, maar verders is het goed. En mijn oudste broer dacht, nou die heeft net ook kanker, nog wel kanker gehad dat ze.. weggehaald hebben, zeg maar.

Gesprekleidster 1: Zo zijn er best wel grote verschillen, hè? Het is de 1 heeft niets gehad en de ander behoorlijk. <u>00:14:13-9</u>

V: Wat het benadrukt bij Lynch syndroom vooral ligt op de dikke darm. Maar ik kom uit een hele grote familie en van mijn moeder zijn er tien overleden tussen de 30 en 50 en die hadden lang niet allemaal darm kanker. Mijn broers en zussen ook overwegend lang niet allemaal darmkanker.

Gesprekleidster 1: Wat voor type kanker hebben zij gehad?

V: Ik ben begonnen met eierstokkanker. Mijn zus baarmoeder kanker, um, mijn broer die begon met dikke darmkanker en die heeft nu prostaatkanker. En onlangs is mijn nichtje is ook overleden 37 aan borstkanker, daar wordt wel van gezegd dat het niet erfelijk is. En dat het niet met het Lynch syndroom te maken heeft. Maar ik heb het idee dat met voorschrijdend inzicht het wel iets mee te maken heeft.

Wat voor informatie heeft u verkregen?
Gesprekleidster 1: En vanaf het moment dat u weet dat u drager bent van het Lynch syndroom, heeft u, wat voor informatie heeft u gekregen? Van uw zorgverleners? <u>00:15:23-5</u>

V: Je gaat zelf op zoek denk ik hè, dat doe je het meeste. Je krijgt wel informatie van je specialist enzo maar <u>00:15:32-5</u>

Gesprekleidster 1: Wat voor informatie bijvoorbeeld heeft u gekregen van uw specialist vanaf het moment dat u het te horen kreeg? <u>00:15:32-6</u>

V: Dat het een erfelijke aandoening is. Na het DNA onderzoek weet je in welk gen het zit. Maar dan ga je daar niet over leefstijl ofzo. Alleen maar informatie over dat is de ziekte en wat je daar verder van moet weten, ja, dat kan ik zelf allemaal op gaan zoeken. 00:15:57-1

V: Meer technische informatie vond ik, hè. Wat houdt het onderzoek in en dat soort dingen? Maar over leefstijl helemaal niet. Emotioneel vond ik sowieso helemaal geen."

M: Ze zeiden van je bent gendrager. Oh, leuk, zei ik. 00:16:15-5 Dan word ik ook elk jaar onderhouden.

V: Dat is wel een voordeel.

M: In principe leuk dat ze er bij zijn, maar je kunt niet laten stoppen. Meestal is het altijd te laat. <u>00:16:31-0</u>

V: Ja, maar dat gaat eigenlijk alleen maar in het geval van darmkanker. Altijd kom je er achter aan. <u>00:16:38-9</u>

M: Ja, altijd er achter aan, ja.

V: Nu kun je het weghalen het is een voorstadium, maar aan de andere vormen van kanker, je

M: Je kan het toch krijgen.

V: Je komt pas tot een onderzoek als er iets is.

M: Het rare is, dat wat u zegt dat klopt ook eigenlijk wel. Mijn moeder heeft dus heel lang, het is begonnen met borstkanker. Daaruit voort kwam dus botkanker, of um, borstkanker daar werd het door '..' En die is uiteindelijk overleden aan leverkanker, maar die dronk een wijntje hier en een wijntje daar. En dat sukkelde zo twee jaar door. Er werd steeds gefocust op borst en bot. <u>00:17:21-9</u>

Gesprekleidster 2: Waren er geen uitzaaiingen van. <u>00:17:22-9</u>

M: Nee, het was helemaal aan voorbij gegaan. En dan zeg ik ook altijd van ze zijn heel gefocust op mijn

darmen mijn nieren en mijn blaas. En ik ga dadelijk dood aan hersentumor. Haha, dat zien ze niet.

V: Waarschijnlijk wel, hè? 00:17:36-1

M: Daar gaan ze aan voorbij, want ze zijn zo gefocust op, althans, ik heb het Lynch syndroom, dus als ik iets heb, dan zal het daar wel mee te maken hebben.

V: Ja, en wat mij stoort, is dat het zo snel weg geworpen wordt als je met andere klachten komt van dat daar nooit, of nou ja, nooit, dat is veel te sterk gezegd. Je moet een goede arts treffen die zegt van, ja, misschien heeft het er wel mee te maken toch. Van toen ik in '98 in een perifeer ziekenhuis kwam, eierstok kanker. Was de eerste reactie van de arts daar, dat heeft helemaal niets daarmee te maken. Ik ben dat blijven volhouden van ik heb dat gevoel toch. Ik ben doorgegaan naar *ZKH7*. Daar hebben ze het in de literatuur gezocht en in de Canadese literatuur vonden ze dat het er wel mee te maken heeft. Nu onlangs met mijn nichtje ook weer, dan denk je die stelligheid waarmee gezegd wordt van dat het er niet is.

Gesprekleidster 1: Het heeft ook met voortschrijdend inzicht te maken, denk ik. De wetenschap die ontwikkelt zich steeds verder. 00:18:43-1

M: Belangrijk zelf, wat ik ervaren heb, en dat geef ik ook aan familieleden door, 00:18:43-9 stel dat er iets is, ga gewoon langs het gespecialiseerd ziekenhuis 00:18:49-3 waar ze weten waar ze mee bezig zijn. Met alle respect voor andere dokters ook, voor wat dan ook. Ze weten net het laatste stukje niet.

V: En ik denk dat het heel belangrijk is.

M: Ja, dat is van, ik ga naar mijn eigen ziekenhuis lekker dichtbij, maar dan rijd je een stukje verder. Mensen zijn daar, die weten waar ze mee bezig zijn. Ze weten waar ze moeten kijken, want dan ben je een stukje voor. En als er iets is aan de bel trekken en doorverwijzen. Als je niet verwezen bent verder zoeken, want je hebt het zelf in de hand.

V: Je weet het pas als je gediagnosticeerd bent.

M: Ja, maar dan ben je weer gewoon mens, dus je hebt andere gevoelens en andere dingen wat kan komen. Dus ja, of dat is dat weet je niet, maar je moet er zelf continu er achter aan, want ze gaan je niet opbellen van hoe is het met je? Gaat het nog. Want we zijn met te veel. 00:19:33-8

M: Je moet heel mondig zijn.

M: Ja

Gesprekleidster 1: Dat is ook een beetje van deze tijd ook, hè? 00:19:41-2

M: Ja, maar dat moeten we ook leren, want anders dan blijf je zitten.

Gesprekleidster 1: En wat er ook mee te maken heeft, is dat natuurlijk een syndroom is dat niet heel vaak voorkomt. Dus een normale huisarts ziet bijvoorbeeld niet heel vaak mensen met het Lynch syndroom. 00:19:53-3

V: Ik heb daar wel regelmatig naar geïnformeerd, maar zit het wel in de studie van de artsen. Voor was dat een beetje wazig. Er wordt veel meer naar gekeken. Dat ze daar rekening mee moeten houden. 00:20:10-6

M: Het is ook met twee verschillende afwijkingen met MSA6 en het andere daar kan ook wat in zitten, hè?

V: Het kan in meerdere genen zitten.

M: Ja, ze zeggen van een hele andere kant, ja, bij de vrouw is het hoofdzakelijk de baarmoeder en bij de

man de darmen.

V: En het ligt aan in welk gen het zit.

M: Ja, en dat is wel heel bepalend.

M: Ik had een opgezette klier en dan wordt er heel gauw gezegd, van ah, weet je. Ik wil gewoon dat er naar gekeken wordt. En dat wordt al heel snel, ja, nee, maar, dat hoeft niet. 00:20:44-5 Het gaat wel over, kom over 14 dagen maar terug. En dan moet je echt mondig zijn. Daar wordt inderdaad een punctie gemaakt en daar komt niet veel uit. Ja, nee, het zal allemaal wel meevallen, nou, nee, het valt niet mee 00:20:53-5 . Ik wil wel weten wat het is. Dus toen heb ik nu een scan gehad, dat is iets wat er niet thuis hoort. En dan kom je bij de chirurg, ja, ja, ja, wacht jij een week of 6, maar goed, tenminste kreeg je het van de assistent te horen. Toen heb ik de chirurg gesproken en drie dagen moest ik al komen. Dus je moet echt mondig zijn door je en nogmaals, ze zullen ook heel veel publiek binnen krijgen wat ja niks mankeert. Denk ik ook wel. Maar ik denk dat het dan zaak is van artsen dat ze je achtergrond kennen, 00:21:30-5 misschien heeft het er wel helemaal niet mee te maken, het 1 met de ander. Maar toch.

V: Ja precies, er zijn ook momenten dat het niet bekend is. De reclames van het KWF van als je bepaalde klachten hebt, ga je naar de huisarts. Ik vloeide en ik ben naar de huisarts geweest, ik heb een jaar bij de huisarts gelopen. En ja, u zegt specialistisch ziekenhuis, ik ben behandeld in het *ZKH5* dat heeft drie maanden geduurd en nog steeds wordt er tegen mij gezegd ga er maar vanuit dat het niets is, ga er maar vanuit dat het niets is. Totdat ik hier op de uitslag zat in mijn eentje op de poli en dat ze zeiden het is kanker. En alles moest eruit, dat ik dacht van, woow, hoe kan dat nou? Er is al die tijd tegen mij gezegd elke keer waar men er dus meer er van afweet, ga er maar vanuit dat het niets is. 00:22:17-1

V: Toen wist je nog niet dat je het Lynch syndroom hebt?

V: Nee, want dat is daarna pas gaan rollen. Ik was 41 en dat is wel wat de gynaecoloog gezegd heeft. Je hebt op die leeftijd komt baarmoederkanker eigenlijk zelden voor. Of het zou iets anders kunnen zijn. Nou, en dat andere, dat, dat leek voor mij een betere verklaring, totdat ik een half jaar later mijn tante kwam. Toen was het, dan is het niet een andere verklaring, dan is het dit. En dat was al wel heel snel helder. 00:22:48-2

M: Bent u eigenlijk het eerste voorbeeld van de familie. De eerste keer dat men signaleert dat dat naar buiten komt. Dat hadden wij dus ook in de familie. Zo gauw eentje het heeft van het is abnormaal komt er steeds meer uit, dan ga je onderzoek doen. Ja, dan ben je er sneller bij. Dat is dan eigenlijk pech, misschien dat u dat bent de eerste, maar het is wel voor de rest van de familie dan wel fijner dat er dan sneller actie ondernomen kan worden.

V: Ja, voor de rest van de familie wel, maar als ik kijk hoe het gegaan is en bij de huisarts en dan toch hier een beetje in het *ZKH5*. Dat ik denk van ik heb uiteindelijk meer dan anderhalf jaar met klachten gelopen en en en terwijl er gezegd wordt, heb je iets afwijkends ga zo snel mogelijk naar de dokter. Dat je dan denkt. Maar..

M: Ik zeg ook van je kunt het op een rij zetten, als je ergens in als het klikt dat het goed gaat. Helemaal begrepen, helemaal geholpen, dan is dat goed. En ja, het onderzoek ja, dan ben je ook afhankelijk van, er zijn maar een aantal gespecialiseerde ziekenhuizen die dat misschien wel zien. 00:23:45-3

V: Maar het *ZKH5* is gespecialiseerd, snap je.

M: Maar die tool heb ik dus nooit gehad. Er werd ons wel gezegd, toen het bekend was, tegen m'n zus en mij, dan krijg je een gesprek met een psycholoog of tenminste die tekst uitleg, nou, dat is nooit geweest. Ik heb nooit geen uitnodiging voor gehad. Dus wij hebben ook nooit geen handvaten gekregen van, ga daar zo mee om. Misschien ook wel mijn eigen schuld, ben er ook niet achteraan gegaan. Maar ik denk wel dat het wijs is, want op het moment. Bijvoorbeeld, ik woon in Veghel, dus ik ben verbonden aan het ziekenhuis. Ik heb ooit een virus in mijn darm gehad, wat heel veel leek op blinde darmontsteking. Ben ik voor geopereerd, dat bleek geen blinde darm te zijn wat wel is gezien. Ik had toen zoiets gezegd tegen de arts, ik ga liever

naar *ZKH5*. Daar kom ik al elke 2 jaar voor mijn darmen, daar is al meer bekend. Maar dat werd afgehouden. Nee, je gaat niet naar *ZKH5*, je gaat daar naar toe. En dat vind ik al heel vreemd dat want tot op vandaag weet ik nog steeds niet wat het is.

Gesprekleidster 1: Wat het dan geweest is?

M: En nou, ik verging op het eind. 00:24:49-9

V: Kunnen ze dat navragen?

Gesprekleidster 1: Ja, maar ze wisten toen niet wat er aan de hand was.

M: Ze hebben toen uiteindelijk wel die blinde darm verwijderd. Ja, nou ligt de boel al open. 00:25:02-1

Gelach 00:25:07-3

M: Dan halen we dat er vast uit, dan kan dat het in ieder geval nooit meer zijn. Maar als je dan vraagt, ja mevrouw wat is het dan? De laatste dag, zei tegen mij later, van ja, het zou een virus kunnen zijn. Maar ja, dat is ook zoiets. Ja

Gesprekleidster 1: Zie, wat betreft zie je toch wel, dat in de medische wetenschap ze zijn heel ver, maar op, soms weten ze het ook gewoon niet. Er zijn gewoon niet antwoorden op al onze vragen, zeg maar. Eventjes terug naar het onderwerp, heeft u, we hebben het net gehad over wat voor informatie u heeft gekregen nadat u wist dat u drager was. Is al kort even ter sprake geweest hoor, maar heeft u daarbij ook informatie over leefstijl in relatie tot Lynch syndroom of op het krijgen kanker gekregen? 00:25:51-7

M: Ik kreeg informatie van de vereniging. Waarbij ik geabonneerd ben 00:25:55-7 daar lees je veel in over het onderzoek van jullie uit en zeg het maar. En daaruit eigenlijk en je gaat zelf wat gezonder leven en bepaalde dingen beter kunt verdragen. 00:26:07-2

V: Noodgedwongen haha

Gesprekleidster 1: Ja precies, en is dat voor jullie ook allebei, hebben jullie ook geen informatie gekregen?

V: Daarna had ze wel verteld wat ik wel en niet moest doen. Elke dag een frikadel speciaal op die manier. En dat in China veel maagkanker kwam door scherpe kruiden, dus hier en vooral in het **** darm kanker. 00:26:31-3

M: Dat hebben ze mij nooit verteld. Maar goed, natuurlijk weet je zelf wel grotendeels wat goed en slecht voor je is. Roken, drinken en beweging, voeding dat weet je zelf wel, maar echt specifiek dat wordt gezegd let hier op. Nee, dat is nooit het geval geweest.

M: Bij jullie kan dus natuurlijk wel dat de informatie gewoon van het roken, dat is gewoon echt slecht. Dat was wel gerelateerd dat je sneller poliepen zou krijgen. En wat dan weg komt. Er was wel advies van heb ik wel gekregen van als je zou roken kunt u bij ons opgeven en dan gaan we helpen om te stoppen. Het is duidelijk bewezen dat dat invloed heeft. 00:27:08-1

V: Dat dat invloed heeft, ja oké.

M: Maar, misschien dat dat komt omdat ik vanaf het begin af aan meer gedaan heb. En leefstijl veranderen, het onderzoek wat ik eet elke dag, zeg maar. Want je hoort steeds dat dat stress opgeleverd heeft en dat 00:27:23-4 en dat heb ik gewoon elke keer aan meegedaan. Dat heb ik ook gestuurd. Misschien dat het dan is dat, ja, dan in het systeem zit en sneller.

M: Ja, dat doe ik ook.

V: Ik ook 00:27:33-5

M: Ja?

M: Ik ben er wel achter gekomen zelf, als ik pudding at dan tien minuten later zat ik op de wc. En denk dat ik eens een keer geen pudding neem en dan is het ook over. Dus ik kan niet tegen melkproducten. 00:27:51-2

Gesprekleidster 2: Je bent het zelf een beetje gaan uitvogelen.

Meerderen: ja

Gesprekleidster 1: Dat hoor ik een beetje terug hè, dat je zelf eigenlijk van zelf merkt waar je wel en niet tegen kan. 00:27:59-6

M: Wat ik 's morgen wel kan eten is een bak geitenkwark met bosbessen.

V: Ja, anderen kunnen er weer wel tegen. 00:28:05-4

M: En dan als het niet kan, dan merk je het ook meteen.

M: Nee, nee, dat stopt wel. En dan zelf zoeken wat je kunt verdragen, en dat moet je weglaten. Je weet van, het is een belasting je hebt niet zoveel zouten. En je merkt gewoon, ja, ik heb tenminste mijn dikke darm, ik mis gewoon heel veel zouten. Als ik last krijg, dan moet ik zouten hebben, dat stukje je kan niet meer opnemen als het binnen komt. Als het teveel is komt het er ook weer uit. Dat is die balans zoeken. 00:28:35-7

V: Toch blijf ik zelf toch altijd wel een beetje ambivalent als het gaat om leefstijl en de invloed moet ik zeggen, hoor. Als ik naar mezelf kijk, ik heb eigenlijk altijd heel veel gesport, heel veel bewogen, um, relatief ook gezond gegeten. En dan denk je en toch krijg ik het. Hè, en, en, waarom zou ik dan mezelf allerlei beperkingen opleggen?

M: Maar, dat is ook bewezen hè, dat het niks mee te maken heeft met hoe je gezond leeft of slechte gewoontes hebt, het gaat gewoon door het gen. 00:29:02-3

V: Ja, precies 00:29:02-3

M: Dat is bewezen. Alleen het is wel zo, als je slechte voeding pakt, um, neemt, dan kan het wel, hè, maar niet specifiek, want het gen heb je staat erin. 00:29:15-6

V: Krijg je echt met een blauwe bes niet weg. 00:29:19-2

Gelach 00:29:19-2

M: Kan een placebo zijn hè, als ik het gevoel heb dat het goed is, hè, dat zit hier ook.

M: Tussen je oren.

V: Ja, nou mijn dochter heeft ook het gen, een van mijn dochters heeft ook het gen geërfd 00:29:32-7, minder erg. En ja, ze is nu 30, ik heb gezegd van probeer zo gezond mogelijk te leven want als het gaat komen, dan heb je een gezond mogelijk lichaam die strijd aan moet gaan.

Gesprekleidster 1: Dat is inderdaad een heel goed.. 00:29:47-7

M: Dat is heel belangrijk.

Gesprekleidster 1: Want dat weten we inderdaad wel, dat op het moment dat je een gezonde leefstijl hebt

dat je een algehele gezondheid hebt en dat je ook beter de behandelingen kunt doorstaan. Dat is van belang. 00:30:03-4

V: Maar goed, dat geldt natuurlijk voor alle vormen van kanker.

Meerderen: ja

V: Maar ja, niet iedereen heeft een grotere kans erop.

V: Nee dat is wel zo, maar goed de link is natuurlijk wel. Dus of je nou het syndroom hebt of een ander soort kanker, ja

Gesprekleidster 1: Het is ook zo. Het is altijd goed. 00:30:28-5

M: Maar ja, inderdaad je moet wel je kwaliteit van leven in de gaten houden

M: Ik drink niet, gewoon omdat ik het niet lekker vind, 00:30:35-6 dat is altijd leuk. Maar ik rook wel en dat is niet goed. Dat is gewoon slecht, dat weet ik. Ik heb ook wel is een cursus stoppen met roken gedaan, nou, dat ik twee maanden volgehouden. Ik ben er al eens eerder 12 jaar vanaf geweest. 00:30:50-4 Maar goed, dat is het ding, dat weet ik voor mezelf, daar moet ik iets mee gaan doen. Maar dat is niet alleen voor je, het is voor alles goed.

Gesprekleidster 1: Ja precies. Ik hoorde je net zeggen, jullie hebben eigenlijk sinds dat jullie weten dat jullie drager zijn, met name door de operatie, de behandeling daarvan, aanpassing in je beweegpatroon, of, sorry, in je voedingspatroon gemaakt. Niet allemaal, maar met name eigenlijk door de ingreep doet dat je minder dingen kunt verdragen. Dat je zelf leert, wel, van hé, dit wel, dit niet. Dus eigenlijk noodgedwongen maak je aanpassingen in je voedingspatroon. Maar zijn er in andere leefstijl gedragingen ook bewust of onbewust aanpassingen geweest? In bijvoorbeeld beweegpatroon of met roken? Kan zijn dat je op het moment dat je diagnose krijgt van, nou stop ik of iets dergelijks. Of zijn er andere aanpassingen geweest? Of is het met name gewon de voeding aan de hand van de operatie en klachten eventueel die je daarvan krijgt?

M: Aanpassingen, dat ik wel wat meer rust. Haha. Kijk ik heb vijf batterijtjes op een dag 00:31:54-7 Dat moet je niet willen, dat gaat te snel op. Dus ja, dan weet je van het gaat niet. 00:31:59-2

Gesprekleidster 1: Dus energie wat beter verdelen op een dag. 00:32:03-3

M: Ik ben nogal gedreven. Ik leg de lat hoog 00:32:03-9 en ik weet, ja, dat gaat fout. Naarmate je ouder wordt, en dan realiseer je van toch even terugschakelen. Dan gaat het gewoon niet. En dan bewust zeggen, dan maar parttime 00:32:13-8 werken, gewoon bewust, dat je je rust neemt. 00:32:22-6 Dan is geld niet belangrijk, hoor.

V: Wat dat betreft blijft gezondheid toch het belangrijkste, hè?

M: ja

M: Ja, ik heb het idee ondanks dat ik het syndroom heb, dat ik kerngezond ben.

Meerderen: Ja

M: Gelukkig maar

M: ‘door elkaar gepraat’ 00:32:44-2

Gesprekleidster 1: De pudding, dat er niet heel veel andere dingen zijn die je moet laten staan. Dat is alleen maar fijn. 00:32:58-9

M: "Dat is ontzettend verschillend"

Gesprekleidster 1: Ja precies, dat blijkt eigenlijk al wel, hè? Nu het zo verschillend is.

M: Maar je vindt daar wel een weg erin.

M: Ik heb er wel een half jaar veel last van gehad toen ze het zeiden, en toen had ik zoiets van ja, luister whatever, ik moet vooruit. En ze houden mij goed in de gaten. Dus ja, wat kan mij overkomen dat klinkt heel kort door de bocht.

M: Ja, zo denk ik ook.

Gesprekleidster 2: Denkt de rest daar ook zo over? 00:33:23-0

Meerderen: ja

V: Ik denk ook leefstijl, dat heb ik niet aangepast omdat ik die uitslag heb gekregen, maar omdat ik gewoon vind dat je gezond moet leven. Dus het was voor mij niet gekoppeld aan, um. 00:33:36-1

V: Niet zozeer aan het Lynch syndroom.

V: Nee

V: het geldt voor ieder mens. 00:33:37-6

V: ja

Gesprekleidster 1: Maar dat, betekent dat dan dat u daar van te voren al goed mee bezig was voordat u überhaupt met een gezonde leefstijl? 00:33:50-0

V: Ja, ik voelde me behoorlijk gepakt.

Gesprekleidster 1: Ja dat kan ik me voorstellen. Wat V1 ook zegt, hè, ik ben altijd zo actief geweest en altijd zo voor je gevoel hè. Wat dat betreft is het inderdaad ook, ja, we weten ook gewoon niet precies hoe en wat. Hoe het allemaal werkt, we weten natuurlijk al wat over de relatie tussen leefstijl en kanker. Daar kom ik zo meteen ook op terug, um 00:34:21-9

M: Maar wat is gezond, hè? Als je gaat zoeken wat is gezond, en dan kun met sla, hè, die '..'   Er is overal iets.   00:34:33-0

M: Je bent ook mens, hè, we hebben dit als extra erbij.

M: Ja, dat weet ik wel. Maar als je echt gaat zoeken wat is nou gezonde voeding? Er is overal we iets mee aan de hand.   00:34:43-3

Gesprekleidster 1: Je bedoelt dat bestrijdingsmiddelen bijvoorbeeld op groente zitten?

M: Ja, er is jaren gezegd want een glas wijn elke dag goed en nou is het weer niet goed.
door elkaar gepraat.

V: "ligt er ook aan vanuit welke discipline het gezegd wordt. 00:35:02-6

Gesprekleidster 1: Ja, dat is ook belangrijk, welke bron komt dat vandaan?

V: Als je kijkt inderdaad van wat is gezond eten, los van wat er wel goed is en wat er niet goed is. Ik heb wel

lijsten gezien waarin je dan kunt beoordelen van jezelf doe ik dat of doe ik dat niet. Dan denk ik van, ja, ik denk dat ik gezond eet, maar in praktijk valt dat wel heel erg tegen vind ik, hoor. Dat, ik eet geen volkoren brood, ik eet tarwebrood. Dus daar zit al iets in. Ik eet veel kaas, terwijl het eigenlijk beter is van niet. Dus als je dan hebt van, ja, heb je een gezonde leefstijl? Voordat ik het kreeg, had ik dat wat meer, vind ik. Maar als ik dan nu kijk, dan denk ik van, nou. 00:35:40-7

V: Dat is ook die constant veranderende inzichten, hè? Ze zijn natuurlijk opgegroeid van je moet melk drinken en kaas eten, dus dat veranderd, maar bij mij bijvoorbeeld niet. Ik vind het nog steeds heel lekker dus.

V: Ja, precies.

M: Als ik kijk bij ons op school, wij krijgen de gezonde school, daar hebben ze de automaat aangepast. Wat zit er in allemaal light met zoetstoffen, ja, dat is gewoon troep. Dan denk ik, ja, waar zijn we dan mee bezig. Het wordt dan door de maatschappij, dat is gezond. Nou, echt niet. 00:36:10-4

V: Dat heeft ook heel veel te maken met omgevingsfactoren, hoe gezond is de lucht om je heen? Heleboel dingen kun je bedenken wat invloed zou kunnen hebben op je lichaam.

M: Ik denk dat je daar helemaal niet zoveel bij stil moet staan. Anders kun je echt niet meer leven, want je moet vooruit. Het overkomt je en daar vraag je ook niet om. En dat kan je plaats geven en het gaat sneller een jaar is zo rond. Je moet er wat van maken. 00:36:38-4 Carpe Diem.

M: De enige aanpassing die ik heb gedaan is met werk. Ik maak heel veel uren, zit heel veel in de auto. Ik maak 70, 80.000 kilometer per jaar. 's morgen vaak heel veel rusten, vaak 's morgen om half 6 in de auto, 's avonds om 7 uur, half 8 thuis. Daar heb ik wel tegenin gezegd van, het is allemaal wel goed. 'S morgens ben ik wel op tijd weg, maar mijn streven is het voor 's avonds maximaal om 6 uur thuis zijn. Kunnen ze hoog en laag springen, maar daar heb ik wel een streep doorgezet.

Gesprekleidster 1: Ja 00:37:09-9

M: Dat is ook niet goed natuurlijk, om 60,70 kilometer te maken. En maar, dat, dan loop je toch wel vaak tegen onbegrip op. Ja, maar jou functie deed dat, dat zal allemaal wel. 00:37:23-6

V: Maar weet men dat dan dat jij dat hebt?

M: Ja, dat heb ik verteld. En het lullige is, mijn direct leiding gevende die zei: ik snap niet dat jij je hebt laten onderzoeken. Wil je dat weten? Zegt hij. Is toch alleen maar lastig als je dat weet?

V: Dat deden ze bij ons in de familie ook.

M: Ja, dan denk ik nou, ja, laat maar. Wil ik me voor de rest ook niet mee bezig houden. 00:37:41-2

V: Ik denk altijd als je aanvoelt dat je niet begrepen wordt. Moet je er geen woorden meer aan vuil maken, want je raakt alleen maar gefrustreerd. 00:37:50-8

V: Ja maar, dan zie ik je ook vaak door stress, telt natuurlijk ook mee.

V: Stress vind ik altijd duidelijk een factor waar je niet altijd invloed op hebt.

V: Nee, daar zijn geen knopjes voor.

Gesprekleidster 1: Dat zou wel handig zijn, als je die vindt dan.

V: Ja, dan geef ik het door.

M: Ik bedoel, wij zitten in een hele moeilijke markt, met heel weinig marges. Ik heb ook afscheid genomen, ik heb Civiel, weg en waterbouw gestudeerd. Ik heb heel vaak 's nachts voor Rijkswaterstaat op de weg gezeten, de nachten begonnen, ’s middags. 00:38:22-7 Was elke avond stress, want om 6 uur moest dat lijntje op de weg staan, anders kreeg je een boete. Daar heb ik afscheid van genomen, vervolgens heb ik gezegd, ik heb het 15 jaar gedaan, dat doe ik niet meer. Binnen dezelfde organisatie leg ik nu sportvelden aan. 00:38:38-3 En dat is al veel “relaxter”. In heel Nederland leg ik sportvelden aan. 00:38:42-9 Omgeving is anders, je hebt niet de stress, de druk elke nacht of elke weekend, want het is vaak meer weekenden. Dus, die dingen heb ik wel allemaal gedaan.

Gesprekleidster 1: En wat is de motivatie daarvoor geweest, om die beslissing te maken?

M: Nou, omdat ik mij daar wel wat beter bij voel. Het heeft mijn huwelijk gekost en ik heb mijn kinderen niet op zien groeien. Daarna had ik zoiets van, nou dat is niet goed. 00:39:09-5

V: Dat kan ik me wel voorstellen, ja. 00:39:12-3

M: Triest, ja 00:39:12-3

Factoren leefstijl

Gesprekleidster 1: Even iets anders, ik wil even naar dit bord toe gaan. We hebben het gehad over leefstijl en over aanpassingen in uw leefstijl die u al dan niet gemaakt heeft. En wij waar wij benieuwd naar zijn zijn factoren die nou die samenhangen met danwel het aanpassen van uw leefstijl, danwel het niet aanpassen. Factoren die eraan bijdragen dat je gezonder gaat leven. Factoren die er aan bijdragen dat je niet gezonder gaat leven. Dat je ervoor kiest om niet gezonder te gaan leven. Daarvoor hebben we dit schema gemaakt en niet alleen voor het aanpassen van leefstijl maar ook het volhouden van bepaalde aanpassingen. Want daar zijn natuurlijk ook allerlei factoren van invloed. Dus wat ik, het doel is dus daar over hebben en eigenlijk wat dingen op een rijtje gaan zetten. Nou, is het misschien handig om even te kijken naar leefstijlfactoren die u in het verleden ooit heeft veranderd. Volgens mij zijn er niet zo heel erg veel dingen die jullie bewust hebben aangepast. Of in ieder geval niet in relatie tot het feit dat je te horen hebt gekregen dat je het syndroom hebt. Maar ik hoor je wel zeggen dat jullie in het verleden wel meer zijn gaan bewegen of in ieder geval gelet hebben op gezond eten. Gesprekleidster 2 zal daar wat dingen uit opschrijven. Wat zijn nou factoren voor jullie dat ertoe bijdragen dat je bijvoorbeeld zou willen stoppen met roken. Dat je wilt letten op je voedingspatroon. Dat of, dat je meer zou willen bewegen. Wat zijn factoren die daar voor jullie van invloed zijn? Of wat zijn redenen dat jullie..?

V: Tijd, keuzes

Gesprekleidster 1: En tijd is dus met name dan een iets wat aan de minkant moet. Hè, dat is dan iets wat in de weg staat? En dan is dat dan voor zowel voeding en bewegen? 00:41:31-6

V: Ja, nou met name beweging. 00:41:37-0

Gesprekleidster 1: Want voeding kan natuurlijk ook. Als je weinig tijd hebt wil je misschien snel iets in elkaar zetten, en heb je weinig tijd om uitgebreid te koken bijvoorbeeld. 00:41:46-7

V: Nee, dat meer tijd, bewegen 00:41:46-9

Gesprekleidster 1: Bewegen, ja oké

Gesprekleidster 2: En nu noemde nog een factor volgens mij.

V: Keuzes maken 00:41:52-5

Gesprekleidster 2: Keuzes maken

Gesprekleidster 1: En wat bedoelt u daarmee?

V: Nou, wat ik net eigenlijk ook zei, als ik kijk naar mijn leefpatroon voordat ik dit kreeg, zeg maar. Was ik eigenlijk nog alleen en nu heb ik een partner. En nu zie ik mijn leven relatief ongezonder vind ik, vaker uit eten.

V: ja, ja, precies.

V: ".." dat dan nu laten staan. Nee, precies, dus maak je die keuze of niet? Om gezonder te willen leven. 00:42:22-7

M: Maar maakt je wel gelukkiger om dat te doen. Ja, nee dat is ook belangrijk. Je moet je er goed bij voelen je moet niet alles.

V: ".." 00:42:30-5 wat ook weer van invloed heeft, hè. Op je hele gesteldheid. 00:42:34-9

Gesprekleidster 1: En zo blijven we bezig, haha

M: Ja, maar wat is goed en wat is fout? Ik merk gewoon ik heb overdag vanmorgen beweging, echt sporten doe ik niet. Maar ik heb bijvoorbeeld heel lang drie keer in de week 15 km hardlopen. Ik krijg dat met mijn gewicht niet voor elkaar. Ik eet twaalf snee brood op een dag met roomboter en goede vleeswaren. 00:42:56-6

V: Lekker

Gelach

V: Dat zijn gezonde keuzes 00:43:07-6

M: Dat gewicht is voor mij zo belangrijk als we bijvoorbeeld op vakantie gaan dan is dat altijd rommelig 00:43:10-8 Dan je zit je dus een dag in de auto dan ben ik gewoon twee kilo kwijt. 00:43:21-8

Gelach

M: Ja, maar goed, snoeper ben ik daarentegen niet. Dat is gewoon zo.

V: Stoppen met roken kom je vanzelf ook aan in gewicht. 00:43:29-7

M: Ja, dat is wel een goede

M: Maar goed, je moet keuzes maken inderdaad. Maar ik vind ook in mijn achterhoofd ik wil wel en dadelijk moet ik uit eten, ja, dat is gewoon leuk.

V: Ik merk dat het voor mij een keuze is om bijna geen vlees meer te eten. Maar dan niet, ja, ook voor mijn gezondheid, maar ook voor dierenwelzijn. En de manier waarop wij daarmee omgaan. Ja, dat heeft eigenlijk niets te maken met mijn syndroom, maar wel dat ik mij lekkerder voel.

V: *DR.2* geeft wel aan dat je minder vlees moet eten. 00:44:09-5

Gesprekleidster 1: Wie geeft dat aan? *DR.2* bedoelt u, ja, ja, ja

V: Die geeft dat wel aan.

V: Die zei dat rauw vlees, rood vlees

Gesprekleidster 1: Dat is de professor waarmee ik samenwerk, professor voeding en kanker 00:44:27-7

Gesprekleidster 2: uit Wageningen 00:44:32-0

V: Ik heb laatst een keer op de bijeenkomst van de vereniging gesproken. Het is een mooi verhaal.

M: Maar goed, inderdaad keuzes als je dan echt zegt. Ik ben fan van motorrijden ik heb altijd op Japanners gereden tot tien jaar geleden. Altijd scheurijzers. Toen is die knop ook omgegaan, ja, dat kan ik ook niet blijven doen. Leeftijd, gevaar. Ik ben echt zo gek als een deur geweest 00:44:51-9 Dan ik wilde per se een Harley Davidson hebben, nou goed, als je daar in de winkel staat, dan slik je een keer. Maar uiteindelijk heb ik dat wel gedaan. Maar dan heb ik daarna mijn schildklier, dan heb ik zoiets van nou, dat is de zoveelste. En het is een hele bak geld, maar ik heb er wel zoveel plezier van. Dat zijn de keuzes. 00:45:14-3

Gesprekleidster 1: Ja, dat klopt.

M: En daar haal ik heel veel energie uit.

Gesprekleidster 1: Meer dat je ervoor kiest om op dit moment te genieten ook. 00:45:23-2

M: Ja 00:45:26-1

Gesprekleidster 1: Om dat soort keuzes dan wel nu te maken.

M: Ja, ik ben minder gaan werken. Ik had een eigen zaakje die heb ik verkocht. Want toen werkte ik van 9 tot 's nachts 10, horeca. En dat heb ik verkocht en dat voel ik me rustiger aan. Nou werk ik gewoon, dat is heel iets anders, ook wel wennen. 00:45:52-4

Gelach

Gesprekleidster 1: tijd over 00:45:52-4

M: Maar ja, pilske, die houd ik nog, maak ik niet minder door. Moet ik eigenlijk wel, maar ja, dat ben ik gewend ook en ik doe nou een beetje, op twee plaatsen werk ik nou. En dat is ook voldoende. Dan heb je toch ".." 00:46:10-0 Dadelijk heb ik een dag ".." 00:46:14-1. Een maand geleden dan is mijn zwager begraven, dat gaat zo snel. Dan denk ik ja, de volgende ben jij, hè. 00:46:20-9

Gesprekleidster 2: Dus wat u eigenlijk ook in uw omgeving ziet, heeft ook invloed op..

M: Ja, dat heeft ook invloed, ja

Gesprekleidster 2: Op wat u zelf doet

M: Ja, mijn broer was 62 en toen was hij ook in 1 keer dood. En dan denk ik, ja, dan ben ik het ook. 00:46:38-5

V: Dat weet je nooit, hè? 00:46:38-5

M: Nee, maar dat hoeft niet. Ik hoef het ook niet te weten.

V: Nee, liever niet. 00:46:42-8

M: Maar ik denk ook waarom zou ik zo lang blijven werken? Want we hebben geen kinderen, dus waar moet het dan naar toe, naar wie? 00:46:53-0

V: Ik denk dat je eigenlijk heel erg moet doen waar je jezelf goed bij voelt en dat kan verschillende dingen zijn. De 1 let heel erg op zijn eten, voelt zich daar goed bij en voor mij is het heel erg van, ik leer al ontspannen en niet steeds vinden dat je van alles moet. En van niemand, moet van mezelf. Maar dat leer ik van veel meer, gewoon toestaan dat je rust pakt en in rust leeft. 00:47:31-2 Dat soort dingen meer

00:47:33-2

M: Dat is heel moeilijk 00:47:33-2

V: Ja

M: Ontspannen, want dat hebben wij niet geleerd thuis, hè? Thuis was het alleen maar werken, hè. We waren met 12 thuis en ik kende maar 1 ding, dat is werken. Vakantie, daar had ik nog nooit van gehoord. 00:47:46-9

V: Ja, maar ja, als je eruit valt, dan merk je dat de wereld gewoon door gaat, toch?

M: Daar hebben wij thuis allemaal heel veel moeite mee, ontspannen, omdat wij dat niet gewend zijn.

V: Ik heb een jaar in het ziekenhuis gelegen, van, nou, de wereld ging door, hoor.

M: Ja, ja, dat weet ik ook wel, haha

V: Ik kwam eruit en ik ging weer mee doen. 00:48:05-7

M: Misschien is dat ook wel de leeftijd die een rol speelt. Maar ik heb wel inderdaad jaren geleden de knop omgezet van het interesseert mij helemaal niks meer wat een ander van mij denkt. 00:48:11-3 Daar hield ik heel veel rekening mee. wat vind een ander daarvan. Dan heb ik zoiets van het zal allemaal wel ik doe mijn ding.

V: En ik denk dat dat een hele belangrijke factor is dat je goed bij jezelf nagaat wat goed voelt 00:48:31-7 Maar inderdaad wat voelt bij jou goed? 00:48:44-1

M: En dat zijn best wel veel dingen, hoor. In de begin jaren 2000 heb ik zelf ook een huis laten bouwen. En veel te groot uitgevallen

Gelach

V: zoals de motor 00:48:54-9

V: thema van je leven denk ik

M: Ik heb, in 2011, heb ik dat verkocht en ik ben kleiner gaan wonen. Dat zijn ook keuzes. En ik merk wel, als dat groots is, dat zegt niets over kwaliteit van leven, een huis is voor rust. 00:49:17-6

Gesprekleidster 1: U hecht er minder waarde aan ook.

M: Ik heb wel dat ik dat een operatie heb 00:49:33-9 ondergaan. En ik heb toen bij Centerparcs gewerkt en daar ben ik drie keer terug gevraagd en daarmee mijn contract gewoon niet verlengt. Ik heb zelf het idee omdat ik dat heb gehad. Nu ben ik een ziek persoon, niemand wil iemand met kanker en dat heb ik heel lang verzwegen bij al mijn sollicitaties. En sinds twee jaar geleden heb ik dat pas verteld, omdat ik toch weet dat ik elk jaar een onderzoek moet doen omdat ze weten, oh, je bent ziek. Wat heb je dan, je moet het wel aan iemand vertellen. Dan komt de directeur naar mij toe van, oh, ja, um het is wel fijn dat je het nou vertelt. Ik zeg, ja, zeg eens eerlijk, als ik dat op een sollicitatiegesprek had gezegd als je moet kiezen tussen een gezond iemand en mij, had je mij niet gekozen. Maar was je wel eerlijk geweest, ik zeg eerlijkheid heeft mij niets gebracht. Ik zeg dat is heel makkelijk dat je dat kunt vertellen. Moet je eens weten hoeveel tijd en geld ik investeer in mijn energie en in mijn gezondheid. Ik zeg, dat wil jij niet weten. Dus ja, ik heb sceptisch erin want heel snel, dat heeft mij wel geraakt toen. Om werk te vinden en te zoeken.

V: Als jij ziekt wordt, dan ga 2,5 jaar, moet de werkgever jou betalen.

M: Terwijl hij misschien zieker is als ik, hoor. 00:50:47-5

M: Maar dat niet alleen, als je in WAO terecht komt dan is de werkgever helemaal

M: Dat zijn de vooroordelen wat de mensen hebben, hè? 00:00:59-2

V: Dat is de wetgeving die niet klopt naar mijn mening. 00:00:59-7

M: Nee, klopt zeker niet. 00:00:57-0

V: Grote bedrijven kunnen dat wel dragen, maar als jij een onderneming hebt met enkele mensen kan je er bijna niet dragen. 00:01:11-7

M: En daarom hebben ze je ook het advies gegeven, laat je kinderen niet controleren, voordat ze dertig zijn dat ze alles op orde hebben. En dat ze dan pas controleren of ze drager zijn of niet. 00:01:18-1

M: Nou, maar het is wel belangrijk, dat heb ik met mijn zussen ook afgesproken die hebben ook heel veel dochtertjes op een leeftijd van dan is het wel verstandig om gewoon een keer te laten doen dan hoef je er niet bij te zeggen dat het DNA bevestigd is. Maar je kunt dat onderzoek wel laten doen. En dan staat er niets op papier, maar het is wel verstandig om dat te doen. Want dan ben je wel rond 30 te laat, hoor. 00:01:39-9

M: Ja 00:01:43-5

M: Nee, je bent te laat 00:01:43-5

V: Als jij in een familie zit waarin HNPCC voorkomt, hoef je je DNA nog niet vast te laten stellen, toch in een screeningsprogramma te komen. Dus dan ben je niet te laat 00:01:52-5

V: Nou, de kinderen van mijn zus die hebben bij een hypotheek afsluiting het gewoon niet gemeld. Terwijl ze wel drager zijn. 00:02:00-2

V: Dan vraag ik me af, ik heb het ook wel vaker gehoord, ik vraag me af of dat strafbaar is, maar dat weet ik niet. 00:02:07-2

V: Nee, het is je privacy. 00:02:06-8

M: Er staat er wel onder dat het naar waarheid is ingevuld wordt. 00:02:16-0

V: Als ze er achter komen. 00:02:18-0

V: Ja, maar goed, ik geef ze niet weg. 00:02:18-0

V: Tot op dit moment heeft '..' dat het gevolgen kan hebben. 00:02:25-9

V: Op welke manier zou het voor een bank die iedere maand, het zou alleen gevolgen kunnen hebben als zij niet meer kunnen aflossen. Maar, zo lang zij gewoon werken en aflossen en is er geen enkele bank die op onderzoek uitgaat. 00:02:40-3

M: En zou het alleen maar zijn, omdat het nou bekend is dat dat niet mag dan. 00:02:41-9

M: Nee, alleen maar als je een levensverzekering naast afsluit, wat heel veel gebeurd, die moet dan tot uitkering komen. 00:02:50-1

V: En krijgt de verzekering dan uitkering? Ik ken geen enkel geval. 00:02:56-1

Aanbevelingen WCRF:

Gesprekleidster 1: We gaan weer even terug naar het onderwerp. Ik denk dat wij, er staan inmiddels al behoorlijk wat factoren op papier en ik denk dat wij eventjes naar de richtlijnen gaan. Naar het volgende deel van het gesprek. We hebben het al gehad over leefstijl en de invloed die de leefstijl kan hebben op het ontstaan van kanker. Nu is het zo dat er algemene aanbevelingen zijn ter preventie van kanker. Zijn jullie daarvan op de hoogte dat er überhaupt aanbevelingen zijn ter preventie van kanker? 00:03:35-6

V: Geldt voor ieder mens, hè? 00:03:36-3

Gesprekleidster 1: Ja, dit is echt ter preventie van kanker, dit geldt voor ieder mens inderdaad. Niet specifiek voor mensen met het Lynch syndroom. Kunt u ze allemaal zien? Ik ga ze langs, hoor. Dit zijn aanbevelingen die zijn gebaseerd op onderzoek van het WCRF, het World Cancer Research Fund. Of de wereld kanker onderzoek fonds in Nederland. Kennen jullie dat fonds? 00:03:59-4

stilte 00:04:01-6

Gesprekleidster 1: Oké, nou het is in ieder geval een stichting die, vind ik, wel heel goed werk doet, want zij bekijken namelijk in de wetenschappelijke literatuur wat er bekend is over de relatie van leefstijl en het ontstaan van kanker. En dat stapelen ze allemaal op en op basis daarvan trekken ze een conclusie, van waar nou genoeg wetenschappelijk bewijs voor is, welk voedingsmiddel of welke voedingsstof gerelateerd is aan het ontstaan van kanker. Ze hebben, we noemen dat een systematic review of een meta analyse, gaan ze dan doen en op basis daarvan doen ze een conclusie en doen ze ook aanbevelingen en die aanbevelingen gelden voor de algemene populatie. Die aanbevelingen staan hier, er zijn er tien, er staan er overigens 9 hierop. haha Dat komt ook, daarom zijn nou die groepsgesprekken. 00:05:06-2

Gelach 00:05:06-2

Gesprekleidster 1: Er zijn nog 2 andere, die hebben te maken met borstvoeding. Er is bekend dat het geven van borstvoeding, het krijgen van borstvoeding dat werkt beschermend tegen het krijgen van kanker, en de andere is voor kankeroverlevenden en kankeroverlevenden zijn eigenlijk iedereen die ooit de diagnose kanker heeft gehad, wordt aanbevolen om gewoon deze aanbevelingen te, ja, aan te houden, zeg maar. En dat is eigenlijk omdat het onderzoek dat is gedaan bij de mensen die de diagnose kanker hebben gekregen wat voor invloed leefstijl dan heeft. Op het krijgen van kanker, nou ja, het kwaliteit van leven, daar is wel steeds meer onderzoek naar, maar eigenlijk niet genoeg om op basis daarvan leefstijl aanbevelingen te doen dus daarom zeggen ze, volg deze leefstijlaanbevelingen. Deze aanbevelingen kun je beetje vergelijken met de aanbevelingen die bijvoorbeeld door de hartstichting worden gedaan om hart en vaatziekten te voorkomen. Of door de diabetesstichting om diabetes te vorkomen. Dit is specifiek gericht op kanker. In grote lijnen komt het overeen met preventie van hart en vaatziekten of diabetes. Zo laag mogelijk gewicht, een slanke taille, maar vermijd ondergewicht het is vooral gericht op geen overgewicht hebben, ook omdat dat ook gerelateerd is met het ontstaan van kanker. Beweeg iedere dag minimaal 30 minuten, hoe meer fysieke activiteit, hoe minder risico op kanker, dat is eigenlijk de trend die we terug zien in de wetenschappelijk literatuur, vandaar dat deze aanbeveling gedaan wordt. Dit is overigens gewoon de beweegnorm die hier staat en dat wordt gehanteerd ter preventie van hart en vaatziekten en ouderdomssuiker. Eet minder calorierijk en suikerrijke dranken. Dat heeft te maken met de relatie met overgewicht, hoe meer energierijke producten je tot je neemt, hoe sneller je in gewicht aankomt en hoe hoger het risico op kanker. Eet veel groente, fruit, volkoren producten en peulvruchten. Wat zegt u? 00:07:15-0

M: Niet dat groente en fruit niet gespoten is. Haha 00:07:24-1

Gesprekleidster 1: Oh, ja, ja, precies, maar de reden dat er niet een advies gegeven wordt voor een bepaald product of een bepaalde groente of fruit omdat we niet precies weten welk stofje in die groente en fruit nou precies verantwoordelijk is voordat verlaagde risico. Daarom zeggen ze, varieer zo veel mogelijk dan krijg je altijd dat stofje binnen. Minder rood vlees en geen bewerkt vlees dat is nou een typische aanbeveling die alleen ter preventie van kanker is. Dat geldt niet voor hart en vaat ziekten niet voor diabetes, maar dit is echt een aanbeveling en met name ook dus voor darmkanker. Er is met name aangetoond dat een grote

consumptie van rood vlees, en daar bedoelen we niet vlees dat rauw is als je het bakt, maar vlees dat rauw, rood uitziet. Dat is rood vlees. En bewerkt vlees zijn vleeswaren, allerlei producten aan toegevoegd zijn om langer goed te houden. Liever geen alcohol. Ter preventie van kanker is het zo, dat eigenlijk aangeraden wordt om geen alcohol te drinken. Maar, ter preventie van hart en vaatziekten is de aanbeveling maximaal 1 glas per dag voor een vrouw en maximaal 2 glazen per dag voor een man. En daarom zeggen ze bij het WCRF, wij houden die aanbeveling aan, want we kunnen heel specifiek nu een aanbeveling voor kanker maar als het goed voor het 1 is en minder goed voor het ander. Ja, dan weten mensen natuurlijk ook niet meer wat ze moeten doen dus daarom houden ze het maximum aan, wat ook door de hartstichting gehanteerd wordt. Minder zout eten, dat is ook iets wat in andere aanbevelingen terugkomt. Wat niet direct misschien als een aanbeveling overkomt, maar wat blijkt uit wetenschappelijke literatuur is dat niet aangetoond is dat het nemen van voedingssupplementen dat dat een invloed heeft op het voorkomen van kanker. Dus dat wordt eigenlijk geadviseerd, de aanbeveling bij 8 is eigenlijk, probeer je voedingsstoffen te halen uit je voeding zelf en niet uit je voedingssupplementen. Maar er zijn natuurlijk altijd redenen waarom je wel voedingssupplementen zou gebruiken en het is ook niet zo dat je dat helemaal niet moet doen. Maar over het algemeen is het advies, probeer zo veel mogelijk voedingsstoffen uit je voeding te halen. En natuurlijk niet roken. Dat WCRF focust zich met name op voeding en beweging, maar niet, roken, hoort daar natuurlijk ook bij. Eerst wat ik sowieso daarover wilde zeggen, dat kwam net ook al ter sprake, deze aanbeveling is wat gevonden is uit wetenschappelijk onderzoek, dus het is wel zo dat als je aan 1 of meerdere aanbeveling voldoet het risico wel lager wordt, maar het is niet zo dat als je aan aanbevelingen allemaal gaat doen, dat daarmee kunt voorkomen dat je kanker krijgt. Wat dat betreft is het een wat ingewikkelder verhaal. Want je krijgt wel kanker of je krijgt niet kanker, dus het is ja of nee. Je kunt het risico wel naar beneden halen, maar dat wil niet zeggen dat je niet meer krijgt, dus wat dat betreft komt het gewoon, wat V1 toen straks ook al zei, het wil niet, zou zijn, je kan perfect gezond leven en wel kanker krijgen en je kunt ook gewoon hartstikke ongezond leven en gewoon helemaal.. 01:01:09-0

M: Maar dat is het stuk wat in het boek staat, tijdschrift, een dun persoon en een gezet persoon en dan staat er van wie denkt u dat er niet gezond is? En dan denk je eerder slank persoon, terwijl de gezet persoon beter in de vel zit als de slanke. 01:01:29-4

V: De laatste dingen zijn mensen met wat meer gewicht, van dat die bescherming hebben. 01:01:35-2

Gesprekleidster 2: Iemand gaf als voorbeeld pas geleden je kunt het een beetje vergelijken met inbraak preventie. Je kunt je hele huis geweldig goed beveiligen, maar verlaag je de kans dat er wordt ingebroken, maar het kan nog altijd gebeuren. 01:01:49-3

M: Mijn oma rookt een pak sigaretten per dag en die werd 91. 01:01:59-7

M: Het eten van vis, want hier wordt alleen over vlees. 01:02:04-8

Gesprekleidster 1: Er staat inderdaad geen aanbeveling over in. 01:02:09-7

Gesprekleidster 2: Dan zijn er niet voldoende bewijzen dat het beschermend werkt. 01:02:12-0

Gesprekleidster 1: Of het is niet genoeg onderzocht. 01:02:15-3

Gesprekleidster 2: Dat kan ook, maar er is onvoldoende aanleiding om het op te nemen in het tijdschrift. 01:02:18-5

M: Omdat de ene keer wordt gezegd, vette vis is wel goed voor je. 01:02:22-3

Gesprekleidster 1: Maar dat is voor je algehele gezondheid gewoon zo inderdaad. Die vette vis is inderdaad gezond, maar het dit is echt wat er in relatie met kanker is onderzocht. En op basis daarvan aanbeveling, dus het is niet zo dat, um, dit nou helemaal volledig is. Het is natuurlijk ook wat ik al eerder zei, het is wetenschap, het is gebaseerd op voortschrijdend inzicht dus er komen iedere keer meer onderzoeken bij. En deze aanbevelingen zijn gebaseerd op onderzoek wat continu op gang moet zijn. Er zijn mensen die werken voor het WCRF en die houden iedere dag, nou iedere dag weet ik niet, maar in ieder geval wel

regelmatig, houden ze bij welke nieuwe onderzoeken er zijn en op basis van een keer in de zoveel tijd wordt er een update gemaakt en dan wordt er gekeken, hé, moeten we die aanbevelingen nou aanpassen? Op basis van het onderzoek wat er bij is gekomen, dat, zij zijn daar echt mee bezig. 01:03:14-4

M: Op 2 staat beweeg iedere dag 30 minuten, er zou ook kunnen staan op stressvolle momenten voor mij, ontspanningsdag. Dat je zegt van, neem de tijd voor jezelf. Vooral in deze jachtige wereld van het moet, je krijgt er steeds meer erbij. Dat stukje naar jezelf toe. Lekker bezig. 01:03:33-6

Gesprekleidster 1: Daar is.. 01:03:36-2

M: Mensen kijken tegenwoordig naar wat ze niet hebben. Dat wij hier zitten, beseffen gewoon dat er veel meer is en koester de momenten dat we er zijn. En dat is het stuk, dat je echt met je neus op de feiten gedrukt wordt. 01:03:48-3

Gesprekleidster 1: En dat is heel waardevol, dat inzicht. 01:03:48-9

M: Ja, die niks meegemaakt heeft, of wat dan ook, die zien dat niet. Dat kan ook, dat contrast is groot. Dat is voor sommige dan. 01:04:00-7

Gesprekleidster 1: Nee, maar dat is heel goed. En ik denk dat ook een hele belangrijke bevinding is. Niet dat niet alleen in dit gesprek. 01:04:12-7

M: Daarom is het ook heel raar, ik doe eens niet mee. Ik heb daar, ik wil niet altijd de beweegreden, zal ik moeten verantwoorden waarom, want ze snappen het toch niet. Want je moet het altijd uitleggen, barbecue of eten, het ziet er allemaal, nee sorry, ik doe het niet. Ja, dan ga ik naar huis. Ik doe er niet aan mee. Ze snappen dan niet. Anderen wel misschien, maar ja, dat is een keuze die je moet maken. Als je in het buitenkant, dan zie je het niet hè? 01:04:36-9

Gesprekleidster 1: Nee, inderdaad 01:04:36-9

M: Je weet gewoon dat je zelf er last van krijgt. 01:04:43-2

Gesprekleidster 1: Um, even kijken, u was niet op de hoogte van deze aanbevelingen? Ik ben even kwijt of ik dat al gevraagd had ja of nee. Je hebt *Prof* ook horen praten, hè? 01:04:58-0

V: Ik heb ook tien jaar voor de vereniging gewerkt. 01:05:02-7

Gesprekleidster 1: Ah, ja, ja, dan krijg je dit soort dingen natuurlijk allemaal mee. 01:05:08-8

M: Met je neus er boven op, hè? 01:05:09-3

M: Ik denk dat het wel bekend is bij een gemiddelde Nederlander. 01:05:15-0

V: Niet zo specifiek als 10 aanbevelingen, maar de dingen die er staan.. 01:05:18-7

Gesprekleidster 1; De boodschap zeg maar is wel bekend, maar zo specifiek, de aanbevelingen, dit komt van het WCRF en dit zijn aanbevelingen ter preventie van kanker, wat zegt u dan? Die heb ik al een keer eerder gezien of niet? 01:05:33-3

M: Nou, niet specifiek van, maar wel algemeen beeld 01:05:36-6

Gesprekleidster 2: En ze lijken op de algemene 01:05:38-4

Gesprekleidster 1: Ja precies, dat klopt inderdaad ook wel. 01:05:40-3

V: Die voorlichting ontbreekt van jou. We worden overspoeld met dit soort dit soort voorlichting. Je kunt

geen krant of tijdschrift openslaan of noem maar op dit krijg je steeds 01:05:53-6

M: De media maakt veel meer kapot natuurlijk, want het ideaal beeld heeft de media, er zijn natuurlijk mensen die super slank zijn en zo is het met heel veel, ook met voeding, hè, daar maakt de media natuurlijk ook heel iedereen gek mee. En want je moet dit eten wat dat is gezond, bijvoorbeeld de suikervrije producten blijkt achteraf dat dat helemaal niet zo gezond zijn, hè? Want je zegt inde automaat. 01:06:16-9

V: Bedoel jij de reclame? 01:06:18-6

M: Ja, de reclames 01:06:20-4

V: Want in de media wordt juist alle onderzoeken gepresenteerd vind ik. Dan denk je, oh, oké, en dire weken laten krijg je een tegen onderzoek. Of het onderzoek blijkt niet te deugen. En dan is het voor niets. 01:06:29-3

Gesprekleidster 2: En daarom is het belangrijk dat het gebundeld wordt, hè? 01:06:35-3

In hoeverre voldoen jullie aan deze aanbevelingen?

Gesprekleidster 1: En dat er naar het algehele plaatje wordt gekeken, van al het onderzoek en niet naar 1 specifiek onderzoek. Maar ja, dat is een beetje hoe het werkt in de media hè, hoe dat net opgepikt wordt. En dan is het belangrijk omdat dan in de context te zien, als er een media bericht komen van dit is heel belangrijk. En dan is het inderdaad wat jij ook zegt, wat is de bron? Waar komt het vandaan? Belangrijk omdat in de gaten te houden. En als jullie zo naar die aanbevelingen kijken op dit moment, zijn er dan dingen van jullie denken op dit moment, of, in hoeverre voldoen jullie hieraan, laat ik dat eerst vragen? Is het zo dat als jullie hier naar kijken van nou.. 01:07:26-1

V: punt 6 ontbreek ik 01:07:28-0

M: Dat hoort ook bij te kort aan ontspanning. 01:07:31-2

V: Ja, dat vind ik ook. 01:07:30-5

M: Daarna moet je uit eten en dan het geeft ook 01:07:34-2

V: 1 glas mag wel, hè? 01:07:38-5

M: Maar hoe groot is dat glas? 01:07:37-4

gelach 01:07:48-2

Gesprekleidster 2: standaard glas

M: Je pakt gewoon een glas wijn, maar hoe groot is dat glas? Hoe groot mag dat zijn? Ja, zegtie gewoon 01:07:56-8

Gesprekleidster 2: Je hebt standaard maten inderdaad. 01:07:58-5

M: Alles met mate, alles waar “te” voor staat is slecht. Maar ja, wat is “te”? 01:08:03-0

M: Ik drink wel alleen water, ik drink nooit ".." 01:08:04-2

V: Hier worden wel goed hierover voorgelicht. 01:08:07-0

Verandering leefstijl:

Gesprekleidster 1: En als je nu kijkt wat hier staat, zijn er dan dingen die je op dit moment zou willen veranderen? Staan hier dingen bij, dat wil ik inderdaad, dat wil ik aanpakken, of juist niet, kan ook? Dat je

zegt van nou, daar wil ik verder helemaal niks mee doen. 01:08:22-3

M: Ik zou wel van het roken af willen. 01:08:26-7

M: Ik zou wel minder willen wegen. 01:08:32-7

Gesprekleidster 1: En hoe is dat voor u? 01:08:33-2

V: Ik leef daar aardig naar. Dat zouten, er zitten veel verborgen zouten in producten daar heb ik niet zo'n zicht op, maar ik moet zeggen dat ze aardig overeenkomen. 01:08:43-3

V: Dat kan je weer voorkomen door kant en klare dingen niet eten, hè? Zelf zouten maken 01:08:49-2

V: precies, ja 01:08:49-0

V: En zelf de soep maken. 01:08:52-7

M: Er staat laag mogelijk gewicht, dat is net wat ik al zeg, op gewicht te blijven dat is voor mij ook van belang. In principe kan ik alles eten, van nou, ik komt toch niet aan. Maar het is gewoon slecht, ik kan het niet verdragen. Dus ik kom ook niet aan, hoor, het blijft gewoon hetzelfde. Dat is gewoon, daar voel je je goed bij. Maar paar kilo's komen er gewoon niet meer bij, dat lukt gewoon niet meer. Dat is gewoon een uitwerking van wat er met de darm is gebeurd. 01:09:26-3

M: Het kan ook aan stress liggen, want sinds dat ik uit de café ben, ben ik tien kilo aangekomen. 01:09:31-4

V: Oh ja, dat zal wel. 01:09:32-7

M: in 1,5 jaar 01:09:33-9

V: ja, maar dan loop jij misschien niet zo veel 01:09:35-6

M: Ja klopt ".." fiets 01:09:42-3

V: het is wel hard werk horeca, ik wel, hoor. 01:09:43-5

M: Maar ik denk ook dat gewicht heel bepalend is door regelmaat. Want dat zegt helemaal niet dat je elke dag om vijf uur moet eten en exact om elf uur naar bed moet gaan, 7 uur op moet staan. Ik denk wel dat een bepaalde regelmaat ik denk dat dat wel belangrijk is. 01:09:55-9

Gesprekleidster 1: Klopt ook.. 01:09:56-9

V: de rust-reinheid regelmaat 01:10:02-5

Gesprekleidster 1: ja, maar zo is het wel. Maar hoe is dat voor jou V2, zou jij iets willen aanpassen als je zo kijkt naar de aanbevelingen? Is er iets wat je zou willen..? 01:10:10-5

V2: Nou, ik ben gestopt met roken, toen kwam het ook in een keer kwam het aan. En als je in de overgang zit, val je niet meer zo snel af. Dus dat is wel lastig, ik zou ook wel minder wegen maar dat gaat niet zo heel makkelijk. 01:10:22-9

Gesprekleidster 1: En voor jou V1, zijn er dingen die jij zou willen aanpassen. Of denk je, ik voldoe er eigenlijk al aan, of ik voldoe er niet aan, maar ik wil het gewoon niet aanpassen. Dat is ook een optie 01:10:33-9

Gelach 01:10:37-4

V1: Nee, ik probeer het wel, de dingen zijn wel bekend, dus het minder zout eten. Wij zijn twee weken terug in Duitsland geweest en denk ik, mijn god, alsof de zoutbus op het eten leeg gestrooid was. En richting de horeca, doe er iets mee, dat is echt ongelofelijk. Want wij koken, ja, zonder zout, niet, wel met pakjes enzo, dat wel, dus dat vind ik wel een goede tip. Dat vind ik wel een goede tip, daar zou ik wel iets mee kunnen doen. 01:11:06-3

V: Van zout kan je een heel eind van verminderen 01:11:07-9

V: ja, ja zeker 01:11:11-0

Gesprekleidster 1: Hoe is dat voor jou, V3? 01:11:13-8

V3: Er zit geen verschil in van hoe ik voorheen deed en nu. Ik denk dat ik 15 jaar geleden wat meer alcohol nam en nu paar in de week. Die laat ik ook niet.. 01:11:26-5

Gesprekleidster 1: Maar is iets wat je zou willen aanpassen nu? Of zeg je van nou nee dit.. 01:11:34-2

V3: Nee, nee 01:11:37-2

Gesprekleidster 1: En heeft dat dan met name als reden dat je dat gewoon goed genoeg is voor je eigen gevoel natuurlijk en niet zozeer. 01:11:45-2

V3: Nou, het heeft niet zo veel invloed, want ik heb een high output stoma, 01:11:48-1 dus wat ik eet en drink dat heeft dan niet zo heel veel invloed. Ja, maar kijk wat is, daar, ik ben niet dik, beweeg goed,.. 01:12:07-0

Gesprekleidster 1: Je voldoet er eigenlijk gewoon aan? 01:12:07-5

V: Ja, het is.. 01:12:11-3

V: En verder is eigenlijk gewoon van, oh, ik ga niet in op de detail, want je wordt er een beetje gek van. 01:12:15-1

M: ja 01:12:17-0

Gesprekleidster 1: Ook niet te veel mee bezig willen zijn? 01:12:21-9

V: Nee, je moet daar zeker in relativeren. Beetje nuchter in blijven, van als hij zegt, ik voel me goed bij die geitenspul. En als jij zegt, ik wil geen melk, dan doe je dat niet en zo simpel is het. Je voelt ook zelf al wat je.. 01:12:41-2

M: Wat je lichaam nodig heeft. 01:12:42-8

V: Ja 01:12:45-0

M: Goed luisteren 01:12:45-0

Gesprekleidster 1: Laten we terug gaan naar het bord, ik heb idee dat ik er, heel erg het idee dat ik er voor zit, klopt dat? Ik heb de neiging om te gaan bukken zo.. 01:12:57-6

Gelach 01:12:58-6

V: Om het spannend te maken 01:12:58-6
01:13:03-4

Discussie (punten die missen):

Gesprekleidster 1: Ja, haha, um, zijn er nog punten die hier missen? Bijvoorbeeld stoppen met roken, zijn er factoren die daar mee te maken hebben we hebben het veel over voeding gehad, zijn er factoren die nog bij de plus of min kunnen die te maken hebben met stoppen met roken bijvoorbeeld? 01:13:18-3

Gesprekleidster 2: Of bewegen 01:13:19-1

Gesprekleidster 1: Of bewegen inderdaad 01:13:22-6

M: stress 01:13:22-6

Gesprekleidster 1: Stress hebben we dat al? 01:13:22-7

M: Na ja, ik heb thuis een vrouw zitten die rookt. Goed, we waren samen gestopt, maar toen waren drie weken van elkaar en zij was 4 kilo aangekomen. Ze zegt van je doet wat je wilt, maar ik ga weer beginnen. Dat is het mij niet waard, ik wil niet.. Ja, nou ja, dan ga je die begint met roken. En dan stabiliseert dat gewicht van haar weer, maar goed dan zit ik weer van. Daardoor gaat het wel fout. 01:13:50-7

Gesprekleidster 1: Ja, oké, dat is heel belangrijk, ja. Het is ook belangrijk dat als je inderdaad wilt stoppen. Het gevolg dat je vanuit je omgeving gesteund wordt ook daarin. En het is heel erg lastig als je partner niet mee stopt, om het zelf vol te blijven houden. Dat is heel erg lastig. Um, zijn er nog punten die hier niet in staan die er nog missen? Die er nog bij moeten? 01:14:25-6

stilte 01:14:30-7

Gesprekleidster 1: We hebben eigenlijk nog niets bij volhouden staan. Wat zijn factoren, nou ja, het volhouden van stoppen met roken, de sociale omgeving hoort eigenlijk ook daar bij het volhouden. Zijn er nog, um, nog andere punten die specifiek volhouden..? 01:14:49-7

M: Positief blijven, ondanks alles ja gelukkig ".." langs mekaar 01:14:55-5. Dat is gewoon een feit. Maar toch positief blijven ondanks alles. 01:14:59-9

M: Ja maar, dat is makkelijk gezegd. Ik denk die knop heel makkelijk omzetten, ik heb zo iets van, ik heb niks, that's it. Je moet niet het kop in het zand steken, hoor. En natuurlijk heb ik iets, maar het heeft geen enkele invloed op mijn leven. Dat heb ik, ik word elk jaar onderzocht. 01:15:17-8

V: Ja maar, dan word je niet tegengehouden door ".." 01:15:21-1

M: Nee, en dan krijg ik weer APK stempel en hup ik kan er een jaar weer tegen. 01:15:22-3

V: ja, precies 01:15:25-0

M: En dan zeg ik, feitelijk is het ook zo, degene die niet worden onderzocht, die hebben meer kans om aan darmkanker te overlijden dan ik. Zo zie ik het, want mij houden ze in de gaten. 01:15:35-6

V: Ik snap wel dat jij die opmerking maakt. Ik ben natuurlijk op 41 jarige leeftijd eierstok, of baarmoeder kwijt geraakt. Dus ik ben vervroegd in de overgang gekomen. Dus ik heb wel degelijk ook fysieke klachten, maar het is ook wat jij ook gezegd. Ik laat me er niet door leiden. Ik heb soms dat ik 's avonds zit en dat ik denk oh ja, dit is weer zo'n moment en dan kom ik tot niets. Dan denk ik, oké, dan ga ik zitten wat ik me voorgenomen heb, ja, daar kom ik dan niet aan toe, morgen is er weer een dag. Dat is het. In hoeveel laat je er uiteindelijk door leiden. Dan denk ik ja. Het is niet anders. 01:16:09-0

M: Dat merk ik ook door de schildklier, schildklier begint ook te spelen daar word je heel erg moe van. Daar geef ik dan maar aan toe. 01:16:17-1

V: Ja, precies 01:16:20-1

M: Maar ik ga niet bij de pakken neer zitten. 01:16:23-2

M: Als wij een familie feestje hebben dan word er altijd over gepraat onder elkaar. Er wordt heel veel gepraat, is ook positief vind ik. En ook ".."daar komt een ".." 01:16:34-1

Gelach 01:16:38-6

Gesprekleidster 1: Dat is op zich wel heel fijn. 01:16:43-5

V: En krijg jij die twee liter nog weg. 01:16:48-4

Gesprekleidster 1: Ik kan me heel goed voorstellen, dat je daar steun aan hebt. 01:16:52-2

M: Ik heb 5 zussen en ".." 01:16:58-4

Gelach

M: Dat moet je ook positief kunnen zien. Je kunt wel zeggen, oh wat erg. Maar net wat je zegt, je kunt ook denken, geluk ik word ".." 01:17:11-4

V: Ik heb een poos geprobeerd om lotgenoten groepen te maken. En dan ook proberen mensen, wat jij met je familie doet, wat ook mee te doen. Maar het is niet van de grond gekomen, hoor. 01:17:26-6

M: Ik vind ook zwaar woord, lotgenoten. Ik zo zie ik niet, hoor. 01:17:32-2

M: Wij hebben het gewoon altijd over ".." 01:17:34-7

Gesprekleidster 2: Weet je een beetje waarom dat niet liep? 01:17:41-0

V: Ik denk dat mensen er ook vaak niet mee bezig willen zijn. 01:17:46-0

V: Ik denk dat dat de belangrijkste oorzaak was, ook in de minder opkomst van de bijeenkomst. Want ze komen drie, vier, keer en dan zie je eigenlijk niet meer. 01:17:58-3

Gesprekleidster 2: Ja, dan neemt het af. 01:18:01-4

Gesprekleidster 1: Dat is ook wat we vaker terug hebben gehoord. 01:18:06-0

V: Want je kunt je ook zien praten, hè? 01:18:09-8

M: Dan horen ze iets en dan overmorgen hebben ze het ook. 01:18:12-5

V: Zeker met dit van als je veel over gaat lezen en bekijkt wat er allemaal bij kan komen bij dit syndroom, ja 01:18:27-3

V: Dat wil je niet weten. 01:18:28-6

V: Je moet heel nuchter zijn en sterk in je schoenen staan, want anders kan je er helemaal beroerd van worden. 01:18:33-3

M: Daarom wou ik dat ook niet weten. 01:18:37-0

M: Nee, maar dat is ook wat de risico's zijn van een onderzoek. Ik doe het wel elke keer weer, maar er zitten best wel risico’s aan, maar dan moet je dat dan niet teveel, dan is het goede en kwade afwegen natuurlijk. 01:18:50-8

V: Maar we hoorden toen straks met de voorlichting, van wat voor voorlichting heb je gekregen en dan moet je tegelijkertijd afvragen welke voorlichting wil ik hebben. 01:18:58-1

Gesprekleidster 1: Dat is een goede vraag. 01:19:03-5

V: En voorlichting over waar je wat invloed op kan hebben zoals voeding enzo en roken die wil ik wel hebben, maar ik maak er niet zo druk over, maar ik zie in mijn familie dat mensen daar helemaal hoteldebotel van geworden zijn, van de informatie waar je aan kunt komen. 01:19:28-9

Gesprekleidster 1: En wat bedoelt u dan precies? 01:19:30-3

V: Wat voor, omdat het ook een erfelijke aandoening is, als jij weet dat en dat en dat kan ik allemaal krijgen. En dan ga je het weer projecteren op je kinderen, dan voel je dat veel harder. Daar moet jij eigenlijk heel erg nuchter blijven, van alles kan. Ik kan een heel extreem voorbeeld noemen, mijn zus heeft dat Lynch syndroom, die heeft preventief, baarmoeder, eierstokken, dikke darm laten verwijderen en haar dochter sterft. Zij moeten eigenlijk alles bijzetten om hiermee om te kunnen gaan. Dus te veel voorlichting kan mensen van zijn stuk brengen, waardoor ze niet gelukkig worden 01:20:31-7

Gesprekleidster 1: Maar wat is dan om even terug te komen op wat voor informatie wil je hebben. Wat voor informatie zou je dan willen hebben? 01:20:42-5

V: Waar je wat aan kunt doen, is voor mij een criterium. Waar ik zelf invloed op zou hebben. Maar waar ik geen invloed op kan hebben van, ja. 01:20:53-0

Gesprekleidster 1: Maar wat is dan een voorbeeld van achteraf van nu, wat is een voorbeeld van informatie wat je achteraf zou gehad willen hebben? Wat je nu weet waarvan je achteraf denkt, dat had ik toen willen horen toen ik de diagnose kreeg, of daarna als u terug komt voor de controle? 01:21:05-9

V: Ik zou dat wel willen weten, wat voor mij een signaal zou moeten zijn, om weer een arts te waarschuwen. 01:21:14-1

Gesprekleidster 1: Dat is heel praktisch inderdaad. 01:21:15-4

V: En dan ook welke arts, want ik hoor die meneer toen straks zeggen over die huidaandoening. Je hebt dus 01:21:21-4

M: schildklier was dat 01:21:21-4

V: ik dacht dat je ook huidaandoening 01:21:26-4

M: Ja, ook huid 01:21:24-5

V: tumoren is dat 01:21:25-8

V: Dat soort dingen van, dat zou ik wel willen, als jij belast bent met een syndroom, van daar even opletten, daar even opletten, daar even opletten. 01:21:41-7

Gesprekleidster 1: Meer een soort van checklist 01:21:42-9

V: Maar ook weer niet in de detail. Want je kunt mensen helemaal hoteldebotel maken, hoor. 01:21:45-7

Gesprekleidster 1: Meer een briefje van als dit, dit en dit aan de hand is, neem dan contact op met die die 01:21:55-4

V: En zorg dan die informatie daarop die ik er heel graag bij zou willen hebben is ook al aan de orde geweest, waar moet ik me dan vervoegen 01:22:00-4

Gesprekleidster 1: Ja, precies, ja 01:22:04-6

V: En niet perifeer ziekenhuis, want daar is vaak veel te weinig van. 01:22:09-6

M: Ik zou ook liever al mijn kwaaltjes hier in 1 ziekenhuis onder willen brengen. En ik denk dat het wel makkelijker de link wordt gelegd. Dan dat ik.. 01:22:23-6

V: Nou.. 01:22:23-6

M: Nee? 01:22:25-0

V: Ligt eraan of die artsen onderling contact hebben, voor jou was het.. 01:22:29-6

V: Ik heb een jaar als verpleegkundige hier gewerkt, ook op de poli met vrouwen met borstkanker en dan kwam er iemand die iets aan de teen had en toen was het van, ja, maar je komt hier voor de borst. En voor die teen moet u eerst naar de huisarts. Het enige voordeel is dat op alle afdelingen dan een dossier is, maar voor de rest maakt dat niet zo heel veel uit. Ja, moet wel zeggen dat is wel 10 jaar geleden, dus ik weet niet wat er nu veranderd is. Maar ja, daar verbaas ik me soms ook wel is over, dat was de routing, heb je iets anders, dan ga je maar weer terug naar de huisarts. 01:23:02-3

V: Ik mail wel vaker dokter ** en overleg ik en dan zegt hij van hoe of wat 01:23:09-1

Gesprekleidster 1: Oké, dat is ook mooi hè, dat is wel fijn. 01:23:11-7

V: Ik kan ook allerlei andere dingen doen 01:23:13-9

Gesprekleidster 1: En hoe is dat voor jou, als jij nu terug kijkt, is er dan informatie die jij gemist hebt, die je gehad zou willen hebben in aansluiting met.. 01:23:23-0

V: Nee, wij hebben DNA onderzoek gehad, mijn broer en ik, we hebben het allebei. En ik ben op de terugweg heel boos geweest op mijn moeder, omdat zij het mij bezorgd had. En na een uur was het ook over. Toen heb ik de knop omgedaan en onderzoek en.. Ik heb drie jongens in de leeftijd van 22 tot 18 en ik doe zo nuchter mogelijk als ik dat onderzoek heb. Ik hoop dat ze vaak niet thuis zijn, als ze wel thuis zijn, ja, dan doe ik heel simpel. Ik ga aan de poets en ik laat hun zien dat het allemaal wel meevalt. Ze komen er ook een keer aan, met onderzoeken komen ze steeds verder, mijn moeder zat hier met haar broers en alles op de gang met een slangetje in haar neus. En schoon te spoelen 01:24:04-8 ja, ze komen steeds verder. En hoe meer je aan deze dingen meedoet, ja, hoe verder dat ze komen. Dat DNA zal tien jaar duren en binnen 5 jaar hadden ze het. Zo is met heel veel dingen. 01:24:21-4

Gesprekleidster 1: Wat betreft kan de ontwikkeling heel snel gaan inderdaad. 01:24:24-7

V: Bij mij hebben ze de baarmoeder ook van te voren eruit gehaald, vanwege dat heel de.. mijn moeder heeft het hele circuit gehad, zeg maar, wat er bij hoort bij dit syndroom. 01:24:35-9

M: Mijn nichtje heeft, had een berichtje gehad, dat ze eventueel in aanmerking zou komen met een spuit. 01:24:43-9

Gesprekleidster 1: vaccinatie 01:24:42-7

M: Daar heeft ze niet aan meegedaan, want ze wil dat even niet. 01:24:46-7

V: Dat is voor baarmoederhalskanker, hè? 01:24:49-1

M: Nee, ze heeft ook preventief verwijderd, maar het was wel allemaal voor het Lynch syndroom. 01:25:01-7

Gesprekleidster 2: Het is alleen nog maar in onderzoeksverband. 01:25:02-9

M: Ze zei, ik weet niet wat dat op langer termijn, wat daarmee gebeurd. Ik weet ook niet wat ik daarbij moet voelen, heeft dat misschien wel bijwerkingen daarna, of is dat wel goed. 01:25:08-4

Gesprekleidster 1: Ja, dat weten we nog niet allemaal 01:25:12-0

M: Maar met syndroom is toch grote kans bij vrouwen bij baarmoederhalskanker ook. Voor mijn zusje inderdaad en bij de man bij de nieren dan. 01:25:24-8

V: Eierstokken, je kunt onderzoeken hebben en die hier tot daar en je hebt het, het is niets. Dat is niet fijn. 01:25:34-7

M: Maar nogmaals, je kunt je kop in het zand steken, maar als je nooit niks aan laat doen, nooit laat onderzoeken dan.. Maar, bij baarmoeder is het onderzoek ook niet. Bij mij halen ze iedere keer weefsel weg en het was altijd goed. Maar toen na de operatie bleek dus eigenlijk dat het niet goed was. Was drie maanden er voor onderzocht, dus het is ook geen garantie dat je niets hebt.. Dus in dat opzicht denk ik van, nou dan.. 01:25:56-5

M: Nou, in ieder geval, wat ik dan hoor, mijn zus is hier die PAP-waarde elke keer te hoog. En dan denk ik, geef dan advies als arts van ja, dat klinkt misschien heel kort door de bocht als man zijnde, ik weet niet hoe het voor de vrouw zou het natuurlijk anders zijn. Ze heeft een leeftijd, ik ben 48 zij is dan 50. Ja, haal je het weg denk ik dan. 01:26:20-4

Gesprekleidster 1: Het is een optie in elk geval. 01:26:19-6

V: Er zijn ook weer onderzoeken, met name met borstkanker, borsten weghalen komt het achter het borstbeen. 01:26:32-2

V: Je moet naar de bron naar mijn gevoel. 01:26:33-9

Gesprekleidster 1: Gesprekleidster 2, hoe is het qua tijd? 01:26:32-0

Gesprekleidster 2: kwart voor. 01:26:32-5

Gesprekleidster 1: oké, laten we maar 01:26:40-3

V: Even, wat ik nou wou zeggen net, bij de dokter als je het hebt dan over voorlichting en voeding. Ik heb iedere keer een coloscopie gehad en toen lag er een boekje van de industrie waarin gerechten stonden die je kunt klaarmaken voordat je een coloscopie krijgt. Dat vind ik dan zo jammer, dat ik dat hier signaleert heb, terwijl de voorbereidingen, het is een ramp. En ik denk van, goh.. 01:27:07-1

Gesprekleidster 1: Waarom net te laat? 01:27:08-4

rumoerig 01:27:21-5

Gesprekleidster 1: Dat is wel fijn dat kunt u nu gebruiken natuurlijk, hè? 01:27:26-7

V: Het waren gerechten die door topkoks waren samengesteld, maar weet je, dan moet je oppassen dat je niet drie gangen maakt, want dan zit je nog vol. 01:27:36-9

V: laten spoelen. 01:27:37-7

V: Ja, dan moet je nog laten spelen, maar het waren hele mooie gerechten. Dan maak je het voor jezelf ook plezierig. 01:27:44-3

V: Dat vind ik heel belangrijk iets. Daar houd ik al dagen rekening mee. 01:27:53-5

M: Van te voren moet je er altijd mee beginnen. 01:27:54-4

Gesprekleidster 1: Zijn er nog andere dingen? 01:27:54-1

V: Zijn jullie lid van de patiëntenvereniging? 01:28:00-3

M: Nee 01:28:02-8

V: Daar komt 4 tot 6 keer per jaar een boekje uit. En daar staat een heleboel in, ook dit soort dingen 01:28:10-1

V: gerechten? 01:28:10-8

V: van alles 01:28:11-4

M: Het is jammer dat zo weinig mensen, en zij als vereniging voor een paar euro per jaar, je steunt wel onze groep, zeg maar 01:28:23-8

V: 17 euro per jaar, dus daar hoef je het ook niet voor te laten. 01:28:28-6

M: Ik heb er nog nooit van gehoord. 01:28:33-5

V: Nu heet het Lynch. Nou, daar word je lid van en krijg je die informatie en dan is elke dag een informatie dag. 01:28:41-5

M: Maar, ze hebben niets gevonden voor die drank, van te voren drinken 01:28:45-3

V: ja wel 01:28:43-7

M: iets algemeens 01:28:44-6

M: maar die werken niet 01:28:46-8

M: heb ik ook niet ".. "01:28:48-5

V: Moet wel veel drinken, maar nou mag je ook calorierijke drankjes drinken 01:28:58-2

3 belangrijkste factoren:

Gesprekleidster 1: Oké, laten we weer heel eventjes naar dit schema. Ik wil eigenlijk ook gaan afronden gezien de tijd. Um, en als er, ter afsluiting wil ik voordat we een rondje gaan maken met wat iedereen nog kwijt wil zou ik graag willen dat jullie even kijken naar dit rijtje. En de drie belangrijkste factoren, kan aan de pluskant of aan de minkant zijn. Die voor jullie het meeste invloed hebben op jullie leefstijl zouden willen noemen. Dat kunnen alle dingen zijn, kan ook iets nieuws zijn misschien staat het er niet bij. Wat zijn nou voor jullie belangrijkste dingen die jullie leefstijl beïnvloeden? 01:29:41-3

V: Dus die voeding, beweging? 01:29:39-7

Gesprekleidster 1: En dan alles inderdaad dus voeding, bewegen, roken maakt niet uit. Gewoon de drie belangrijkste factoren. Is er al iemand die al drie in zijn hoofd heeft toevallig? 01:29:50-0

M: Ja, rustig leven en ontspannen en wat voelt bij, dat het goed is, en dan Carpe Diem.. klaar.. 01:29:57-3

Gesprekleidster 1: Ja perfect, nou hartstikke mooi die is duidelijk. 01:29:59-9

V: dat heb ik ook 01:30:04-7

Gesprekleidster 1: dezelfde drie factoren? 01:30:06-2

V: ja 01:30:09-4

Gesprekleidster 1 :oké, V1, ben jij er al uit? 01:30:13-8

V1: carpe diem, en tijd en keuzes maken 01:30:17-4

M: Iedereen weet wat dat betekent toch? 01:30:19-3

V: Wat? 01:30:19-3

M: carpe diem 01:30:19-3

V: ja 01:30:19-3

M: oké 01:30:19-4

M: bewust leven, en waar jij goed bij voelt, en carpe diem 01:30:36-5

Gesprekleidster 1: nou, dat is een winnaar. 01:30:40-5

V: Ik zou carpe diem wel willen, maar keuzes maken denk ik, ontspannen en bewust leven ook. En dat het goed voelt, carpe diem is voor mij dan ideaal, haha, dat werkt helaas niet. 01:30:58-5

Gesprekleidster 1: V2, wil jij? 01:31:01-2

V2: relativeren, op gewicht moeten blijven staat er, keuzes maken, ja 01:31:09-6

Gesprekleidster 2: relativeren 01:31:12-9

Gesprekleidster 1: en derde heb ik even gemist, wat was het net? 01:31:11-6

V2: keuzes maken 01:31:18-3

M: Nou ja, inderdaad ik heb keuzes maken heel duidelijk, op gewicht blijven, maar dan de andere kant op. 01:31:26-2

V: roken, niet bewegen 01:31:33-1

gelach 01:31:33-6

M: ja, maar goed, dat zijn natuurlijk ook keuzes maken, stoppen met roken. Maar dat zal ik.. 01:31:37-9

Gesprekleidster 2: Dus wat was de derde? 01:31:38-0

M: Ja goed, het inderdaad wel dat ik moet stoppen met roken, maar 01:31:47-0

Gesprekleidster 2: Dat was de sociale invloed dan eigenlijk, hè? 01:31:47-8

Afsluiting

Gesprekleidster 1: oké, dan zou ik willen gaan afsluiten met een rondje waarbij je iets kunt vertellen waarbij je nog iets kunt toevoegen aan dit gesprek, of een vraag wat er nog niet ter sprake is gekomen. Um, wie wil

beginnen? 01:32:20-2

Gesprekleidster 1: V4, heb jij nog iets wilt toevoegen, wilt vragen of een opmerking? 01:32:27-9

M: Nee eigenlijk niet, ik vind het fijn dat ik aan het onderzoek mee mag doen. En ik doe overal aan mee als ik iets te weten krijg, altijd. Want baat het niet, dan schaadt het niet. En voor de rest weet ik eigenlijk ook niet. 01:32:47-4

En ik kijk wel als ik hier dan weg ga, dan zeg ik altijd mocht er iets zijn, iets waarvan u denkt dit is geen goede ontlasting kunt u rechtstreeks bellen dan ga ".." gelijk naar toe. Dat vind ik dus wel vervelend aan. ".." 01:33:10-4

M: Nee, misschien iets meer dat je waar je terecht kunt in plaats van stoppen met roken, ik noem maar iets. Dat je daar misschien iets meer, of dat je handvaten kunt krijgen van, goh, dit zijn de mogelijkheden tot en met roken zijn mensen misschien aan het proberen van gewicht hebben. En als.. 01:33:36-5

Gesprekleidster 2: En wat voor vorm van ondersteuning zou je daar.. 01:33:39-0

M: Nou, dat je een handreiking krijgt van, goh, um, probeer te stoppen, maar goed, dat gaat niet dat ze misschien vanuit de vereniging of van welke hoek dan ook handvaten krijgt, van nou, er zijn cursussen voor of bijeenkomsten. Jullie zitten natuurlijk kort op het vuur, die wel werken. Want ik heb gewoon hulpmiddel nodig hoor, ik kan niet zeggen dadelijk, van ik stop er mee en ik ben er mee klaar. Want je hebt heel veel verschillende vormen: ikstopmetroken.nl, zoiets, accupunctuur.. En je hebt heel veel vormen, maar wat is nou goed? Het zou mij niet uitmaken, al kost 250 euro, ik noem maar wat, want dat heb je in feiten zo terug verdient. Maar ja, er is zoveel. 01:34:31-4

Gesprekleidster 1: Ik heb niet bepaald een tip ofzo 01:34:37-3

M: Nee, dat hoeft nu ook niet, maar voor in de toekomst. Misschien is daar om in te verdiepen van dit werkt wel dit werkt niet. Ik bedoel je hoeft de statieven op na te slaan. En als je op internet gaat zoeken het is allemaal heilig en ze zijn allemaal de beste. 01:34:50-9

Gesprekleidster 1: Ja, precies en voor zoiets is de huisarts een aanspreekpunt, hè, die kan je ook daarbij.. 01:35:01-7

M: Ja.. maar die hebben ook vaak maar 1 of 2 sponsorende dingen. Die indruk heb ik wel. 01:35:07-9

M: Maar het blijft toch, je moet er zelf achter staan. Ik rookte ook twee pakjes per dag. Ben op de 1 na de andere dag gestopt. 01:35:20-4

M: Maar goed, dit gaat niet alleen over roken, maar ook over andere dingen. 01:35:23-0

Gesprekleidster 2: Ja precies, gewoon een overzicht van het aanbod. 01:35:26-0

Gesprekleidster 1: Ja, V1 is er iets 01:35:30-4

V1: Het enige is, ben ik wel nieuwsgierig wanneer we de resultaten van het onderzoek kunnen verwachten. 01:35:38-2

Gesprekleidster 1: Ik denk dat je dan moet denken in termen van maanden. Want we moeten dit gaan uitwerken, we moeten, er gaat best wel wat tijd overheen. Dat uitwerken van deze gesprekken dat kost heel veel tijd. Dat gaan we waarschijnlijk ook niet zelf doen, daar komt iemand voor en die gaat dat dan uitwerken. Dan moeten we de resultaten nog gaan analyseren en dan moet het nog mooi opgeschreven worden. En voor ons is dit niet het enige waar we mee bezig zijn, dus dat speelt dan ook mee. Maar een bepaalde hoeveelheid tijd die er aan kan zitten, maar je moet denken in termen van maanden. Dus over een aantal maanden, dan zal er pas iets op papier staan. En ik denk in welke fase wij dit dan willen doorsturen of

dat het op een moment dat het al bij een tijdschrift ligt bijvoorbeeld, of dat we een eerste opzet hebben of dat we moeten even kijken in welke vorm we dat dan willen, reken er maar op dat het nog even duurt 01:36:32-2

V: Jullie sturen het wel rond dus.   01:36:34-9

Gesprekleidster 1: Ja, we sturen het sowieso toe inderdaad, maar reken er maar op dat het even kan duren. 01:36:43-5

M: Ik eigenlijk niks, maar ik heb wel voor mezelf als ik een jaarlijks onderzoek wat de vorige keer eigenlijk niet zo prettig verlopen was omdat ik geen sedatie heb. En het was de venster die erin zou vallen, want mijn arts was op vakantie, dat wist ik niet. Hij wist eigenlijk niet wat ik had, verkeerde slang erin en hoop pijn gehad dus dat was niet prettig. Nieuw computersysteem weer, het ging allemaal fout, het was heel lang en dat dus nooit meer. Ik heb ze laatst nog gebeld, ik zeg van dit dus niet. Jij weet precies hoe het aansluit van binnen, jij weet hoe je er moet gaan. Ik verwacht van de professor dat hij ook zijn boekje gelezen heeft. Dus ik zei zal de volgende keer maar gewoon een sedatie doen want het gaat niet. Ik zeg, nee, het gaat niet goed bij jou, sorry, met alle respect maar doe het elke keer zonder sedatie, korte stukjes, maar soms is het wel een lijdensweg, 01:37:43-4 weg maar het kan.   01:37:46-9

M: Ik deed dat eerst ook altijd.   01:37:51-3

M: ".."Kort stuk, dan denk ik ja het kan *dokter* zei het kan ook, maar in het begin heb je geen verkleving en naderhand heb je het wel. En je hebt allemaal aansluiting, je moet weten hoe het zit waar je moet kijken. Dus dat was wel heel belangrijk. Dus ik heb nou ook gebeld ik wil alleen bij u zolang u in het ziekenhuis werkt onder jouw behandeling. En als het niet kan, als hij weg is dan houdt het op. Oké, maar zolang u hier bent wil ik weten of dat kan. 01:38:11-2

V: Volgend jaar gaat hij met pensioen. 01:38:17-9

M: Bij het laatste onderzoek werd, goed, mijn partner is daar graag bij, ik vind dat plezierig. Zij vindt dat plezierig. De laatste keer begonnen ze eigenlijk moeilijk doen over privacy. Van mag niet meer en nou goed naar veel ".." mocht ze dan wel mee, want in principe is er niets mis mee om er bij te zijn. Dat vind ik eigenlijk een beetje van nou, goed, als je gaat, als je nou eens in de 20, 30 jaar een keer zo'n onderzoek gaat doen. Want het is inderdaad best belastend. Vind ik wel storend "..' je mag niet mee naar binnen, want het is meneer zijn privacy.   01:38:52-0

Gesprekleidster 1: Ja, dat bepaal je dan gewoon zelf 01:38:54-3

M: Ja maar, dan nog is het van nee, nee. 01:38:56-0

M: Dat mocht ".." ook niet meer. 01:38:58-3

M: Dat ervaar ik wel als zeer vervelend, ja. 01:39:01-0

Gesprekleidster 1: Dat kan ik me voorstellen ja. 01:39:01-4

M: Heb ik ook gevraagd, kan er iemand bij zijn, dan heb ik meer rust. Er zit iemand dan op je buik te duwen van gaat het gaat het nog? En dan mag er niemand mee, zijn, en dan heb ik iets meer steun, maar ik wil niet zeggen dat het helpt maar voor me gevoel hè. 01:39:15-8

M: Als antwoord dat ze meer moeilijkheden hadden met partner nog die erbij stond. Dat die er niet tegen konden 01:39:23-2

V: Dat zou natuurlijk kunnen. 01:39:22-5

V: Oh, op die manier. 01:39:23-5

M: Ik was eerst altijd zonder roes, zal ik maar zeggen, en dan kon ik zelf mee kijken. Ging wel pauzeren, daar niet van. 01:39:38-0

V: Je ziet iets over het hoofd   01:39:40-7

gelach 01:39:40-7

M: Ja, denk het wel, je ziet daar niets aan. 01:39:44-0

M: ik heb al zonder ".." 01:39:46-7

V: Ik hoef dat ook niet. 01:39:49-9

M: Je ziet dan poliepen zitten en dan denk je jeetje wat is dat ding groot? Maar dat is dan het speld knopje. Maar daar maak je dan druk om, je ziet dat zitten en dan zeggen ze het eerst niets hoor, het is maar een spelden knopje, maak jezelf niet ongerust, maar toch, je ziet dat zitten, dat maakt niet uit, je probeert te ontspannen en rustig te liggen, maar je voelt je al ellendig door voorbereiden en laxeren dus ik vind het heftig altijd. Ik val zo 3, 4 kilo af en dan ja, om bij te komen heb je een week nodig. Dat heb ik op het werk ook gezegd, ik blijf een week weg of ik me nou goed voel of niet. Niet iedereen accepteert, maar ja, het zij zo. 01:40:34-6

rumoerig 01:40:35-3

Gesprekleidster 1: V2, heb jij nog iets wat je wilt vertellen, toevoegen, of nog een opmerking? 01:40:38-5

V2: Nee, je hebt eigenlijk het idee dat je van te voren heel mondig moet zijn, van is het een goede slang is, het een goede aansluiting. Dat is allemaal, ga maar even kijken. 01:40:49-1

M: Dat ging 5 jaar goed, alleen deze keer niet, ik ging er van uit dat ik al zo was. 01:40:53-9

V: Ik wil een goede slang en dan zit hij in de wasmachine 01:40:59-5

Gelach 01:41:01-9

V: Nee, doe ik niet meer. Dat leer je dan wel. 01:41:07-7

Gesprekleidster 1: Uit ervaring dan, ja   01:41:10-7

V: verder niks 01:41:10-7

Gesprekleidster 1: V1, heb jij nog ? 01:41:14-6

V1: Nee, eigenlijk niet. Ik merk ik heb niet zo'n behoefte om me inderdaad aan te sluiten bij een vereniging, maar ik vind het wel leuk om met anderen er over te praten en dat heeft met mij ook wel te maken, ik heb geen contact met mijn familie. Ik sta wat dat betreft redelijk geïsoleerd. Dus ik vind het ook wel leuk 01:41:31-8

V: Dat is bij ons ook   01:41:33-3

V: Dat is bij jullie ook 01:41:33-4

V: Ik heb wel contact, maar niemand heeft het erover 01:41:32-5

M: In zo'n ".." wel kunnen steunen en daardoor krijg je wel informatie, je hoeft er niet aan mee te doen. maar je kunt wel steunen dat is wel goed. Je krijgt wel informatie wat er speelt, je weet wel wat er is.   01:41:46-4

V: Ja, ik voel die behoefte niet zo. 01:41:50-0

M: Oké 01:41:48-4

V: Het komt denk ik ook wel omdat ik uit die medisch vakgebied kom. Dat ik.. 01:41:55-5

M: Eigen invulling daaraan 01:41:59-3

V: maar ik vind het wel leuk om gezichten te zien. 01:42:03-3

M: Maar, ik vind het wel prettig dat die vereniging er was. Er is zoveel informatie te halen. En ja, ik heb er echt veel voordelen, meerwaarde. 01:42:10-9

V: Nee, dat geloof ik ook wel. 01:42:14-1

Gesprekleidster 2: Zo staat iedereen er anders in 01:42:14-0

Gesprekleidster 1: Maar, het is goed dat de vereniging er is voor de mensen die er behoefte aan hebben en voor mensen die er geen behoefte aan hebben die doen er helemaal niets mee. Maar het is goed dat het er is. 01:42:22-7

M: En juist omdat het een geselecteerd groepje is, is het wel belangrijk. Dat mensen steunen, je hebt meer zeggenschap, je kunt meerdere dingen doen. 01:42:27-7

V: En ze kunnen voor je belangen optreden 01:42:32-9

Gesprekleidster 1: Ja, dat bereik je met zo'n vereniging.. 01:42:34-8

M: Dagje Efteling is duurder 01:42:37-7

Gelach 01:42:41-6

Gesprekleidster 1: V3, heb jij nog? 01:42:48-9

V3: "Ik zou heel graag namen van artsen en ziekenhuizen hebben van waar je echt ".." kan krijgen. 01:42:53-1

M: Dat is ook belangrijk. 01:42:56-2

V: Dat zou ik echt graag willen dat dat er was. Niet elk ziekenhuis is goed in de aanpak van dit.. 01:43:03-6

M: Dat mis ik dus ook, hè, dat het bekend gemaakt wordt van waar kan ik terecht. Dat is zoeken, want er komt geen steun, want je kunt misschien hier terecht waar heb je goede plaatsen. 01:43:14-0

V: Ik hoorde straks ook iemand zeggen, van ik kon niet in een ziekenhuis terecht, niet in een academisch ziekenhuis terecht. 01:43:24-1

Gesprekleidster 1: ja, ja 01:43:28-9

M:".." helemaal los van, zal ook wel. Maar het gebeurd gewoon niet. 01:43:33-4

Gesprekleidster 1: En waar ben je op het moment onder controle dan? Is het nu dat je ergens anders naartoe wilt dat je ergens onder controle staat nu en je wilt ergens anders naartoe. 01:43:44-0

V: Ik ben in Helmond begonnen, naar Maastricht en toen in Eindhoven, *ZKH5* maar ik heb verschillende plekken gehad, daaruit voort zijn heleboel andere problemen ontstaan, nieren en allemaal dat soort dingen

detail werk. Ik zou ik denk wel als ik de hele loop zie van, dan denk ik op dat moment had een andere dokter een andere beslissing genomen als deze dokter. 01:44:10-4

V: Waarom? 01:44:10-4

V: Omdat die niet genoeg geïnformeerd was of niet genoeg op zijn gebied bezig was. Daarvan heb ik, ik neem ook afscheid van mijn dokters, hoor, als ik denk van deze kan niet veel voor mij betekenen om wat voor reden dan ook. 01:44:30-6

Gesprekleidster 1: Ja, heel goed, u moet zich er wel prettig bij voelen, hè? En dat is heel belangrijk. En dat kon ook in Nederland, je kunt gewoon je eigen ziekenhuis kiezen. Je hebt natuurlijk wel een verwijzing nodig dat dan weer wel. 01:44:52-6

V: de ziektekostenverzekering die heeft contracten afgesloten met de diverse ziekenhuizen. 01:44:56-5

Gesprekleidster 1: Dat is waar, daar ligt het ook nog aan. 01:45:08-0
".."

V: Vertellen jullie nou maar waar ik het beste terecht kan. 01:45:14-3

M: Ja, maar daar zit commercieel belang in. 01:45:13-4

V: Ja, maar ik denk hier in een academisch ziekenhuis, moet je ook niet voor liesbreuk komen want daar hebben ze veel te weinig ervaring in. Daar moet je weer ".." 01:45:20-3

M: Gewoon een huis-en-tuin-keuken dingen 01:45:27-1

V: Daar zou ik wel informatie over willen hebben. 01:45:29-4

M: Dat is wel heel belangrijk. 01:45:31-5

Gesprekleidster 1: Weet jij waar ze, nou ja, hier natuurlijk, hè? 01:45:34-5

Gesprekleidster 2: Ja, maar het hangt maar net af van wat.. 01:45:38-2

M: En het gesprek daar ".." en stamboom van, hoever kan ik terugkijken. Hier, waar komt het vandaan, wat wordt er onderzocht.

V: dat doet Stoet eigenlijk weer 01:45:46-0

M: Stoet heeft dat ook begin, ja, maar, daarna weet je echt niet waar je terecht moet. Ik ga er maar naar toe en het zal wel. De artsen zijn kundig denk je dan. Helaas niet altijd. 01:46:01-7

Gesprekleidster 1: Niet altijd op dat gebied, iedereen heeft zijn eigen specialisme. 01:46:06-2

M: Ze weten ook niet waar ze moeten kijken en de tijd er voor nemen. Je moet niet zomaar iets doen, minstens zo belangrijk. 01:46:13-2

V: Ja, dat vind ik ook, maar dat gebeurt al. Dan denk ik, ja, ik ben heel onnozel begonnen. Ik heb dat gekregen en ik ben naar de dichtstbijzijnde ziekenhuis gegaan. Achteraf gezien nooit moeten doen. 01:46:26-2

Gesprekleidster 1: Dat is iets wat je leert, hè, over de tijd. 01:46:33-9

M: En als je hier was geweest had ze het misschien wel weg kunnen halen, had ik mijn darmen wel gehad, maar dat is als.. 01:46:41-1

V: Er kan zoveel fout gaan. Er is ook heel veel fout gegaan. 01:46:42-3

M: hoe groot wil je dat ".." beter is 01:46:42-8

V: nee, maar als je zelf de keuze hebt kunnen maken, van ik ga daar en daar naar toe en dan gaat het niet helemaal goed, dan is het makkelijker te dragen dan als het een ander voor jou beslist heeft. Je eigen dingen kan je het beter aan. 01:47:02-6

V: ja, ik snap wat jij bedoelt, maar als je zelf kiest en het gaat uiteindelijk mis dan heb je misschien de lasten erbij dat je jezelf verwijt, van ik heb er zelf voor gekozen om daar naar toe te gaan. Dat is dan ook weer de andere kant. Snap je? 01:47:21-1

V: Dat kan, maar ik denk dat zo vaak, het vaakste zo werkt, van ja, ik heb er zelf voor gekozen, ik heb niemand iets te verwijten. Ik heb het zelf gekozen. 01:47:30-9

Gesprekleidster 1: Het is maar net hoe je er naar kijkt, hè, dan. 01:47:37-2

V: Ik zou wel meer over ziekenhuizen willen hebben, wie is waar kundig op welk gebied kundig? 01:47:48-8

Gesprekleidster 1: Dat is niet iets waar ik in ieder geval direct, of ja wij hebben toevallig werken samen met de specialisten hier natuurlijk. 01:48:00-6

Gesprekleidster 2: Dat is wel informatie die we kunnen terugkoppelen als voorkomend onderwerp 01:48:07-9

Gesprekleidster 1: Het is niet iets waar ik 1,2,3 antwoord op kan geven. 01:48:08-5

V: Denk ook niet dat het ooit komt. 01:48:11-3

Gesprekleidster 1: Het zou wel, misschien is dat ook wel weer iets wat binnen zo'n vereniging kan ontstaan. Als je binnen een vereniging bouw je met z'n allen ervaring op bij welke artsen je bent. En wie er waar gespecialiseerd in is, dat soort informatie zou wel binnen zo'n verenging bijvoorbeeld kunnen opstapelen. Dan heb je meer overzicht. 01:48:36-2

V: Dan krijg je informatie van iemand persoonlijk en vanuit zijn ervaring. 01:48:43-8

Gesprekleidster 2: Nou, dat is lastig want iedereen heeft andere ervaringen. Dus zo'n overzicht is sowieso persoonlijk misschien wel. 01:48:50-6

Gesprekleidster 1: Ja, maar het is maar net wat je wil, hè, als je een arts zoekt die echt gespecialiseerd is in het Lynch syndroom, dat is natuurlijk wat meer objectiever, dan heeft niet veel met ervaring te maken dat is gewoon kennis. Dan weet je gewoon net als dokter *professor*, dat weten mensen. Dat soort informatie kan je via een vereniging krijgen, ook weer verspreiden, van iemand die nou ergens te horen krijgt dat die Lynch syndroom drager is ergens in een dorpje in Brabant. Die weet niet van het bestaan van professor * waarschijnlijk. En door zo'n vereniging kan je het wel te weten komen bijvoorbeeld. 01:49:40-3

M: Maar, dat is dan persoonlijk zelf wel, hè, waar wij zelf goed bij voelen, van ik hoor daar thuis. Ik denk dat ik daar meer kans maak of kans begrip en dat ze me daar kunnen helpen. Menswerk, hè? 01:49:58-0

Gesprekleidster 2: Er zit hier een studentencafé onder en ik denk dat daar iets gaande is. 01:50:00-5

Gesprekleidster 1: Dat daar iets georganiseerd wordt. 01:50:01-7

M: Dan kan ik die 2 glazen wel. 01:50:04-2

Gesprekleidster 1: nou wij gaan stoppen, wij willen jullie in ieder geval erg danken voor jullie deelname heel erg fijn dat jullie wilden komen en ik denk dat wij heel waardevol gesprek gehad met veel informatie eruit gekomen. En wij hebben wat foldertjes voor jullie.

Focusgroep 4 Nijmegen 141013 #00:00:00-0#

Persoonlijke informatie/Context
Gesprekleidster 1: …Hoe lang u weet dat u drager bent van het lynch syndroom? En of u in het algemeen bijzonderheden wilt vertellen, of u kanker heeft gehad. Of u geopereerd bent, bijvoorbeeld, naja, wat u daarover kwijt wilt. Zou ik mogen vragen of jij daarmee wilt beginnen? #00:00:21-0#

V1: Mijn naam is V1, ik ben 50 jaar en ik weet sinds tien jaar dat ik het Lynch syndroom heb. Helaas heb ik het ook weer doorgegeven aan mijn dochter, mijn zoon moet nog onderzocht worden. Maar het komt allemaal van mijn vadersfamilie kant af, daar is veel maag en darmkanker in voorgekomen. Gelukkig heb ik zelf nog niet hoeven ervaren om kanker te hebben, dus ik hoop dat ik dat heel lang uit kan stellen. Door gezond te blijven leven, dat was het zo'n beetje. #00:00:53-4#

Gesprekleidster 1: Oké #00:00:56-0#

M1: Mijn naam is M1. Ik ben 51 jaar en ik heb sinds 16 jaar, weet onze familie dat we Lynch syndroom hebben en zit bij ons aan de moederkant. We hebben een hele grote familie, mijn neven die ook al speciale vaccinatie hebben. En ik heb het zelf niet, mijn zus boven mij die heeft acute ".." #00:01:23-8# gehad. Dat is nog een agressieve, die heeft dus een donor lever gekregen. En sindsdien weten wij het in onze familie dat wij het hebben. Mijn zus heeft dus, dikke darm is bij haar weg. En uitzaaiingen waren naar haar borst kant toe. Dus dat is, maar, het is allemaal, door de onderzoeken leeft ze nog. Dus ja.. #00:01:50-7#

V: Hoopgevend, hè? #00:01:57-9#

M: Ja, maar zo is het ook. #00:01:57-2#

V: Ja #00:01:57-2#

Gesprekleidster 1: Dat is ook het voordeel van die regelmatige onderzoeken, hè, dat het ook op tijd.. #00:02:03-0#

V: Het is niet leuk maar het is wel .. #00:02:06-5#

Gesprekleidster 1: Ja, oké dank je wel #00:02:10-9#

V2: Uh ja, ik ben V2, 52 en we weten sinds 2001 dat we Lynch syndroom hebben, zowel ik als mijn beide broers, geërfd van mijn moeder. Die begon op haar 50ste met baarmoederkanker, kreeg op haar 52 darmkanker en is toen overleden. Verder weten we niet veel, omdat zij uit het eerste huwelijk van mijn moeder geboren is. Haar vader is heel vroeg overleden en op enige moment werden we benaderd omdat het in de familie voorkwam. Ik heb haar gegevens ook bij het onderzoek betrokken, mochten ook bij het erfelijkheidsonderzoek, daaruit kwam dus uit dat er sprake was van het Lynch syndroom. Daar hebben we ons alle drie laten testen en alle drie positief, of hoe noem je dat? #00:03:00-3#

Gesprekleidster 1: alle drie drager #00:03:04-2#

V2: En inmiddels heeft mijn jongste broer, volgens mij een jaar of vijf geleden, blaaskanker gehad. En sinds twee of drie jaar moeten we volgens mij ook op nieren en blaas onderzocht.. maar verder heb zelf twee keer een afwijking gehad, maar niet in de vorm van je hebt kanker. #00:03:28-9#

Gesprekleidster 1: Nou dat is fijn. #00:03:31-4#

V3: Ja, mijn verhaal is iets anders. Bij mij is het begonnen 1998. Ja, eerst ik ben V3, ik ben 64, sorry. Um ja, weten het sinds ik denk 2002 van het Lynch syndroom. In '98, um ja, werd ik ziek en na heel veel onderzoeken zijn ze er achter gekomen dat ik een tumor had, want ik kreeg een darm afsluiting. Dus ik werd ik hals over kop 's nachts geopereerd, maar dat is wel mijn redding geweest. Um ja, daar bleek dus uit dat ik dat ik darmkanker had. Um, ik heb nog iets meer dan de helft van mijn darmen, maar daar is heel goed mee

te leven. Dat ja, ik moet zeggen dat ik daar weinig van merk, moet een beetje uitkijken kruidig eten, maar verder valt het mee. Er is van mijn vaderskant toen ik 19 was ja ook een soort kanker gehad maar vroeger werd daar eigenlijk niet zo heel erg over gepraat. Hij heeft in toen hij 40 was ook darmkanker gehad, hij is uiteindelijk gestorven al met 42 toen hij een hersentumor had. Van zijn broers, die moeten het ook hebben gehad, een neef en een nicht van mij die zijn al gestorven aan darmkanker zonder dat ze het eigenlijk wisten. Dus toen is het balletje gaan rollen, ze hebben me benaderd nadat er al vier of vijf mensen van de familie dus, ja, die darmkanker hadden, zou het erfelijk zijn. Ja, zo is eigenlijk het balletje een beetje gaan rollen. Dus ja, het is bij mij nou 16 jaar geleden en ik ben er nog. #00:05:36-9#

V: En u blijft nog even, hè? #00:05:38-2#

V3: Ben ik wel van plan, de controles zijn wel heel veel, maar goed, dan heb ik zoiets van, nou laat maar komen. #00:05:47-7#

Gesprekleidster 1: Het is goed dat ze er zijn, hè? Vervelend maar, hè? #00:05:48-1#

V3: Nou weet je, als je die controle hebt gehad en die is goed dan, ja, ben je zo blij dan, ja, heerlijk, ja, ja. Dat was het. #00:06:05-8#

V4: Ik ben V4, ik ben 65 jaar en "..". De oudste broer van mij heeft het en zo ik heb in '91, zowel de gal als de pancreas ben ik voor geopereerd. Ik heb, in 91 heb ik een gezwel gehad aan m’n pancreas. Ik heb dan in 2006, heb ik nog een urinewegen poliep gehad, hebben ze ook moeten verwijderen. En *stilte* ja, ik ben nou met een onderzoek bezig met immuunsysteem te versterken. En daardoor ben ik ook suikerpatiënt en *hebben* 1/3 meer van alvleesklier *heb*. En ja, mijn dochter die zit dan ook in een onderzoek, maar die heeft verschillende ".." stralingen "..". En mijn oudste broer heeft blaaskanker gehad, geopereerd , dat is nou ook weer goed. Een neef en een nicht die zijn gestorven aan darmkanker. Mijn vader is gestorven aan darmkanker op 42 jarige leeftijd. Um ja, dat is dan. #00:08:00-6#

Gesprekleidster 1: M2 #00:08:00-6#

M2: Ik ben M2, ik ben 60. Hoe lang weet ik het? 10, 12 jaar zo ongeveer, de tijd die gaat altijd harder dan dat je denkt. Daarvoor andere vormen van kanker gehad. Talgklier, zaadbal en waar komt het vandaan? Ik heb geen idee. Ik heb broers en zussen en, maar die laten het allemaal niet bekijken. #00:08:30-9#

Gesprekleidster 1: Dat kan natuurlijk ook. #00:08:34-2#

M2: Ik herken wel bepaalde dingen die in de familie gebeuren, maar dat is allemaal weer op latere leeftijd. Het enige wat ik weet, is dat mijn vader op zeker moment steeds colonscopie moest hebben om onderzocht te worden om de 2 of 3 jaar. Dus um maar hoe lang dat dan was weet ik niet. En waarom dat was daar werd niet over gesproken. #00:08:54-4#

Gesprekleidster 2: Maar het is bij u boven water gekomen toen u zelf.. #00:08:58-8#

M2: Ik heb zelf laten onderzoeken van wat er bij mij aan de hand is. Ja.. en steeds wordt er wel poliepje gevonden, maar dat wil niet zeggen, kan alles zijn. #00:09:19-8#

V: Dus dan ben je jaarlijks aan de beurt? Om onderzocht te worden? #00:09:21-2#

M2: om het jaar. #00:09:23-0#

V: Toch om het jaar? #00:09:23-3#

M2: ja #00:09:24-6#

V: Bij mij hebben ze een keer een poliepje gevonden. Toen moest ik het jaar daarop ook weer.. daar komen. Omdat het net weer erfelijker is bij ons in de familie, want jullie praten ook allemaal over hele jonge leeftijd

dat je vader is overleden. Zoals mijn vader was 28, toen kreeg hij de diagnose darmkanker. En is op 30 jarige leeftijd overleden, dus dat is ook heel jong. Valt me op dat vaders heel jong overlijden allemaal. #00:09:53-9#

M: Dat is mij dus niet zo, maar toch wel het syndroom gevonden 1 of 2. Maar het is om het jaar, dus elk even jaar.

Gesprekleidster 1: Oké, en V5?

V5: Ik ben V5, 58. Um, ik ben drager van het gen dat je op een latere leeftijd kanker krijgt volgens mij. En het is ook als laatste ontdekt dacht ik, um, volgens mij weet ik het ook al een jaar of veertien. Want mijn moeder voor de derde keer darmkanker kreeg of baarmoederkanker had gehad, mijn grootmoeder is ook overleden aan baarmoederkanker, nee, darmkanker. Um, twee tantes en een oom, zussen, broer van mijn moeder en toen hadden ze zoiets van misschien moet je eens kijken, je kinderen vragen of ze geen DNA onderzoek willen doen. En dat was wel raak, mijn twee oudste broers, um, hebben het niet, mijn jongste broer en ik wel. Wij zijn met z'n vieren. Dus het is mooi verdeeld, 50% kans. Alleen ik moet wel zeggen als ze geen vier kinderen had gekregen, was die 50% .. geweest. Ik heb een onderzoek laten doen in *ZKH3*, op een gegeven moment werd het ook in verband gebracht met baarmoederkanker dat was het vermoeden. Dat is toen aangetoond, ik heb dan MSA 6. Dat voor vrouwen juist tijdens hun leven 70% kans op baarmoederkanker kan ontstaan en juist weer minder kans op darmkanker. Voor mijn broer geldt dat natuurlijk niet. Um, toen zijn ze, ik zeg altijd maar, laat je baarmoeder preventief verwijderen dan kan je geen kanker meer krijgen. En toen ik hier in *ZKH5* was, heb ik dat probleem voorgelegd. Ik ben nog gezond en toen zeiden ze, hier: nee, wij screenen gewoon elk jaar. En dat was meteen de eerste keer raak. Toen kon ik alsnog, um, onder het mes. En mijn eierstokken zijn verwijderd. Het was, ja, heel pril allemaal, dus ik heb helemaal niet het gevoel dat ik baarmoederkanker heb gehad. Ik ben geen kankerpatiënt, maar ik weet heel van mijn moeder toen alles uitbrak. Ze heeft ook allemaal overleefd en uiteindelijk is ze aan iets anders gestorven. Uiteindelijk zonder dikke darmen, zonder nier, stukje long eruit, dus ja, ze heeft, ja, heel veel overleefd. En um, ik heb om de twee jaar een colonscopie, maar misschien heeft dat te maken met de aard van het gennetje.

V: Volgens mij is dat normaal, dacht ik, want, wat ik vergeten ben te zeggen, de jongste dochter. Ik heb twee dochters, die heeft ook het gen en die wordt 1 keer in de 2 jaar gescreend. Dus, um en ik, ja, ik heb altijd poliepen, dus bij mij is het, om de 9 maanden, als er heel veel zitten om de 6 maanden, en als soms heb ik ook helemaal niets dan mag er een jaar tussen zitten.

Gesprekleidster 1: Beetje afhankelijk van wat ze vinden.

V: Ja, van wat ze vinden. De laatste keer was een poliepje die konden ze niet pakken, want ze halen ze er meteen uit. Dus nou krijg ik van de week nog een CT-scan voor de zekerheid, hoor. Wat dus helemaal niet zegt dat het kwaadaardig is of wat dan ook maar ik ben wel blij dat ze zo secuur zijn. Hè, dat zeker..

V: Nee, je moet er ook niet nonchalant mee omgaan.

V: Helemaal niet, hoor.

Gesprekleidster 1: Je kan beter te voorzichtig zijn, dan maar een CT-scan extra.

V: Ja

Gesprekleidster 1: Nou,..

M: Alleen de vraag bij ons in de familie wordt gesteld, dat de kinderen gewoon wachten, omdat ze nog steeds niet geregeld is op het krijgen van een hypotheek, het krijgen van een levensverzekering. Dus bij ons wachten de kinderen van broers en zussen.

Gesprekleidster 1: Om zich te laten testen..

M: Om het alleen te laten testen.

Gesprekleidster 2: Maar ze zijn wel onder controle?

M: Ze gaan, de oudste neef die is nu 25 en die gaat nu voor het eerst gewone onderzoek doen, test doen. Ja, en niet om op bloed te laten testen alleen hier ook. Omdat het landelijk niet, um..

V: Dat is het kromme.

M: Begrijpelijk.

V: want dat is eigenlijk niet eerlijk iets

Meerderen: Nee

V: Dan wordt het goed onderzocht en dan krijg je toch geen kans voor elkaar te krijgen met de hypotheek

V: Mijn jongste broer..zei ook van wij hebben minder kans op darmkanker, want ik krijg om de twee jaar colonscopie.

M: Ja, maar het bloed wordt onderzocht, nu ook weer met die vaccinatie .. Nou ja, dichter op het vuur kun je niet zitten.

Gekregen informatie

Gesprekleidster 1: Hé en als jullie terugdenken aan het moment dat jullie te horen kregen dat jullie drager waren van het Lynch syndroom, hebben jullie toen, of wat voor informatie hebben jullie toen gekregen? Hè, wat voor informatie kregen jullie toen op dat moment?

V: Er word je dan gezegd, je moet om het jaar moet je gecontroleerd worden. Als ze niets vinden dan wordt het om het jaar gedaan, vinden ze wel iets dan wordt het ieder jaar gedaan. En dat, ja, van mijn vaderskant was een agressieve daardoor zijn ook heel veel familieleden daaraan overleden. Dus ze willen het risico niet nemen. Dan krijg je als vrouw zijnde ook baarmoederonderzoek bij. Dus dat moet je ook eerst ieder jaar, dat doe ik nu ook om het jaar. En nu schijnt ook dus urineweg moet laten onderzoeken als vrouw zijnde. Als je als vrouw zijnde het Lynch syndroom dan ben je aan de beurt, man is alleen bovengedeelte

Gelach

V: Maar in ieder geval en ik moet ook eerlijk zeggen, psychisch kreeg je best even een klap op je kop. Want, je moet het echt even in laten dalen. Een plekje in je leven gaan geven en je moet er even mee leren omgaan. Als je dan kinderen hebt, dan heb je ook gelijk de angst, oeh, als ik het maar niet doorgeef.

Gesprekleidster 2 En heeft u daar informatie over gekregen, of hulp bij aangeboden?

V: Nee, eigenlijk niet. Ja, je kreeg zo’n boekje en je komt na het onderzoek wel bij een verpleegkundige, daar kun je ook alles voorleggen en je wordt heel goed begeleidt, hoor, als je vragen hebt. Dus, ik ben er positief over hoor hoe het ging. Zit je ergens mee? Dan kun je aan de bel trekken, maar toen kreeg je inderdaad te horen van, nou we gaan dit doen, dat doen en als je vragen hebt, stel ze. En het was duidelijk, ik moet zeggen, ik vond het wel duidelijk allemaal. Alleen voor jezelf moet je het allemaal even plaatsen.

V: ja, ja, zeker

V: Je hebt even het idee van word ik wel oud? Kan ik wel oud worden? En ja, je wordt onderzocht dus we gaan er vanuit dat …

Gesprekleidster 1: En hoe was dat voor jullie? Wat voor informatie hebben jullie gekregen?

V: Ja wij hebben wel vrij veel informatie. We hebben hier in *ZKH5* dat we moesten komen. En er werd ook meteen bij gezegd van dat je hulp kon krijgen. Als je dat zou willen, um, ja, ik vond het verder wel goed. Maar het is net wat jij zegt, van te emotioneel, heel heftig en het ergste vond ik het doorgeven aan mijn dochter. Dat vind ik nog heel moeilijk. Ja, het is heel raar, want dan denk ik ook van, ik neem het mijn vader ook niet kwalijk. Maar toch voel je je schuldig.

Gesprekleidster 1: Het is iets wat je zelf niet in de hand hebt. Je kunt er niets aan doen.

V: Ja, en het brengt ook verandering mee want ook onze jongste dochter die, um, zoals met een kinderwens. Ze is nou 34 en dit geeft wel, is de hoofddoorslag dat ze geen kinderen wilde. Het werkt ook in andere dingen door, hè? Het is niet alleen, ja, en in het begin, ja, een beetje of het zorgeloze leven ben je kwijt.

V: Maar voel je je er schuldig over? Dat je het hebt doorgegeven.

V: Ja, nou, dat zij dan misschien ook de lijden krijgt die ik dan de operatie en alles chemokuren en dat is niet prettig. Je bent een half jaar flink ziek en ja, dat zijn er zijn bepaalde dingen dat je denkt, ik hoop dat je dat nooit mee hoeft te maken. En dan is je liefste bezit..

V: het is allemaal aan het licht gekomen..

V: Ja, wel dat weet ik wel, maar gevoel en verstand dat .. in dit geval..

M: Dat is ook zo, maar uw dochter heeft gezegd dat ze geen kinderen wilt.

V: Ja

M: Omdat ze weet dat ze het ook heeft.

V: Ja, zij wordt ook gescreend 1 keer in de 2 jaar.

M: Mijn kinderen die leven daar helemaal niet mee, die laten zich netjes om het jaar ook controleren. Daar geldt ook wel andere dingen voor, behalve baarmoeder ook de huid. Dus dat moet ieder jaar.

V: Maar jouw kinderen zijn dus ook drager?

M: Dat weten ze niet.

V: Oh, dat weten ze niet, preventief

M: Om een hypotheek aanvraag te maken, dus die..maar die laten het wel netjes doen vanaf hun 25ste en de huid eigenlijk vanaf wanneer dat bekend werd. Dus dat gaat goed, maar even bij het informeren. Wat er werd uitgelegd, ik heb dan PMS2 en wat PMS2 dan inhoudt. De helft vergeet je dan meteen weer. Er werd ook iets over voeding gezegd en over leefstijl. Ten minste dat is tegen mij gezegd.

Gesprekleidster 1: Wat kreeg u daarover te horen?

M: Nou gewoon, gezond bewegen, en doen, ik sport al veel en dat doe ik nog steeds, het is een extra motivatie om qua sporten goed te in te delen. Ik doe al behoorlijk lang met *..* mee. Dat is dan 1 van die dingen waar je het hele jaar voor traint.

Meerderen: ja

Gesprekleidster 1: Ja, dat is niet niks.

V: Dan is je conditie wel goed, denk ik.

Gesprekleidster 1: Nou

M: Maar goed, zo hè, en dan voeding, ik weet dat ik bepaalde dingen niet mag en wat niet mag, is dan wel lekker hè? Dus zo als ik daar werkelijk op ga, dan ben ik een stuk lichter. Um, toch in het achterhoofd probeer ik er wel regelmatig rekening mee te houden en zo ja, wat mag je dan wel en wat mag je dan niet. Daar varieert de informatie ook over, vind ik dus dat is best lastig om dat goed te doen. Daarnaast heb ik een bepaalde vorm van artrose dus dat moet ik het lichaam afslanken, hè? Dus dat ga ik nu, maar dat kan dan weer gunstig zijn voor het andere.

V: En sowieso probeer je je een beetje inderdaad je eetpatroon goed in de gaten te houden. De ene dag bijvoorbeeld zoals met vakanties, dan is het vaak, we pakken even een lekker etentje of we een lekker hapje dan probeer ik de volgende dag vaak weer om een beetje gezond te eten, salade of wat dan ook, dat je vezels binnen krijgen en de producten die je nodigt hebt, dat je die ook binnen krijgen, maar daar zitten ook uitschieters bij, maar dat laat ik me niet ontnemen. Je leeft al daarna, je probeert gezond te leven of gezond je voeding aan te passen maar er mag ook wel een keer een feestje tussen zitten. Anders word je helemaal zo..

M: En wat is voor jou dat feestje?

V: Nou een keer lekker pizzaatje eten.

M: Dat hoeft niet vet te zijn.

V: Eens een keer een frietje nemen, weet je wel, lekker makkelijk, huppekee. Dan heb ik zoiets, volgende dag weer goed op mijn eten letten, nee, dan is het niet de volgende dag een Chineesje of croissantje en noem de hele mikmak maar op. In ieder geval, dan probeer ik wel de volgende dag weer goed te eten.

Gesprekleidster 1: En wat is voor jou een belangrijke motivatie om daar bewust mee bezig te zijn, om met je leefstijl bezig te zijn?

V: Nou, sowieso, om je lichaam goed in conditie te houden en je weet dus dat je darmen, verzorg jij je darm slecht, dan kun je wel de gevolgen daarvan verwachten. Dat is ook de reden dat ik goed op mijn voeding moet letten, ook geen rommel eten.

Gesprekleidster 1: En is dat dan iets waar je informatie over hebt gekregen? Over je leefstijl in relatie tot het Lynch syndroom, of heb je daar nooit…

V: Nee, nee, dat is echt iets van mezelf, ik heb dat zelf, misschien, andere mensen wel, die praten er wel even over, maar om daar uitgebreid informatie over te geven, nee.

Gesprekleidster 1: En hoe is dat voor jou bijvoorbeeld? Heb jij daar informatie over gekregen, over leefstijl in relatie tot het Lynch syndroom?

M: Um, wel een paar keer meegedaan met zo'n onderzoek, vragenlijst vanuit Wageningen en daarvoor, bij ons is het dus iets anders gegaan, wij zijn door het UMC in *ZKH8* getest omdat daar mijn zus met die acute lag en van daaruit zijn wij hier, daar was ** aan het opereren. Die is uit *ZKH5* gekomen, kon ik in *ZKH8* opereren. Daar zijn we in contact gekomen, snel naar *ZKH5* gegaan, toen hebben wij hier een onderzoek gedaan door de hele familie op het bloed. Inmiddels, vijf zijn er positief onderzocht en mijn moeder. Daarna zijn wij na alle onderzoeken waarbij, omdat wij met zo'n grote groep zijn, stuurde ze gelijk die voedingsonderzoeken en allemaal, heb ik wel een paar keer gevraagd, stuur dan nog eens een keer je bevindingen. Is leuk om aan onderzoeken mee te doen, dat is leuk voor jullie, maar wij..

Gesprekleidster 1: Dat duurt soms wat langer, hè, dat die bevindingen er zijn. Zeker als het een studie is waar heel lang doorgevraagd wordt.

M: Nu krijgen we wel zo’n nieuwsbrief waar..

Gesprekleidster 1: Oh mooi, dat is fijn.

M: Omdat wij daar om gevraagd hebben. Nu krijgen we zo’n nieuwsbrief en er staat ook elke keer in voedingsbevindingen, dus die krijgen we..

Gesprekleidster 1: Maar het is buiten, hè, we hebben, buiten het onderzoek waaraan u deelneemt, heeft u nooit informatie gekregen over leefstijl in relatie tot het Lynch syndroom?

V: Nee vind ik niet, wel weer net wat je zegt dat onderzoek van Wageningen..

Gesprekleidster 1: Buiten de onderzoeken, zeg maar, niet binnen het ziekenhuis.

M: We hadden wel hier informatieavonden gehad van meneer ** en als er iets nieuws is, of iets anders is, dan werden wij uitgenodigd. We hebben onszelf ook een paar keer gevraagd, van kunt u iets uitleggen. Binnenkort vragen wij ook, omdat bij ons in de familie ook .. gebeurd zijn. Of we weer een informatie avond kunnen krijgen, voor het uitleg aan de jongeren, zodat die het ook kunnen goed uitgelegd worden door meneer ** die doet dan en die regelt het dan ook dat dat geregeld kan worden ten minste. We hebben net *..* van dat gaat bij ons redelijk

Gesprekleidster 1: Nou interessant, ja mooi, fijn, dat dat gebeurt.

M: Ja, omdat bij ons in de familie heel veel zijn. En ja, die willen nu antwoord en we hebben al een paar dingen gevraagd van die dingen gaan ze vragen, dus houd daar rekening mee. Bijvoorbeeld van wanneer kun jij je laten testen, wanneer wordt het vergoed qua levensstandaard?

Gesprekleidster 2: Als ik het goed begrijp, is dat dus op eigen initiatief krijgt u informatie?

M: Ja, ja, nou ja, um, wij komen hier in verschillende ziekenhuizen, verschillende UMC’s in ** word er eentje getest, er wordt er eentje nog steeds in *ZKH8* getest. Wij komen overal dus dan weet je we horen verschillende dingen. Ja, dan stemmen wij gelijk af, hoe kan dat, daar zeiden ze dat, hoe kan dat? Hoe zit dat? Wat bij mij heel erg was het innemen om het schoonmaken van de darmen, kregen we eerst vier zakjes van die, ik noem het maar rotzooi. Totaal niet lekker.

V: Nee, maar het is lang zo vies niet meer, hoor, er zit iets van citroen.

V: Nee, maar je hoort het veel hoor wat jij zegt.

M: Ja, maar nu hebben ze dus verschillende soorten, we hebben vier verschillende soorten van die klinische gehad en toen had ik ook gezegd. Ik zeg, luister die wil ik hebben en anders ook niet meer, anders krijg ik het gewoon niet weg.

V: Wat was dat?

M: Met die citroensmaak die zij zegt, vier zakjes 4 liter water ideaal.

Gelach

V: Volgens mij ik had er meer, hoor, het heette geen clean prep, meer, maar een andere huppeldepup prep. Ik had wel..

M: Met citroensmaak is dat, het zijn 2 zakjes met 4 liter water, je moet wel 4 liter water drinken, daar kom je niet vanaf.

V: Met een ander zakje erbij, is goed te doen, hoor.

Gesprekleidster 1: Maar goed, even terug naar de vragen. Hoe is dat voor jullie geweest hebben jullie informatie over leefstijl gekregen in relatie tot het Lynch syndroom?

M: Ja

Gesprekleidster 1: Oké, voor jou was het..

M: Ik heb er specifiek om gevraagd, maar ik weet niet meer hoe dat in het gesprek ging. Dat is al zo lang terug.

Gesprekleidster 1: En hoe is dat voor jou?

V: Ja, eigenlijk ook via GeoLynch uit dat onderzoek. Maar zal niet zeggen dat het gelijk mijn leefstijl veranderd heeft. Het is gewoon algemeen, als je hartpatiënt bent, ik noem maar iets, krijg je dezelfde regels. Bewegen, gezonde voeding, veel vis en inderdaad..

Gelach

V: Maar, dat is ook lekker

M: Dat is voor iedereen

Gesprekleidster 1: En hoe is dat voor jou? Heb jij informatie gekregen over leefstijl?

M: Geen informatie gekregen, maar omdat ik ook suikerpatiënt ben geworden, zitten er ..en dan moeten er ook vaker weten en dan, ja, toch maar kleine hoeveelheden, dat eet ik altijd al. .. Dat deed ik al heel veel. Dat deed ik dus al.

Gesprekleidster 1: En u bent eigenlijk ook door die diabetes heel bewust ook met uw voeding bezig, hè?

V: Ja, weet je, zoals in mijn geval heb ik al die kanker gehad en dat geldt voor mij, dan dat ik zelf zo veel mogelijk probeer om gezond te leven, om niet nog weet ik veel wat allemaal nog te krijgen. Dus dat is voor mij..

M: Waar let je dan precies op?

V: Ja, dan dat ik niet te vet ik eet, heel mager en niet te vlees, veel bewegen, ik eet heel veel fruit, heel veel groente. Gelukkig vind ik dat ook allemaal heel lekker, dus het is voor mij geen straf. Maar ja, ik let er wel op.

Gesprekleidster 2: En wanneer heeft u dat veranderd, was dat echt nadat u kanker heeft gehad?

V: Dat is, ja, nadat..

Gesprekleidster 1: Ook doordat u kanker heeft gehad.

V: Ja, en dan heb je zoiets van, nou gezond blijven leven. Ik wil nog wel even mee. Hè, dus, ja..

M: Ja, zoals .. vlees dat eten we helemaal niet meer. En als we barbecueën bijvoorbeeld, dan probeer zoveel mogelijk zelf ook te barbecueën vis te barbecueën, zodat ik ook weet wat .. en niet verbrand. En, um, zulke dingen.

Gesprekleidster 1: Dus je bent ook op een manier bewust met je leefstijl bezig. En dat door het Lynch syndroom hebt ook?

M: Ja, sindsdien is het wel, ja, rustig, laat ik het zo zeggen.

V: Je denkt meer na voordat je het in je mond stopt.

Gelach

M: Nou, dat gaat niet bij elke hap hoor.

V: Ik ben bewust veel minder vlees gaan eten, omdat je gewoon weet dat je darmen daar heel hard voor moeten werken om vlees te verteren. Dat soort dingen meer vis of vegetarisch of iets anders

M: of lamsvlees of kip, kip is ook licht

V: Kip je ook goed doen

M: En lamsvlees, je moet er van houden, hoor.

M: Ik heb dan ook medicijnen die breken eiwitten en vet af .. alvleesklier dat eiwit afbreekt heb ik niet meer. Dus daarom zit ook meer .. en dat komt alleen door het medicijn.

Gesprekleidster 1: En wat voor aanpassingen heeft u dan gemaakt dan in uw leefstijl door die medicijnen?

M: Je moet gewoon na het eten innemen en ja, ik eet gewoon met iedereen mee. #00:34:59-3#

Gesprekleidster 1: U slikt gewoon extra medicijnen.

M: Ik moet wel genoeg eten, anders val ik steeds af. ..

Gesprekleidster 1: Ja, en zijn er dan nog speciale producten die u extra eet? Om uw gewicht op peil te houden?

M: Nee

Gesprekleidster 1: Oké, gewoon ook in grotere hoeveelheden dus eigenlijk wat u eet.

M: Het beste is koolhydraten, niet veel suiker in zit is eigenlijk het beste

Gesprekleidster 1: Heeft u daar dan ook begeleiding bij? Bij zo’n dieet? Gaat u naar een diabetes verpleegkundige? #00:35:47-1#

M: Ja, alleen voor de suiker.

Gesprekleidster 1: Ja, precies dat bedoel ik. Dat heeft u wel in ieder geval dat er iemand met u meekijkt. Dat is mooi. #00:35:55-8#   #00:35:56-7#

Gesprekleidster 1: En hoe is dat voor jou bijvoorbeeld, V5, sorry. Ben je bewust met je leefstijl bezig of ben je bezig met bewust gezond leven zeg maar? Of ben je eigenlijk niet zo mee bezig? #00:36:16-9#

V:'..'   #00:36:17-0#

Gesprekleidster 1: Ja, dat is ook los daarvan. #00:36:17-2#

V:".." #00:36:24-4#

Gesprekleidster 2: En wat verstaat u daaronder? #00:36:24-4#

V: Nou, ik ben 25 jaar vegetariër, dus rood vlees, daar hoef ik niet op te letten. Ik drink gewoon een glaasje wijn, misschien zou ik er beter op moeten letten. Maar, ik ben ook niet, ik ben ook niet gewaarschuwd ofzo. #00:36:53-1#

Gesprekleidster 1: U heeft daar verder geen informatie over gehad?

V: Net wat jij zei, het is allemaal zo lang geleden, wel toen ik de uitslag kreeg van je bent drager. Wel uitgebreid gesprek gehad met de onderzoekster en kon ook met al mijn vragen wel terecht bij de klinisch geneticus. Um, daarna dat is aangeboden als je nog iets wilt weten of hulp nodig hebt, dat ik ben ook een keer hier in het *ZKH5* geweest, bij zo'n voorlichtings wat was het avond of middag. Ik weet het niet meer. Dus ja, dat..

Gesprekleidster 2: speciaal voor Lynch?

V: Ja

V: en verder denk ik als er iets is kan ik hier aankloppen en krijg ik gehoor.

M: Ja, dat is perfect ook. Je hoeft mij maar een mailtje of een belletje te doen en hij beantwoordt.

Gesprekleidster 1: Nou dat is fijn.

V: En ik ben ook een tijdje ben ik lid geweest van hoe heet het, club, Lynch geloof ik. Bij de oprichting, toen dacht ik misschien, wel interessant. Um, na een aantal jaren vond ik de informatie minder worden. En ik dacht, ik houd er mee op. Ik ben ook helemaal niet ziek. Dus ja, zo is het voor mij een beetje. 0:38:10.5

Gesprekleidster 1: oké

V: Ik voel me eigenlijk geen Lynch patiënt.

Gesprekleidster 2: Nee precies, patiënt is niet de goede term.

Gesprekleidster 1: Dat is alleen maar positief, hè? Of in ieder geval fijn dat u geen, niet allerlei dingen heeft meegemaakt al. Of in ieder geval niet in die context, zeg maar.

M: Als ik de andere kant op kijk.. tis ook fijn dat we zo goed onder controle staan, hè? 0:38:54.8 0:39:06.6

Factoren

Gesprekleidster 1: Zo, ik denk dat we, ja dat denk ik ook inderdaad, ja, is denk ik wel het beste. Waar we benieuwd naar zijn, zoals we op het bord kunnen lezen, factoren die de leefstijl beïnvloeden, hè, we hebben het al een beetje gehad over of je bewust al dan niet bewust bezig bent met het leven van een gezonde leefstijl. Dat iedereen weet wel een beetje wat daar onder verstaan wordt natuurlijk, hè? En we hebben zo tussendoor wel wat factoren meegekregen van die van invloed zijn op de leefstijl en die ook kunnen motiveren om een gezonde leefstijl te aan te nemen of niet juist. Dat kan ook. We zijn benieuwd, we willen dit schemaatje gaan uitwerken. Eens kijken, wat zijn nou factoren die jullie leefstijl beïnvloeden? Wat is nou van belang om gezond te leven? Welke dingen zorgen er nou eigenlijk voor dat het niet lukt om gezond te leven, of dat je niet wilt gezond te leven, want dat kan natuurlijk ook.

V: Heeft dat dan over kanker in het algemeen zoals jij zei, als je iets aan je hart hebt, krijg je dezelfde adviezen.

Gesprekleidster 1: Nou, we komen zo meteen nog even terug op de adviezen. Ik zal zo meteen ook iets laten zien over, want er zijn inderdaad ook specifieke, ter preventie van kanker. Daar kom ik zo meteen nog op terug. Dan wil ik graag hier een beginnetje maken van te voren. Um, gesprekleidster 2 is al wat dingetjes aan het opschrijven. En jullie hebben al ergens al gehoord dat jullie ook wel bewust bezig zijn met ook een gezonde levensstijl. En we hebben het ook al wel motivatie gehad en zijn er.. We zouden inderdaad in de

groep willen willen horen, wat is voor jullie de motivatie om bewust met jullie leefstijl bezig te zijn? Van jou heb ik het al gehoord, hè? En wat is voor jou bijvoorbeeld een motivatie om er bewust mee bezig te zijn, met de leefstijl, of juist niet?

M: Nou, een paar dingen maken wel..

Gesprekleidster 1: Wat zegt u?

M: Een paar dingen hadden ze wel gelijk in.

Gesprekleidster 1: Zoals wat?

M: Mijn leefstijl qua eten bijvoorbeeld, om daar meer op te letten.

Gesprekleidster 1: Maar wie had daar gelijk in dan?

M: Nou, we hebben toen zo'n onderzoek gehad. Toen kregen wij zo'n lijst gebruiken voor olie voor bakken, beperkt vlees en meer dat soort dingen.. 0:42:08.1

Gesprekleidster 1: U heeft een vragenlijst ingevuld en aan de hand van die vragenlijst heeft u adviezen gekregen om uw leefstijl aan te passen?

M: Ja

Gesprekleidster 1: Oké, was dat vanuit de Lynch studie ook? Ook was dat vanuit een andere studie?

M: Het kwam volgens mij uit Wageningen. Dan bij de eerste keer weet ik niet of dat uit Wageningen was, maar de tweede keer weet ik zeker, want de derde keer nog een naslag gehad kregen elke keer een nieuwsbrief.

Gesprekleidster 1: Ja, en toen u die adviezen kreeg, toen dacht u inderdaad misschien moet ik nu toch gaan overnemen?

M: Nou ja, en dan krijg je, ik werk dan zelf in ploegendienst en toen kregen we, een voedingsconsulist kwam voor ons staan van wat de beste voeding was voor ploegenmensen. En toen kwam daar grotendeels uit dat en dat en dat, dat weet ik wel, dat weet ik wel. Ja, en dan komt het uit van ja, cholesterol als je hartpatiënt bent, dan krijg je dat en dat te horen. ".."0:43:27.8 merendeels was het cholesterol te hoog. 0:43:33.3 En nou word elk jaar wel daarop getest en suiker en ook dat soort dingen, dus ik vind dat prima.

V: Oh sorry, eigenlijk zouden alle mensen inderdaad met het Lynch syndroom zo'n schemaatje of boekje moeten krijgen, foldertje van, wat kunnen wij nu eigenlijk het allerbeste eten. Ook vlees bakken in olie zei je, dat, nou..

M: ja

V: Dat dat beter is dan bak en braden ofzo. Nee, maar ik bedoel maar, dat wist ik ook niet.

Gesprekleidster 1: Toevallig gaat u zo'n foldertje meekrijgen aan het einde van het gesprek.

Gelach

V: Dat is vlug, snelle reactie.

V: Dan denk ik ja, je denkt dat je gezond leeft, maar wie weet zijn er toch nog wat tips van wat je zegt, hé, oh, dat is mooi.

Gesprekleidster 1: En hoe is dat voor jou, V5, wat motiveert jou om al dan niet bewust met je leefstijl bezig te zijn?

V5: Mijn leeftijd, en we zitten erop. Dus veel bewegen, proberen om in je spaarzame tijd die overblijft te bewegen. En ja, wat ik al zei, mijn man heeft vorig jaar een hartaanval gehad. En dan kom je in zo'n circuit terecht en dan ga je toch anders naar voeding kijken, nogmaals niet vanuit het Lynch syndroom.

Gesprekleidster 1: Niet vanuit het Lynch syndroom, oké. En hoe is dat voor jou, V3?

V3: Um, ja..

Gesprekleidster 1: Nou ja, we hebben het eigenlijk ook al wel een beetje over gehad, hè? Want u zei eigenlijk van, sinds dat ik de diagnose heb gekregen, toen ben ik heel bewust bezig met mijn leefstijl, hè?

V3: Het is wel zo, wat ik er zelf aan kan doen, probeer ik te doen. Voor de rest kun je daar gewoon niets mee. Maar ik probeer het wel. Wat ik kan, dat doe ik.

Gesprekleidster 2: En wat doet u dan bijvoorbeeld?

V3: Um, nou ja, bewust gezond eten, hè, wat ik zei, veel groenten, fruit toch ook wel, want ik ben echt heel erg gek op pizza. Maar, ik doe het nog maar zelden, want het is al eigenlijk of zo erg, maar, omdat ik al wel eens vet eet, eens een beetje vet, dat ik dan zoiets heb, oh, had ik het nou maar niet gedaan, weet je wel. Dat is toch ook, je moet ook kunnen genieten en ik neem ook 's avonds mijn glaasje wijn, heerlijk hè? Maar ik was ook gek op chips, maar vind ik nog steeds lekker, maar dat neem ik niet meer, ik neem dan nootjes, hè, amandelen, hè, dat soort dingen, je vervangt het gewoon voor dingen die ook lekker zijn. Op een gegeven moment, wordt dat ook lekker.

Gesprekleidster 1: Raak je daaraan gewend misschien.

V3: Ja en dan ook heel af en toe, wat ik ook heerlijk vind een kroketje en dan 1 keer in de zoveel maanden. Dan ga ik er helemaal voor zitten, heerlijk. Even dat momentje, haha, en dan heb ik ook voor maanden genoeg van.

V: Voor een kroketje. Wat dat wel niet kan doen. 0:46:54.0

Gesprekleidster 1: Hoe is dat voor jou, M2, wat motiveert jou om al dan niet zo bewust met je gezondheid bezig te zijn? Want je bent heel actief, hè?

M2: Ja, op een geven moment is het gewoon lekker ook om te doen, actief zijn. En ik ben altijd al actief.

Gesprekleidster 1: Het motiveert je weer door het te doen.

M2: Ja, dat geeft je.. als ik naar mijn werk fiets, voel ik me frisser dan als ik aankom met de auto. Dus dat voelt ook zo. Daarentegen, ik ben gek op Indonesisch eten, dat is natuurlijk niet altijd even goed. Dan is het moeilijk om dat te laten staan.

V: Ja, ik neem aan dat wij niet zullen roken.

M: Nee

V: Nee, roken dat doen we nou niet, of overmatig drinken doen we ook niet, dus dan als je dan een keer iets eet waar je helemaal gek van bent, dan moet dat kunnen, hoor. Ja, dan denk ik, dit moet niet je leven gaan beïnvloeden.

V: Niet beheerst

V: Je moet niet in de gedacht van het Lynch syndroom gaan leven.

M: Nee, het is wel zo, als ik overmatig vet heb, dan heb ik er meteen last van, terwijl ik het zelf niet eens in de gaten heb, dat het zo overmatig vet was.

V: En kruiden?

M: Kruiden, heb ik bijna geen last van. Nee, alleen dat het tijdelijk even wat meer vocht vast houdt. Dat er veel zout zit hè, dus dat, maar verder eigenlijk niet. Maar vet hecht meteen, is ook goed fout.

Gesprekleidster 1: Dat is ook meteen een goede motivatie om dat gewoon te beperken, dat vet, want als u er last van krijgt, u zegt..

M: Ja, ik merk het in eerste instantie helemaal niet. In bepaalde salades waar het gewoon, ja in zit, ja dan is het al te laat. Als je er een paar happen van hebt genomen. En omdat je zelf ook niet meer zo vet eet, hebben we in onze familie dan ook last van.

V: Mag ik vragen, merken jullie dat dan ook ontlasting of zeg je je krijgt geen winderigheid of diarree?

M: Gewoon diarree en gelijk helemaal ziek zijn

V: Oké

M: Dan hebben wij hebben bijna en dan 1 keer in het jaar wordt bij ons dus eerst worst gegeten met oud en.. Die wordt gewoon um, maar nu wordt er rekening mee gehouden, alleen nu wordt gewoon niet te vet, ja, gebruikt, ja, maar, gewoon beperkt allemaal. Tegen mijn kinderen zei ik ook van, ja, gebruik geen jus, doe alleen het worst, dan gaat het goed. En dan houden ze alles netjes binnen en dan gaat het goed. En winderigheid en dat soort dingen wel hoor.

V: krijg je wel last van?

M: Ja, maar dat krijg ik ook als ik knoflook en dat soort dingen eet. 0:49:57.9

V: Nee dat is zo, spruitjes ook zoiets. Bepaalde gerechten, dan is het gelijk, volgens mij zijn wij sneller met het darm verwerken dan andere mensen heb ik het idee. Dat wij gauw last van gassen krijgen dan andere mensen die iets eten.

M: Mijn vrouw vindt het altijd vreselijk als ik een windje laat en dat zeg.

Gelach

M: Die gelooft dat gewoon niet.

V: Het valt mij op

M: Maar zij is vaker bij de onderzoeken erbij?

M: um, als ik een scopie heb, ofzo? Nee, in het begin is ze wel eens mee geweest, maar op een gegeven moment heb je het allemaal wel gezien. Dat is ook niet verstandig als dat ze vragen stellen en je hebt een roesje gehad en je weet niet meer wat je gezegd hebt. 0:50:52.4

Gelach

M: Eens een keer dat ik zoiets zei over zo'n ".." 0:51:09.1 en toen zei ze bedankt voor de informatie over de directie van het ziekenhuis. 0:51:14.7

Ontbrekende factoren

Gesprekleidster 1: Oké, laten we eventjes naar het lijstje gaan intussen zijn er al best wel veel factoren naar boven gekomen, die op zowel een positieve manier dan wel negatieve manier de leefstijl beïnvloeden of factoren beïnvloeden dat je juist gezonder gaat leven of die je motiveren om gezond te gaan leven. Er werd goed voelen genoemd, goed voor de gezondheid, goed voor de darmen zorgen en het hebben diabetes dat zorgt er ook voor dat je op je leefstijl let. Um, ontbreken daar dan nog dingen in dat lijstje? Als je zo naar het lijstje kijkt? Zit ik er voor, trouwens?

V: nee, hoor

V: stress

M: Ja, dat is een goede

Gesprekleidster 2: En aan welke kant zou je dat willen zetten?

V: Ik denk dat stress slecht is."..''0:52:19.2
En als je dan ergens kankercellen hebt, misschien hebben we het allemaal wel bij ons. Dat het sneller kanker ontwikkelt.

Gesprekleidster 2: Ziet u stress ook in relatie met leven, dus heeft stress invloed heeft op uw leefstijl?

M: Gehaast, geen tijd voor het eten, geen tijd nergens meer voor. Alles moet snel, snel, snel..

V".."0:52:57.2

V: en niet goed eten dan, hè, dat je met die haast, haast, snel, snel dingen pakken 0:53:03.3

Gesprekleidster 1: Dat je juist meer geneigd bent om ongezondere dingen te pakken misschien, hè, of als.. Kan ook als een soort van beloning. Dat je dan extra vaak denkt, ik mag wel die pizza pakken, want ik ben al zo gestrest.

M: Voor je gemoedsrust, even goed zoiets

Gesprekleidster 1: Ja

M: Maar ook dat, goed kauwen enzo, dat zit er dan ook in. Dat gaat zo snel met elkaar gepaard.

Gesprekleidster 1: Dus haastig eten misschien

V: Dat is wel dat je gebit dus goed functioneert, zeg maar, dat komt er dan ook weer bij kijken.

V: Nou ja, ik moet zeggen dat ik stress bij het eten, ook al eet ik alleen. Ik dek altijd de tafel, ik zet alles neer. Ik neem altijd ’s morgens, ook al moet ik werken, dan sta ik gewoon eerder op. Ik ga rustig eten. Dat is voor mij gewoon, begin van de dag dan gaat alles fout als ik moet haasten, daar heb ik geen zin in. Ja, daar sta ik eerder voor op.

Gesprekleidster 1: Dus dat is dan eigenlijk een gewoonte, hè?

V: Daar heb ik echt aan gewerkt. Mijn man die staat regelmatig, aan het aanrecht staat hij te eten. En als we dan inderdaad een keer haast hebben. Ik vind het gewoon niet fijn. Ik wil gewoon lekker en ik ga ook niet erbij lezen of telefoon kijken of wat dan ook. Gewoon ja, bewust eten.

Gesprekleidster 1: Bewust ontbijten, ja.

V: Ja, maar ook lunchen, hoor. ’S avonds, als het eventjes kan.

V: Ik hoorde ook van dat het heel goed is om de hele dag kleine hoeveelheden te eten. En dat ook minder van je maag last en het is ook voor de darmen minder belastend. En je houdt je stofwisseling goed op gang. 0:55:00.2

M: 's morgens aan, het begint met goed eten, dan komt dat op gang. 0:55:11.4 En er zijn een heleboel mensen die 's morgens helemaal niets eten en maar 1 keer per dag eten.

V: Oeh, dat is heel slecht.

M: Ja, dat zijn er heel veel.

Gesprekleidster 2: Regelmatig,

Gesprekleidster 1: uit gewoonte, regelmaat hè?

V: En dan kleine hoeveelheden, geen massaal, zeg maar.

Gesprekleidster 1: En zijn er nou ook nog specifieke *..* die er voor zorgen dat je het volhoudt om gezond te leven. Om te blijven bewegen, om gezond te blijven bijvoorbeeld.

Gesprekleidster 2: Ja, we hebben het voornamelijk over voeding wat ".."0:55:49.1 Maar beweging is natuurlijk ook een factor.

V: Ja, je voelt je er lekkerder bij, vind ik heerlijk om lekker te wandelen en sowieso om buiten te zijn.

Gesprekleidster 1: En je er prettig bij voelen, zei je net ook al, hè, als je eenmaal beweegt, is het ook prettig om te bewegen en dat zorgt er dan ook voor dat je weer nog een keer gaat bewegen. Dus dat volhouden is ook het fijn vinden.

M: ".."0:56:22.3

Gesprekleidster 1: Ja, nou dat is inderdaad heel belangrijk.

M: Ik heb altijd op de bouw gezeten en dat is wel het actieve buiten hard werken en dan ben je al veel in beweging 0:56:31.5 als iemand die op het kantoor zit. Die komt daaraan te kort.

Gesprekleidster 1: Het is eigenlijk, het werk kan zowel positief als negatief zijn, hè, want jij zei eerder, zittend beroep is voor een, hè, dat speelt mee, dat ik daarom juist weer meer wil bewegen, omdat ik vaak zit en bij jou is het dan juist, nou ja, goed je beweegt eigenlijk al, bewoog eigenlijk al heel veel tijdens het werken, hè?

Gesprekleidster 1: Zijn er nog andere factoren die in het lijstje missen of die juist bij het volhouden nog kunnen? Eigenlijk iedere keer een heel kort lijstje volhouden..

Gesprekleidster 2: Die overlappen die elkaar

Gesprekleidster 1 : Ja, precies

V: Nou ontspanning, zoals na de sauna gaan. Als dat kan, of schoonheidsspecialiste of allemaal dat soort, jezelf even helemaal lekker opknappen.

M: Het kan ook zijn gewoon een krant lezen in rust.

V: Of een boek lezen

V: Of lekker gaan liggen

Gelach

V: Zo'n powernapje

Gesprekleidster 2: beweging genoeg

WCRF Leefstijlaanbevelingen

Gesprekleidster 1: Nou, laten we maar naar de leefstijl aanbevelingen gaan. Nou, hè, ik weet niet of jullie ervan op de hoogte zijn, maar er zijn, is meteen ook de eerste vraag eigenlijk. Er zijn namelijk leefstijl aanbevelingen ter preventie van kanker, dit zijn algemene leefstijl aanbevelingen ter preventie van kanker en die zijn opgeschreven door het WCRF, World Cancer Research Fund, heet het, internationaal. Zijn jullie daar bekend mee, met die leefstijlaanbevelingen? Hebben jullie daar wel eenss van gehoord, überhaupt, van het fonds en deze leefstijl aanbevelingen? Ik zie jullie een beetje ja knikken allemaal.

M: Maar dat is meer in de algemeenheid, hè?

Gesprekleidster 2: Nou dat..

M: Niet specifiek voor het Lynch syndroom

Gesprekleidster 1: Het lijkt.. nee het is niet specifiek voor Lynch inderdaad

M: Dus gewoon algemeen

Gesprekleidster 1: Ter preventie van kanker wel inderdaad en die lijken zoals u gezien heeft heel veel op..

M: Bijvoorbeeld die taille is van niet zo lang volgens mij

Gesprekleidster 1: Die slanke taille, ja, nee, die hoort ook wel bij, hè..

V: Als je een dikke buik hebt ofzo

M: Ja, mannen hebben meer buikvet 0:59:20.3

V: Die is heel lang geleden

V: Nou

M: Die zitten meer te vetten

Gesprekleidster 1: Wat zegt u?

M: Die slanke taille heeft ook meer met de vet te maken

Gesprekleidster 1: Ja, die heeft daar ook mee te maken inderdaad, met de vetverdeling over het lichaam heeft dat ermee te maken inderdaad. Want veel vet rondom de buik is niet zo gezond. En dat wordt in verband gebracht met een verhoogd risico op hart en vaatziekten, suikerziekte, en dus ook met kanker.

V: Ja, maar weet je, dat vind ik dan weer zo oneerlijk eigenlijk, als je wat ouder wordt, dan gaat dus alles rond je taille zitten.

Gesprekleidster 1: Dat kopt

V: Hè, ik zeg altijd alles gaat zakken en blijft hier hangen, ja

Gesprekleidster 1: Maar, 1 van de belangrijkste risicofactoren voor het krijgen van kanker is ook leeftijd. Hoe ouder je wordt hoe meer kans je daarop krijgt. Dat heeft ook daarmee te maken. Laten we ze eventjes doornemen, de eerste zorg voor een zo laag mogelijk gewicht en een slanke taille en vermijd ondergewicht. Dat sluit eigenlijk ook aan bij preventie van hart en vaatziekten. Probeer niet, geen overgewicht te hebben, probeer een normaal gewicht te hebben. Beweeg iedere dag minimaal 30 minuten, beweegnorm en dat is eigenlijk de algemene norm eigenlijk ook. Los van sporten en dat soort dingen. Eet minder calorierijk en weinig suikerrijke dranken. En dat heeft inderdaad ook weer te maken met als je veel energierijk voedsel eet dat je daar juist weer van aan gaat komen. Dat slaat dan weer op de eerste aanbeveling. Eet veel groente en fruit. En dat is voor verschillende kankersoorten aangetoond, dat een hoge consumptie van groente en fruit, dat dat leidt tot een verlaging in het risico op kanker, we weten alleen niet zo goed welk stofje daarmee te maken heeft. Daarom wordt er gezegd, varieer zo veel mogelijk dat je dat stofje op een of andere manier binnen krijgt. Volkoren producten, peulvruchten zit in vezels, die zijn ook belangrijk, zitten ook in groente en fruit. Minder rood vlees en geen bewerkt vlees, met rood vlees wordt dan geen rauw vlees bedoeld maar vlees wat er rood uitziet als het al rauw is.

V: Ook vleeswaren enzo?

Gesprekleidster 1: Met bewerkt vlees, dat slaat dan ook inderdaad op bewerkte vleeswaren, waar allerlei toevoegingen inzitten, zodat het langer houdbaar is. Um

M: Is wel flauw hoor, als ik een stuk Ossenworst neem. Dat is rauw, hè.. Daar heb ik eigenlijk nooit bij stil gestaan. 1:02:03.2

V: Jammer, hè?

Gesprekleidster 1: Het is eigenlijk best veel als je erover na gaat denken, dan omvat dat best wel veel.

M: Maar verschillende soorten ham weer niet.

Gesprekleidster 1: ja, dat ligt eraan of het al inderdaad..

M: Als het gerookt is, als het gewoon natuurlijk gerookt is dan kun je het wel goed eten.

Gesprekleidster 1: Oké

M: Maar als het, ja, ze hebben toen een keer kreeg ik ook toen alle dingen zo zoals rosbief weer niet en gerookte ham op de oude manier, dat was gewoon, dat was wel weer goed. En andere dingen niet zoals Parmaham enzo en die gedroogde ham dat kon wel, maar alles beperkt natuurlijk.

M: Nee, alles waar "te" voor staat..

M: Maar ja, het varieert ook weer, want nu hadden ze het ook weer over de spinazie enzo, dat er maar absoluut 1 keer in de week met nitraat had dat weer te maken. En nu was het weer zo van, laat dat maar los, want aan de andere kant is het veel beter..

Gesprekleidster 1: Ja, verder liever geen alcohol. En dat heeft te maken als je puur, ik zal zo meteen ook even ingaan op de achtergrond van deze leefstijl aanbevelingen, dat stukje heb ik net even overgeslagen. Um, als je puur kijkt naar de literatuur die in de wetenschap is, over alcohol in relatie tot het ontstaan van kanker, dan zie je dat het beter zou zijn om helemaal geen alcohol te drinken, maar omdat voor hart en vaatziekten juist de aanbeveling is om juist 1 glas bijvoorbeeld voor vrouwen maximaal en 2 voor mannen maximaal te nemen, hebben ze de grens daarop gelegd. Je kunt niet voor iedere ziekte nieuwe aanbevelingen gaan uitgeven, dan is het weer goed voor het 1 en niet goed voor het ander, dus daarom hebben ze die literatuur ook meegenomen in de aanbevelingen, hè, wat minder zout en aanbeveling 8 is eigenlijk niet echt een aanbeveling, maar dat is meer een bevinding dat niet bewezen is dat voedingssupplementen bijdragen aan het voorkomen van kanker, um, en natuurlijk niet roken. Er zijn nog twee andere aanbevelingen trouwens, die zijn voor mensen die ooit de diagnose kanker hebben gekregen

de aanbeveling is om die aanbevelingen te volgen. Dat geldt eigenlijk ter preventie van kanker, maar ook, ja, voor mensen die de diagnose al hebben gehad. Om ook aan de aanbevelingen te voldoen. Um en er gaat een aanbeveling ook over borstvoeding. Dat komt omdat uit de literatuur bekend is dat het krijgen van borstvoeding en het geven van borstvoeding beide eigenlijk preventief werken. Nou iets over nog de achtergrond van die aanbevelingen. Bij het WCRF zijn een hele hoop mensen werkzaam die werken namens het WCRF en die houden dus eigenlijk de wetenschappelijke literatuur bij. Op het gebied van leefstijl en het ontstaan van kanker. En zij maken dan zogenaamde systematische reviews en meta analyses waarbij ze, ja, de hele literatuur doorzoeken op onderzoek wat daarover gaat en dat vatten ze samen en ze maken een soort samenvatting van de effecten die ze vinden en op basis daarvan concluderen ze in hoeverre er bewijs is dat er een verband is tussen een bepaalde leefstijlgedraging en het ontstaan van kanker. En op basis daarvan zijn er natuurlijk heel veel leefstijl gedragingen, bijvoorbeeld ook melkproducten zijn onderzocht en voedingssupplementen en zijn allerlei gedragingen die mee zijn genomen en op basis van die literatuur worden er leefstijlaanbevelingen gedaan. En dit zijn leefstijlaanbevelingen die eruit komen en zoals al gezegd komen die eigenlijk best wel overeen met de aanbevelingen die er ook zijn ter preventie van hart en vaatziekten. Eigenlijk is de enige uitzondering minder rood vlees en geen bewerkt vlees, geldt niet voor hart en vaatziekten en..

Gesprekleidster 2: Nee, en het is denk ik, het zijn de deze aanbevelingen, ze doen alleen aanbevelingen op factoren waar zoveel onderzoek naar is gedaan en dat ze echt met redelijke zekerheid kunnen zeggen dat het ook echt zo is. Er wordt ook onderzoek gedaan naar andere dingen, maar daar is nog niet voldoende over bekend om echt aanbevelingen over te doen.

Gesprekleidster 1: Het is niet zo dat dit het nu is. De wetenschap blijft altijd in beweging, hè, dus er komen steeds weer nieuwe ontdekkingen bij en dat is ook het mooie wat, hetgeen wat nu loopt bij het WCRF dat mensen zich daarmee bezig houden, want er wordt ook steeds weer een update gemaakt. Er komt steeds weer een nieuwe versie uit van die, we zijn toevallig allebei pas geleden geweest waarbij die resultaten ook weer gepresenteerd werden dat wordt allemaal bijgehouden. Dat die inzichten, die ja ".."1:07:22.6

V: Wanneer is daar meer over bekend dan? Dit lijstje ik neem aan dat we, die kennen volgens mij allemaal. Je hebt ook zo'n discussie over volle of halfvolle producten.1:07:35.3 Nu, wordt er weer gezegd, nou doe maar niet. Je haalt iets uit een product wat er eigenlijk in hoort te zitten. Halvarine of boter? Wel, niet? Dat soort dingen vind ik eigenlijk veel minder consequente informatie over, terwijl ik denk dat, dat, ja, nou ja, dit lijstje kunnen we allemaal we blindelings opzeggen. Er zijn veel factoren die, waar je van op de hoogte zou moeten zijn, denk ik.

Gesprekleidster 1: Wat dan bijvoorbeeld?

V: Nou, halfvolle producten

Gesprekleidster 1: Nou, wat belangrijk hierbij is om te zeggen is dat dit wordt ondersteund door de wetenschap, dit is op basis van de wetenschappelijke literatuur. Dat aangetoond is dat dit een effect heeft. En halfvolle melk, volle melk daar is geen wetenschappelijk bewijs voor.

Gesprekleidster 2: Nou, er zijn verschillende studies maar dat spreekt elkaar tegen en daardoor kan er geen duidelijke uitspraak over gedaan worden. Dat is, in de media wordt er bijvoorbeeld..

V: ".."1:08:33.8 meerdere berichten gehad

Gesprekleidster 1: Ja, goed

V: Dat is ook met light producten, dat schijnt ook weer niet goed te zijn.

M: Ja, ik mag alleen maar light drinken, en een paar jaar terug was daar een hele taboe, dat daar aspartaam in zit. Maar dat is, nou hebben ze wel bewezen dat dat helemaal niet meer van toepassing is. Dat light drinken kan nu helemaal nergens geen kwaad aan. 1:09:09.0

V: Oké

Gesprekleidster 1: Wat wij ook nog wel even, van belang is om te benadrukken dat als, dat het niet zo is dat als je aan al die leefstijlaanbevelingen voldoet, dat je dan nooit kanker krijgt, hè, dat is niet zo. En het is van de andere kant ook niet zo, dat als je helemaal, hè, als je hier helemaal niet aan voldoet, dat je het dan wel krijgt, of het werkt een beetje twee kanten op, het is niet zwart wit, zeg maar. We weten dat als je bepaalde leefstijlgedragingen, dat die invloed hebben op het risico op kanker, maar of je het wel of niet krijgt, dat is niet dat hangt niet 1 op 1 samen. Dus dat is wel even van belang, je had een hele mooi analogie, hè, met inbraakpreventie.

Gesprekleidster 2: Je kunt je huis heel mooi beveiligen met allerlei preventie middelen daarmee verklein je de kans dat er wordt ingebroken, maar het kan nog steeds gebeuren. Dat is misschien goed vergelijken, om bij voor te stellen..

Aanpassen leefstijl
Gesprekleidster 1: Dat is misschien goed om inderdaad nog bij te vermelden. Um. Als jullie zo naar dit lijstje kijken, zijn er dan aanbevelingen die daar bij staan van dat zou ik nog wel willen aanpassen of eigenlijk voldoe ik, als je je eigen leefstijl vergelijkt met dit lijstje, komt dat dan overeen of zijn er nog dingen die je zou willen verbeteren?

V: Nou, ik drink iedere avond een glaasje wijn. Nou staat er dat dat mag maar, hè, ja

M: Je leeft maar 1 keer.

V: Ja, weet je, zoveel dingen waarvan ik denk dat ik het allemaal gezond doe en denk ik heerlijk mijn wijntje.

V: Dan krijg je het in ieder geval niet aan het hart zo moet je maar denken.

Gelach

M: Er is ook verschil tussen rode en witte wijn.

Gesprekleidster 1: Voor hart en vaatziekten is het specifiek de rode wijn inderdaad, dat daar zit blijkbaar een stofje in

V: Ik drink rode

M: Maar minder calorieën, dat is vaak best wel lastig om dat in te schatten. Broodsoorten daar zit hartstikke veel in en er zijn broodsoorten die hebben het dus weer niet. Ik bak tegenwoordig veel zelf brood. En dan zie ik het dus bij het bakken, dan ga ik naar de molenaar en dan als je op die etiketten leest en ook wat daar aan E-stofjes inzitten, ook vaak veel. Dus calorie-arm eten, dat is best moeilijk. Ik noem nu als voorbeeld dan brood, maar er zijn ontzettend veel producten, ga eens naar de yoghurt soorten kijken..

Gesprekleidster 1: Het is iets waar je eigenlijk mee bezig moet zijn, hè?

M: Je moet ieder etiket gaan staan lezen. Dat is natuurlijk iets wat je niet zo gauw doet.

V: Ik denk dat wij haast onderling het beste zouden kunnen zeggen, 1:12:23.7 laten we voor de zekerheid ook biologische producten zo veel mogelijk pakken, daar zitten zo min mogelijk toevoegingen ".."1:12:31.0

M: Maar ja, je moet wel wat voor biologische, soms staat er een keurmerk op waarvan ik dan ook denk, moet je even goed kijken.

M: We kopen zo groene stroom, we weten niet waar het allemaal vandaan komt. Niet uit een *clown*.

M: Dus ja, weet je, ik vind dat lastig om te bepalen. Ik heb eens geleerd dat 1 glas bier is net zoveel als 2 bruine boterhammen met kaas. Maar ja, waar zit het allemaal nou precies in. Lastig..

Gesprekleidster 1: Wat dat betreft is het natuurlijk aan de hand van, op deze manier staat ook wel lastig om in te schatten of je eraan voldoet ja of nee. Er is een specifieke..het is wel specifieker weergegeven.

V: Maar wat jij zegt met dat glaasje wijn, ik denk bijvoorbeeld ik drink nooit geen wijn, maar dan heb je een feestje en dan drink je bijvoorbeeld een paar glaasjes. Dan zou je haast zeggen, oh, dan ben ik niet verantwoord bezig. Je zou dan denk ik te veel op 1 avond drinken. We moeten ook kunnen leven, hè? Het moet niet je leven gaan beheersen.

Gesprekleidster 1: Dat is volgens mij ook wat er bij staat, hè? Niet overheersen inderdaad. Ja, precies.

M: Je zou helemaal normaal willen worden. Regelmaat is het beste, eigenlijk. 1:14:08.7

V: En overal waar “te” voor staat is niet goed.

Verandering leefstijl

Gesprekleidster 1: Als jullie naar dit lijstje kijken zijn er dan dingen waarvan je denkt,dat zou ik eigenlijk willen veranderen ten opzichte van leefstijl die je nu hebt. Nee, zie ik jou schudden. Jij ook niet, hè, M1. ".." 1:14:41.4 Zijn er voor jou dingen waarvan je denkt, daar zou ik wel, als ik dit zo zie, oh, dat zou ik wel willen aanpassen of heb jij ook zoiets van, nou, nee, het is gewoon, het is al .. en is dat dan omdat je denkt van nou, ik voldoe daar al aan.

V: Ja

Gesprekleidster 1: Of is het gewoon van dat je denkt van uh..

V: Ja, eigenlijk wel, ik geloof wel dat ik eraan voldoe, alleen minder rood vlees, dat nou, ik eet al niet zo heel veel rood vlees maar rundvlees is voor mijn man dus veel beter.

Gesprekleidster 1: Ja

V: Dus dat is wel een beetje vervelend.

M: ".."1:15:24.4 slecht

V: Maar dat bak je toch helemaal door

V: Ja, uiteindelijk wel.

M: Dan is het goed.

V: Zoals een biefstukje, ja.. ".."1:15:36.7

Gesprekleidster 2: Maar rood vlees betekent niet alleen vlees dat rood is als het gebakken is maar gewoon als het rauw is rood.

V: Dus eigenlijk zou je moeten zeggen minder vlees.

Gesprekleidster 2: Bijna wel, ja, kip.

V: Ja, kip is dan infecteerbaar, dat is dan goed te doen, maar zoals zeg maar rund, varken dat zou je eigenlijk moeten schappen.

Gesprekleidster 2: Nou, in de richtlijn staat maximaal 500 gram per week dus dat is best nog wel..

M: Is dat rauw of bereid?

Gesprekleidster 1: Beide

Gesprekleidster 2: Dat durf ik niet te zeggen.

M: Ik heb het in ieder geval nog nooit gehoord, hoor.

V: Maar dat is toch bijna alles, allemaal rood?

Gesprekleidster 1: Er is heel veel..

V: Ik zit een beetje te denken, wat eten we dan allemaal, zoals rundervink. Dat is ook rood.

Gesprekleidster 2: Ja, klopt.

Gesprekleidster 1: Maar, dan is het dus minder vlees, in het algemeen als dat het vlees. Betekent dat dus minder vlees, of juist inderdaad meer een keer iets anders. Of juist relatief meer kip. Vis in plaats van vlees.

M: Ja, is ook lekker, hoor. Fruit vind ik ook wel moeilijk. 1:16:57.0

Gesprekleidster 2: Want hoeveel fruit eten jullie op een dag?

M: In de zomer is het veel fruit, maar zo rond deze tijd zakt het weer weg, hè. De kersen zijn weg, dus dat zachte fruit is verdwenen, de sinaasappels komen nu weer een beetje, maar die komen uit de koelcel dus die zijn nog steeds droog. Appels ook uit de koelcel als ik naar de Betuwe rijd en ik haal een kilo appels van de boom dan is het een wereld van verschil als bij de, ja, gewoon bij de groenteboer haal. Fruit is dus niet zo gek veel in de wintermaanden.

Gesprekleidster 1: Ik hoor een beetje dat u het dan ook minder lekker vindt. Het fruit, dat dat dan een reden zou zijn..

M: Sinaasappels schoonmaken hebben mannen altijd een beetje moeite mee.

Gelach

Gesprekleidster 1: Sinaasappel schoonmaken

M: Het is eigenlijk hartstikke droog van binnen.

M: Moet je grapefruit eten

M: Dat kan

M: Is sterker en die

Gesprekleidster 1: Hoe is dat voor jou, V5? Als je die lijst zo ziet.

V5: Ik vind het een goed lijstje.

Gesprekleidster 1: Nee maar, wat, zou je iets willen veranderen wat daar op het lijstje staat?

V5: Dingen die er niet op staan, zei ik net ook al een beetje, die er wel op zouden moeten, dus

Gesprekleidster 2: Waar nog te weinig over bekend is.

V5: Ik denk dat er nog heel veel onbekend is. Dat er nog veel meer boven tafel mag komen.

Gesprekleidster 1: Maar dat is ook het veld dat in ontwikkeling is, hè?

V5: Moet een beetje meer op z’n plek vallen.

Gesprekleidster 1: Ja, maar zijn er, ja en wat je had het net al over, melk, hè, zijn er nog andere dingen?

V5: Bijvoorbeeld

Gesprekleidster 1: Ja precies, maar zijn er dan nog andere dingen?

V5: Ik ben geen onderzoeker.

Gesprekleidster 1; Nee, maar goed, u zegt er zijn nog veel meer dingen die niet bekend zijn, die er eigenlijk op zouden moeten staan op dat lijstje. Zijn er nog specifieke dingen die je in je hoofd hebt, waarvan je denkt die zouden er nog bij moeten staan? Of is dat meer in zijn algemeenheid dat je bedoelt.

V: Wat je wel en niet moet eten? Is het het beste producten te eten die gewoon hier om me heen groeien en geen rijst of sinaasappels.

Gesprekleidster 1: Ja

V: Er wordt wel gezegd om sinaasappels te eten maar die groeien hier niet. Dus wie zegt dan dat dat voor mij in dit klimaat goed is om te eten? En dat staat ook een beetje bekend ".."1:19:52.2 voedingsadviezen. Dus ik neem dit met een korreltje zout, want volgens mij zit daar een hele wereld achter.

Gesprekleidster 1: Dat is ook zo.

V: Daar ben ik heel erg benieuwd. Maar volgens mij ga ik daar niet meer achter komen in mijn leven.

Gesprekleidster 2: Nou ja, de stappen gaan wel snel.

Gesprekleidster 1: De stappen gaan wel snel inderdaad, veld wat echt wel flink in ontwikkeling is, hoor.

V: Ik vind het heel langzaam, want ik geloof sinds een paar jaar een beetje is aangepast terwijl er al heel veel bekend was wat er niet echt goed aan was.

Gesprekleidster 1: Maar, wat wij dan meekrijgen het veld van voeding en kanker, dat is wel in ontwikkeling, daar gebeurd best veel. En onderzoek gaat natuurlijk altijd relatief langzaam. Want het liefst zou je nu resultaten willen hebben, maar zo werkt het gewoon niet. Als je onderzoek doet naar voeding en de relatie tot het krijgen van kanker, ja, daar zit natuurlijk, hè, jaren aan vooraf. Dat is een onderzoek waarbij mensen gevolgd worden, hè. Waarbij mensen vragenlijsten invullen van wat eet u nou wat doet u nou? En dan wordt daarna gemonitord krijgen deze mensen nou kanker ja of nee. Maar dat duurt natuurlijk jaren en jaren en jaren voordat je die gegevens boven tafel hebt, het is een heel goed onderzoek, maar dat is niet zo dat..

V: Nee, en niet iedereen vult ook hetzelfde in. Ik bedoel..

Gesprekleidster 1: het is complex, hè?

V: Als het nou standaard was, dan was je er zo uit, maar je hebt zoveel verschillende mensen met zoveel verschillende gewoontes.

Gesprekleidster 1: En die producten die ontstaan uit verschillende voedingsstoffen, ja, dat is complex

M: Maar die vraag is wel grappig, die jij stelt, datgene dat niet hier om je heen groeit of dat wel zo verstandig is om te eten. Heb je, dat, hooikoorts, kun je het beste honingen nemen vanuit je eigen omgeving, hè.

V: Wij hadden het laatst over, vroeger werd er puur alleen maar aardappels en groente gegeten. En zo'n variëteit in eten en alles zal dat ook niet invloed kunnen hebben dat nu is ons lichaam daarop gebouwd om al die aparte gekruide dingen te eten.

V: Nou, dat zei ik niet eens..

V: Jij zegt van, ja, die groeien inderdaad geen rijstplanten en noem maar op. Nu zijn de aardappels, mensen aten dat vroeger, maar nu is er zo'n variatie uit voeding waar je uit kan kiezen. Is dat eigenlijk wel bevorderend voor ons?

V: Moeten we ook niet vergeten. De supermarkten wat bijna allemaal werkt met iets toegevoegd.

V: Als we dat nu met z'n alle zouden schrappen, zou het verboden worden, wat zou er dan over blijven om te eten?

V: Gewoon je eigen moestuintje.

V: Hele andere dingen, meel van de molen in plaats van voorbewerkt uit een bakje. Dus het is ook een beetje de voedselindustrie, die ja ..

M: Maar als alle directeuren van Friesland Campina zeggen, we hebben de suiker in de halfvolle yoghurt met 20% verminderd, daar zijn we al heel blij mee en dan ben ik zo'n iemand waarom niet met 100% verminderd. Nee, dat vinden mensen niet meer lekker, overal zit suiker in. Dus het zo flauw.

V: En zout

M: Zout ook, maar dat is toch flauw, maar zout kun je nog zelf

Gesprekleidster 1: Kan je zelf doseren.

M: Maar hoewel in brood enzo

Gesprekleidster 1: Daar zit het ook in.

V: Neem de krankzinnige cup a soup. Wat dacht je daarvan?

V: Dat is volgens mij 1 zoutbal.

V: Dat kan toch helemaal niet.

V: Het gebeurt wel.

M: Omdat het je een beetje opkikkert.

M: Bij lange vergaderingen, zei ik altijd van jongens even een beetje ossetaartsoep nemen 1:24:13.0 soep nemen, hè.

Gesprekleidster 1: Ja

M: Dat was het meest zoute wat er was.

Gelach

V: Nou ja, vroeger aten ze natuurlijk heel veel vet, hè ".."1:24:34.6 en al dat soort spul.

V: Ja maar, weet je. Ze bewogen veel meer, hè.

M: Ja, ze bewogen veel, ja zeker.

V: Dus de verhouding was beter, maar dat is nu niet meer.

V: En de dieren leefden anders, het was meer gewoon op het land allemaal.

M: Ja, ga eens naar Friesland fietsen, dan zie je hoe weinig koeien er in de wei staan. Maar de boer maakt zich wel druk over het grasmaaien.

V: Ja, ja precies.

Gesprekleidster 1: Hé, we hebben nog een kwartier. Dan zitten we alweer aan de 2 uur. Die zijn alweer voorbij.

Gelach

Persoonlijke top 3

Gesprekleidster 1: Maar, ik wil een beetje gaan afronden. Wat we in ieder geval nu nog even gaan doen is sowieso eerst nog even een rondje, waarbij we die lijst nog eventjes verder aanvullen met factoren waar jullie eventueel nog opkomen. En dan zou ik graag..

Gesprekleidster 2: De persoonlijke top 3 vooral, toch?

Gesprekleidster 1: Ja, en inderdaad, of jullie willen aangeven of dat, wat voor jullie de 3 belangrijkste factoren zijn die in jullie geval je leefstijl beïnvloeden. Dat kan voor iedereen, maar misschien staan er bepaalde factoren nog niet genoemd. Zou jij misschien willen beginnen staan er factoren op het lijstje waarvan je zegt die zouden er eigenlijk nog op moeten staan?

V: Um

Gesprekleidster 1: En misschien willen jullie daar ook alvast over nadenken. Als jullie dingen zien staan waarvan jullie denken, die moeten er eigenlijk bij staan dan roep maar tussendoor. Wat zijn voor jou 3 belangrijkste factoren? Jou bijvoorbeeld, wat beïnvloedt jou het meest? Als je kijkt naar dit lijstje?

V: Ik kan nergens antwoord 1:26:40.8 op geven.

Gesprekleidster 1: Nee?

Gesprekleidster 2: Ik hoorde net volgens mij heel erg dat het vooral een gewoonte is, gezond leven. Klopt dat?

V: Nee, dat kwam niet van mij.

Gesprekleidster 1: Oké, maar hoe komt het dat je daar geen antwoord op kan geven? Staan.. Vind.. Vind je het lastig om te kiezen hieruit?

V: Nee, ik vind het lastig om antwoord te geven. Misschien sluit ik straks achteraan de rij.

Gesprekleidster 1: Nou prima, We gaan naar de andere kant.

Gelach

Gesprekleidster 1: Sorry, we gaan even zo. V1?

V1: Ik ga het proberen. Die voor mij het belangrijkst zijn. Dat is dus gezond zijn, gezondheid en hoe dan dus dat je gezond eet, gezond beweegt voor geen spanning ofzo, hè. Um informatie die je iedere keer toege.. dat je iedere keer goed informeert moet worden van alles. En leuke dingen doen in je leven dat dat heel belangrijk is.

Gesprekleidster 1: Dat zei je toen straks ook al, hè, dat je niet er door geleefd wordt, door die gezonde leefstijl.

V: Dan zou ik zeggen, goed voor je darmen zorgen. Sowieso voor al je organen moet je zorgen, maar omdat wij nu in deze situatie zitten, zou ik dat je goede voeding binnen krijgt.

M: Daar heb je zelf ook het meest plezier van.

V: Ja, precies.

Gesprekleidster 1: M1, wat zijn voor jou de drie belangrijkste factoren?

M1: Nou, we hebben dus in de familie op een hele jonge leeftijd mensen verloren. Sindsdien is wel oorspronkelijk bij ons thuis.. Je leeft maar 1 keer, geniet gewoon van elke minuut elke dag wat je hebt. En dat is bij ons de eerste en dan we letten ook op onze gezondheid en we proberen onze conditie goed te bij te houden. Voor de rest het gaat zoals het gaat. En dat is het. De ene keer gaat die linksom en de andere keer gaat die rechtsom en.. Bij een feestje genieten we daar van. En de volgende dag kijken we weer en zo leven wij, misschien niet helemaal goed, maar zo leven wij wel.

Gesprekleidster 1: Nou mooi, en jij wat zijn voor jou de drie belangrijkste dingen.

V: Ja, genieten denk ik wel. En ja, conditie en eten dat patroon hoort voor mij eigenlijk bij elkaar.

Gesprekleidster 1: En wat is dat patroon eigenlijk?

V: Een eetpatroon

V: Ja, gezond leven, ja

Gesprekleidster 1: Oké, dat is het belangrijkste.

V: Ja, het werk beïnvloed je leefstijl natuurlijk, daar kun je niet omheen, maar dat beïnvloed soms ook het genieten. En ik denk als je, het je geeft al aan, je ziet om je heen mensen op jonge leeftijd sterven, dat hebben wij dan niet. We zitten met z'n drieën in zo'n onderzoeken maar uiteindelijk komen we er iedere keer goed van af. Dan je gaat pas genieten als je het heel dichtbij meemaakt. En ook dat ".."1:30:31.2 weer weg, moet ik zeggen. Dat zag ik in het ziektebeeld van mij moeder ook, die heeft ook een periode gehad dat ze zogenaamd genezen was en dan na een aantal maanden komt toch het gewone leven weer om de hoek kijken. Totdat het voor een tweede keer weer gebeurt. En ja.. genieten is natuurlijk.. ja, je probeert te genieten, maar ja..

Gesprekleidster 1: V3, wat zijn voor jou de belangrijkste factoren?

V3: Nou, ik heb dan de kanker gehad en nog langer willen leven dat is voor mij dan heel belangrijk. En dat goed gecontroleerd worden in een ziekenhuis, dat ja. En um, even kijken hoor, ja, ik vind je goed voelen, conditie, gezondheid ja dat vind ik dat hoor bij elkaar.

Gesprekleidster 2: Dat is één eigenlijk.

V3: Dat vind ik heel moeilijk dat te onderscheiden. Dat is voor mij dan de top 3.

Gesprekleidster 1: Oké, M3?

M3: Goed gezond, genieten en alles ik vind zeker ook heel belangrijk een goede nachtrust, hè, op tijd rust en de volgende dag toch goed vooruit kunt. Ja ..

Gesprekleidster 2: Is dat een beetje ritme, regelmaat?

M: Ja, rust, regelmaat en reinheid

Gelach

V: Dat zit er zo ingebakken, hè. Het is verschrikkelijk haha

Gesprekleidster 1: M2 wat zijn voor jou de drie belangrijkste?

M2: Ja, ik zat te denken, um, veel drinken mis ik nog.

Gesprekleidster 1: Veel water drinken of veel?

M2: Veel water, koffie dus niet, hè. Maar um gewoon voldoende, ruim voldoende drinken. Dat is heel belangrijk, anders is die darm helemaal, wordt de ontlasting zo naar. Je merkt ook dat het afneemt, dus dat is zo, heeft ook te maken met goed voor de darmen zorgen. Dus dat is eentje en dan goed voelen, conditie, gezondheid is dan ja. En genieten.. Nou ja, genieten maar *stilte*

V: Nou doe mij dan maar goed voelen.

Gelach

Gesprekleidster 2: Staat erop

V: Dank je wel.

V: Ik denk dat het de basisvoorwaarde is om..

V: Ik denk dat eigenlijk alles wat daarop staat eigenlijk voor ons van belang. Want je kan ze zeggen dan kies je er drie maar eigenlijk ieder punt heeft iets voor ons.

Gesprekleidster 2: En voor iedereen weer wat anders.

V: Ja

Gesprekleidster 2: Ik vind het wel mooi om terug te zien dat er toch een aantal zijn die bijna allemaal aangeven als belangrijke dat geeft toch wel aan dat die heel belangrijk zijn.

M: Je moet eerst wat gehad hebben, om, voordat je je bewust wordt 1:33:51.4

Gesprekleidster 1: Dat is voor ons ook gewoon heel informatief als we op die manier turven ook als we kijken naar wat jullie het belangrijkste vinden. We zien natuurlijk ook dat in de andere groepen, als je dan toch bepaalde factoren naar boven krijgt die heel veel terug komen dan zijn die op 1 of andere manier toch wel extra waardevol extra belangrijk. En dat is het een beetje waarom we dat doen.

V: Dat wil ik nog even aanhalen, toen ik dan dus te horen kreeg met mijn man en kinderen dan dat ik dus het Lynch syndroom heb. Ja, dat veranderd het leven eventjes, krijg je een andere kijk op het leven. En toen is bij ons wel heel, heel sterk en vooral bij mijn man is dat heel sterk naar boven gekomen van wat moeten genieten. We moeten van ieder moment genieten als we iets leuks kunnen doen, dan moeten we het doen. Dus bij ons staat genieten in het gezin heel centraal.

M: Maar ja, genieten kan nogmaals ook ".."1:34:50.8

V: Dat zou gewoon met een niks doen, met een kop koffie in de tuin. Maar gewoon, dat je ook tijd voor elkaar maakt en dat je geniet. Hè, kan heel simpel zijn, bijvoorbeeld gewoon lekker samen in de tuin eten. Dat je mensen uitnodigt, soms met je eigen gezinnetje maar juist die kleine momenten dat je er van geniet. Dat komt eigenlijk heel sterk bij ons naar boven.

M: ".."1:35:15.3 van iets. Want ze zou bijvoorbeeld ".."1:35:17.8.

V: Ja, precies, ja

V: Mag ik nog heel even, wat er eventueel nog bij zou kunnen, hè, naar aanleiding van jou, is tijd voor jezelf. Want dat merk ik dat ik dat heel sterk met mezelf heb, omdat er toch wel heel veel dingen gebeuren iedere keer dat ik denk van, nu ik wil helemaal niks, alleen maar tijd voor mezelf. Ik noem maar wat, uitslapen en dan niet de dingen doen die je normaal doet, hè, als je moet werken moet je gelijk douchen en hup en weg, nee, dan ga je lekker zitten, ontbijten kopje koffie, krantje nou dat is voor mij genieten. Vind ik heerlijk, gewoon tijd voor jezelf.

Afronding

Gesprekleidster 1:Oké, ja laten we gaan, ik moet even de boekjes waren we even vergeten om mee te nemen hier naartoe. Die gaat Gesprekleidster 2 eventjes halen en in de tussentijd maken wij gewoon even een rondje met wat laatste opmerkingen, vragen of wat eventueel nog niet gezegd is in het gesprek. Wat jullie nu nog kwijt willen en dan gaan we afsluiten. Zou jij misschien, kan ook zijn dat je helemaal niets toe te voegen hebt.

V: Nee, ik ben het helemaal met je eens. Ik vind, ja, ook heel leerzaam dit.

Gesprekleidster 1: Nou mooi, M1 had jij nog iets wat je? Nee

V: Nou, eigenlijk ook niet, maar ik vond het wel fijn om ook anderen te horen dus ik heb het wel.. Ik wist niet wat ik moest verwachten, maar ik vond het wel heel prettig.

Gesprekleidster 1: Goed zo, dat is fijn om te horen. Mooi, M3 heb jij nog iets wat je wil?

M3: Nee, het staat er eigenlijk op.

Gesprekleidster 1: Nou mooi, M2 is er nog iets wat je?

M2: Nee niet, wat ik wel interessant vind, waarom doe je dit onderzoek? Want je kunt ook kunnen doen naar oogheelkunde of dermatologie ik noem maar is wat. Waarom doe je dit?

Gesprekleidster 1: Bedoel je mij persoonlijk, waarom ik dit nou kies? Omdat het binnen mijn onderzoekslijn valt, omdat het hè, ik vind het heel interessant om net zoals ik al zei, ik doe onderzoek binnen voeding en kanker en met name ook met leefstijl en gedragsverandering, met name, en dit valt daar helemaal binnen. Dit is eigenlijk meer, als je onderzoek doet, je gaat op een gegeven moment toch naar je eigen niche toe. Hè, je gaat op een gegeven moment een beetje werken, samen met *Prof* ook met voeding en kanker. Zij is epidemioloog en zij hebben meer vanuit het perspectief van de publieke gezondheid en ik werk samen met *prof* hij is hoogleraar Preventie van overgewicht en ook voeding en hij doet ook veel met leefstijlinterventies en, um, dit is eigenlijk een beetje mijn gebied waar ik mee bezig ben en, um, dit project heb ik samen met *prof* aangevraagd. En dat is gehonoreerd en dat is een van de onderzoeken die wij nu uitvoeren. Dus het eerste antwoord is eigenlijk interesse puur, ja, dit is mijn interesse, je hebt allemaal zo je gebiedje waar je onderzoeken aangevraagd en onderzoeken uitvoert. Iedereen heeft zo z'n eigen niche, zeg maar, van, dit is die van mij. Daarom doe ik daar onderzoek naar. En dat is eigenlijk goed, we hebben dat nu met name zoveel natuurlijk, hè, dit is eigenlijk de nog niet eens helemaal precies waar ik mee bezig.

Het heeft natuurlijk wel raakvlakken daarmee, um ja, ik vind het gewoon heel erg interessant, ik vind het heel leuk om te doen.
V5, Is er nog iets wat jij nog zou willen toevoegen?

V5: Nee, wat ik jullie heb ".." 1:39:57.8 vertel hebt toen ik hoorde dat ik een bepaald gen heb. Er kwam een meneer aan de telefoon en toen zei en nu dan en toen zei die ja voor zover we nu deze gegevens hebben, kijken wie een vervolgonderzoek oppakt. Toen dacht ik, dat hangt dan ook dus een beetje af van toeval of je nou mutatie verder wordt onderzocht of niet. Ik geloof dat er iemand gepromoveerd is of dat weet ik zeker, dus daar heb ik dan wel het geluk bij, want nu weet ik ook, daardoor is ook bekend hoe de kanker een grote rol speelt. Maar nu heeft die persoon het laten liggen, ben haar naam kwijt maar dan was dat misschien niet boven tafel gekomen zo snel. Het is een beetje..

Gesprekleidster 1: Het is ook maar net inderdaad wie daar onderzoek naar doet en inderdaad, je ziet vaak inderdaad dat als zo'n onderzoeksproject af is gelopen als iemand gepromoveerd is, inderdaad, dat duurt meestal 4 jaar. En als dat voorbij is, komt er opnieuw geld, wij moeten ook ons eigen geld binnen halen. We moeten onderzoeksvoorstellen schrijven en dan soms lukt het om geld binnen te halen om vervolgonderzoek en soms lukt het niet. Nu, in onze wereld, in de onderzoekswereld, is het ook soort van crisis, hè, er is ook natuurlijk een beperkt budget beschikbaar waar je op kunt aanvragen en soms lukt het niet om op een bepaald onderzoek verder te kunnen gaan, want je hebt uiteindelijk toch geld nodig. Iemand die gaat daarop werken die gaat daar, het is ook maar net vanuit een ander perspectief bekeken vanuit de subsidie verstrekkers die bepalen waar het geld naartoe gaat en soms zijn er andere prioriteiten en dan, dus, het is maar net inderdaad wat loopt op dat moment en ja, gaat daar weer iemand op door.

Gesprekleidster 2: Jullie hebben het rondje gemaakt.

Gesprekleidster 1: We hebben het rondje gemaakt, oké, nou, dan zou ik jullie als laatste heel erg willen bedanken voor jullie deelname aan..

Focusgroep 5 Transcript 5 Nijmegen 141013 #00:00:00-0#

Gesprekleidster 1: ... te gaan houden en dan zou ik willen vragen om te melden hoe lang weet dat u Lynch syndroom heeft. En nou, u mag over uzelf vertellen. Of u de diagnose al **, of dat u geopereerd bent, wat u kwijt wilt daarover. Over hoe het met u gaat. Mag ik u vragen om te beginnen? Sorry, daar begin ik al, hè, we zijn maar net bezig. #00:00:30-3#

M: Ik streep gewoon mijn achternaam door. #00:00:31-8#

Gelach #00:00:35-5#

V1: Nou, ik zal beginnen. Ik ben V1 en ik weet, ik weet niet helemaal precies, maar ik dacht 1997, naar aanleiding van dat mijn vader al heel jong is overleden aan darmkanker, zijn vader. En nou, toen overleed mijn broer ook op 48 jarige leeftijd. ".." #00:00:57-9# Een zus van mij die had al met 40 jaar kanker, maar dat is gewoon goed gegaan door darmkanker. Dus zodoende is het aan het rollen gegaan. Dus *ZKH8*, en nou ja, het heeft eigenlijk twee jaar geduurd voordat ze er uitkwamen. Ze hebben het weefsel van mijn vader uit 1966 hadden ze nog. Maar, daar hebben ze het niet in gevonden uiteindelijk in het bloed. Dus ik kom uit een gezin van 6 en nou ze zeiden altijd van nou, jij hebt het in ieder geval niet. Zo kwam het eigenlijk over. Dus van de 6 kinderen hebben dus 4 Lynch. En nou, daar hoor ik dan ook bij. Waarvan dus mijn oudste zus ook overleden is en mijn jongste zus heeft ook al darmkanker gehad. En tot nu toe ben ik de enige nog die schoon is. Er is 1 keer heel klein poliepje, dat was, zeg maar, 2 jaar, maar ja, verder niet. #00:01:58-1#

V: Ja, ik kom van vroeger uit en daarom vonden ze het ook heel erg interessant, uit een inteelt familie, dus verwar me niet met, 1/6 inteelt familie. Dus mijn, ik heb nog een oude bijbel en ik heet dus **. En dat kreeg ik iedere keer weer terug, vroeger was het dan ** van ".." #00:02:26-6#. Dus er werd altijd met neef en nicht getrouwd. Dus dat zijn dus, dokter **, die zullen jullie ook wel kennen van hier, wat zullen ze hiervan smullen van het lab. Het was namelijk een beetje bekend met dat in bepaalde groepen, die dus het hadden, waar dus Lynch in voorkwam. Ik heb zelf 3 kinderen, de jongste zoon, ja, die weet het nog niet. Die is 24, maar jongste, ik heb nog 2 dochters. Mijn jongste dochter weet het niet, mijn oudste dochter heeft het toch maar laten doen. En die heeft het Lynch syndroom. Die heeft controle, krijg wel veel, toch wel informatie, paar keer meegedaan met de leefstijl in Wageningen. Heb ik al een paar keer de dikke darm op een camera van de dunne darm #00:03:23-9#, dunne darm. Ja, levenswijze, ik heb nooit gerookt, maar dat is niet dat ik vroeger erop tegen was, maar ik was gewoon te zuinig, want ik ging liever sporten. Dus dan ga je dus, ik heb eigenlijk nooit gerookt. Eet wel frituur, maar heel erg zelden. Maar dat is niet omdat ik daar zo bewust mee bezig was, maar ik heb er nooit behoefte aan gehad. Dus dat is eigenlijk, drink wel altijd koffie, maar ik heb wel altijd een hele levenswijze, wat ze zeggen dat dat gezond is. Maar het is niet zo dat, ik ben er zo mee bezig, nee. Dus verder niet. Ik geef dus zelf dus sport #00:04:05-8# ik heb altijd, ja, beweging genoeg. Nou, dat was het wel zo'n beetje. #00:04:08-6#

Gesprekleidster 1: Dank u wel. #00:04:11-0#

M1: Ja, M1, getrouwd en 2 kinderen van 24 en zoon van 22. We wonen hier in Wijchen. Ik weet sinds 1998 dat ik in bezit ben MSA2 gen, um, mijn broer en zus die ook allebei hebben. Qua familie, mijn moeder is overleden aan, uiteindelijk, het is begonnen in de baarmoeder maar uiteindelijk zat het overal. Niet wetende van en, um, familiair, paar jaar later, mijn moeder stierf met 53 jaar, um, kwam dat eigenlijk aan de orde. Mijn moeder was een, was eigenlijk, de moeder van mijn moeder was 2 keer getrouwd. En wij kwamen in contact met de familie van de eerste vader en daar blijk dus een, dat er zeg maar, neven en nichten waren omgevallen en gestorven aan darmkanker. En um, uiteindelijk kwamen die met de opmerking aan mijn vader #00:05:25-1#, is eigenlijk een volle nicht van ons.. Misschien moeten jullie je ook eens laten testen #00:05:32-3#
En wij waren alle drie in bezit van, toen zijn ,zeg maar, sind 97/98 #00:05:42-0# nu bezig met onderzoeken. Eerst alleen de darmen um uiteindelijk kregen we het advies dat we ook de urineleiders zouden moeten laten nakijken. Ik loop nu een jaar of 4. Dus 1 jaar een echo voor de nieren en urinewegen en daarna inwendig onderzoek. #00:05:58-6# Om de 2 jaar een darmonderzoek, um, mijn broer heeft inmiddels, um, blaaskanker gehad. Mijn zus heeft baarmoeder en is inmiddels weer afgenomen en dat was het en ik ben nog steeds schoon. Al die jaren, um, levenswijze ik rook niet kort gezegd, ik kijk niet naar eten, ik eet

gewoon wat ik lekker vind. Ik sport twee keer per week, ik drink op z'n tijd een borreltje. Door de week nooit, maar in het weekend wat drank. En dat was het eigenlijk wel, dat is wel lekker. #00:06:49-4#

M2: M2 dan sinds '88 is het gebeurd, het is familieonderzoek gekomen omdat een aantal ook kanker hadden. De universiteit Wageningen gekomen en toen is hele familie is dan in Wageningen bloed afgenomen. Dat geeft zo'n 4 jaar geduurd tot dat ze eindelijk er achter waren waar het zat bij ons. In 1995 heb ik bloed afgestaan hierin *ZKH5* en meerdere van de familie sommige wilden het niet weten die zijn terug gekomen. Dus dat was echt goed mis. Um, mijn vader had het dus ook met 37 jaar, ik heb elk jaar onderzoek, poliepen worden steeds groter. Vorig jaar was een grote, dit jaar hebben ze drie grote poliepen verwijderd en 2 kleine. Dus dat was wel, dit jaar zat ik even te knijpen. Um, het is bij mijn broer wel gevonden, die is in 1943 of '44 overleden aan kanker. Ik heb ooms en tantes, eentje was 34 andere was 36 volgens mij, en mijn vader is zelf 54 geworden. Die is, toen hij 37 was, heeft hij ook een stuk dikke darm toen weggehaald. En in 1996 hebben ze ".." 0:08:37.3 preventief verwijderd, de twaalfvingerige darm, dunne darm, maar dat is niet gelukt. Mijn vader is toen in augustus geopereerd hier en toen is hij overleden.

Gesprekleidster 1: Aan de complicaties van die operatie dan ook?

M: De tweede chemokuur was er was eigenlijk ook gezegd als operatie lang duurt dan is het een goed teken en als het kort duurt is het een slecht teken. Wat is lang en wat is kort? Rond 3 à 4 uur was kort, maar toen werd ik thuis opgebeld van, ja, we kunne verder niets meer doen. Toen is mijn vader chemokuren, en bij de tweede is het niet goed gegaan. En ik heb zelf drie kinderen, eentje 15, de ander is 13 en 1 van 10. En de oudste begint wel te begrijpen, bloed afstaan en kijken of het genetisch bepaald is. Zij is daar meer mee bezig dan de anderen twee, leeftijdsgebonden. Ik leg ook niet de stempel erop.
En ik leef verders, ja, gezond, ik eet wel voldoende groente en fruit, 2x per dag fruit af en toe in het weekend een Bacardi cola en dan houdt het bij mij wel op. 0:10:06.1

Gesprekleidster 1: Oké, nou M3?

M3: Ja, 1997 was het jaar dat mijn moeder het onderzoek ging doen, het bloedonderzoek om te kijken of dat wij de erfelijke vorm van darmkanker hadden. Aangezien mijn oma aan darmkanker is overleden, op 68 jarige leeftijd dat was in 1990. Mijn moeder en al haar broers en zussen, zij komt uit een gezin van 6, hebben het onderzoek gedaan en van de 6 kinderen van mijn oma waren 5 zeg maar gendrager. Maar, heel veel ooms en tantes van mijn moederskant, bijna allemaal overleden aan darmkanker, voor zover ik weet. Ik hoor jou net 1988 noemen, waren wij de tweede familie in Nederland in 1997 die gecontroleerd worden van het HNPCC gen. Dat was het voorjaar, mijn zus en ik zijn in het najaar gegaan van al mijn neefjes en nichtje was de uitslag ver boven de 50 %, zeg maar, die gendrager zijn. Um, het advies van toen, vanaf je dertigste levensjaar 1 keer per jaar gecontroleerd te worden. Ik was 29, nee, ik was nog 28, toen zat er bloed bij mijn ontlasting en bij mij ik had dus net bloedonderzoek afgerond en toen had ik al darmkanker. Toen ben ik 1 april '99 geopereerd, hebben daarbij de hele dikke darm weggehaald. Dat doen ze nu niet meer overigens. Nu lachen ze er volgens mij een beetje om, stukje tussen uit en over 5 jaar misschien nog een stuk. Maar ik heb het dus helemaal niet meer. Maar bij mij hebben ze de dunne darm aangesloten op de endeldarm, gaat redelijk. Je hebt wel een vorm van een handicap, um, van mijn moeder, broers, zussen is er niemand die tot nu toe darmkanker gehad. Vooral mijn neefjes en nichtjes,1 neef die was 23 toen hij het kreeg en die is op z'n 26ste overleden. Die had het onderzoek niet gedaan. Dus de leeftijd van nu, kijken of je gendrager bent is in onze familie terug gebracht naar 18 jarige leeftijd. Dus ik hoor jullie al zeggen ik heb kinderen, nou ja, goed, mijn neef was in ieder geval al te laat. En goed, neven en nichten van mijn moeder daar ken ik de verhalen niet van. Die zouden hier wel bekend zijn op zich. Dus, eigenlijk ja, mijn neef en ik zijn de enige twee die het gehad hebben en poliepen komen maar heel weinig voor in de familie. Maar ja, goed, wij hebben ze wel allebei gehad, ik ben degene die het overleefd heeft en hij niet. Maar goed, ook hun huisarts die vond hem zo jong. Hij had allang klachten maar hij had de link niet kunnen leggen. Terwijl toch die huisarts de rest van de familie allemaal heeft gehad, die heeft de link niet kunnen leggen naar iets ergs. Um
Hoe leef ik? Ja, ik denk in mijn jeugd best een leuke tijd gehad, zoals velen denk ik. Je wordt uiteraard steeds serieuzer. En ruim een jaar geleden het plan nog gepakt heb om de Alp d'huzes 0:14:11.6 te gaan fietsen. Dat heb ik gedaan het afgelopen jaar en twee potjes tennis die ik in de week deed bleek lang niet genoeg te zijn om die berg op te gaan. Sindsdien, ik tennis nog steeds en daarbij fitness ik 1 keer in de week en fiets ik, tot aan juni fiets ik 2 keer in de week. Ja, dus, sport behoorlijk veel. Let wel heel erg op voeding en

uiteraard zondig ik ook wel eens. Um, ik drink elke avond een glas rode wijn en op zaterdag misschien 2. Maar, ik kan het helaas niet laten op 's avonds een sigaretje te roken. Maar goed, daar ga ik hopelijk binnenkort mee stoppen 0:14:57.6

Gesprekleidster 1: oké, V2

V2: Um, ik ben, heb in 2001 te horen gekregen dat ik gendrager ben, word met drie weken 21. Vrij snel besloten om dat te laten onderzoeken. Mijn vader is ziek geworden toen hij 34 was, in '88 was dat. En mijn oom twee jaar later die is 32 en toen was er daar in het ziekenhuis, dat hoort niet. Mijn oma ook al een jaar of 25 daarvoor, ook al darmkanker had gehad. De hele familie is ook onder andere hier in het onderzoek gekomen op het moment dat de mogelijkheid er was om bloed te laten onderzoeken hebben ze ook gedaan en toen bleek dat van de 7 er maar 2 hebben. Dus mijn vader en oom die allebei al ziek waren. In tegenstelling tot die van jou is het bijna minimaal, um, mijn oom heeft geen kinderen dus daar houdt het op. Mijn vader heeft drie kinderen, we hebben echt alle drie laten onderzoeken en ik ben de enige. Dus van de ruim 30 nakomelingen van mijn oma zijn er 3, dus we zijn wat dat betreft er goed vanaf gekomen. Um, mijn oma had broers en zussen, broers waren van kanker overleden maar die had geen kinderen en bij de broers wordt een beetje bot gevangen, werd in de tijd een beetje bot gevangen door mijn vaders, ooms en tantes dus hoe het daar in de familie zit, dat is niet bekend dat. Ze willen daar niet over praten, dus misschien is er nog een tak van de familie waar het wel wat.. Sinds begin 2002 laat ik me elke 2 jaar onderzoeken dus het hoefde eigenlijk nog niet. Ik mocht nog een paar jaar wachten, maar ik wilde het graag weten. Het eerste onderzoek was niet hier, was in een ander ziekenhuis, was heel dramatisch verlopen dat ik heel netjes ben gegaan. Tot nu niets gevonden, afgelopen keer een heel klein poliepje, maar dat bleek een poliepje dat niet kwaadaardig kon worden, dus het was geen .. poliep 0:17:18.3 heet dat. Was het niet, um, en sinds maart van dit jaar doe ik hier in het *ZKH5* mee aan een vaccinatie programma dendritische cellen, dus heb net de tweede ronde achter de rug. Krijg over een maandje ongeveer de uitslag over hoe dat gegaan is. De eerste ronde is goed gegaan.

M: Is dat kapselen van het gen of niet?

V2: Nee, ze halen via bloedfiltering dendritische cellen uit je bloed, die gaan naar het lab en die worden eigenlijk getraind om te reageren op bepaalde eiwitten. Je moet een beetje voorstellen dat als je een keer een bepaald virus hebt gehad, als het virus je lichaam aanvalt. Herkent jou lichaam dat en weet daar moet ik tegen werken. Het is dus eigenlijk het trainen van dendritische cellen van bepaalde eiwitafwijkingen die horen bij poliepvorming horen. Ja inderdaad, de theorie erachter dat die mijn eigen immuunsysteem dan hopelijk inderdaad de zaak gaat opruimen voordat het zich tot poliep vormt.

V: Ik dacht dat jij er toen ook was. 0:18:39.9 Wij zijn toen naar een groep geweest met informatie waar we te weten kwamen dat we allemaal in aanraking kwamen, tot 70 jaar was dat. En dan kon je ervoor opgeven en in eerste instantie was het 0:18:53.2 heel erg duur. Kregen ze dan weer de subsidie, hè.

V2: Ja, het wordt volledig vergoed, er gaat niks aan mijn zorg..

V: Nee, maar ook niet voor jou, ook voor de mensen zelf krijgen ze dus voor die onderzoeken werd daar zoveel geld voor uitgetrokken.

V2: 20 miljoen euro geloof ik. 1 zo'n behandeling kost geloof ik 38.000 euro.

V: Ja, dat was wel heel interessant. Dus dan gaan ze dus, ja, ik weet niet precies hoe dat in elkaar zit, maar als dat klaar is en het werkt wel, dan werkt als het ware als een soort soldaatjes en als ze dan kanker oplopen wordt het eigenlijk opgevreten als het waren.

V2: Zelfs als bij poliepjes, bij poliepjes

V: Ja, als het in begin al is.

Gesprekleidster 2: Ben je toen ook geïnterviewd over dat..?

V2: Ja, ik ben op tv geweest

Gesprekleidster 2: Ik herken jou daarvan.

V2: Ja, dan ben je, dan is het gewoon leuk onderwerp.

V: En je bent daar niet, dat ze zeiden van, dat je even ziek bent als je..

V2: Een dagje, ik heb nou nog een paar hechtingen in mijn rug, want ik heb net bijna twee weken geleden. Zijn de laatste dingen gebeurd en dan een half jaartje en dan inderdaad.

V: twee jaar, hè, duurt dat?

V2: Nou, eigenlijk maar een jaar hoor, ben in maart begonnen, dan in september en in maart. Dus dan..

V: Oh ja, ze zeiden als het dan niet volledig is, dan kunnen ze nog een jaar doen dacht ik, hè?

V2: Nou, ze hadden een beetje, ze hadden gebracht alsof 1 keer was en misschien nog twee daarna, maar het is eigenlijk binnen een jaar tijd.

V: Valt het dan weer mee.

V2: Ja, het valt sowieso heel erg mee. De begeleiding is heel goed vanuit het ziekenhuis. Dus dat is heel fijn en dat is dus een kans die ik heb aangegrepen. Um, mijn vader en oom leven allebei nog. Prima gezondheid, mijn vader is wel een beetje door het oog van de naald gekropen, want hij kreeg voor de derde keer een operatie waar er een hapje darm uitgehaald werd. Alleen die operatie is een beetje geëscaleerd, dus hij heeft uiteindelijk 5 weken in het ziekenhuis gelegen door die complicaties. Maar goed, hij is laatst weer in het ziekenhuis geweest en kan weer hardlopen enzo.0:21:11.7"
Dus dat gaat heel goed, en voor betreft mijn eigen gezondheid. Ik heb dus nergens last van en je probeert inderdaad wel op bepaalde dingen te letten. Ik rook niet, ik drink amper, ik ben 15 jaar vegetariër, maar ja.

V: Als je zo aan het koken bent dan is het wel is moeilijk om er rekening mee moet houden, als je ook nog allerlei andere dingen gezonde voedingsstoffen enzo ook nog rekening mee moet houden. Dus ik heb nog geen kinderen, maar als die gaan komen, dan is het wel een optie om dat even, heel extreem, via een biopselectie te doen, dat mag tegenwoordig in Maastricht. Dus ik heb de mogelijkheid in handen dat ik in mijn familie gewoon de laatste ben, dat het bij mij stopt.

Gekregen informatie
Gesprekleidster 1: Nou, dank jullie wel voor jullie introductie, hè, nou, dan zou ik graag verder willen gaan met de volgende vraag en dat is "wat voor informatie, wat voor voorlichting hebben jullie gekregen op het moment dat jullie te horen kregen, dat jullie te horen kregen dat jullie Lynch syndroom hadden. Het is wel een beetje, in de introductie een beetje naar voren gekomen al, hè. Maar zouden jullie daar wat dieper op in kunnen gaan? Wat voor informatie kregen jullie te horen toen? Misschien al even geleden.

M: In 1997 kreeg je nog helemaal niks, kreeg je niks te horen.

V: Nou, ik toch wel. Ik had ook in '97 hier in het *ZKH5*, werd wel alles netjes uitgelegd. En er kwam zelf een maatschappelijk werkster. In ieder geval iemand of ik, ja, hulp nodig had. Kreeg een formulier van, als ik er niet zelf, er toch mee zat, ik kon altijd bellen.

M: Ja, dat stukje wel

V: De psychische kant wel.

M: Ja, de psychische kant werd wel belicht van het wel of niet willen weten. Ja, dat klopt, maar of wat het daadwerkelijk inhield of, volgens mij stond het DNA onderzoek nog zo in de kinderschoenen.

V: Ja, nou, dat mijn oudste zus had het al gedaan en mijn zus daaronder ook en die hadden in *ZKH8* en die hadden al heel veel informatie en die hadden in *ZKH5* de informatie en die hadden in *ZKH8* hebben zij dus bloed afgegeven. En ik dus hier, dus ik heb misschien, ben ik helemaal van hen had ik dan heel veel informatie van hoe het allemaal zat. En Dr. ** heeft toch heel uitgebreid, heeft alles laten zien ook met stambomen en ook met hoe het allemaal zit. Ik heb persoonlijk hele goede, toch wel, informatie en zelfs dat was van later. Zelfs nog een paar jaar geleden, de mogelijkheid om inderdaad, wat jij dan, de darm weg laten halen, maar dan kunnen ze het zo maken, dat ze dan wel gewoon met een uitgang 0:24:24.3 wel veel vaker naar het toilet, omdat je geen calcium, in elk geval geen dikke darm kanker, kunt krijgen. Ik heb van hieruit, ik heb dan, ik ga dan binnenkort met 0:24:41.2 pensioen. Maar, ik heb van Dr **, heb ik hele goede ervaring.

V2: Maar, het was wel heel medisch, wat ik te horen heb gekregen inderdaad, die de achtergrond en hoe het zat. Als je ermee in de knoei kwam, ik ben nog een paar keer een paar sessie bij **0:25:01.5 geweest. Maar praktische handvaten als wat ik moet je nou wel en niet doen heb ik niet gehad.

V: Nee, maar wat bedoel je met wel en niet doen, levenswijze?

V2: Bijvoorbeeld, ja, eten

V: Dat was toen ook nog niet denk ik nog niet zo veel bekend, hè, misschien ook wel, maar goed, iedereen weet natuurlijk dat bijvoorbeeld te veel roken, te veel drinken niet goed voor de darmen is. Dat is natuurlijk altijd slecht. Iets wat te veel is, hè?

M: Ja, ook 0:25:35.6 varkensvlees, was toen met name heel erg, hè, er werd, varkensvlees, was eigenlijk funest voor poliepen.

V: Ja, maar goed, de ene keer zijn tomaten tegen kanker, en de andere keer krijg je er weer kanker van. Dus dan weet je ook niet meer wat nou wel en niet wat nou niet goed is, hè. En ja, waarom krijgt de ene wel en de ander niet. Wat ik al zei, mijn vader was 46 en die klaagde eigenlijk al toen hij nog maar 31 was. Maar goed het was toen in die tijd, hè, dat kun je niet met nu vergelijken. Maar, de laatste dus mijn oudste zus, heeft darmkanker gehad toen ze ook eind 30 was. En later, dus kreeg ze, dus, die onderzoeken en die werden allemaal onderzocht en die zeiden, weet je, ik vertrouw het niet. Die man heb ik maar een paar gehad, ik wil een andere dokter. Ik weet het niet, hoor, dit is helemaal niet goed, iemand anders, maar ja, dat kon niet. En toen voelde ze zich helemaal niet goed. Dit is niet goed, ik voel me helemaal niet goed, nou, toen gaat ze weer naar de artsen, ja, bloed is niet goed. Kreeg bloedtransfusie, ja, darmkanker kan het niet zijn, want u wordt onderzocht. Ja, waar moeten we dan zoeken, ja, ik ben ziek, maar ze weten niet wat het is. Toen is ze naar ** gegaan naar het UMC, had ze wel darmkanker, maar ze waren nooit tot het klepje gegaan. Dat bovenin en bij ons, weet niet hoe het bij jullie het zit, bij ons meestal dus dikke darm, endeldarm daar is het meestal, maar nu zat het dus bij haar dikke darm, dunne darm. Nou ja, dus ze had het wel, ze hadden haar gewoon niet goed onderzocht. Ze was al twee jaar bezig dan heeft ze chemo's gehad. En toen zeiden ze, nou mevrouw, u heeft geluk gehad, u bent helemaal schoon. Het is helemaal in orde en zij overwinterde altijd in Portugal of in Spanje, nou, met de kerst gingen ze daar naar toe en, ja, ze zei net voor de Pasen, zei ze, het is echt niet goed, hoor. Ik voel me niet goed, nou, is ze daar, ze had een Nederlands huisdokter. Ze is daar naar de dokter gegaan en die zei, ga maar naar het ziekenhuis. Nou, toen hebben ze een echo gemaakt, ze zat helemaal vol, voor Pinksteren was ze overleden. Dus zo kan het ook gaan. Ik bedoel, dan heb je de onderzoeken, zo kan het natuurlijk ook gaan, dus ik heb altijd wel, als ik hier ben, dan zeg ik wel tot dat klepje, hè.

V: Dat je dus wel, ja, dan heb je die onderzoeken, het kan natuurlijk een keer dat je die pech hebt. Als je er dan zo'n tijd mee loopt en je weet gewoon zeker dat er niet in orde en je bloed is helemaal niet goed. Je krijgt een keer een bloedtransfusie en het is 0:28:29.2 niet goed, dan moet er iets zijn, hè. Ja, dat die pech gehad.

M: Bij ons in de familie ook dat volgend onderzoek dikke darm dan, in de overgang van de 0:28:50.2 dikke darm naar dunne darm, dat het daar ook begint. Mijn vader gehad, mijn nicht is vorig jaar overleden met 40 jaar, die had het ook en die neef van mij heb ik niet zoveel contact mee, maar daar hebben ze het dit jaar ook ontdekt. Dus ja, en was ook een sporter.

M: Darmkanker, je kunt zoveel sporten als je wilt, maar als je het hebt, dan heb je het gewoon, volgens mij.

M: En hij heeft ook geen ongezonde levensstijl.

M: En weet je, ik ben hier naar het ziekenhuis gegaan en mijn zwager zei, het is misschien wel gunstig om je kinderen te laten onderzoeken. En ja, we gingen hier naar toe, bij Dr. ** zijn wij geweest. En eerst gesprek gehad dat, ja, je hebt de vrijheid om het te doen, onderzoek maar. Uitgelegd wat de risico’s waren, als je het Lynch hebt. En het was toch wel zeker dat je tussen 90 en 95 % kans had om het te krijgen. Nou, we scoorden alle drie hetzelfde gen. En we zijn gestart met onderzoeken om de twee jaar. De coördinaten liggen al bij hen, maar nooit van gehoord, hoor. 1 keer in de twee jaar toe. En dan wordt het van binnen bekeken, en of ze tot het klepje gaan. "You know?"

V: Ja, dat komt natuurlijk als je een heel ding meemaakt.

M: Ja, nee, precies, maar we hebben ook geen contact met de..

V: met de arts

M: met de andere kant, zeg maar, de familie van mijn moeder, de echte familie van mijn moeder. Dus ik heb geen idee hoe het daar allemaal reilt en zeilt op dat gebied. Daar wordt, het enige wat mij, wat je dan hoort door de jaren heen, is dat je, ja, ze leren, uiteindelijk kregen we te horen, het is misschien wel gunstig als je ook je urinewegen laat controleren. Daar laten we je vrij in. De keuze is volgens mij niet heel moeilijk, want je kunt beter maar gecontroleerd worden dan niets doen. Um, dus daar loop je gewoon in mee en onderzoeken en de verhalen die ik om me heen hoor. Beangstigend wordt het, ik heb geen idee. Ze gaan tot het einde en ze kijken hem goed na en dan ga ik met een gerust gevoel weer weg. En dan wordt er geroepen, als je daar uiteindelijk doordat je kinderen leeftijd bereiken van de kritische grens van 25 jaar wordt het interessant van wanneer ga je starten en wat is er aan de hand. En dan wordt er duidelijk aangegeven, dat, laat je je onderzoeken zodat je er wel op tijd bij bent. En daar houd ik me aan vast, daar drijf ik op, zal ik maar zeggen.

M: Langzaam groeiende vorm van kanker, ik weet niet of dat voor jullie allemaal geldt, maar..

M: Is mij ook verteld en als je het hebt dan, um, of krijgt, dan blijf je, ja, het is niet een kanker die zich uitzaait heb ik me laten vertellen. Maar het is ook..

V: Bij mijn zus was het ook helemaal uitgezaaid, hoor, door alles.

V: Maar er zijn verschillende soorten, mutaties, hè? Dus het kan ook verschillend zijn wat u heeft?

M: H-PCC

V: Bij was het HUPCC, ik weet nog, V2, hè?

V2: Ja, je hebt MSA2 ik heb ook MSA2 en een vriendin van mij heeft MSA6.

V: Gennummer 7 hebben wij, heb ik tenminste.

V: Dat is een hele andere.

V: En dat houdt dan ook in dat ik elk jaar dus de baarmoeder en eierstokken, net zoveel kans, dus ook die 90 tot 95% kans op baarmoederkanker. Dus dat heb ik ook op een gegeven moment laten verwijderen, want

daar had je net zoveel kans op. En het zit allemaal aan mijn vaderskant en zijn zussen, zeg maar, zijn ook inderdaad aan baarmoederkanker overleden. En broers aan darmkanker, toen ben ik een beetje, we hebben niet zoveel contact, maar ook neven en nichten hebben ook al verschillende die leven, die hebben het ook allemaal gehad. Dus het is echt puur aan mijn vaderskant.

Gesprekleidster 1: En voor wat betreft, als jullie kijken naar de informatie die jullie gehad hebben in het verleden, werd en dan ook informatie gegeven over leefstijl in relatie tot het krijgen van kanker?

Meerderen: Nee

M: Dat is echt iets van de laatste 2 jaar ofzo.

M: Sinds ik met de Wageningen Universiteit in contact ben eigenlijk. 0:33:34.2 Ik heb die dikke boekwerken zitten invullen, eigenlijk vanaf dat moment. 0:33:44.7

V: Ik ben sinds 2002 wel heel erg naar op zoek geweest. Ik weet nog, het was met kerst, en ik kwam met mijn vader het ziekenhuis uit. En er stond een oliebollenkraam. En ik dacht, oh, dan mag ik nooit meer een oliebol, want het eerste wat mijn ouders deden toen mijn vader ziek was, was de frietpan de deur uit. We aten niet vaak friet, maar het was meteen van, dat, ze zeiden in het ziekenhuis wel, dat soort dingen zijn gewoon niet goed.

V: Ja, het hangt er ook vanaf natuurlijk, ik eet ook friet, alleen ik maak ze wel zelf en als je een goede, betaalde olie..

V: Ja, maar goed, dat zeggen ze dan nu weer, gezegd, maar toen was het gewoon, vet is niet goed. En ja, ik ging wel op het internet kijken maar ik vond zo ontzettend weinig en heel erg getekend door emotie ook. Dat mensen, heel erg was ook in zo'n tijd. Je moest geen deodorant gebruiken, want dat kon in je lymfeklieren trekken en borstkanker van krijgen.

Gesprekleidster 1: Maar je komt op internet van alles tegen, hè?

V2: Ja, dan word je helemaal gek.

M: Ik heb, ik weet dat ik het, heb uiteindelijk heb ik gekozen om mee te doen met Wageningen Universiteit onderzoek, meer, zo van, ik heb ook kinderen en alles wat we ervan leven. Voor de rest op het internet zoeken.

V: Dat was ook de in *steek* van mijn vader, in heel veel dingen, van, ik ga er heen voor jou. En ik doe dat voor jou en ik werk daar aan mee voor jonge mensen, dat het voor hem. Kijk, hij zegt al, hij leeft in de verlenging. 0:35:25.1

M: Ik heb wel met mijn dochter, was net geboren, zeg maar, dat was eerste reactie was, ik heb er spijt van. Wat jij zegt van die oliebol eten, elke keer was het zo van, als ik het eerder had geweten, had ik nooit geen kinderen genomen. Stopt het gewoon, wat doe ik ze aan. Kan je wel denken, we zijn inmiddels 20 jaar verder, ze kunnen steeds meer. Als ik dat van jou hoor, nou, weet je, nou..

M: In ons geval, je hoeft er niet aan dood te gaan. Dus dan denk ik, dat maakt het minder erg om kinderen te nemen. Ik heb uiteindelijk nu drie kinderen gekregen. En..

M: Gewoon, hoe noem je dat? Een bloedreactie die je op sommige momenten hebt. En uiteindelijk kwam er een tweede en kwam het weer goed. Maar ja, weet je, dat zijn reacties, maar voor de rest met eten en drinken.. Ik leef gewoon mijn leven en geniet van de dingen die je kan pakken.

Gesprekleidster 1: En hoe is dat bij jullie, we hebben net al een beetje over leefstijl gehad, hè. Zijn jullie daar bewust mee bezig, met jullie leefstijl verder? Jij zegt, doe gewoon mijn ding en ik houd eigenlijk nergens geen rekening mee. Hoe is dat voor jullie?

V: Ja maar, wat is nergens rekening mee houden? Als je altijd al, ik heb eigenlijk altijd al bewust geleefd, maar het ging eigenlijk altijd van zelf. Ik eet gewoon mijn groente en aardappels, rijst, die dingen. En ik ben altijd een matig mens geweest. En ik kan, ik weet niet hoe het bij jullie zit. Ik kan ook heel makkelijk als iets niet mag, dan doe ik dat gewoon niet. En ik voelde mij eigenlijk bevoorrecht dat ik die onderzoeken mocht doen. Ik heb ook geen, ik hoefde ook niet met iemand te praten verder. Ik kon er gewoon makkelijk over praten. Omdat, ja, ik ben bevoorrecht, hoeveel mensen die het misschien hebben, maar niet weten en misschien krijgen en ik mag in ieder geval. Het is niet een feit, hè, de ene keer ".."0:37:24.4 en de andere keer niet. Echt geen fijn 0:37:26.6 onderzoek, maar je moet ook weer de positieve dingen bedenken. Ik ben weer schoon, ik kan, ik heb weer energie. Je kunt weer, het is ook maar net hoe je er zelf beetje in staat, hè?

M: Het is een zegen dat ik het onderzoek mag doen. Heeft wel even geduurd 0:37:41.7 Dat heeft wel even jaren gekost en ik doe alles. Ben geen, hoe noem je dat, iets dat, ik ben geen bourgondier om te 0:37:54.0 zien. Ik sport op tijd en alles met mate, maar ik, het is niet zo dat ik alles afweeg en kijk..

V: Ik ook niet, ik zeg ook niet. Nee, dat heb ik ook nooit gedaan. Maar goed, ik was ook een stuk jonger, kijk als je een stuk jonger ben en je krijgt het dan te horen, dan dat je al die, ja, een stuk ouder bent. Je bent ook nog vrij jong, hè, dat je jonge kinderen hebt, dan en, nou ja, M3 helemaal. Ik weet het natuurlijk veel langer. En ja, eigenlijk wil je allemaal, ik denk ook verreweg de oudste.

M: Mijn leven heeft wel zeker 0:38:35.6 beïnvloedt, in positieve zowel als negatieve zin. Als je zo jong bent, met name de jaren erna, dan denk je daar ontzettend veel over na. En ja, ik denk dat ik op dit moment, hoewel ik al jaren, zeg maar, een dubbel leven leidt, zeg maar. Ik heb gelukkig maar heel weinig slaap nodig, 6,5 uur per nacht ofzo. Um, was vroeger als klein jongetje altijd bang, bang, om te vallen ergens vanaf te stappen of noem maar op. En nu, ja, ik ga racen op circuit, en in het weekend ga ik Quad 0:39:19.5" rijden. Ik zoek gevaarlijke dingen op. Ja, dat is denk ik wel een bewuste keuze, zeg maar, om, ja, ik wil bijna elke dag wel het gevoel hebben gehad dat ik iets leuks gedaan heb en dat is niet altijd, hè. En begrijp me niet verkeerd, ik weet eigenlijk dat de leukste dingen bijna altijd zitten in de kleinste dingen van het leven. Dan weet ik verdomd goed maar ik zoek echt heel extreme dingen op. En dat neemt mijn omgeving mij niet altijd in dank af.

Gesprekleidster 1: En wat bedoel je dan met ik leef een dubbel leven, wat bedoel je daarmee?

M: Um, ja, hoe moet ik dat nou zeggen? Um, ik ben altijd wel iets aan het doen en ben altijd dingen aan het verzinnen of, um, ja, ik lig nooit lui op de bang of..

Gesprekleidster 1: Bedoel je dat je eigenlijk gewoon heel veel doet?

M: Ik besteed mijn tijd, ik wil niet zeggen altijd nuttig, want ik vind naar de kroeg gaan met mijn vrienden nog steeds heel erg leuk.

M: Kan ook nuttig zijn.

M: Um, maar ja, wat ik al zeg of vakanties, maar dan niet drie weken aan het strand gaan liggen, maar met onze kinderen gewoon drie weken naar Zuid-Afrika geweest, daar alles gedaan wat je maar zo ongeveer kunt doen. Um ja, nu liggen alle programma's, afgelopen jaar Alpe d'Huzes 0:40:56.8 gefietst. Dat is op zich niet heel heftig, maar wel vermoeiend, laat ik het dan zo zeggen. Ja, nu zijn we aan het kijken of we Dakar 0:41:04.4 mee kunnen krijgen. Dat is niet gevaarlijk, maar dat soort uitdagingen, heb ik of de één of andere manier. Tien jaar geleden zou ik dat nooit gezocht hebben. En nu gaan dingen in mijn leven wel tot op het randje, zeg maar. Ja, ik zeg altijd, maar goed, mijn vrouw kan daar mee omgaan, maar mijn ouders, mijn moeder bijvoorbeeld die snapt dat, die ziet daar totaal het nut niet van in. Als ik met 250 km/uur ergens circuit aan het rijden ben. Overigens mijn zonen, de ene rijdt Quad 0:41:48.8 en de ander rijdt crossmotor, dan ben ik als de dood. Dan denk ik bij mezelf van, joh, verkopen dat ding en nooit meer doen, veelste gevaarlijk, dus dat is dan misschien het ouderinstinct. Maar ja, die behoefte heb ik heel erg om met mijn leven elke dag dingen te doen en ook zakelijk gezien. Ja, waar een ander misschien drie weken over na

moet denken is bij mij gewoon ja of nee. Nee is nee, en als het ja is dan ga ik er ook voor. En ja, dat is wel extreem, ja.

Gesprekleidster 1: En hoe ben jij bezig met je leefstijl, ben jij bewust mee bezig of juist helemaal niet?

M: Jawel, ik ben wel heel bewust leven, die paar sigaretjes nou 's avonds, dat heb ik al verteld. Ja, ik ben daar wel heel bewust mee bezig. Maar ja, ik heb gewoon uitdagingen nodig of spanningen. Ja, dan gaat er soms een knopje om, ja, heb je schijt aan alles.

Gesprekleidster 1: En wat motiveert je om daar bewust mee bezig te zijn? Wat motiveert je om bewust met je leefstijl bezig te zijn?

M: Om uiteindelijk gewoon lang te leven, heel simpel.

Gesprekleidster 1: En hoe is dat voor jullie? Wat motiveert je om juist wel of niet bewust met een gezonde leefstijl bezig te zijn?

V: Ik voel me gewoon beter. Ik weet gewoon, weet je, wat mijn lichaam prettig vindt. En dan doe ik wel eens dingen waarvan ik denk, dat is niet zo goed voor me. Maar dat is niet omdat ik iets slechts eet, maar mijn lichaam reageert..

Gesprekleidster 1: Je beter in je vel voelen ook.

V: Ja, ja ik heb dat heeft eigenlijk niets met dat HNPCCC met het Lynch te maken, maar ik heb gewoon heel snel last van mijn buik dus snel buikpijn en dat soort dingen dat heb ik eigenlijk altijd gehad. Ik ben voor wat dingen allergisch, zeg maar, dan laat je dat wel. Dat is bij mij ook al een reden, maar eigenlijk sla ik nooit een dag groente over ofzo. Ik begin dan met fruit en dan met hazelnoot, ik eet 's ochtends altijd hazelnoten. En fruit vooraf, het is, ik ben gewoon echt zo’n pap kind. Vond het vroeger ook altijd heel lekker, en vooral die rauwe die zelf moet koken. Dan doe ik wel een beetje donkerbruine basterdsuiker op dat vind ik dan wel weer lekker. Daar kan ik van genieten, maar ik eet ook wel een koekje, hoor. En ik drink ook wel koffie, maar ik drink eigenlijk geen melk, maar ja, ook omdat ik daar niet zo goed op reageer. Ja, ik drink dan geitenmelk, iedereen zegt, bah, geitenmelk, maar ja, als je daaraan gewend bent. Ook veel kwark, ook vanwege het sporten, een bak kwark tussendoor met wat walnoten. Ik eet wel zo, ook gewoon, ik het ook lekker..

Gesprekleidster 1: En hoe is dat bij jou bijvoorbeeld?

M: Wel vezelrijk, ik heb mijn darm op drie plaatsen, spastische, mijn darmen aan de rechterkant.. "..''0:45:21.5 aan de rechterkant. Als ik te weinig vezels eet, dan sta ik eigenlijk op de kop, daar vinden ze ook altijd de poliep. Via"..''0:45:36.0 de poliep verwijderd. Twee poliepen verwijderd normaal als ik thuis kom ga ik rustig eten, koekjes, boterhammen met korstjes, beetje licht verteerbaar. Maar het zat me de hele avond niet lekker. 0:45:50.8 Ik heb dat onderzoek in ** en dan mag ik de huisartspost niet bellen, mocht er wat zijn. Toen ben ik 's avond rond een uur of 11 toch maar naar het ziekenhuis gegaan. Want het was echt niet goed, het ziekenhuis eerst gebeld, toen ging ik het uitleggen, kwam ik in het ziekenhuis, werd er gelijk bloed afgenomen. Er werd gelijk bekeken, röntgen foto gemaakt om te kijken of er iets in de buikwand zat. Maar daar zat, dus twee poliepen verwijderd, spastisch gedeelte waar ik meestal last van heb. Dus daarom stond ik op de kop, maar verder is, sla bijna niks over qua eten. "..''0:46:38.4 ".." laten..kinderen moeten het eten dus papa ook "..''0:46:47.7

Gesprekleidster 1: Je moet het goede voorbeeld geven natuurlijk dan. Maar, zou je zeggen dat je bewust met je leefstijl bezig bent, bewust met gezond leven.

M: Van huis uit eet ik altijd groente en fruit. En mijn vrouw kan zo ook, dus ja.. Ze eet ook gewoon fruit.

V: Ik denk ook dat een stukje, net wat je zegt, als je dat meekrijgt, ga je het verder ook zo doen, hè?

Gesprekleidster 1: Speelt wel een rol, hè?

M: Het is bij ons geen magnetron maaltijden, hoor, dus, het is gewoon groente, vers gemaakt. Schijnt tegenwoordig ook heel weinig uit te maken of je nou alles uit pot 0:47:27.6 eet of vers.

V: Ja, het is wat lekkerder, vind ik.

M: Vers? Ja

V: Ja, het is niet zo dat ik nou speciaal biologisch koop ofzo. Dat is dan ook, mensen die zeggen van, nou dat ..

Gesprekleidster 1: En hoe is dat bij jou V2? Ben jij bewust met gezond leven bezig, of houd je daar niet zoveel rekening mee?

V2: Ja, ik ben dus vegetariër. Ik ging op een gegeven moment het huis uit en ja, gezellig in een studentenhuis, dus dan, en toen kwamen de kilootjes erbij. Ik heb ze met hangen en wurgen eraf gekregen. Toen ging ik samenwonen, gezellig dus, bij is het eten altijd wel, ik heb geen geen slechte band mee ofzo, maar ik ben wel een gezellige eter. Ja, dat zorgt dus voor problemen en ik ben helaas niet heel erg sportief dus en dat is toch wel iets waarvan ik in mijn achterhoofd wel heb van daar moet ik wel iets aan doen want, gezond lijf is natuurlijk wel belangrijk, wat je er in stopt, maar ook wat je ermee draagt gewoon.

Gesprekleidster 2: Ja, want je zei ook dat je daarna, nadat je wist dat je Lynch hebt, dat je op zoek was gegaan naar informatie over leefstijl. Heb je die uiteindelijk, je zegt je kwam een hoop zooi tegen, heb je uiteindelijk wel iets goeds gevonden?

V2: Ik ben daar eigenlijk, ik ben op dat moment, was ik gewoon zo erg op zoek naar een leidraad enzo dat ik daar een beetje in verloren ben. Uiteindelijk ook, um, mijn studiegenoot niet lekker, dus dan loop je een aantal dingen gewoon mis. En, um, dus ja, ik heb op een gegeven moment ben ik daar toch wel mee gekapt met te zoeken want je komt steeds dezelfde dingen tegen. En eigenlijk zijn het geen dingen waar je wat mee kunt, want dan kom je bij Moermandieet en dat soort dingen. Zo extreem wilde ik niet bezig gaan, want voor je het weet heb je een hele andere problemen, maar, um, ja, ik eet vegetarisch en ik denk dat met het laten staan van vlees en heel veel bewerkte vlees producten al, um, een bepaalde bron van voedingsstoffen die niet zo goed voor je zijn in ieder geval eruit gooi. En potjes en zakjes en dat soort dingen, ik doe ook altijd vers koken.

V: Ja, dat gebruik ik ook niet, kruiden ook niet, ik kook helemaal niets uit pakjes. Voel ik me ook helemaal niet goed ging, kan ik maar heel zelden, kan ik bij de Chinees wat eten. Als wat van iets vets 0:50:11.6 erop gooien dan word ik doodziek van, krijg hartkloppingen, en dus ik gebruik ook eigenlijk zelf, ik maak ook graag Indiaas en ik vind koken gewoon heel erg leuk. Maar, ik maak zelf dus alle kruiden en masala's maak ik helemaal alles zelf. Dat wel ja, ookal heb je westerse curry gerechten. Nu vind ik eten ook wel lekker, vroeger had ik alleen maar om in leven te blijven. Vond eten helemaal niet, vanaf m'n 18de tot mn 30ste ofzo, ik at wel gezond, omdat ik denk moet die vitamines binnen hebben, maar ik vond eten verschrikkelijk. Ik at echt alleen maar om in leven te blijven. Want paprika's, tomaten, als ik die foto's zie dan denk ik, oh, ja, dan zie ik het wel weer. Dan zie je er ook niet zo gezond uit, hoor. Als je te weinig eet en later heb ik het toch leren waarderen, dat je ook van eten kunt genieten. En ik kon echt vroeger niet van eten genieten en dat kan ik nu wel.

Leefstijl factoren

Gesprekleidster 1: Nou, dat is fijn. Um, ik denk dat wij even naar het lijstje gaan van.. Hij kraakt heel erg, ik zal niet trekken anders horen wij het niet terug. We gaan even kijken naar factoren die jullie leefstijl beïnvloeden. We proberen voor onszelf een overzicht te krijgen van welke dingen nou beïnvloeden of je gezond leeft dus, hè, goed beweegt, gezond eet, en niet rookt en wat voor factoren die daar nou een positieve invloed op heeft, wat er nou voor zorgt dat dat lukt, dat je dat wilt, en welke dingen nou beïnvloeden dat je dat juist niet doet. Dat dat juist niet lukt en niet wilt en dan er zijn al behoorlijk wat

dingetjes zo gaande weg eigenlijk tussendoor wel genoemd. Gesprekleidster 2 heeft die ook opgeschreven en het is de bedoeling dat we straks een beetje een overzicht daarvan hebben. Zouden jullie zo wat dingen kunnen noemen die beïnvloeden of jullie, wat al genoemd is, hè, lang willen leven dus daarom probeer je te letten op een gezonde leefstijl en je goed in je vel voelen dat je ook aan je lichaam merkt, van, nou, mijn lichaam kan eigenlijk niet zo goed hebben als ik bepaalde dingen eet, dus dat is voor mij een reden om te letten op wat ik eet.

V: Ja, dat hoeft niet altijd voor een ander doet om gezond te zijn.

Gesprekleidster 1: Nee, dat is ook zo.

V: Bepaalde dingen nog, wat bij jou niet goed voelt dat wel gezond is en dat voor de ander juist heel goed voelt.

M: Het afgelopen jaar op vakantie ervaren, Zuid-Afrika, daar gaat na de klok van half 7 eten soms half 8 en na het eten gaan mensen naar bed. Als ik ergens niet tegen kan is het.. Na ja, je hebt geen keus, want als het daar donker wordt, dan ja, nachtleven is daar niet. En ja, dan moet je naar bed, met een volle maag, goed, na drie weken daar, heb ik geen fijne buik gehad.

V: Verschrikkelijk

M: En ik zet dat opzij, omdat we graag dingen willen ondernemen, dat we graag dingen zien, maar je bent dan wel elke dag bezig van, oh ja, gaan we zo weer pauzeren ergens, want we hadden een groepsreis gemaakt, kan ik dan ergens naar de wc of dat is wel heel vervelend. Ik zou het liefst dan, ja, dat is wel heel Nederlands, om half 6 willen eten, en kleinere porties en dat gaat goed. Elke, altijd als ik in een restaurant ga eten dan moet ik zo ontzettend opletten wat ik eet. Niet te veel, en ja kwart over 9 's avonds lagen we in bed. Ja, dat gaat bij mij niet goed, maar ja..

V: Ik kan dat sowieso niet, want ik heb natuurlijk traag werkende spijsvertering, dus dan merk je, er moet echt vier uur tussen zitten om dan te gaan slapen dus..

M: Naja, goed, ik zet best veel dingen opzij, maar dan..

V: Maar goed, dat is dan tijdelijk, hè?

M: Als ik na twee dagen weer thuis ben dan is het ook meteen weer over, ja.. Dus ":.."0:55:06.0

Gesprekleidster 1 En dus 0:55:06.6 bepaald ritme

Gesprekleidster 2: Dat vat 'm goed samen?

M: Ja, dat denk ik wel, want eigenlijk sinds dat wij kinderen hebben, heb ik eigenlijk heel veel regelmaat in mijn leven gekregen. En daarom slaap ik denk ik ook. Ja, ik slaap 6,5 - 7 uur maar, elke avond eigenlijk rond dezelfde tijd naar bed ga. En dan ben ik 's morgens eigenlijk altijd rond dezelfde tijd wakker, dus regelmaat is voor mij heel belangrijk.

V: Ik eet heel vaak, heel weinig.

Gesprekleidster 2: Kleine porties bedoel je.

V: 5, 6 keer op een dag en als ik dat niet doe dan word ik even misselijk en de ene keer moet ik soms iets zoets hebben, de andere keer heb ik al heel lang, maar dan voel ik me dan, dus ik eet altijd best wel veel eetmomenten.

Gesprekleidster 1: Oké, we hebben het nou heel veel over eten, hè, maar is nog met betrekking tot bewegen, bijvoorbeeld zijn er nog dingen die we hier in dit rijtje neer kunnen zetten?

V: Ja, bewegen dat is wel, 0:56:13.7 tenminste, met sporten, ja, bewegen is voor mij heel essentieel. Als ik een dag niet kan bewegen, voelt het al anders. Ik heb dat stofje toch wel nodig van dat bewegen.

Gesprekleidster 2: Zelfde als dat goed voelen eigenlijk.

V: Ja

M: Dat is absoluut waar alleen, hè, de ja, mijn operatie ben ik nooit zo intensief heel vaak tot het uiterste gegaan. En als je uiteindelijk die berg op wilt komen, heb ik heel veel voor getraind. En voornamelijk dan op de zondagochtend en dan werd 's ochtends misselijk. Of zondagsmiddag werd ik misselijk en ‘s avonds werd ik helemaal leeg. En um, dus die intensiteit, zo heel intensief, heel vaak gefrustreerd, misschien dat je dan te ver bent gegaan. Wat voor iemand normaal was, 50 km fietsen op een bepaald tempo, ja, daar kwam ik 1,5 jaar geleden niet, hoor. Dan ging ik dood, nu is dat met twee vingers in de neus. Helemaal van de kaart van raken, dat klinkt misschien voor jullie..

M: ".."0:57:32.9 toch?

M: Jawel, maar goed, daar ging het mis met eten en daar zat ik op die wielrenfiets en daar zat je heel erg in elkaar.

Gesprekleidster 1: Dit zit natuurlijk ook helemaal

M: Dat zit niet lekker

V: Ik kreeg zelf fietsles, dus dan weet je dat je naar voren moet gaan en je heupen recht.

M: Ik ben dus uiteindelijk op de mountainbike, de 6 op gegaan, daar zit je niet zo, maar daar zit je zo. Dat was in het begin, daar kwam ik vrij snel achter, dat ik continu, er zat gewoon twee uur lang altijd tot hier.

V: Ja, bewegen was voor mij als kind al een uitlaatklep. Als ik ergens mee zat, of als er thuis, er werd al snel dat we met ziekten en alles te maken hadden. Ging ik als kind..

M: Ja, dat heeft er allemaal mee te maken, maar die beperkingen heb ik nu niet meer met sporten. Moet wel op voeding letten, want ik kan niet te veel eten. Dat als ik mezelf ga volproppen, ja, dan voelt dat niet lekker, maar ik heb nu geen beperkingen. Ik kan echt gewoon tot het uiterste gaan en dan gebeurt er niets met mijn lichaam, dus dat is wel kwestie van trainen.

Gesprekleidster 1: Trainen, ja conditie opbouwen inderdaad. Ja, *stilte*

V2: Ik denk dat aan beide kanten ook wel de verwachtingen zijn die anderen van je hebben, negatief, je bent op een feestje, ach neem nog gezellig een stukje taart.

Gesprekleidster 2: sociale druk eigenlijk

V2: Sociale druk inderdaad

Gesprekleidster 1: En ook ja, aan de andere kant heb je dan weer..

V: Het helpt ook.

V: Maar goed, als je iets, ik neem ook wel eens een vette hap, hoor. Als je er achter staan is het gewoon lekker. Maar je moet niet denken, oh, ik mag het eigenlijk niet, dat moet je niet doen. Maar als je denkt, he, weet je.

M: Als er te voor staat is het niet goed.

V: tevreden

Gelach

V: Dat is dan het antwoord

M: Als je dan net een keer gesport hebt, en ze komen langs met een schaal bitterballen. Dan smaakt het toch heel lekker.

V: en zo is het, toch?

M: Lekkerste biertje drink je na het sporten.

V: Als je dan getennist hebt, nou pilsje, nou, lekker zeg.

M: Nou, dat doe ik dan weer niet 0:59:43.7

V: Als het heel warm is, dan is een pilsje wel lekker.

V: Is goed om het door te spoelen.

Gesprekleidster 1: Zijn er nog meer dingen die er in het lijstje bij kunnen? Bijvoorbeeld wat voor dingen zouden er meespelen om een gezonde leefstijl vol te houden zijn er specifieke dingen waarom het juist niet lukt om het vol te houden? Of waarom het juist wel lukt?

V: Nou ja, als je positief, als je er goed bij voelt en dan heb je dus, ja..

Gesprekleidster 1: Is dat weer een motivatie op zich..

V: Misschien ook voor mensen die iets, misschien wel mensen die vinden, dat ze iets vinden dat ze iets te zwaar, als ze dan wat meer gaan sporten, valt dan ook nog wat af. Dan zeggen ze, oh, nou

Gesprekleidster 1: Dan zie je ook resultaat van wat je doet, zeg maar.

V: Dan zie je ook resultaat.

M: ".."1:00:49.4 meteen een reden om aan te komen

V: Ja

M: ".." 1:00:54.3 zegt van, nou. Ik mag er wel wat bij hebben, maar, ik zeg dat is misschien dan ook wel te veel

V: Maar goed, je hebt ook mensen die heel dun zijn. En die, zeg maar, allerlei slechte dingen eten die toch een hartinfarct kunnen hebben. Die kunnen dus toch dichtgeslibde aderen, dus het is niet altijd aan het.. Mensen die toch wat steviger zijn gewoon hartstikke gezond zijn. Dus.. maar goed dan is het moeilijk als het er niet bijkomt, maar ja, aan de andere kant, je hoeft je ook niet mee te slepen, toch? Of zou je er wel wat bij willen hebben.

M: Ik wel.

V: Ja?

Gesprekleidster 2: V2, jij zei dat je niet zo van het sporten was? Is daar een reden voor?

V2: Ja, ik ben gewoon een ontzettende hark.

Gelach

V2: Ja, dan kan ik er ook niet van maken. Maar dat is al van kleins af aan en mijn broer en mijn zusje hebben dat ook, wij waren van die kinderen, wij gingen liever een boek lezen. Dan dat we buiten gingen spelen, dus..

V: Precies

V2: Dat is voor mijn vader heel frustrerend. Mijn vader is heel sportief, die heeft tot een paar jaar terug marathons gelopen enzo, dus die had daar drie kinderen zitten, die..

Gelach

V2: liever een boek lezen en hij zag ons liever naar buiten dus.. ja, dat maakt het..

Gesprekleidster 1: Je moet het ook leuk vinden om te doen natuurlijk, hè?

V2: Ben nog nooit over die grens gekomen, nou voel ik het leuk.

Gesprekleidster 1: Of je hebt nooit die activiteiten gedaan waarvan je denkt, dat vind ik leuk.

V2: Ja, inderdaad.

V: Maar moet je.. met de fiets, hè, als je ergens naar toe moet..

V2: Jawel, jawel, ik ga altijd met de trein naar mijn werk en dan fiets ik een stukje met de vouwfiets.

V: Nou, dan beweeg je toch ook?

V2: Paar kilometer dus en dat is wel iets. Het is niet dat ik in de auto zit, ik heb niet eens een rijbewijs. Maar het is niet dat ik in de auto stap om boodschappen te doen.

V: Nee, het hoeft ook niet altijd op sportschool te zijn, hè, je kan ook een wandeling maken.

V2: Maar, het mag ook wel wat meer en dan wat minder gezellig eten. Dan denk ik, oh, nog een beetje kaas eroverheen. Of, oh, de crème fraiche erin, dat is ook wel lekker. En..

M: Nee, dat laatste moet je niet doen, inderdaad wel heel lekker 1:02:58.9

V2: Nee, maar dat zijn van die dingen dat is ook wel, um, ik ben er wel heel erg mee bezig, um, tegelijkertijd moet ik natuurlijk ook zorgen dat ik aan eiwitten wel kom en aan calcium en nou bleek het sinds, dat ik ook altijd al gehad, maar ik ben nu wat extremer dat ik ijzertekort heb. Dus ja, dan moet je daar ineens weer aan gaan denken, mag je die combinaties, mag dan weer geen melk bij volkorenproducten, want dat werkt elkaar dan weer tegen, dus dan moet je dat allemaal gaan indelen. Ik denk soms het zal wel eens handig zijn als iemand dat voor me deed.

V: Ja, maar dat is ook wel lastig, als je vegetarisch bent, dan moet je toch lastig dat je met vis en vlees enzo dan heb je wel sneller toch je eiwitten en dingen al binnen.

V2: Ja, en ik mis dan gewoon een beetje de.. soms zou ik zou het handig zijn dat je een soort van, um, hoe heet die vrouw ook al weer Sonja Bakker met hele menu’s enzo, dat ik denk, van nou, dat is handig

Gesprekleidster 1: Kant en klaar

V2: Ik hoef gewoon niet te denken, ik krijg als ik dit eet, dan krijg ik al mijn voedingsstoffen binnen, dat het elkaar niet tegen werkt, dat zou ik wel eens handig vinden. Maar ja, een passende oplossing is er niet altijd.

Gesprekleidster 1: Nee, dat is waar.

M: We hebben fitnessleraren en die dringt heel erg aan om, zeg maar, supplementen, zeg maar, bij te slikken, daar ben ik op tegen. Überhaupt op elke manier van pillen slikken en ook..

V2: Goede voeding is dan niet nodig.

M: Nee, maar hij zegt dan van, joh, alle groente en fruit die wij tegenwoordig in de winkel kopen, daar zit die, wordt ook veel te vroeg geoogst, 1:04:34.7 ziet er alleen maar mooi uit. Daar zitten bijna geen vitamines meer in. Hij is daar zo van overtuigd dat de kwaliteit van groeten en fruit eigenlijk gewoon helemaal niet meer goed is. Dus ja..

V: Ja, maar de pillen blijkt ook, bepaalde stoffen in zitten.

M: Ja, maar ik slik ze ook niet, hoor. Ik vertik het, maar.. ja, ik eet ook elke dag 2,3 stuks fruit en altijd groente ja, daar houd ik het dan wel bij. Maar goed, ik vroeger als ik ergens in het buitenland ben, hè, sinaasappel daar wel lekkerder is als in Nederland in de supermarkt. En als we dan gingen kijken, mijn dochter had dan in Afrika, die had een een kipspies besteld, dat ik dacht van goh dat is lekker. Dan weet je dus van eigenlijk recht voor zijn raap wat voor troep wij in Nederland gewoon te vreten krijgen. Ja, dat wordt wel duidelijk. Dat is gewoon allemaal opgespoten, met hormonen, maar ja, kun je er iets aan doen? Nee, ik kan er niet zoveel aan doen. Ja..

V: Je eigen kippen houden.

M: Ja, dat zou kunnen

V: Ja, waar haal dan weer voedsel uit? Die zijn van scharrelkippen, die halen ze ongeveer uit, dan zeggen ze wel biologisch, maar kippen, dan moet die grond ook weer goed zijn, hè. Soms is de grond ook helemaal niet goed. En die eet je, dan is het, wel de kippen zelf maar ook weer met die eieren, dus ja, valt ook niet, valt zo veel voor te zeggen.

M: Scharrelkippen die worden ook bij gevoerd ook met ".."1:06:07.5 alleen ze lopen vrij rond ja, dan heb je scharrel ook niks

V: Ja maar, dat zeg ik, dan heb je scharrel kip, maar dan weet je ook niet wat uit de grond komt.

M: Ik denk dat het meer ligt aan hoe snel ze groeien, ja, en dan denk je die groeien gewoon rustig op. Ik bedoel.. Ik zie stress niet staan.

Gesprekleidster 1: Kijk, dat is een hele goede. Dat is een hele goede, ja. Zijn er nog andere dingen die wij missen?

M: Rust

Gesprekleidster 1: En dat moet dan juist aan de plus kant denk ik.

M: Ja rust, ontspanning

WCRF Aanbevelingen

Gesprekleidster 1: rust, ontspanning. Anders gaan we eventjes, ook gezien de tijd eventjes naar de aanbevelingen toe. Die hebben we zo mooi daarover heen gevouwen.

M: Is dat vanuit jullie kant of?

Gesprekleidster 1: Nee, nee, dit zijn aanbevelingen vanuit het WCRF, hebben jullie daar van gehoord? World Cancer Research Fund? Of het Wereld Kanker Onderzoeksfonds? Dat ik zal eerst wat achtergrond..

V: Ik heb daar ook wat recepten van gekregen.

Gesprekleidster 1: Nou, oh dat is, nou

V: Die zijn wel heel lekker receptjes, alleen daarna heb je wel wat sneller trek weer. Maar wel lekker, ja

Gesprekleidster 1: Nou, dit zijn leefstijlaanbevelingen ter preventie van kanker. Um. Die leefstijlaanbevelingen zijn gebaseerd op onderzoek die gedaan is, ook bij het WCRF. We lopen ze zo nog even door. Ik heb er ook wat dingen bijgezet, we hadden ze niet heel specifiek opgeschreven. Ik zal er zo doorheen lopen. Het is, houden ze eigenlijk het wetenschappelijk onderzoek wat er verschijnt over voeding en bewegen en allerlei leefstijl factoren en het ontstaan van kanker. Dat houden ze daar bij, dus dat verzamelen ze. Je hebt allerlei databases waar onderzoek in verschijnt. En daar zoeken zij uit en 1 keer in de zoveel tijd maken ze daar een update van. En ze gaan op basis van onderzoek wat zij ophalen kijken ze in hoeverre er wetenschappelijk bewijs is, dat er bepaalde voedingsmiddelen zijn die bijdragen in het verlagen van het risico op kanker. En dat, op basis van de literatuur hebben zij leefstijlaanbevelingen gedaan, die leefstijl aanbevelingen die daar uitkomen, zoals jullie hopelijk kunnen lezen, lijken die leefstijl aanbevelingen heel veel op de leefstijl aanbevelingen die er ook zijn ter preventie van hart en vaatziekten en diabetes. Dat heeft er heel veel mee te maken, de enige die je ziet staan die echt op het ontstaan van darmkanker met name gericht, is het eten van minder rood vlees en geen bewerkt vlees. Dat is de enige specifiek op het ontstaan van darmkanker gericht is ook.

M: Wel een vreemde.

Gesprekleidster 1: Welke?

M: Die rood vlees.

Gesprekleidster 1: Hoezo is dat een vreemde?

M: Um, ik laat me regelmatig bloed prikken en kijk vervolgens hoe hoog mijn cholesterol is en de aanbeveling van de huisarts was als je cholesterol te hoog is qua vlees dan moet je altijd eten wat zwemt, vliegt, rent al het vlees dat beweegt, dat is juist rood vlees. Varken beweegt weinig dus varkensvlees is slecht.

Gesprekleidster 2: Maar varkensvlees is ook rood, hè, dat is niet alleen het vlees dat rood is na het bakken maar rood is voor bereiding.

Gesprekleidster 1: Wat rauw roodkleurig is. Dat wordt bedoeld hier.

Gesprekleidster 2: Dus geen kip.

Gesprekleidster 1: Is dan inderdaad kip, kalkoen zijn inderdaad de uitzonderingen. En verder rundvlees en varkensvlees dat is ..

V: Is wel voor je bloed, dat rood vlees, dat klopt maar er werd wel gezegd bij mij voor de darmen was dat het beter om geen rundvlees te eten, maar wel weer wild. Dat is ook al rood, zoals herten, biefstuk..

M: Ja maar, dat klopt dan wel voor je cholesterol, maar op zich rundvlees..

Gesprekleidster 1: Maar het zijn ook twee verschillende dingen, hè, ter preventie van kanker of voor je cholesterol. De adviezen kunnen elkaar af en toe ook bijten, hè, dat komt ook wel een beetje uit punt 6. Liever geen alcohol, als je kijkt naar het wetenschappelijke bewijs wat er is voor alcohol consumptie en het

bestaan van kanker dan blijkt daaruit dat je eigenlijk beter helemaal geen kanker moet drinken, maar als je kijkt naar literatuur die er is voor het drinken van alcohol en het ontstaan van hart en vaatziekten, dan zeggen ze juist weer rode wijn een glaasje is juist weer heel goed, met mate, dus maximaal 1 glas voor een vrouw, maximaal twee glazen voor een man. En die aanbevelingen zijn eigenlijk overgenomen, dus puur als je kijkt naar literatuur van het ontstaan van kanker, zeggen ze eigenlijk liever helemaal niet, maar het is ook raar aanbevelingen aan te geven dat goed is voor het 1 en slecht is voor het ander. Dus daar hebben ze rekening mee gehouden en meer algemene aanbevelingen, dus dat is ook wat elkaar een beetje wringt inderdaad. En verder als ik even gewoon bij het begin begin, geen overgewicht hebben, normaal gewicht, let erop, geen ondergewicht. En dat betekent een BMI tussen de 18 en 24.9, zal niet iedereen iets zeggen maar, hè, iedereen weet waarschijnlijk wel een beetje wat, of je een normaal gewicht hebt. En een slanke taille, de vetverdeling is ook van belang en waar het vet, met name het vet dat om je buik zit is nadelig. Nadelig ook voor het ontstaan van hart en vaat ziekten.

V: Dat is moeilijk natuurlijk, of je een peer of een appel bent. Peer, gaat het op mijn billen zitten heupen dat blijkt geen verkeerd vet te zijn. Mensen die een appel figuur hebben, vrouw ja, borsten en als ze wat dikker worden gaat het hier en dat is dan net verkeerd vet, dus het is ook een beetje wat je meekrijgt, hè.

Gesprekleidster 1: Dat is ook zeker zo, ja. En nou, daarna staat een aanbeveling over bewegen, iedere dag 30 minuten, dat is de beweegnorm, die geldt ook voor preventie van hart en vaatziekten. En diabetes. Eet minder calorierijk en weinig suikerrijke dranken dat heeft ook weer te maken met aanbeveling 1, ter preventie van overgewicht. En eet veel groente en fruit, minimaal 5 porties per dag. Dat heeft met name ook mee te maken, het is bekend verschillende kankersoorten, dat hoe meer fruit en groente je eet hoe lager de kans op het ontstaan van kanker. Maar we weten eigenlijk nog niet in welk stofje het zit, dus daarom zeggen ze varieer zo veel mogelijk zodat je in ieder geval dat stofje binnen krijgt.

V: "..''1:14:06.4

Gesprekleidster 1: Precies, ja, en dat geldt ook voor volkoren producten, peulvruchten dat zijn ook allemaal vezels natuurlijk die daar in zitten. En dan krijg je de rood vlees aanbeveling, daarvan wordt gezegd maximaal 500 gram per week, dat is het vlees dat rauw rood uitziet. En geen bewerkt vlees, dat zijn vleeswaren waar allerlei toevoegingen zijn gedaan om het langer houdbaar te houden ook.

M: maar dat heb je niet voor het uitkiezen.

Gesprekleidster 1: Hoe bedoelt u?

M: wat niet bewerkt is,

Gesprekleidster 1: Ja..

Gesprekleidster 2: Vleeswaren, valt bijna alles onder inderdaad.

M: Maar, wat zeg je nou over vleeswaren, kon het niet helemaal goed verstaan.

Gesprekleidster 2: Heel veel vleeswaren zijn bewerkt, dus inderdaad wat je zegt, het is wel lastig

M: Als je naar een normale slager gaat of je gaat naar een supermarkt ik denk dat dat weinig ..

V2: Maar bewerkt is denk ik ook kroket, frikadellen en al dat soort dingen waarvan je eigenlijk niet wil weten wat er in zit.

V: Nou dat valt eigenlijk wel mee, het zijn met name de worstproducten ..

M: resten van vetten

V: Dat is meer..

M: Ook als je rosbief of ham of wat dan ook?

V: Nou rosbief is dan minder weer.

M: rauwe ham en dat soort is dat ook?

V: Dat is ook minder.

Gesprekleidster 2: Meeste is inderdaad wel bewerkt

M: Wat bedoel je met bewerkt dan? Je hebt ook gekookte ham.

Gesprekleidster 2: Nou ham, daar zit, ik weet niet precies welke

Gesprekleidster 1: Je kunt het ook vaak op het etiketje zien, hè, als je bij een supermarkt bijvoorbeeld vleeswaren koopt, dan staat daar ook een etiketje met ingrediënten, en als daar veel spullen bijstaan bijzitten om het allemaal goed te houden, dan is dat dus bewerkt. Dat is ook toegevoegd om het vlees goed te houden.

V: En je kunt op internet wel zien, de E daar heb ik ook wel eens naar gekeken. Dat is dan weer minder slecht of en als er bepaalde producten waar kleurstoffen in zitten, het is niet meer vleeswaren, dan kan het wel, dan zijn het natuurlijke kleurstoffen. Daar heb je dan veel dingen wat bewerkt is, maar vleeswaren, heel veel vleeswaren. Maar bijvoorbeeld, rauw ham enzo, dat minder is omdat het gedroogd wordt door de zouten.

Gesprekleidster 1: Nou, dat is meteen de volgende, minder zout dat is ook een aanbeveling, overigens niet zo dat al deze aanbevelingen slaan op alle typen kankersoorten maar er is natuurlijk een algemene leefstijl aanbeveling gedaan. Op, en punt 8, dat is de, eigenlijk, probeer je voedingsstoffen uit je voeding te halen en niet uit voedingssupplementen, dat is de uiteindelijke aanbeveling. Hè, dat, de gedachte daarachter is dat het niet bewezen is dat voedingssupplementen ter preventie van kanker..

V: Ja, want als je veel eet en je hebt al die voedingssupplementen, die moet ook afvalstoffen moeten er ook weer uit. Dus dan moeten de nieren enzo veel harder werken.

Gesprekleidster 1: Er zijn veel verschillende, literatuur, op sommige momenten kan het zelfs heel nadelig zijn voedingssupplementen te gebruiken, bijvoorbeeld als je chemotherapie krijgt, dan wordt het echt afgeraden, dan kunnen de voedingssupplementen gaan vechten met de chemotherapie.

V: En veel water drinken, hè, veel vocht, waaronder koffie is geen vocht. 1:18:38.0

V: Maar ik tel er 9, waar is de, ergens staan er dan twee in denk ik.

Gesprekleidster 2: Dit zijn de 8 algemene aanbevelingen er zijn ook twee specifiek, hebben wij niet genoemd want de ene is gericht op mensen die kanker hebben gehad en daarvan zeggen ze, we hebben geen specifieke aanbevelingen voor die groep. Maar volg gewoon deze algemene ter preventie van kanker en er is er nog eentje voor zwangere vrouwen. De relatie van het geven en ontvangen van borstvoeding is de tiende. Wij dachten, we houden het bij de 8 algemene aanbevelingen, 2 specifieke kunnen we toelichten.

V: Als kindzijnde toen mij vader dus kanker kreeg, dat hij wel een dieet kreeg vanuit het ziekenhuis. Die moest veel groente en juist wat minder eiwitten, die kreeg dus toen een dieet.

Gesprekleidster 1: Was dat ter voorbereiding op een operatie?

V: Nee, hij had al een operatie gehad. Die had helemaal geen behandeling, alleen bestraling. 1:20:01.9 Maar die kreeg wel een, maar ja, dat kwam ook vanwege stoma, hè.

Gesprekleidster 1: Ja

V: Dan heb je dieet en geen koolzuur en dat soort dingen.

Gesprekleidster 1: En natuurlijk niet roken, concentreert zich met name op leefstijl, anders dan roken dat hoort er ook extra bij.

V: Ik heb ook nooit gerookt, vroeger ja, iedereen rookte bijna hè

Gesprekleidster 1: Als jullie dit rijtje zien, waar jullie op de hoogte van deze aanbevelingen? Specifiek ter preventie van kanker.

V: Ja, natuurlijk, ook omdat ik zelf fitness, mijn fitness instructeur doet veel met voeding en dat is mij wel bekend.

M: De enige die mij verbaasd, is het rood vlees.

Gesprekleidster 1: Daar was u nog niet van..

V: Er staat minder, hè, er staat niet dat je geen rood vlees mag.

M: Er staat wel maximaal 500 gram en als ik dan kijk dan kom ik daar met gemak aan. Hoef ik geen moeite voor te doen.

M: Ik denk dat het toch te merken is aan *restaurant, zat porties eten vlees eet minder groot waren*. Dus 250 gram, ja, joh, daar kan ik drie dagen van eten.

Gelach

M: Ja, die krijg ik wel weg, hoor dat is niet zo'n probleem.

M: Maar, ik zie, vis zie ik niet staan, um, maar niet alle vissoorten zijn gezond, of wel?

Gesprekleidster 1: Wat hier staat, dat zijn, dat is gebaseerd op de leefstijlgerelateerde factoren die veel zijn onderzocht, er staan hier dingen niet bij zoals zuivel bijvoorbeeld, of inderdaad vis en dat is dan onvoldoende onderzocht of er zijn teveel resultaten die met elkaar in tegenspraak zijn. Op basis daarvan niet echt aanbevelingen kunnen doen, dus daar doen ze geen uitspraak over.

Gesprekleidster 2: Dit zijn de meest overtuigende bewijzen die er zijn. Die hebben ze gebundeld over de rest volgt misschien later.

V: ".."1:22:38.0

Gesprekleidster 1: Dat zou kunnen.

V: De laatste keer dacht ik ook dat ik met 1 meegedaan hebt ook aan het onderzoek, jij had ook meegedaan? 1:22:54.3 derde keer ofzo in 4 jaar. Ik doe iedere keer mee en dan krijg je dus na zoveel tijd, jaren krijg je weer een stapel "..1:23:03.8"

Gesprekleidster 1: En is dat dan vanuit de GeoLynch studie, of dat weet u niet?

V: Ja

Gesprekleidster 1: Ja, jawel

V: Kreeg ik ook een kerstkaart van mensen die daar werken omdat we er zo lang aan meedoen.

Voldoen aanbevelingen/Aanpassen leefstijl:
Gesprekleidster 1: En als jullie naar dit rijtje kijken, in hoeverre, ja voldoet daar aan, in hoeverre zou u daar iets aanpassen of juist niet?

Gesprekleidster 2: Misschien voldoe je er niet aan en vind je het eigenlijk wel prima.

M: Die onderste dat is heel makkelijk, want die is al klaar.

Gesprekleidster 2: U heeft wel gerookt?

M: Nee, ja weet je, 1 en 2 daar ben ik wel mee bezig, eigenlijk houd je dat allemaal een beetje in de gaten. Ik ben er niet bewust mee bezig maken. Ik probeer niet te overdrijven, aanpassen, ja ik weet het niet. Er zijn een aantal dingen daar voldoe ik wel aan.

Gesprekleidster 1: Hoe is dat voor jou, M2?

M2: Als je dat zo ziet, geen bewerkt vlees eten, die is wel lastig.

Gesprekleidster 1: Kan je uitleggen waardoor dat dan..

M2: Ja, ik wil graag vleeswaren eten, met name op brood.

Gesprekleidster 2: Is dat misschien ook gebrek aan goede alternatieven, wat doe je anders op je brood bijvoorbeeld.

M2: Hagelslag?

Gelach

M2: Ja, ik neem altijd brood mee naar het werk, ja, appelstroop wordt een zooitje, hagelslag. Ik eet ook roggebrood met kaas en dat bewerkt vlees. Ja, ik wil gewoon graag vlees op brood hebben. Ik verbrand ook aardig wat op een dag, dus.

Gesprekleidster 1: Hoe is dat voor jou, als je zo naar dat rijtje kijkt zijn er dan dingen waarvan je zegt, dat zou ik willen aanpassen?

M: Ja, de onderste nog, hè, voor de rest voldoe ik wel 100% aan dat rijtje, ja.

Gesprekleidster 2: Wil je het ook stoppen, het roken?

M: Ja.. ja tuurlijk.

V: Niet hard genoeg denk ik.

V: Dat doe je alleen maar ’s avonds, verder niet? Overdag niet?

M: Zelden in het week rook ik niet, maar ja, weet je, wat het is, dan rook je toch 2 of als je een keer wel naar de kroeg gaat 2 of 3 pakjes in de week is gewoon te veel. 1 pakje in de week zou ik nooit stoppen. Maar, dus ik ben niet degene, zeg maar, die 1,5 pakje per dag rookt, zit hier ook niet trillend aan tafel ofzo, zo spannend is het ook allemaal niet. Maar, als ik straks thuis ben na een glas wijn, ja, dan ja, dan vind ik dat altijd wel prettig. Heel veel mensen roken als ze stress hebben, wat ik altijd een bijzonder prettig moment vind, als bijvoorbeeld de stress gedaan is of je hebt iets cools gedaan of iets spannends om daarna dan een sigaretje te roken.

Gesprekleidster 2: Om te vieren met een sigaret eigenlijk.

M: Ja, misschien is vieren het juiste woord, ja.

Gesprekleidster 1: En heb je wel eens geprobeerd om te stoppen?

M: Jawel, ik rook ook niet van mijn 13e of 14e, eigenlijk heel laat pas, vanaf mijn 22ste ofzo 23ste misschien. En nooit geen pakje per dag gerookt ofzo, maar ja, voor mij is het altijd een heel ontspannen moment van de dag.

Gesprekleidster 1: En wat zijn dingen die je tegenhouden om te stoppen met roken? Of waardoor zou het niet lukken denk je?

M: Het is gewoon lekker.

M: Ja, zeker niet vies, stinkt altijd, maar goed, ik rook niet binnen, ik rook niet in de auto of ..

V: Lijkt me helemaal niet gezellig om buiten nog, kun je net zo goed stoppen..

M: Ja, je mag binnen toch niet meer roken.

V: Nee

M: Dus dan staat toch iedereen buiten.

M: Misschien is het net een verslaving, hè

Gesprekleidster 1: Ja het is een verslaving.

M: Het is een verslaving, dus dat is wel lastig.

V: Dat bleek bij mij ook de grootste boosdoener te zijn, allergie van rook. Konden ze eerst niet achter komen, toen kreeg ik in die tijd al die prikjes en ook uit gassen. Maar vooral rook, vroeger naar een café ging of disco was ik gewoon twee dagen hartstikke ziek. Was zo ziek en het bleek dus door rook te zijn en het gekke is, ik heb nooit een vriendje gehad die rookte, want op één of andere manier zat er bij mij ingebouwd als ik dan verliefd op afstand, zeg maar even, en dan was het bijna zover en dan was het over, want dan rook ik dat en dan gaat bij mij. Ik werd er een beetje agressief van.

Gelach

V: Maar, ik had het later onder controle, omdat ik toen wist waarom doe ik nou zo lelijk tegen, het is toch een aardig iemand ofzo. Maar of 1 of andere manier heb ik nooit iemand, dus bij mij is er eigenlijk nooit iemand, 25 jaar is er nooit iemand gerookt bij, want als ik er dus benauwd van krijg werd er bijna astmatisch van, ja.

Gesprekleidster 1: Ja, dat is vervelend. Hé, hoe zit het voor jou, V2? Als je kijkt naar het lijstje, zijn er dingen waarvan je zegt, dat zou ik wel willen aanpassen, of juist niet?

V2: Het gaat er bij mij met name om, ik moet gewoon meer bewegen, want qua eten, ik houd van lekker eten. Maar ik zie ook voldoende mensen, ik werk op kantoor en dat is geen compliment natuurlijk, maar daar hoor ik niet bij de dikke mensen. Ik bedoel, er wordt continu naar binnen gestopt en dus op zich valt het nog wel mee, maar het bewegen erbij is wel heel belangrijk. Ik denk als ik gewoon echt goed beweeg dan ben ik er en dan punt 1 en 2 in 1 keer gehad. En ik denk als je daar bewust mee bezig bent met die andere, zou voor mij meer een ondersteuning zijn, dan de leidraad. Waar ik zelf op dit moment zelf voor wil oppassen, toen ik net vegetariër was, was ik er best wel strikt in. Ik heb ook wel eens ruzie gemaakt met mensen en ik wil niet meer zo strikt worden met zoiets, omdat het je gewoon beperkt in sociale contacten enzo. Dus, um,

ja, dan ben je soms ook wel een stuk makkelijker ofzo, denk dat het dat is. En misschien wat makkelijker, dat taartje dat kan nog wel, dat soort dingen. Dat heb je met kantoor, hè, er is altijd wel iemand jarig. Dus..

V: Maar houd je nog van lezen?

V2: Ja

V: Ik ken mensen die dus fitnessen, die een boek lezen en fietsen.

V2: Ja, dat maakt niet uit, dan moet je nog steeds fietsen

Gelach

V: Maar dan heb je er geen erg in.

V2: Nee, ik heb echt een ontzettende hekel aan zweten. Dat vind ik heel naar. Maar ik ga binnenkort echt, echt weer beginnen met aqua jogging. Dan zit in het zwembad en dat heb ik ook al een keer gedaan.

Gesprekleidster 1: En wat is dan voor jou dat je zegt, ik ga toch, ik vind het eigenlijk niet leuk om te doen bewegen. Maar je gaat het wel doen of je gaat wel weer bewegen. Wat motiveert jou dan?

V2: Omdat het beter voor me is, omdat ik vanuit 2,5 jaar geleden weet, ben ik gewoon in een 1/2 jaar 10 kilo afgevallen. Omdat ik volgens de schijf van 5 ging eten en ook meer ging bewegen, dan gaat het gewoon goed. En dan haal ik dat doel en nu blijkt als ik dat een beetje loslaat dat het dan makkelijk is om het helemaal los te maken en dan komt dan ook nog bij, ik ben ruim een jaar 1,5 jaar geleden naar *ZKH5* verhuisd. Dan moet je je hele sociale leven weer opbouwen, ja, dat heeft ook gewoon even geduurd voordat dat gaat. En dan, ja, dan is het gewoon helaas dat je bepaalde dingen wel laat schieten.

V: Ja, je moet het maar leuk vinden, anders krijg je daar stress van.

Gesprekleidster 1: Dat is heel belangrijk inderdaad, je moet het leuk vinden om te doen.

V2: Ja klopt, en we gingen samenwonen en dan wil je gewoon met je liefje op de bank zitten. En ja, dan moet tegelijkertijd je sociale leven opbouwen, hoewel het maar 20 km van ** verwijderd is. Dat vraagt wel iets, dan is het soms wel even moeilijk om dat weer een beetje op peil te krijgen en andere dingen tussen door komen, zieke vaders en schoonvaders. Dan is een snelle hap te dichtbij.

Gesprekleidster 1: Is het dan ook een kwestie van tijd, dat je bezig bent, dat andere dingen, doordat je te weinig tijd hebt om juist, voor dat bewegen..

V2: Klopt, en verwachtingen, want je moet op je werk, goed je werk doen en tegelijkertijd moet je een goede vriendin zijn en voor je vriendinnen die problemen hebben, bied je ook een luisterend oor en familie wil je voor zijn. Dan is soms makkelijk om jezelf te vergeten. Het motortje dat brandt.

Gesprekleidster 1: Tijd voor jezelf hebt ..

V: Kan ook een excuus zijn, hè

V2: Ja, klopt dat is altijd een excuus.

".."1:34:09.5

M: Ik heb ook altijd te weinig tijd. Hoe ver moet je naar je werk? 1:34:19.0

V2: Niet zo ver, maar ik ga met de trein en de vouwfiets en het neemt toch wel een uur ruim een uur onderweg.

M: En hoeveel km's?

V: 20, ruim 24, kun je net niet fietsen

M: Wel hoor, half uurtje

V2: Ik niet

Gelach

V: Je moet heel rustig je hartslag erachter houden

V2: Ja maar, als ik 24 km moet fietsen, moet ik nog een uur eerder van huis weg.

M: Dat is maar de vraag, als je nu je zegt, je bent een uur onder weg met de trein en met de vouwfiets. Ik zeg jou als je een uur met het OV en met dat fietsje dan met de fiets sneller bent dan..

M: Met een goede fiets ben je drie kwartier onderweg.

".."1:35:08.6

M: Op een gegeven moment, ik moest gaan trainen en ik heb ook tijdsgebrek. Ik heb een groot eigen bedrijf, drie kinderen, veel te veel hobby's, veel te veel sociale contacten maar ja, je geeft je commitment aan iets en ik denk, ja, er blijft maar 1 ding over. Met de fiets naar het werk, voor mij is dat 27 km, en dan ga je daar gewoon op de fiets en dan blijkt uiteindelijk met de auto in het meest gunstige geval doe ik er een half uur over. Maar als je file hebt, doe ik er ook wel eens drie kwartier over, in het ergste geval een uur. Ik kan 27 km, dat fiets ik in 50, 52 minuten en dan blijkt, uiteindelijk heb je een effectieve trainingstijd gehad. En je gezinsleven lijdt er niet onder, de kilo's vliegen eraf en ik kan het ook niet elke dag, hoor.

V: En je hoeft niet te koken, hè, als je thuis komt?

M: Nee, ik hoef niet te koken.

Gelach

V2: Dat is het. Weet je mijn vriend werkt in het ziekenhuis, dus heeft hij wisselende diensten. Dan denk ik, oh, dan is die in de late dienst, kan ik eindelijk weer eens friet eten. Heel slecht natuurlijk.

M: Van de andere kant, we hebben het over een wielrenfiets, mountainbike of weet ik veel wat. Een andere vriend gewoon uit nood, die hebben 1 auto en ze konden geen tweede betalen die hebben een elektrische fiets gekocht en die gaat, nou die moet ook elke dag hetzelfde stukje ongeveer, en die gaat 3 dagen in de week met een elektrische fiets. En dan ben je wel aan het bewegen en dat gaat een stuk makkelijker. Je wordt..

Gesprekleidster 1: Je hebt minder zweet.

V: Als je keihard moet fietsen en je komt bezweet aan op je werk, dat is natuurlijk ook niet prettig.

V2: Nee

M: Nou dat..

V2: Dan moet je nog een half uur eerder, dan kan je daar douchen.

M: Maar een elektrische fiets, die kosten tegenwoordig 1500 euro ofzo en dan heb je toch je bewegen en heb je niet het gevoel dat je uitgeput bij je werk aankomt.

V2: Maar, ik heb geen 1500 euro.

M: Nou ja, fietsplan, ik zeg tegen jou..

V: Ik ben in vaste dienst, ja, ik heb altijd wel een smoesje voor, hoor.

Gesprekleidster 1: Maar, ik hoor je ook zeggen, als je dan fietst naar je werk, dan pas je het tegelijkertijd ook in je leven. Of dan vind je een manier waarop je dan, ja..

M: Je moet het voor jezelf willen. Uiteindelijk als je daar naartoe gaat het kost je tijd, je ziet niet op de bank je gaat er naar toe, je sport een uur, je gaat naar de douche en je gaat op de bank zitten en je denkt van, ja, het was wel lekker.

M: Gewoon leeg zijn.

V2: Het is ook belangrijk dat fitness bij je past.

M: Ik haal daar voldoening uit, ik sta daar tussen, ik ga met een neefje van mij die is ongeveer de helft van mijn leeftijd. Ik kan dan meedoen, dat ik denk ik van nou, ik ben de oudste lekker. Ondertussen zie ik een paar afhaken en dan denk ik, zie je het gaat best nog wel goed.

Gesprekleidster 2: Maar dat zijn persoonlijke verschillen inderdaad.

M: Je moet ergens je motivatie uithalen

V: Ik geef bijvoorbeeld, ik ben 64 ik word 65, ik geef nog 3 keer spinning in de week. Drie keer in de week yogalessen en pilates lessen, ik geef voor senioren geef ik 3 keer in de week gym. Ik geef zo'n 12, 13 lessen.

M: Dat heet ook verslaving dan, hè?

Gesprekleidster 1: Een positieve verslaving haha

V: Ja, maar goed, het is ook mijn werk meteen erbij, fitnessdienst. Maar ja, mijn man zei, oh, het is donker, ik breng je wel even. Ik kom uit *** is niet zo ver, ik pak toch zeker niet de auto om van hier naar het *ZKH5* te komen. Ik ga op de fiets, ik ben nu ook op de fiets. Naar *ZKH5* grote stukken, ik ga ook op de fiets. Als ik naar *** zou moeten, ga ik ook op de fiets. Ik ben nooit zo .. dat vind ik gewoon lekker. Ik heb wel gezegd te weinig bewogen, eigenlijk wil ik er even fietsen, dan ga ik naar ** ofzo weet je. Dan heb ik toch mijn beweging gehad, ja, dat is ook verslaving.

M: Uiteindelijk werkt het wel

V: Maar goed, wat ik ook zei, voor mij is beweging altijd essentieel geweest, als kind bewoog ik al zoveel, voor mij was dat een uitlaatklep. Ook als ik verdrietig was, ik ging als kind maar rennen. Zo kon ik het kwijt raken, voor mij was bewegen, voor jou lekker rusten op de bank, zo zijn mijn kinderen ook, hoor.

Gesprekleidster 1: Hé, gezien de tijd, sorry

Gesprekleidster 2: Mag ik nog 1 vraag stellen?

Gesprekleidster 1: Ja, tuurlijk

Gesprekleidster 2: We hebben het nu steeds over beweging, maar ik hoorde ook een paar mensen zeggen, ik wil rood vlees minderen of bewerkt vlees minderen, stoppen met roken. Zijn er bepaalde dingen waarvan jullie zeggen, dat zou handig zijn, of dat zou mij helpen om ervoor te zorgen dat ik dat punt kan aanpakken?

V: Ik ben wel een beetje een zoetekauw, stuk chocolade is ook wel lekker. Naja, dat doe ik ook niet elke dag, ja, en een glaasje wijn, dat vind ik toch wel, wat jij dus zegt, wil ik ook wel 's avonds even 1 glaasje, ook niet elke dag.

Gesprekleidster 2: Maar zijn er dingen die ervoor zouden kunnen zorgen die jou erbij zouden kunnen ondersteunen om te stoppen met roken, of is het iets waarvan je zegt, dat pak ik zelf aan?

M: Ik zou wel willen weten waar je dan, in de ogen van de wetenschappers, waar je dan het beste voedsel zou kunnen koken.

Gesprekleidster 2: Dus de informatie eigenlijk

M: Ik ben, ik woon in een klein dorpie en wij hebben 2 supermarkten, maar ja, goed, ja, de onbewerkte vleeswaren, of de biologische kip of weet ik veel wat. En we kunnen het op zich makkelijk betalen, bij ons in de buurt niet. Ik zou wel willen weten van, goh, ik heb er best voor over, als ik zeker weet, dan heb ik goed voedsel, want dan heb ik er een half uur voor moeten rijden of weet ik veel wat. Dat maakt dus wel uit, als ik er meer voor moet voor betalen, maar daar zal ik behoefte aan hebben.

V: Zelf brouwen?

M: Ja maar, goed ik heb al zoveel hobby's..

Gelach

3 Belangrijkste factoren

Gesprekleidster 1: Ik wil een beetje gaan afsluiten. Ik wil nog 1 keer naar het lijstje toe ter afsluiting, nog 1 keer iedereen het woord geven en ik zou heel graag van jullie willen weten wat voor jullie de 3 belangrijkste factoren zijn die jullie leefstijl beïnvloeden. Ik willen jullie vragen om 3 punten eigenlijk te noemen, van dat lijstje te noemen, kan ook een punt zijn wat nog niet op het lijstje staat. Wat zijn voor jullie de 3 belangrijkste dingen die jullie leefstijl beïnvloeden? Mag ik bij jou beginnen?

V: Ja nou, in ieder geval goed voelen en ik ben een hoop, zeg maar, vooral ook als ik zelf merk dat ik iets te zwaar zou worden, dan stop ik ook onmiddellijk, er wordt gewoon 2 weken 3 weken niet gesnoept. Ik ben toch wel erg op figuur dat je slank blijft. Dat vind ik belangrijk.

Gesprekleidster 2: Uiterlijk staat er niet bij.

Gesprekleidster 1: Aan je zelf zien dat je uiterlijk verandert en op basis daarvan aanpassen.

V: Natuurlijk ook vanwege gewoon dat je dus net wat je zegt lang willen leven. Ja.. het gaat mij vooral om het goed voelen en het .. regelmaat ja..

Gesprekleidster 2: Lang willen leven, ik hoor regelmaat, wat zal ik als derde er bij zetten?

V: Eigenlijk is het voor mij dat je je goed voelt

Gesprekleidster 1: Dan maken we er gewoon 2 van. En M1?

M1: Bovenste, lang willen leven dat is toch 1 van de doelstellingen. Mijn rust en ontspanning pakken. En uiteindelijk als totaal resultaat mij goed voelt.

Gesprekleidster 1: M2?

M2: Mezelf goed voelen, iets meer rust en ontspanning, iets meer conditie opbouwen

Gesprekleidster 1: Staat bij volhouden

M: Ik heb er maar twee, lang willen leven en niet te..

V2: Bij mij goed voelen, en um, bij mij is lang willen leven, maar zo lang mogelijk gezond willen zijn. Kijk als ik op mijn 75ste poef neerval dan en ik ben niet ziek geweest, dan is het voor mij veel waardevoller dan ik 93 wordt en aan alle kanten open ben gemaakt al eens een keertje.

V: Definiëring

Gesprekleidster 2: Is dat voor jou ook zo?

Meerderen: ja, ja gezond leven. Dat moeten we misschien even aanpassen.

M: Ik hoef niet op bed te liggen en allerlei ".." 1:45:49.3

M: Uiteindelijk is wel alles relatief, hè, je kijkt toen, ik zeg maar ziek werd, eerst wil je gewoon leven en heb je nooit nagedacht over ziek worden, dan kom je voor het feit te staan dat je geopereerd moet worden, waarvan je niet weet hoe dat je eruit komt. En dan is het hebben van een stoma minder erg, ik heb het gelukkig niet. Ik kan me voorstellen als je 75 bent en je moet een been missen en je zit in een rolstoel, maar je kunt je kleinkinderen nog zien, dat is ook maar relatief. Als het op je 40ste gebeurt, dan ben je veel meer beperkt. Dus ik, ja.. dat moet je wel allemaal in perspectief zien. Als ik 93 ben, kijk als een kasplantje dat wil niemand, maar beperkt moeten leven en om je heen gebeuren nog leuke dingen, volgens mij dan ga je dat relativeren.

V2: Dan is het voor mij lang, maar met plezier willen leven.

M: Lang willen leven, moet relateren aan het leven op dat moment en de situatie op dat moment. Het moment eigenlijk.

V2: Ik denk dat ik me nog teveel laat tegenhouden met dat vind ik niet leuk of niet prettig.

Gesprekleidster 2: Nou, ik denk dat we hier een paar factoren uit kunnen halen die voor jullie belangrijk zijn. Geeft voor ons een goed overzicht op deze manier.

Afsluiting:
Gesprekleidster 1: Nou, heel mooi, dan zou ik ter afsluiting nog even graag een rondje willen maken waarin jullie kunnen aangeven of jullie nog vragen hebben of opmerkingen. Maakt niet uit wat. Zou je willen beginnen, kan ook zijn dat je niets meer te zeggen hebt. Ook prima.

V: ..

Gesprekleidster 1: M1?

M1: Nee, ik heb veel gehoord. Ik kom er nog goed vanaf als ik hierzo tussenzit. Waar ik wel, dit is een algemene lijst, ik ben wel geïnteresseerd wat nou specifiek voor mijn type afwijking een gezonde levensstijl is.

Gesprekleidster 2: Wij hebben daar wat boekjes liggen, daar staan 10 algemene in, maar ook 1 blokje specifiek voor darmkanker. Dat is darmkanker voor de algemene populatie, voor mensen met Lynch zou helemaal mooi zijn, die zijn er nog niet. Wordt wel onderzoek naar gedaan, maar dat is te weinig om echt aanbevelingen op te doen. Dus daarom wel in ieder geval die 2 boekjes mee.

Gesprekleidster 1: En er zit ook een mooi receptenboekje bij.

M: Ik vind het wel fijn om zo een avond bij elkaar te zitten, het is ook de eerste keer sinds ik weet dat ik 1:49:14.4 Lynch syndroom heb. Dit is de eerste keer dat ik echt een keer extra uitleg krijg. Je kunt op internet wel allemaal dingen gaan vinden, maar ja daar word ik ook niet happy van.

Gesprekleidster 1: Het is ook moeilijk, er staat zoveel informatie op internet. Het is net maar waar kom je terecht, en van welke bron komt dat af .. Lastig om dat onderscheid te maken.

M: Ja, daar zitten ook nog wel verschillende gradaties in 1:49:47.3

Gesprekleidster 1: M3?

M3: Niets meer toe te voegen

Gesprekleidster 1: V2?

V2: Ik heb ook niets toe te voegen.

Gesprekleidster 2: Dan kunnen we afronden, of wilde jij nog iets zeggen?

Gesprekleidster 1: Nee hoor, ik heb ook verder niets toe te voegen, lijkt me een mooi moment om af te sluiten.
